# Health-Related Quality of Life (HRQoL) Assessments in Research on Patients with Adult Rare Solid Cancers: A State-of-the-Art Review

**DOI:** 10.3390/cancers17030387

**Published:** 2025-01-24

**Authors:** Catarina S. Padilla, Cristiane D. Bergerot, Kim Dijke, Evelyne Roets, Gabriela Boková, Veronika Innerhofer, Samantha C. Sodergren, Rosanna Mancari, Cristiana Bergamini, Kirsty M. Way, Olga Sapoznikov, Jacobus A. Burgers, Daniel Dejaco, Margot E. T. Tesselaar, Winette T. A. van der Graaf, Olga Husson

**Affiliations:** 1 Department of Medical Oncology, The Netherlands Cancer Institute—Antoni van Leeuwenhoek, 1066 CX Amsterdam, The Netherlands; e.roets@nki.nl (E.R.); m.tesselaar@nki.nl (M.E.T.T.); w.vd.graaf@nki.nl (W.T.A.v.d.G.); o.husson@nki.nl (O.H.); 2 Department of Medical Oncology, Erasmus MC Cancer Institute, Erasmus University Medical Center, 3015 GD Rotterdam, The Netherlands; 3 Psycho-Oncology Services, Oncoclinicas & Co—Medica Scientia Innovation Research (MEDSIR), Sao Paulo 04543-906, Brazil; cristiane.bergerot@oncoclinicas.com; 4 Department of Gastrointestinal Oncology, The Netherlands Cancer Institute—Antoni van Leeuwenhoek, 1066 CX Amsterdam, The Netherlands; k.dijke@nki.nl; 5 International Accreditation Office, Masaryk Memorial Cancer Institute, 7656 53 Brno, Czech Republic; gabriela.bokova@mou.cz (G.B.);; 6 Department of Otorhinolaryngology-Head and Neck Surgery, Medical University of Innsbruck, 6020 Innsbruck, Austria; veronika.innerhofer@tirol-kliniken.at (V.I.); daniel.dejaco@i-med.ac.at (D.D.); 7 School of Health Sciences, University of Southampton, Southampton SO17 1BJ, UK; s.c.sodergren@soton.ac.uk (S.C.S.); k.m.way@soton.ac.uk (K.M.W.); 8 Gynaecologic Oncology Unit, IRCCS Regina Elena National Cancer Institute, 00144 Rome, Italy; rosanna.mancari@ifo.it; 9 Fondazione IRCCS Istituto Nazionale dei Tumori, 20133 Milan, Italy; cristiana.bergamini@istitutotumori.mi.it; 10 Department of Thoracic Oncology, The Netherlands Cancer Institute—Antoni van Leeuwenhoek, 1066 CX Amsterdam, The Netherlands; s.burgers@nki.nl; 11 Department of Surgical Oncology, Erasmus MC Cancer Institute, Erasmus University Medical Center, 3015 GD Rotterdam, The Netherlands; 12 Department of Public Health, Erasmus MC Cancer Institute, Erasmus University Medical Center, 3015 GD Rotterdam, The Netherlands

**Keywords:** adult rare solid cancers, health-related quality of life, systematic review, EURACAN

## Abstract

Background: Health-related quality of life (HRQoL) is an important patient-reported outcome for all cancer patients, including adult patients with rare solid cancers. However, current knowledge of HRQoL in this population is limited, which hinders the delivery of personalized care. This review aimed to explore the heterogeneity of HRQoL among adult patients with a solid rare cancer across the ten European Reference Network for Rare Adult Solid Cancers (EURACAN) domains and to summarize the HRQoL measures used in clinical research. Methods: A systematic literature search was conducted to identify all clinical studies assessing HRQoL in adult patients with a solid rare cancer. Four databases (MEDLINE, PubMed, PsycINFO, and Web of Science/Scopus) were searched (February 2023). Results: The search yielded 18,704 articles, of which 1416 articles were fully screened and 463 were eligible for analysis. Of these, 397 studies used generic tools to assess HRQoL, while 270 used tumor-specific instruments. Three EURACAN domains (sarcoma, endocrine tumors, and thoracic tumors) primarily assessed HRQoL using generic questionnaires. Additionally, the rare gynecological tumor, rare male genitourinary, and sarcoma EURACAN domains lacked specific HRQoL measures. Brain, head and neck, and uveal melanoma EURACAN domains used tumor- or domain-specific questionnaires in more than half of the studies. Conclusions: This state-of-the-art literature review shows that HRQoL assessment is gradually becoming more prevalent in adult solid rare cancer research. A combination of generic, tumor-specific, and domain-specific questionnaires across various rare cancer domains has proven effective in capturing a broad range of HRQoL issues. However, many EURACAN domains still lack specific strategies for assessing HRQoL, which limits the ability to fully understand and address patients’ experiences. Future research should prioritize developing comprehensive and robust HRQoL measurement strategies and tools to enable meaningful clinical research and to ensure that the patient voice is incorporated in their clinical care.

## Simple Summary

Rare cancers are known to be a group that suffer from a lack of information and delays in diagnosis. Patients often have an impaired health-related quality of life (HRQoL), with their lives being affected socially, mentally, and physically because of their diagnosis and/or treatment. The aim of our research was to unravel the heterogeneity of HRQoL in patients with a solid rare cancer per EURACAN domain, and to summarize the HRQoL measures used in research. A total of 464 articles investigated HRQoL in this population. Different measurement approaches were taken per EURACAN domain. Overall, this review shows that HRQoL measurement is slowly entering the field of clinical rare cancer research. Future research should invest in creating adequate HRQoL measurement strategies and/or HRQoL tools for use in solid rare cancer research and care to optimize patient outcomes.

## 1. Introduction

Rare adult solid cancers are characterized by an incidence of <6/100,000 people/year [1]. In the European Union (EU), around 500,000 new patients with rare adult solid cancer are diagnosed yearly, and more than 5 million people are living with a rare cancer diagnosis in the EU [2,3]. The heterogeneity of this population and the absence of a typical and uniform presentation make it difficult to diagnose and treat these patients [1]. Additionally, healthcare professionals’ limited experience with rare adult solid cancers often results in diagnostic delays, inappropriate treatment, and late referrals to centers of expertise. These factors negatively impact patient clinical outcomes [1,4,5,6].

The European Reference Network for Rare Adult Solid Tumours (EURACAN) is the main body for rare adult solid cancer care and divides rare adult solid cancer diagnoses into 10 clinical domains [7]. By splitting rare adult solid cancers into these domains, EURACAN aims to better address each group’s specific needs and challenges, connecting and integrating the different national healthcare systems across Europe, alongside facilitating the creation of guidelines for managing rare adult solid cancer [3].

Beyond the diagnostic challenges and limited clinical expertise, patients diagnosed with rare adult solid cancer frequently experience poorer psychosocial outcomes and an impaired health-related quality of life (HRQoL) compared to patients with more common cancer types [6,8]. Unfortunately, patient-reported outcomes (PROs) like HRQoL are still not routinely incorporated into the care or research of rare adult solid cancers [9,10]. Limited research has shown that patients with adult rare solid cancer diagnoses experience higher levels of distress compared to the general cancer population. Additionally, they report lower HRQoL [8,11].

Due to its heterogeneity, HRQoL in patients with a rare adult solid cancer is often evaluated by generic tools such as the EORTC QLQ-C30 questionnaire, which lacks content validity for this population and limits the full understanding of the precise HRQoL challenges this specific group of patients face [12]. Creating tumor-specific HRQoL tools is typically the solution for the issue of a generic measure’s inadequate content coverage [6,12]. However, developing a single questionnaire to assess HRQoL for all rare adult solid cancers may not be feasible. This is due to the immense diversity in tumor types, locations, and the variety of treatments applied [6,12]. Therefore, it is essential to understand the variances in HRQoL between patients with rare adult solid cancers to come to an optimal measurement of the patient perspective in clinical research and care [9].

Consequently, the aim of this review is to explore the heterogeneity of HRQoL measurement strategies and tools among patients with rare adult solid cancer across EURACAN domains and to summarize the HRQoL used in clinical research.

## 2. Materials and Methods

This review followed the Preferred Reporting Items for Systematic Reviews and Meta-Analyses (PRISMA) guidelines [13]. This review has not been registered. The full study protocol is published online [6].

In order to provide an overview of the current HRQoL instruments used in the literature, we made use of the EURACAN domain division [3,7,14] (Table 1). Two medical doctors (D.v.d.W and I.P) reviewed the categorization of tumor location within domains.

### 2.1. Search Strategy

A systematic literature search was performed by C.S.P via four search engines—MEDLINE, PubMed, PsycINFO, and Web of Science/Scopus—on 08 February 2023, combining terms focused on adult rare solid cancer (e.g., neoplasms, cancer, and tumor), specific EURACAN domains and subtypes, and HRQoL. A librarian from the Netherlands Cancer Institute (S.v.d.M) assisted in creating the search string. The search string was based on terms related to HRQoL, adult rare solid cancers, and questionnaires and instruments that assess HRQoL. EURACAN domains were used as the foundation for categorizing tumor subtypes, as described in the RARECARE net classification database [2]. The entire search string is presented in Supplementary File S1.

### 2.2. Selection Process

Literature published before 2011 was excluded, as the definition of rare cancers was published and adopted in the European Union in 2011 [1,15]. Both quantitative and qualitative studies were considered for inclusion if they met the following criteria:

1. Included one (or more) type(s) of solid rare cancer;
2. HRQoL was described as the study outcome;
3. Published in English;
4. Included patients of adult age (i.e., >18 years), regardless of cancer site and stage of disease.

Studies were excluded if they met the following criteria:

5. Were case studies, small case series, conference posters, or abstracts;
6. The study sample included a combination of mixed rare and common cancer types without separate results for rare cancer types;
7. The population consisted of children or adolescents (<18 years), and no separate analyses were presented;
8. Other reasons for ineligibility (e.g., full text not available).

**Figure 1:**
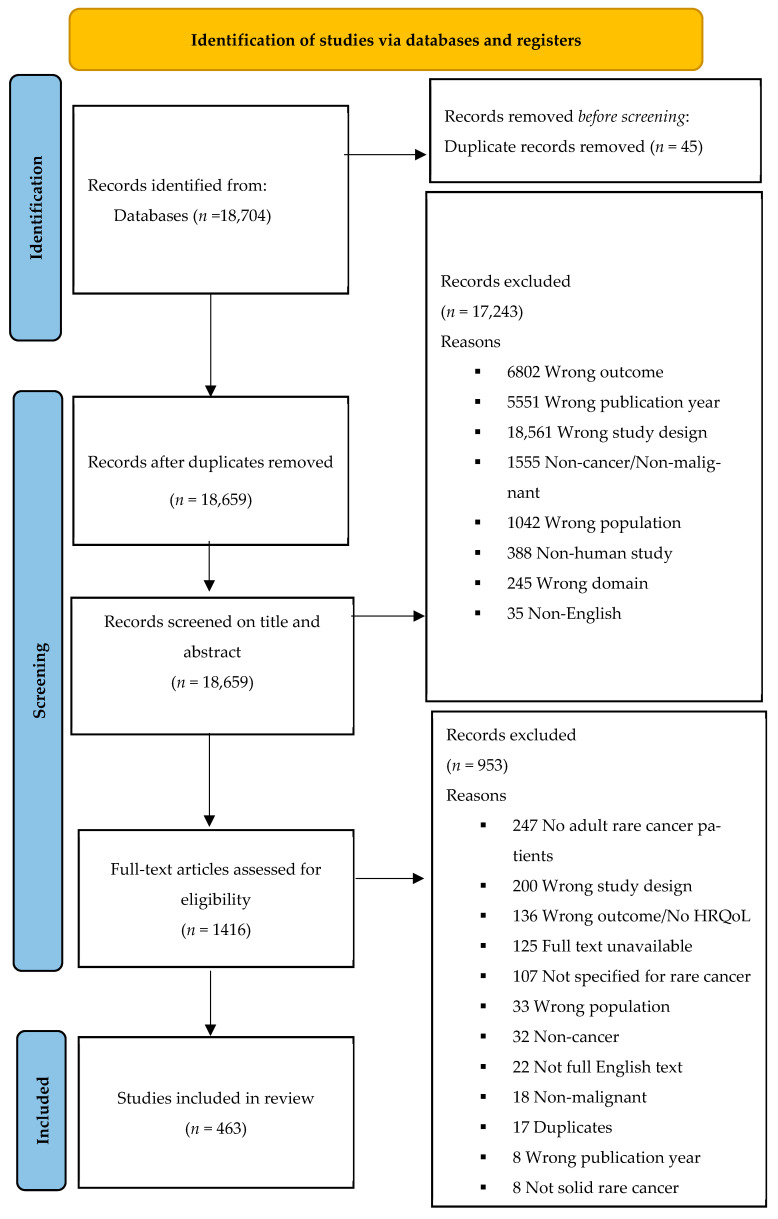
PRISMA diagram.

All titles and abstracts were screened by three independent reviewers (C.S.P., K.D., and E.R.) to determine eligibility. Papers selected by all reviewers advanced to the full-text review phase, while papers with differing evaluations were discussed to reach a consensus. After this selection, full-text articles were retrieved and screened. Eleven reviewers (C.S.P., S.S., C.B., C.B., V.I., D.D., O.S., G.B., J.B., K.W., and R.M.) examined the full texts and extracted all relevant data according to the predefined protocol. Reviews were not included in the final dataset but were used for cross-referencing purposes only. The reference lists of relevant articles were also checked to identify additional studies. The PRISMA diagram and number of included articles are presented in Figure 1.

## 3. Results

**Figure 2:**
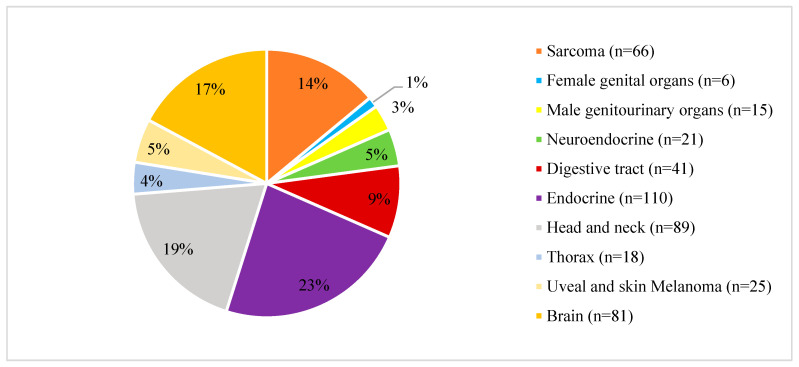
Distribution of articles per EURACAN domain.

The literature search identified 18,704 articles. Of these, 6802 abstracts were excluded due to the absence of HRQoL assessments. An additional 5551 abstracts were excluded for being published before 2011. A total of 1416 articles were reviewed fully. Of these, 463 were deemed suitable for inclusion. Figure 2 presents the number and percentage of articles per EURACAN domain.

**Figure 3:**
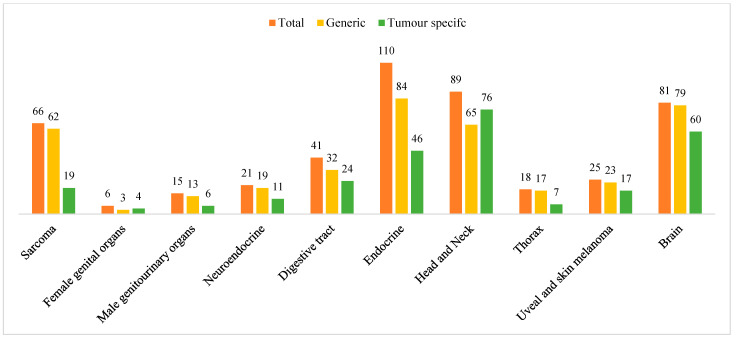
Distribution of HRQoL assessments across EURACN domains.

The majority of the identified studies were quantitative (*n* = 449), and 26 were qualitative (either as a part of a mixed methodology or purely qualitative). Generic questionnaires (397 out of 463 studies), such as the European Organisation for Research and Treatment of Cancer Quality of Life Questionnaire-Core 30 (EORTC QLQ-C30), the 36-Item Short Form Health Survey (SF-36), the EuroQol 5-dimensions or 3-dimensions (EQ-5D-5L/3L), and Functional Assessment of Cancer Therapy—General (FACT-G), were commonly used most often to assess HRQoL [16,17,18,19]. Additionally, 270 studies used a tumor-specific questionnaire, either as a supplement to a generic questionnaire or as the HRQoL instrument. The distribution of generic and tumor-specific HRQoL assessment, both overall and per EURACAN domain, is shown in Figure 3. A complete table of all included studies, organized by EURACAN domain, can be found in Supplementary File S2.

### 3.1. HRQoL of Patients with a Rare Cancer of the Connective Tissues (Sarcomas) (EURACAN G1)

Sixty-six studies examined HRQoL in patients with sarcoma (Supplementary Table S2.1). Six studies had a qualitative or mixed-method design and used interviews to assess HRQoL. All six studies reported physical issues such as pain, fatigue, and sleep disturbances, alongside psychosocial issues, including fear of cancer recurrence, uncertainty about the future, loss of independence, limitations in work and social life, and body image changes [20,21,22,23,24,25]. More specifically, patients with uterine sarcoma reported issues with urological, gastrointestinal, and sexual problems and missed HRQoL issues with urination and wheezing [25], patients with a thoracic sarcoma often reported difficulty with respiratory function, patients with breast sarcoma displayed tightness and limitations with tasks needing their arms [24], and patients with metastatic synovial sarcoma mentioned issues with the ability to undertake strenuous activities, disrupted sleep, and shortness of breath [20].

Sixty-two studies employed a generic instrument, such as the EORTC QLQ-C30 (*n* = 42), the FACT-G (*n* = 4), the SF-36 (*n* = 4), the EQ-5D-5L (*n* = 4; assessing issues like mobility, pain, discomfort, and anxiety), the World Health Organization Quality of Life Scale (WHOQOL-BREF; *n* = 1; assessing physical, psychological, and environmental health and social relationships), the Greek version of the M.D. Anderson Symptom Inventory (G-MDASI) (*n* = 1), or the Quality of Life Cancer Survivor Scale (QoL-CS) (*n* = 1), most often assessing general domains like physical, social, emotional, and psychological well-being according to the biopsychosocial model.

In addition, 19 studies used a domain-specific questionnaire, either the Toronto Extremity Salvage Score (TESS; assessing physical disability for patients undergoing surgery for extremity tumors), the Disability of the Arm, Shoulder and Hand (DASH; assessing the ability to perform certain upper extremity activities and the interference with that in their daily life with items like difficulties in using a knife or pushing a heavy door), the Lower Extremity Functional Scale (LEFS; assessing the difficulty, because of a lower limb problem, of performing activities like putting shoes on or walking), or the Short Musculoskeletal Function Assessment Questionnaire (SMFA; investigating scales like upper extremity dysfunction, problems with daily activities, and mental and emotional problems). Additionally, tumor-specific questionnaires were used, including the Musculoskeletal Tumour Society Score (MSTS; assessing functional outcomes in patients with neoplasms in the upper or lower limbs), the EORTC QLQ-Endometrial Cancer Module (EORTC QLQ-EN24; investigating HRQoL with scales like sexual enjoyment, pelvic pain, and body image problems), or the Functional Assessment of Cancer Therapy—Endometrial (FACT-EN; items such as vaginal discharge, cramps in the stomach area, or constipation) when investigating gynecological sarcomas.

A total of 17 studies used a combination of a generic and tumor- or domain-specific questionnaires. In these studies, when investigating soft tissue sarcomas (STSs) and bone sarcomas, combinations of questionnaires varied between one of the generic questionnaires (EQ-5D-5L, EORTC QLQ-C30, SF-36, the World Health Organization-Five Well-Being Index (WHO-5), and QoL-CS) and the tumor- and domain-specific questionnaires (TESS and MSTS). All studies identified HRQoL issues related to functional disabilities (e.g., pain, fatigue, sleep disturbances, reduced strength, and poorer mobility) and their impact on daily activities (e.g., showering, carrying objects, gardening, leisure activities). Additionally, some studies found psychosocial HRQoL issues, such as anxiety, higher preoccupation, lack of motivation, and body image insecurities [26,27,28].

For uterine sarcomas, the measurement of studies used either the FACT-G together with FACT-EN or EORTC QLQ-C30 and EORTC QLQ-EN24. These studies identified HRQoL issues related to urological and sexual functions [25]. Finally, when investigating patients with GIST, the Gastrointestinal Quality of Life Index (GIQLI) was used as the tumor-specific questionnaire, and HRQoL issues related to weight, reflux, and coughing were found.

In summary, six studies investigated HRQoL using qualitative methods. A total of 62 studies utilized cancer-specific generic questionnaires, while 17 employed both tumor- and domain-specific tools. Only 1 study solely used a domain-specific questionnaire, and 44 studies assessed HRQoL with only generic tools.

### 3.2. HRQoL of Patients with a Rare Cancer of the Female Genital Organs and Placenta (EURACAN G2)

Six articles involving patients with a rare cancer of the female genital organs and placenta explored HRQoL as their primary objective, with only one utilizing a qualitative approach (Supplementary Table S2.2). In the qualitative study [29], patients with vaginal and vulvar carcinoma were interviewed to assess how their psychosocial functioning was affected. The issues identified were related to their diagnostic trajectory (e.g., lack of information and knowledge about the rare cancer type), recognition of symptoms (e.g., pain and loss of blood being attributed to common diseases rather than indicating a possible cancer diagnosis), and difficulty in addressing sensitive topics (e.g., sexuality) due to the tumor’s location and associated taboos [29].

Two studies employed the generic SF-12 questionnaire alongside the Female Sexual Function Index (FSFI) [30,31]. The FSFI assesses sexual feelings such as sexual intercourse, stimulation, and desire in vulvar cancer patients. Nevertheless, the SF-12 and FSFI do not capture tumor-specific alterations in sexual sensations. The HRQoL issues identified in these studies were mainly related to physical symptoms (e.g., lymphadenectomy and lymphedema) that impact the patients’ sexual function [31].

One study used a tumor-specific questionnaire, the FACT-Ovarian (FACT-O), to evaluate physical and emotional well-being among ovarian cancer patients. HRQoL issues identified included physical and functional well-being (e.g., weight loss, appetite loss, change in appearance, pessimism, and blood loss) [32,33]. Lastly, one study selected patients with ovarian or uterine cancers and used specific items from the Core Notebook questionnaire, which was designed for oncological outpatients undergoing either active treatment or palliative care [34,35]. This study primarily assessed the physical domain, with the most significant issues related to symptom control and its correlation with radiological findings [35].

Finally, one study focused on patients with neuroendocrine carcinoma of the cervix, an ultra-rare group of cervical cancer patients [36]. This study employed the tumor-specific FACT-Cervix (FACT-Cx), which assesses HRQoL in women with cervical cancer. It includes scales such as for emotional and functional well-being, as well as broader items related to discomfort during urination, interest in sex, and vaginal odor. The sexual function domain from the PROMIS scale was also used, assessing aspects such as interest in sexual activity, lubrication, and overall sexual satisfaction. Results indicate that most HRQoL issues were found in the emotional well-being of patients rather than in sexual function [36].

In summary, one study employed a qualitative approach to investigate HRQoL, while five studies used quantitative methods. Of the five, three utilized generic HRQoL questionnaires, primarily assessing domains within the biopsychosocial model. Two articles used domain-specific questionnaires, and two used a tumor-specific questionnaire.

### 3.3. HRQoL of Patients with a Rare Cancer of the Male Genital Organs and the Urinary Tract (EURACAN G3)

A total of 15 studies examined HRQoL in patients with testicular tumors, germ-cell tumors, or penile tumors (Supplementary Table S2.3). Two qualitative interview studies were conducted showing the impact of diagnosis and treatment on the patient’s physical and mental health (e.g., body image, sexuality, urinary limitations, and masculinity) when having penile cancer [29,37]. One study highlighted physical symptoms that interfered with daily life, such as itchiness, pain, visible scars, reduced mobility, and urinary issues, alongside psychosocial issues like lack of sexual gratification, feelings of emasculation, and coping mechanisms [37]. Meanwhile, the other study primarily focused on body image (e.g., feelings of mutilation) and sexuality (e.g., inability to engage in sexual intercourse) [29].

Thirteen articles used generic HRQoL questionnaires, such as the EORTC QLQ-C30, SF-36, the Patient-Reported Outcomes Measurement Information System (PROMIS; assessing physical activities like walking and engaging in daily tasks, anxiety, and fear), the EQ-5D-3L (assessing mobility, self-care, pain/discomfort, and anxiety/depression), and FACT-G. These questionnaires focused on social, physical, and emotional well-being within the biopsychosocial model. Additionally, six studies used more specific questionnaires. Two domain-specific questionnaires were used to investigate issues regarding erectile function and incontinence in patients with testicular and penile cancer, namely the Index of erectile function (IIEF-15; assessing the effect of erection problems on the patient’s sex life) and the International Consultation on Incontinence Modular Questionnaire for Male Lower Urinary Tract Symptoms (ICIQ-MLUTS; evaluating the impact of urinary tract symptoms on the quality of life with items on strength of stream, frequency, and urgency). In addition, one study employed the tumor-specific EORTC QLQ testicular module (EORTC QLQ-TC26), which evaluated treatment-related issues as well as future perspectives, sexual activity, functioning, and enjoyment [38].

In studies investigating germ-cell tumors, six studies used different generic questionnaires depending on their specific population cohorts (e.g., cancer survivors, testicular germ-cell patients, patients receiving surgery alone or surgery and systemic therapy), and one employed a domain-specific questionnaire, the Aging Males’ Symptom scale (AMS; assessing symptoms related to male aging, such as psychological well-being (e.g., depression), sexual function (e.g., libido), and somatic complaints) [39]. Five studies investigating either age, time since diagnosis, or impact of surgery on patients did not find an association with HRQoL issues from the questionnaires used in their patient populations, even though different questionnaires were used to assess HRQoL [40,41,42,43,44]. In contrast, one study found that patients undergoing retroperitoneal lymph node dissection presented a lower score on their emotional well-being throughout their trajectory compared to patients receiving chemotherapy [40].

For penile cancers, two studies investigated HRQoL with a quantitative approach using generic questionnaires. The EQ-5D-3L was used to assess HRQoL with scales such as mobility, self-care, usual activities, pain/discomfort, and anxiety/depression in a more general way, and the two domain-specific questionnaires IIEF-15 and the ICIQ-MLUTS focused on erectile function and incontinence issues [45,46]. One study did not report differences in HRQoL when investigating functional outcomes after organ-sparing reconstructive surgery [45]. The second study noted that a longer gap between surgery and questionnaire completion could introduce recall bias and that different disease stages and reconstructive procedures might impact HRQoL [46].

Four studies used the EORTC QLQ-C30 in patients with testicular cancer as the generic questionnaire, with scales such as emotional, social, and physical well-being. When looking at the tumor-specific questionnaire, one study used the EORTC QLQ-TC26, and two used the domain-specific questionnaire for erectile function, the IIEF-15 [38,47,48]. However, the IIEF-15 is only applicable if the patient is sexually active.

In summary, 2 studies used a qualitative methodology, while 13 employed quantitative methods. All quantitative studies described HRQoL using generic questionnaires, and six also utilized domain-specific questionnaires focusing mainly on erectile function and incontinence.

### 3.4. HRQoL of Patients with a Rare Neuroendocrine Tumor (EURACAN G4)

Twenty-one studies investigated HRQoL in patients with neuroendocrine cancer (NET) in the lungs, digestive tract, or pancreas (Supplementary File Table S2.4). Only one study used a qualitative approach and found that HRQoL deteriorated in areas such as treatment-related toxicities, physical symptoms such as stomatitis and pneumonitis, and changes in or discontinuation of treatment in patients with advanced NET. The authors concluded that the stage of the disease significantly impacts HRQoL and should always be considered [49].

In the quantitative studies, a notable trend emerged, with nine studies combining two EORTC instruments: the generic EORTC QLQ-C30 and the tumor-specific EORTC QLQ-GI.NET21, which focuses on muscle and/or bone pain, endocrine and gastrointestinal symptoms, and treatment-related effects (Supplementary Table S2.4).

Of the 21 studies, 15 used the EORTC QLQ-30 to assess HRQoL, while only 4 employed a different generic questionnaire [50,51,52,53]. The FACT-G scale was used in two studies [51,53], while the visual analogue scale (VAS; assessing pain intensity) and the EQ-5D-5L were each used in one study [50,52]. The study that used the EQ-5D-5L instrument found that HRQoL issues may vary daily, and due to tumor grade. For example, they showed that issues related to pain and discomfort impacted patients in all NET grades, while feelings of anxiety and depression were seen more in patients with a grade 2 tumor than in patients with a grade 1 tumor [52].

There is a noticeable gap in tumor-specific HRQoL assessments. Eleven studies used the EORTC gastroenteropancreatic NET-specific tool, the EORTC QLQ-GINET21. However, patients with pancreatic NET were not assessed with a tumor-specific questionnaire [54].

In summary, 21 studies assessed HRQoL in patients with NETs. Only 1 study used a qualitative approach, while 19 used a generic questionnaire. The EORTC QLQ-C30 was used in 15 of these studies. Of the articles using generic tools, 12 also employed a tumor-specific questionnaire, with 11 using the EORTC QLQ-GINET21.

### 3.5. HRQoL of Patients with a Rare Cancer of the Digestive Tract (EURACAN G5)

Forty-one studies reported HRQoL of patients with rare cancer in the digestive tract (Supplementary Table S2.5). Five studies qualitatively investigated HRQoL through interviews [29,55,56,57]. One qualitative study involving patients with biliary tract cancer (BTC) found that, beyond physical HRQoL issues (e.g., insomnia, fatigue, difficulty walking), patients experienced additional psychosocial impacts, including trouble meeting the needs of the family, difficulty with self-care, inability to travel or work, and financial burden [55]. Qualitative studies on anal cancer patients revealed a wide range of HRQoL issues. These included psychosocial concerns such as reactions to diagnosis (e.g., shame about the tumor’s location, shock, and misleading initial symptoms), and physical impacts from the disease and treatment (e.g., skin damage, hair loss, scars, and late toxicities from chemoradiation therapy affecting sexual, urinary, and bowel function). Social concerns included fears of remaining single due to the diagnosis’s characteristics and consequences [29,56,57,58,59].

Seventeen articles described HRQoL in patients with anal cancer with a quantitative approach. The choice for using generic questionnaires, which assessed domains like physical, functional, and emotional well-being, varied between the EORTC QLQ-C30 (*n* = 11) and the FACT-G (*n* = 3), and one study used the Patient’s Global Impression of Change Scale (PGIC, with a single scale on how their condition changed since a specific time point) [58]. One study solely used the EORTC QLQ-C30 to assess HRQoL, together with self-created questions to identify missing bodily function-related issues (e.g., bowel and urinary) [60]. In addition, in the study using the FACT-G tool, HRQoL issues focused on receiving insufficient information about the disease and inadequate support in managing late toxicities [59].

Fifteen studies used a tumor-specific or domain-specific questionnaire to investigate HRQoL. The selected EORTC tumor-specific questionnaires included the anal questionnaire (EORTC QLQ-ANL27; assessing issues such as pain, bowel, and sexual problems [61]) and the colorectal-specific questionnaire, EORTC QLQ-CR38 and the updated EORTC QLQ-CR29 (assessing HRQoL issues like urinary frequency, blood or mucus in the stool, stool frequency, and body image [62]). The primary HRQoL issues identified with the EORTC QLQ-ANL27 were sexual problems [61]. When the EORTC QLQ-CR38 or EORTC QLQ-CR29 was chosen, the majority of studies identified HRQoL issues with pain (e.g., buttocks), sexual dysfunction (e.g., impotence, low sexual interest, dyspareunia), and urinary function (e.g., urinary frequency and incontinence), but mainly bowel and gastrointestinal function (e.g., diarrhea, controlling flatus, fecal leakage, stool frequency, fecal incontinence) [63,64,65]. Finally, social functioning issues were found with respect to social life (e.g., limitations due to gastrointestinal symptoms) and mood changes [66]. Two additional tumor-specific questionnaires were used in two other studies, including the FACT-C (with generic scales such as social/family and physical well-being combined with specific items related to control of bowels, appetite, and digestion) where HRQoL issues included appearance, bowel and sexual function, and the difficulty in talking about symptoms [67,68], and the ANCHOR Health-Related Symptom Index (A-HRSI; assessing HRQoL for patients with high-grade anal cancer), where issues including pain, intimate relationships, sitting, and moving around were identified [58]. Finally, one study made use of the FACT-G7, together with the domain-specific questionnaires IIEF-15, ICIQ-MLUTS, ICIQ-FULTS, and FIQoL (assessing erectile function, incontinence issues, female lower urinary tract symptoms, and fecal incontinence), and found issues related to difficulty controlling flatus and frequency and urgency of bowel movements [69].

Another 18 studies investigated HRQoL in patients with BTC and cholangiocarcinoma (CCA). Generic HRQoL issues were assessed with the EORTC QLQ-C30, the FACT-G, or the SF-36, all focusing on issues from the biopsychosocial model. Most studies using only generic tools did not address specific HRQoL issues. However, one study found that the effects of symptoms, uncertainty around the future, and social support impacted HRQoL in patients with CCA [70]. In the studies using a tumor-specific tool, the FACT-Hep (evaluating specific issues for hepatobiliary patients like itchiness, constipation, control of bowels, and discomfort or pain in the stomach area) was most frequently chosen to measure HRQoL, being used in five different studies (Supplementary Table S2.5). The main issues were found to be unmet needs, uncertainty about treatment effectiveness, and support care [71,72]. One study investigating CCA used the EORTC QLQ Cholangiocarcinoma module (EORTC QLQ-BIL21; assessing scales such as eating and jaundice symptoms and pain and anxiety symptoms along with three single-item assessments on drainage bags and weight loss) and found HRQoL issues with weight loss and drain sites [73].

To summarize, 5 studies investigated HRQoL using a qualitative approach, while 36 studies used a quantitative methodology. Of these, 32 studies used a generic questionnaire, differing mainly between the EORTC QLQ-C30, the FACT-G, and the SF-36. In addition, 24 studies assessed HRQoL by means of a tumor-specific questionnaire, which varied according to the tumor population of the investigation. Finally, five articles solely used a tumor-specific tool to assess HRQoL in patients with rare cancer in the digestive tract.

### 3.6. HRQoL of Patients with a Rare Endocrine Tumor (EURACAN G6)

One hundred and ten studies assessed HRQoL in patients with an endocrine tumor (Supplementary Table S2.6). Of these, 108 investigated HRQoL in patients with a thyroid cancer diagnosis, and 2 focused on HRQoL in patients with adrenocortical carcinoma (ACC) [74,75]. Six studies investigated HRQoL using a qualitative approach.

In the two studies focusing on patients with ACC, one used interviews to investigate HRQoL and identified issues in four domains: physical complaints (e.g., abdominal pain, nausea, fatigue, gastrointestinal symptoms, itchy skin, and wound healing), mental consequences (e.g., insecurity and irritation), social consequences (e.g., difficulty performing household tasks and caregiving), and functional limitations (e.g., restrictions on hobbies, social events, and mobility) [75]. The other study investigated HRQoL by validating an ACC tumor-specific questionnaire (ACC-QOL; assessing items such as emotional effects, physical limitation due to surgery, need for peer support, and mitotane side effects) and found that fatigue, impact of disease on sexual life, and emotional problems were the main HRQoL issues [74].

Five articles used qualitative methods to assess HRQoL in thyroid cancer patients. In those studies, issues were reported with physical aspects (e.g., fatigue, pain, sleep disturbances, voice changes and numbness, brain fog), with psychological and emotional problems (e.g., feelings of shock, distress, frustration, fear of recurrence, uncertainty, mental exhaustion, and mood swings), and in the patient’s social life and daily activities (e.g., inability to exercise, limitation at work due to cognitive issues, lack of support from friends and family) [76,77,78,79,80].

Using a quantitative approach, 103 studies investigated HRQoL in patients with thyroid cancer. A total of 83 studies used a generic questionnaire, and 45 used a tumor- or domain-specific one. Fifty-nine studies solely used a generic questionnaire measuring HRQoL more broadly with physical, psychosocial, emotional, and social well-being items. The options ranged between the SF-36/12 (*n* = 20), the EORTC QLQ-C30 (*n* = 24), the FACT-G (*n* = 2), the EQ-5L-5D/3D (*n* = 3), the GQOLI (*n* = 1), the PROMIS-29 (*n* = 5), the DLQI (*n* = 1), the WHO-QoL BREF (*n* = 1), the SOMS-7 (*n* = 1), the FACIT (*n* = 1), and the HUI-12/13 (*n* = 1).

Additionally, 24 studies used a generic questionnaire (either the EORTC QLQ-C30 or the SF-36) alongside a tumor- or domain-specific questionnaire, such as the EORTC QLQ Thyroid module (EORTC QLQ-THY34; *n* = 3; assessing muscle cramps, pain, arm raising limitations, restlessness, and dry mouth), the Voice Handicap Index (VHI; *n* = 1; evaluating voice impairment), and the THYroid CANcer-Quality of Life (THYCA-QOL; *n* = 11; assessing throat and mouth issues, voice, and neuromuscular and sensory problems), the Patient and Observer Scar Assessment Scale (POSAS; *n* = 1; evaluating scar quality by visual, tactile, and sensory characteristics), the City of Hope Quality of Life-Ostomy Questionnaire (COH-QOL-THY; *n* = 1; examining items like fatigue, dry skin, weight gain, and feelings of isolation), the EORTC QLQ-GINET21 (*n* = 1), the Billewicz Scale (*n* = 2; evaluating hypothyroidism’s clinical aspects such as coarse skin, hoarseness, diminished sweating, and weight gain), the Disabilities of the Arm, Shoulder and Hand (DASH; *n* = 1; evaluating the patient’s ability to perform certain activities with their arm, shoulder, and hand), or the EORTC QLQ Head and Neck module (EORTC QLQ-H&N35; *n* =3; assessing HRQoL issues specifically relating to the head and neck region).

Twenty-one studies exclusively used tumor- or domain-specific questionnaires. For the tumor-specific questionnaire, options included the EORTC QLQ-H&N35/H&N43 (*n* = 3), the COH-QOL-THY (*n* = 2), the University of Washington Quality of Life Questionnaire (UW-QOL; *n* = 4, assessing issues such as pain, appearance, swallowing, chewing, taste, and anxiety), the THYCA-QOL (*n* = 2), the Quality Of Life in Thyroid Cancer (QOL-TV; *n* = 1; assessing items on physical, social, and emotional well-being), and the Thyroid-specific patient-reported outcome (THY-Pro; *n* = 2; HRQoL items on physical symptoms like goiter and hyperthyroid symptoms, tiredness, and mental health symptoms like anxiety, cognitive impairments, and impact of thyroid disease on social, daily, and sex life, and cosmetic complaints). Overall, HRQoL issues were mainly physical, such as tiredness, sensory change, dry eyes and skin, itchy skin, pain, voice change, tingling and numbness, hair loss, and brain fog. Additionally, issues like emotional changes, the fear of cancer recurrence, the impact of receiving a diagnosis, and the uncertainty of the future and test results also appeared.

When opting for a domain-specific questionnaire, choices were either the Xerostomia Quality of Life Scale questionnaire (XeQOLS; *n* = 1, assessing the impact of salivary gland dysfunction on physical, social, and psychological functioning and pain issues), the VHI (*n* = 3), the Swallowing Impairment Score (SIS-6; *n* = 2, assessing the impact of swallowing on a patient’s life), the Neck Dissection Impairment Index (NDII; *n* = 1; assessing the impact of major neck dissection with items like pain, stiffness, and limitation in lifting objects), or the Billewicz scale (*n* = 1). The main HRQoL issues were sensory issues and voice change, swallowing discomfort, pain, and salivary morbidity.

To summarize, 6 studies investigated HRQoL qualitatively, while 21 used a quantitative approach with only a tumor- or domain-specific questionnaire. A total of 83 studies used a generic HRQoL questionnaire, and 45 used a tumor- or domain-specific questionnaire. Additionally, 24 studies combined both generic and tumor-specific questionnaires.

### 3.7. HRQoL of Patients with a Rare Cancer of the Head and Neck (EURACAN G7)

Eighty-nine articles investigated HRQoL in patients with rare cancer in the head and neck region (Supplementary Table S2.7). Two studies used a mixed-method approach, employing interviews and questionnaires. Findings revealed that the main HRQoL issues were emotional distress (e.g., anxiety, irritability, fear, and uncertainty), physical issues (e.g., voice and speech problems, tracheal secretion, fatigue, neck and shoulder stiffness, and weight loss), changes in role functioning and social functioning (e.g., decreased family contacts, isolation, social eating, and decreases in outdoor activities) [81,82].

A total of 65 articles assessed HRQoL with generic domains (e.g., physical domain, well-being domain, and psychosocial domain) with questionnaires such as the EORTC QLQ-C30 (*n* = 48), the SF-36 (*n* = 4), the EQ-5D-3L (VAS) (*n* = 5), the 15D (*n* = 2, assessing items like mobility, breathing, eating, speech, distress, and mental function), the FACT-G (*n* = 3), the Brief Symptom Inventory (BSI, *n* = 1), or the Bochum questionnaire (*n* = 1). In the studies that exclusively used generic questionnaires (EORTC QLQ-C30, *n* = 8; 15D, *n* = 2; SF-36, *n* = 1; VR-12, *n* = 1; Bochum questionnaire, *n* = 1; and a self-developed questionnaire), HRQoL issues were primarily related to fatigue, xerostomia, pain, eating problems (e.g., swallowing, tongue mobility, restrictions due to eating), mouth dryness or sores, dysphagia, and mental well-being (e.g., anxiety and depression) [83,84,85].

In addition, 23 studies employed only a tumor- or domain-specific questionnaire. Tumor-specific questionnaires included the UW-QOL (*n* = 17; assessing items on pain, appearance, swallowing, mood, chewing, employment, and taste), the EORTC QLQ-H&N35 (*n* = 2; assessing pain, speech problems, dry mouth, sticky saliva, coughing, and trouble with social eating and social contact), the Performance Status Scale for Head and Neck Cancer Patients (PSS-HN, *n* = 1), FACE-Q Head and Neck Cancer (FACE-Q, *n* = 1, assessing items like facial appearance, oral competence, salivation, smiling, speaking, eating distress, and speaking distress), the FACT head and neck questionnaire (FACT-H&N, *n* = 2; items like ability to eat solid foods, communication with others, voice quality, pain in mouth), the Vanderbilt Head and Neck Symptom Survey (VHNSS, *n* = 1, assessing items like swallowing solids, swallowing liquids, nutrition, mucous, dry mouth, voice, and teeth), and the Head and Neck Quality of Life Instrument (HNQOL, *n* = 1; assessing domains like eating, communication, and pain). Looking at the domain-specific questionnaires, studies opted for the Sino-Nasal Outcome Test 22 Questionnaire (SNOT-22, *n* = 2; items like nasal blockage, sneezing, ear fullness, need to blow nose, and ear pain), the Oral Health Impact Profile Questionnaire (OHIP, *n* = 1, assessing physical pain, psychological limitations, and social and function limitations), the Eating Assessment Tool (EAT-10, *n* = 1, with items related to swallowing aspects), the VHI (*n* = 1), the Xerostomia Index (XI, *n* = 1), and the Speech Handicap Index (SHI, *n* = 1) (Supplementary Table S2.7). Overall, HRQoL issues included pain, chewing, saliva, swallowing, social contact, senses, and rhinology symptoms such as mucosal dryness alongside xerostomia, dysphagia, and sleep disturbances [86,87,88,89].

Finally, 52 studies used both generic and tumor- or domain-specific tools to assess HRQoL, with 42 articles combining the EORTC QLQ-C30 and EORTC QLQ-H&N35/H&N43. HRQoL issues commonly found in most studies were dry mouth and sticky saliva, fatigue, swallowing problems, speech difficulties, mouth opening, sensory problems (hearing loss, difficulty with taste and smell), weight gain/loss, appetite loss, social functioning (social contact, social eating, trouble with eating), emotional well-being (feelings of isolation, depression, and loneliness), pain, coughing, and facial disfigurement. One study mentioned that even though the EORTC QLQ-H&N35 is a tumor-specific questionnaire, it lacks items on how to deal with adverse radiation effects [90].

To summarize, 89 studies investigated HRQoL in patients with rare head and neck cancer. Among these, 2 studies used a qualitative approach, while 65 employed a generic cancer-specific questionnaire, most often assessing domains based on the biopsychosocial model. Of those, 52 also used a tumor- or domain-specific questionnaire. Specifically, 68 studies used a tumor-specific questionnaire, while 16 used a domain-specific one. Finally, 23 articles only employed a tumor- or domain-specific tool.

### 3.8. HRQoL of Patients with a Rare Cancer of the Thorax (EURACAN G8)

Eighteen articles described HRQoL in patients with rare cancer in the thorax area (Supplementary Table S2.8). None of the studies used a qualitative approach to investigate HRQoL. Of the 18 articles, only 1 focused on patients with a thymoma diagnosis, using two generic questionnaires, the EQ-5D-3L and the Edmonton Symptom Assessment Scale (ESAS), which evaluates common cancer-related symptoms such as pain, nausea, drowsiness, appetite, and shortness of breath [91].

The remaining 17 studies investigated HRQoL in patients with a mesothelioma diagnosis with a quantitative approach. Generic HRQoL questionnaires covering broader domains, like physical, emotional, and social well-being, were mainly assessed with the SF-36, EORTC QLQ-C30, and EQ-5D-3L. Other generic questionnaires, such as the WHO QOL-BREF and the Comprehensive Quality of Life Outcome (CoQoLo), which assesses aspects like physical and psychological comfort, independence, and good family relationships, were also used. The most common HRQoL issues across studies related to dyspnea, physical symptoms (e.g., pain, fatigue), and lung function. Only one study did not use a generic questionnaire and opted to use a tumor-specific tool alone, the Lung Cancer Symptom Scale (LCSS, assessing six major symptoms associated with physical and functional quality of life dimensions) [92]. However, the HRQoL issues found were similar to those in studies using generic questionnaires, with physical issues like fatigue, dyspnea, and pain being the most common [92].

For tumor-specific questionnaires, two other studies used the modified LCSS questionnaire for patients with mesothelioma (LCSS-Meso), which assesses issues like appetite loss, dyspnea, hemoptysis, cough, and symptomatic distress, and three articles investigated HRQoL employing the EORTC lung-specific questionnaire (EORTC QLQ-LC13), which evaluates issues like coughing, sore mouth, peripheral neuropathy, and dyspnea. Finally, one article described HRQoL with the St. George’s Respiratory Questionnaire (SGRQ, with items on symptom frequency, symptom severity, and chronic airflow) and reported HRQoL issues with pain and dyspnea [93]. The main primary HRQoL issues were physical symptoms (e.g., pain and fatigue), dyspnea, and role functioning (e.g., dressing, washing, and walking) (Supplementary Table S2.8). One HRQoL issue, hemoptysis (discharge of blood through the mouth), was identified as missing from all questionnaires [94].

In summary, no qualitative study investigated HRQoL, and only one study focused on a thymoma population using a generic questionnaire. A total of 17 articles described HRQoL in mesothelioma patients, with 16 using a generic HRQoL tool. Of these, six also used a tumor-specific questionnaire.

### 3.9. HRQoL of Patients with a Rare Cancer of the Skin and Eye Melanoma (EURACAN G9)

Twenty-five studies investigated HRQoL in patients with rare skin and eye melanoma tumors (Supplementary Table S2.9), and only one used a qualitative approach. In the qualitative study, patients with uveal melanoma were interviewed, and HRQoL issues were identified in different areas. These include social and role functioning, such as the impact on work and home life (e.g., more frequent breaks at work or difficulty with daily household chores), as well as physical symptoms like vision impairment and eye irritation (e.g., pressure, pain, drooping of the eyelid) [95]. Finally, issues related to the emotional impact of diagnosis and stress were also discussed (e.g., stress from waiting to see a specialist or for surgery, how the diagnosis was shared with the patient) [95].

Four studies quantitatively investigated HRQoL in patients with Merkel cell carcinoma (MCC). All four studies used the same tools to assess HRQoL: the generic EQ-5D-5L questionnaire and the tumor-specific Functional Assessment of Cancer Therapy—Melanoma (FACT-M), which assesses changes in the skin, pain, swelling, numbness, and feelings of isolation or being overwhelmed by the condition (Supplementary Table S2.9). The studies found HRQoL issues mainly related to the functional and physical well-being scales [96].

The remaining 20 studies described HRQoL quantitatively in patients with choroidal or uveal melanoma. Nineteen studies used a generic questionnaire to assess HRQoL and supplemented it with a tumor-specific tool. From the generic questionnaires, the EORTC QLQ-C30, FACT-G, and SF-12 were selected in 16 articles to assess broader domains like physical, emotional, and social well-being. The others made use of the Common Terminology Criteria for Adverse Events (CTCAE) or the NCCN distress thermometer to evaluate physical and emotional concerns (e.g., physical symptoms like pain and anxiety) and practical concerns (e.g., taking care of others or changes in appearance). For tumor-specific tools, the studies used either the FACT-M (assessing changes in skin and feelings of isolation), the EORTC ophthalmic questionnaire (EORTC QLQ-OPT30, assessing ocular irritation, vision impairment, and problems with driving or reading), or the 25-item National Eye Institute Visual Function Questionnaire (VFQ-25, assessing global vision rating, limitations in social functioning, dependence on others, and peripheral and color vision issues) (Supplementary Table S2.9).

In patients with choroidal melanoma, most HRQoL issues were related to the physical domain, such as ocular discomfort and visual difficulties, as well as functioning and social well-being concerns, including dissatisfaction with appearance, fear of losing the eye, and anxiety [97]. Among studies with a uveal melanoma population, 16 used a generic questionnaire, and 9 used an additional tumor-specific tool. In the studies which solely used generic tools, HRQoL issues were found with broader domains like physical well-being (e.g., vision, fatigue), role functioning (e.g., ability to drive a car), and emotional well-being (e.g., feelings of anxiety) [98,99,100]. In studies that included a tumor-specific questionnaire, HRQoL issues related to physical limitations such as reduced vision and visual acuity, psychosocial issues like body image and appearance concerns, and problems with driving and reading were identified [101,102].

To summarize, only one study investigated HRQoL qualitatively. Twenty-four studies used a quantitative approach. Of these, 23 used a generic HRQoL tool, and 16 opted for using a tumor-specific questionnaire alongside it. Only one study solely used a tumor-specific questionnaire.

### 3.10. HRQoL of Patients with a Rare Cancer of the Brain (EURACAN G10)

Eighty-one articles studied HRQoL in patients with a rare brain tumor (Supplementary Table S2.10). Two studies assessed HRQoL employing a qualitative mixed-method approach with a focused interview combined with questionnaires. Both studies identified HRQoL issues in the physical (e.g., pain), cognitive (e.g., memory problems), and psychological domains (e.g., anxiety) [103,104]. Patients reported that the main HRQoL concerns were related to language and motor and memory functionality [103]. Additionally, one study also described psychosocial HRQoL issues, such as feelings of depression and anxiety before an evaluation consultation, uncertainty, lack of independence, and impact on work capability (e.g., lack of energy, reduced ability to think clearly) [104].

In the quantitative studies, 21 used only generic tools, such as the EORTC QLQ-C30 (*n* = 3), the SF-36 (*n* = 6), the EQ-5D (*n* = 8), WHOQOL-BREF (*n* = 2), the Functional Living Index Cancer (FLIC; *n* = 1), and the EORTC QLQ Core questionnaire for Palliative Care (EORTC QLQ-C15-PAL; *n* = 1), assessing broader domains (e.g., physical, social, and emotional well-being) based on the biopsychosocial model. Only two studies exclusively used a tumor-specific questionnaire. One study used the Functional Assessment of Cancer Therapy—Brain (FACT-Br), which assesses memory issues, concentration, seizures, coordination, and headaches. The main HRQoL issues identified were pain (e.g., headaches), neurocognitive function, and functional well-being. The other used the Sherbrooke Neuro-Oncology Assessment Scale (SNAS; assessing HRQoL issues like symptom severity and fear of death, neurocognitive function, autonomy in personal care, and acceptance of disease), and found HRQoL issues related to constipation, nausea, and fatigue (Supplementary Table S2.10).

The remaining 58 studies used a combination of generic and tumor-specific questionnaires. Of these, 46 studies used the EORTC QLQ-C30 and the EORTC QLQ-BN20. The HRQoL issues were primarily identified in the physical and functional symptom domains (e.g., pain, headache, fatigue, bladder control, hair loss, constipation, nausea, drowsiness, and weakness), cognitive impairments (e.g., communication issues, motor dysfunction, visual disorders, working memory deficits, attention problems), and the psychosocial domain (e.g., uncertainty about the future, disease acceptance, and financial difficulties).

Other combinations of questionnaires included the SF-36 and the EORTC QLQ-BN20 (*n* = 4), the FACT-G and the FACT-Br (*n* = 5), the Ferrans and Powers Quality of Life Index Cancer (FPQLI-C) and the FACT-Br (*n* = 1; main HRQoL issues were with fear, worry, coping, independence, and pain), the EORTC QLQ-C15-PAL and the EORTC QLQ-BN20 (*n* = 1), or the PROMIS and the Quality of Life in Neurological Disorders (Neuro-QOL, assessing emotional and behavioral dyscontrol issues like impulsivity) (*n* = 1). HRQoL issues included aphasia severity, anxiety, cognitive distress, and pain (mainly headache) [103].

In summary, two studies assessed HRQoL using interviews. Additionally, all studies used either a generic or a tumor-specific questionnaire. Of these, 21 studies used only a generic questionnaire, 2 studies used only a tumor-specific questionnaire, and 58 used both.

## 4. Discussion

This study is the first systematic literature review investigating HRQoL in patients with rare adult solid cancers. It identified how HRQoL is currently assessed among patients with rare adult solid cancer, both in general and within each EURACAN domain. The assessments ranged from generic questionnaires (e.g., SF-36, EORTC QLQ-C30) to tumor-specific tools (e.g., FACT-Br for brain cancer or EORTC QLQ-LC13 for mesothelioma). The frequent use of generic questionnaires suggests a preference for well-established instruments, though there is a lack of tumor-specific questionnaires in some domains. However, in more than half of the studies, a domain- or tumor-specific approach was employed, reflecting the development of tailored measures to address key tumor-specific HRQoL concerns. Generic questionnaires were predominantly used in the sarcoma (G1), male genitourinary (G3), endocrine cancer (G6), and thorax (G8) domains. In contrast, the female genital organ domain (G2) more frequently utilized tumor- and domain-specific questionnaires. Additionally, tumor- or domain-specific questionnaires, alongside generic ones, were employed in over half of the studies in the brain (G10), head and neck (G7), digestive tract (G5), and uveal and skin melanoma (G9) domains. Finally, this review highlights potential future strategies and areas of focus for improving the measurement of HRQoL in patients with rare adult solid cancers, emphasizing the need for more tailored tools in certain EURACAN domains (Table 2).

In patients with sarcoma, the use of generic instruments, such as the EORTC QLQ-C30, persists, even though these tools may not adequately capture the diverse impact of sarcomas on patients’ HRQoL. It is essential to consider the unique issues associated with different sarcoma locations, such as respiratory problems in thoracic sarcomas, sexual difficulties in uterine sarcomas, or difficulty in walking for limb sarcomas [105]. Depending on the symptoms, tools for other diseases, such as colorectal cancer questionnaires for gastrointestinal complaints, may be appropriate [105]. Similarly, both reproductive organ tumor domains (G2 and G3) show a lack of specific questionnaires to explore HRQoL. For example, in patients with penile cancer who undergo radical penectomy, concerns about sexuality, erectile function, and incontinence may differ or become irrelevant compared to other tumors. Instead, issues like body image and masculinity take on greater importance [29,37,45]. Inconsistencies in the choice of instruments across studies hinder the comparability of results and the understanding of HRQoL issues unique to male genital cancers. Likewise, in patients with vulvar cancer, relying solely on generic instruments may overlook specific HRQoL issues, such as psychosocial functioning and sexual health (e.g., masturbation) [31].

On the other hand, patients from the neuroendocrine domain (G4) benefit from a combination of validated generic and tumor-specific tools (e.g., EORTC QLQ-GI.NET21 and the Norfolk quality of life tool), which is considered the reference method for addressing HRQoL issues in patients with gastroenteropancreatic NET. Correspondingly, patients with a rare thoracic diagnosis (G8), such as mesothelioma, benefit from a well-established combination of generic and tumor-specific instruments, including the LCSS-Meso or the EORTC QLQ-LC13, which are validated and tailored for this population to capture their specific HRQoL needs [106,107].

However, despite the availability of tumor-specific questionnaires that primarily capture physical complaints, NET and rare thoracic tumor populations still require more nuanced assessments that account for additional factors. For instance, in patients with NETs, the existing tools may not fully address all NET types, leading to variations in HRQoL depending on tumor location, grade, disease trajectory, or treatment-related factors. These variables should be incorporated into a stratification matrix when developing more comprehensive questionnaires [108]. Furthermore, current tools do not account for daily fluctuations in symptoms, dietary variations, or malnutrition, which can significantly impact patients’ HRQoL [52,109,110,111]. Tumor-specific questionnaires for certain NET types, such as pancreatic NETs, or specific rare thoracic cancers, like thymoma, are still lacking, highlighting a significant gap in the literature.

HRQoL assessment for patients with endocrine tumors has made significant progress, particularly for those with thyroid cancer. Even though various tumor-specific questionnaires are available to address important HRQoL issues in this group, the high number of available questionnaires might not help this field of research as it is not clear when to use which questionnaire. In addition, this focus on thyroid cancer has resulted in a lack of research on other subtypes, such as adrenocortical carcinoma (ACC). Fortunately, a recently developed ACC-specific questionnaire allows for more targeted assessments of symptoms and experienced treatment side effects [74,75].

The head and neck (G7) and brain (G10) domains have demonstrated that HRQoL issues can be fully captured when a precise strategy exists. More than half of the studies used the same combination of generic and tumor-specific questionnaires from the EORTC (EORTC QLQ-C30 with EORTC QLQ-BN20) for patients with rare adult brain tumors. This combination highlights the importance of using both measures to comprehensively address the multifaceted HRQoL concerns of these patients, from physical symptoms to cognitive impairments and psychosocial challenges.

In certain domains, such as rare cancers of the digestive tract (G5) and skin and eye melanoma (G9), our review shows that only specific diagnoses have a clear strategy using both generic and tumor-specific questionnaires. Other subtypes still lack specialized tools to assess their specific concerns. For example, in patients with cholangiocarcinoma, the EORTC QLQ-BIL21 has been validated specifically for this population, capturing HRQoL issues related to treatment side effects, such as weight loss, in addition to disease-specific issues like eating difficulties and jaundice symptoms [73]. Conversely, although a tumor-specific tool exists for anal cancer patients (EORTC QLQ-ANL27), its use remains limited as it was only recently validated.

For patients with skin or uveal melanoma, even though the FACT-M has been the primary choice for assessing tumor-specific HRQoL issues in patients with Merkel cell carcinoma (mMCC) due to the absence of an mMCC tumor-specific tool, it was developed for melanoma patients, and may not fully capture mMCC-specific issues [96]. While different combinations of generic and domain-specific questionnaires effectively capture HRQoL issues in uveal melanoma, no tumor-specific tool has yet been developed for this population. The optimal combination of tools should be considered on a case-by-case basis to ensure the best results, as some tools may focus more on melanoma or treatment-associated symptoms.

All in all, the inconsistency in the selection of HRQoL instruments across studies limits the comparability of findings and hampers the development of a comprehensive understanding of HRQoL issues for patients with rare adult solid cancers. A one-size-fits-all approach using generic tools is insufficient to capture the full scope of HRQoL, and there is a pressing need for domain-specific instruments to accurately assess patient well-being. Standardizing the use of specific, validated tools where possible, or developing new instruments where they are lacking, will be crucial for ensuring robust, comparable data across rare cancer types.

The review highlights the diverse and heterogeneous nature of HRQoL issues in patients with rare adult solid cancers, encompassing physical, emotional, social, and psychological issues. Healthcare professionals often underestimate the impact of these diseases on HRQoL, emphasizing the need for more personalized HRQoL assessments. While some of the EURACAN domains have made progress in developing generic, domain-specific, or tumor-specific questionnaires, such development is not always feasible due to low patient numbers. In these cases, individualized approaches can be created by utilizing existing items from item libraries or input from other questionnaires [112].

For domains where a clear strategy is missing but sufficient evidence exists, efforts should be made to develop tumor-specific questionnaires, create tailored combinations of existing questionnaires, or make use of the EORTC Item Library to complement instruments [112]. An ongoing EORTC QLG study by Padilla and co-authors (2023) aims to identify relevant questionnaires for different adult rare solid cancer diagnoses and assess the validity and reliability of existing tools, with the ultimate goal of developing customized HRQOL strategies [6]. This EORTC project is a starting point from a larger framework that seeks to provide a strategy for domains that currently rely solely on generic instruments, which may not capture the full spectrum of patient issues, or that do not have applicable instruments. By recommending and developing more tailored HRQoL strategies, either by creating shorter questionnaire packs or by adding items from the EORTC item library to the used questionnaires, the project aims to help reduce patient burden and overload with (irrelevant) questionnaires [6]. Using an item library allows researchers and clinicians to create tailored, context-specific HRQoL measures that better capture the nuances of patient experiences across different EURACAN domains. This approach enhances the accuracy of assessments while minimizing patient burden.

An additional challenge arises when developing a strategy for ultra-rare cancers. This group of patients with adult rare cancer subtypes is defined as those with an incidence of less than 1 in 1,000,000 people per year [113]. Patients with adult ultra-rare solid cancer subtypes face even greater challenges due to a lack of research and evidence on their HRQoL compared to those with adult rare solid cancer. As a result, HRQoL assessment strategies may vary in their adequacy. One example is epithelioid hemangioendothelioma (EHE), an adult ultra-rare vascular sarcoma. Patients with EHE suffer from a lack of information and an unpredictable disease course, and currently have their HRQoL assessed only through generic questionnaires, such as the EORTC QLQ-C30 [113]. Unlike patients with more common soft tissue sarcomas, patients with EHE cannot rely on domain-specific questionnaires. This makes developing a tailored HRQoL strategy more challenging, requiring a different approach to address their unique HRQoL needs.

One critical point that emerged is the growing recognition of the importance of involving patients in the development of HRQoL tools, and a potential solution to more accurately identify issues and increase participants in studies is increased patient involvement. Existing instruments, while valuable, often fail to give patients a voice in expressing the full spectrum of their lived experiences. Patient organizations’ initiatives and a more patient-centered approach can provide valuable insight into their struggles and help create an item list specifically for that population. Future research should emphasize patient-reported outcomes that not only measure clinical aspects but also prioritize the subjective experiences that matter most to the patients themselves. However, it is essential to remember that while an item list can be developed, validating a questionnaire may be challenging due to the small number of participants, and conducting a phase 4 study may not be feasible.

### Strengths and Limitation

The main strength of this review is that it addresses all domains of HRQoL across a wide range of rare adult solid cancers, considering all domain subtypes and providing a comprehensive understanding of the current state of HRQoL assessment in this population. Another strength is its broad scope, which includes both qualitative and quantitative studies. Furthermore, by including articles from 2011 onwards, we avoid incorrect interpretations of rare adult solid cancer definitions, as was the case in 2011 when the first epidemiological analysis on the incidence of rare cancers in Europe was performed [1]. This provides a complete overview of all available studies on HRQoL in patients with rare adult solid cancer.

The main limitation of this review is that we could not describe every study in detail due to the large number of publications included, which required us to omit some specific findings.

## 5. Conclusions

This review found that while generic questionnaires are commonly used, they often lack the specificity needed to capture the unique HRQoL issues of patients with rare adult solid cancers. Therefore, combining generic and tumor- or domain-specific questionnaires across EURACAN domains may provide a more robust approach to understanding HRQoL in patients with rare adult solid cancers, addressing both generic and specific patient needs. Some specific questionnaires, such as those for neuroendocrine tumors, have been validated to address distinct HRQoL needs, whereas others, like those for sarcomas, lack suitable assessment tools. Our findings highlight the need for a more tailored, individualized approach to HRQoL assessment, particularly for patients with adult ultra-rare solid cancers, where research is limited, and existing tools are often inadequate. Further research could benefit from exploring the use of multiple HRQoL instruments, utilizing item lists, and developing or refining questionnaires to better address the diverse HRQoL concerns of patients with rare adult solid cancers, ultimately providing a more focused understanding of these patients’ experiences.

## Figures and Tables

**Table 1:** EURACAN domain division of adult solid rare cancers.

Domain	Domain Name	Rare Cancer Diagnosis
G1	Sarcoma	Rare cancer of the connective tissues (sarcoma)
G2	Gynecology	Rare cancer of the female genital organs and placenta
G3	Male genital organs	Rare cancer of the male genital organs and of the urinary tract
G4	NET *	Rare cancer of the neuroendocrine system
G5	Digestive tract	Rare cancer of the digestive tract
G6	Endocrine	Rare endocrine cancer
G7	Head and neck	Rare head and neck cancer
G8	Thorax	Rare thoracic cancer
G9	Skin cancers and uveal melanoma	Rare skin cancer and uveal melanoma
G10	Brain and spinal cord	Rare cancer of the brain and of the spinal cord

* NET: Neuroendocrine.

**Table 2:** Summary of current HRQoL strategies per EURACAN domain.

CURRENT HRQoL MEASUREMENT STRATEGY	
GENERIC QUESTIONNAIRE	G1 SARCOMA
	G3 MALE GENITOURINARY ORGANS
	G8 THORAX
	G6 ENDOCRINE
TUMOUR- OR DOMAIN-SPECIFIC QUESTIONNAIRE	G2 FEMALE GENITAL ORGANS
COMBINATION OF BOTH	G10 BRAIN
	G7 HEAD AND NECK
	G5 DIGESTIVE TRACT
	G9 SKIN AND UVEAL MELANOMA
	G4 NEUROENDOCRINE

## Supplementary Materials

The following supporting information can be downloaded at https://www.mdpi.com/article/10.3390/cancers17030387/s1: File S1: Complete Search String, File S2: Table S2.1: EURACAN G1: Rare cancer of the connective tissues (sarcomas), Table S2.2: EURACAN G2: Rare GYN—rare cancer of the female genital organs and placenta, Table S2.3: EURACAN G3: GU—rare cancer of the male genital organs and of the urinary tract, Table S2.4: EURACAN G4: NET—rare cancer of the neuroendocrine system, Table S2.5: EURACAN G5: Digestive tract—rare cancer of the digestive tract, Table S2.6: EURACAN G6: Endocrine—rare cancer of the endocrine organs, Table S2.7: EURACAN G7: Head and neck—rare cancer of the head and neck, Table S2.8: EURACAN G8: Thoracic—rare cancer of the thorax, Table S2.9: EURACAN G9: Skin and eye melanoma—rare cancer of the skin and eye melanoma, Table S2.10: EURACAN G10 Brain—rare cancer of the brain and spinal cord.
