# Supplementary material for: Health-Related Quality of Life (HRQoL) Assessments in Research on Patients with Adult Rare Solid Cancers: A State-of-the-Art Review
Source: Cancers (Basel). 2025 Jan 24;17(3):387. doi: 10.3390/cancers17030387 (PMC11816368; doi:10.3390/cancers17030387)
Supplement: Supplementary file 1 [file cancers-17-00387-s001.zip › Supplementary File 2_ EURACAN domain tables.pdf]

Supplementary table 2.1. EURACAN G1: Rare cancer of the connective tissues (sarcomas)

Author, year [ref]	Article title	Continent	Subtypes of cancer	Study design*	HRQoL end point	Number of HRQoL assessments	Type of HRQoL assessment	Generic questionnaire [a]	Tumour/domain-specific questionnaire [b]
Bonvalot et al., 2022 [1]	Final Safety and Health-Related Quality of Life Results of the Phase 2/3 Act.In.Sarc Study With Preoperative NBTXR3 Plus Radiation Therapy Versus Radiation Therapy in Locally Advanced Soft-Tissue Sarcoma	Europe; Asia; Oceania; North America	Soft tissue sarcoma	RCT	Secondary	18	Generic questionnaire	EQ-5D-5L	TESS, MSTS
Üstündag et al., 2015 [2]	Factors affecting the quality of life of cancer patients undergoing chemotherapy: A questionnaire study	Asia	Sarcoma: not specified	Cross-sectional	Primary	1	Generic questionnaire	N-SAS	
Davidson et al., 2016 [3]	Health-related quality of life following treatment for extremity soft-tissue sarcoma	North America	Soft tissue sarcoma	Longitudinal observational cohort	Primary	2	Generic and tumour-specific questionnaire	EQ-5D	TESS, MSTS
Day et al., 2022 [4]	Patient reported quality of life in young adults with sarcoma receiving care at a sarcoma center	North America	Soft tissue sarcoma	Cohort	Primary	2	Generic questionnaire	FACT-G	
den Hollander et al., 2022 [5]	Symptoms reported by gastrointestinal stromal tumour (GIST) patients on imatinib treatment: combining questionnaire and forum data	Europe	GIST	Cross-sectional	Primary	1	Generic questionnaire	EORTC QLQ-C30, EORTC-SBQ	
den Hollander et al., 2022 [6]	I thought I had fibroids, and now I don't': a mixed method study on health-related quality of life in uterine sarcoma patients	Europe	Soft tissue sarcoma	Mixed-method	Primary	1	Interview + Generic and tumour-specific questionnaire	EORTC QLQ-C30	EORTC QLQ-EN24
Dong et al., 2018 [7]	Quality of life and Q-twist were not adversely affected in Ewing sarcoma patients treated with combined anlotinib, irinotecan, and vincristine: (Peking University People's Hospital Ewing sarcoma trial-02, PKUPH-EWS-02)	Asia	Soft tissue sarcoma	Prospective	Primary	7	Generic questionnaire	EORTC QLQ-C30	
Drabbe et al., 2021 [8]	The age-related impact of surviving sarcoma on health-related quality of life: data from the SURVSARC study	Europe	Bone sarcoma; soft tissue sarcoma	Cross-sectional	Primary	1	Generic questionnaire	EORTC QLQ-C30	
Dressler et al., 2016 [9]	Long-term functional outcomes of laparoscopic resection for gastric gastrointestinal stromal tumors	North America	GIST	Cohort	Primary	10	Generic and tumour-specific questionnaire	QoL Survey developed by TJUH dept. of surgery	GIQLI
Fauske et al., 2020 [10]	Striving towards Normality in Daily Life: A Qualitative Study of Patients Living with Metastatic Gastrointestinal Stromal Tumour in Long-Term Clinical Remission	Europe	GIST	Qualitative exploratory	Primary	1	Interview		
Ferguson et al., 2022 [11]	Self-reported cognitive impairments and quality of life in patients with gastrointestinal stromal tumor: Results of a multinational survey	North America	GIST	Cross-sectional	Primary	1	Generic questionnaire	SF-36	FACT-Cog
Fiore et al., 2021 [12]	A Prospective Observational Study of Multivisceral Resection for Retroperitoneal	Europe	Soft tissue sarcoma	Observational	Primary	3	Generic and tumour-specific questionnaire	EORTC QLQ-C30	EORTC QLQ-CR29, LEFS

	Sarcoma: Clinical and Patient-Reported Outcomes 1 Year After Surgery								
Furtado et al., 2015 [13]	Physical functioning, pain and quality of life after amputation for musculoskeletal tumours	Europe	Bone sarcoma; soft tissue sarcoma	Cross-sectional	Primary	1	Generic questionnaire	QoL-CS	TESS
Yoo et al., 2016 [14]	Impact of imatinib rechallenge on health-related quality of life in patients with TKI-refractory gastrointestinal stromal tumours: Sub-analysis of the placebo-controlled, randomised phase III trial (RIGHT)	Asia; North American	GIST	RCT	Primary	5	Generic questionnaire	EORTC QLQ-C30	
Younger et al., 2020 [15]	Health-Related Quality of Life and Experiences of Sarcoma Patients during the COVID-19 Pandemic	Europe	Bone sarcoma; soft tissue sarcoma; GIST	Cross-sectional	Primary	1	Generic questionnaire	EORTC QLQ-C30	
Lazenby et al., 2012 [16]	Associations among Patient Characteristics, Health-Related Quality of Life, and Spiritual Well-Being among Arab Muslim Cancer Patients	Middle East	Bone sarcoma	Cross-sectional	Primary	1	Generic questionnaire	FACT-G, FACIT-Sp	
Le Cesne et al., 2021 [17]	A randomized phase III trial comparing trabectedin to best supportive care in patients with pre-treated soft tissue sarcoma: T-SAR, a French Sarcoma Group trial	Europe	Soft tissue sarcoma	RCT	Primary	At least 13	Generic questionnaire	EORTC QLQ-C30	
Lim et al., 2020 [18]	Retrospective quality of life study in patients with retroperitoneal sarcoma in an Asian population	Asia	Soft tissue sarcoma	Cross-sectional	Primary	2	Generic questionnaire	EORTC QLQ-C30	
Lopez-Guerra et al., 2011 [19]	Health related quality of life and late side effects of long-term survivors of Ewing's sarcoma of bone	Europe; North America	Soft tissue sarcoma	Cross-sectional	Primary	1	Generic questionnaire	EORTC QLQ-C30	
Weidema et al., 2020 [20]	Health-related quality of life and symptom burden of epithelioid hemangioendothelioma patients: a global patient-driven Facebook study in a very rare malignancy	Europe; North America	Soft tissue sarcoma	Cross-sectional	Primary	1	Generic questionnaire	EORTC QLQ-C30	
Weschenfelder et al., 2020 [21]	Factors influencing quality of life, function, reintegration and participation after musculoskeletal tumour operations	Europe	Soft tissue sarcoma	Cross-sectional	Primary	1	Generic and tumour-specific questionnaire	EORTC QLQ-C30, SF-36	SMFA
Wong et al., 2017 [22]	Long-Term Quality of Life of Retroperitoneal Sarcoma Patients Treated with Pre-Operative Radiotherapy and Surgery	North America	Soft tissue sarcoma	Cohort	Primary	18	Generic questionnaire	EORTC QLQ-C30	
Eliason et al., 2022 [23]	Qualitative study to characterize patient experience and relevance of patient-reported outcome measures for patients with metastatic synovial sarcoma	North America	Soft tissue sarcoma	Qualitative	Primary	1	Interview + generic questionnaire	EORTC IL31, EORTC QLQ-C30	
Eichler et al., 2022 [24]	The association of Health-Related Quality of Life and 1-year survival in sarcoma patients—results of a Nationwide Observational Study	Europe	Soft tissue sarcoma; GIST; bone sarcoma	Observational	Primary	2	Generic questionnaire	EORTC QLQ-C30	
Eichler et al., 20220 [25]	The Health-Related Quality of Life of Sarcoma Patients and Survivors in Germany—Cross-Sectional Results of a Nationwide Observational Study	Europe	Soft tissue sarcoma; GIST; bone sarcoma	Cross-sectional	Primary	1	Generic questionnaire	EORTC QLQ-C30	
Eichler et al., 2021 [26]	Quality of life of GIST patients with and without current tyrosine kinase inhibitor treatment: Cross-	Europe	GIST	Cross-sectional	Primary	1	Generic questionnaire	EORTC QLQ-C30	

	sectional results of a German multicentre observational study								
Edmondson et al., 2021 [27]	Phase 2 study of anastrozole in rare cohorts of patients with estrogen receptor/progesterone receptor positive leiomyosarcomas and carcinosarcomas of the uterine corpus: The PARAGON trial (ANZGOG 0903)	Europe; Oceania	Soft tissue sarcoma	RCT	Secondary	1	Generic and tumour-specific questionnaire	EORTC QLQ-C30	FACT-ES
Hentschel et al., 2020 [28]	Quality of life and added value of a tailored palliative care intervention in patients with soft tissue sarcoma undergoing treatment with trabectedin: a multicentre, cluster-randomised trial within the German Interdisciplinary Sarcoma Group (GISG)	Europe	Soft tissue sarcoma	RCT	Primary	7	Generic questionnaire	FACT-G, M-DASI	
Holzer et al., 2020 [29]	Body image, self-esteem, and quality of life in patients with primary malignant bone tumors	Europe	Bone sarcoma	Cohort	Primary	1	Generic and tumour-specific questionnaire	SF-36	MSTS
Hudgens et al., 2017 [30]	Evaluation of Quality of Life at Progression in Patients with Soft Tissue Sarcoma	Europe; North America	Soft tissue sarcoma	RCT	Primary	2	Generic questionnaire	EORTC QLQ-C30	
Sachsenmaier et al., 2015 [31]	Quality of life, physical and mental status and contentment of patients with localized soft tissue or bone sarcoma: a questionnaire analysis	Europe	Soft tissue sarcoma; bone sarcoma	Survey	Primary	1	Generic questionnaire	Self-created questionnaire	
Saebye et al., 2017 [32]	Factors associated with reduced functional outcome and quality of life in patients having limb-sparing surgery for soft tissue sarcomas – a national multicenter study of 128 patients	Europe	Soft tissue sarcoma	Cross-sectional	Primary	1	Generic and tumour-specific questionnaire	EORTC QLQ-C30	MSTS, TESS
Saebye et al., 2020 [33]	Changes in Functional Outcome and Quality of Life in Soft Tissue Sarcoma Patients within the First Year after Surgery: A Prospective Observational Study	Europe	Soft tissue sarcoma	Observational	Primary	4	Generic and tumour-specific questionnaire	WHO-5	MSTS, TESS
Schöffski et al., 2022 [34]	Patient-reported outcomes in individuals with advanced gastrointestinal stromal tumor treated with ripretinib in the fourth-line setting: analysis from the phase 3 INVICTUS trial	Europe	GIST	RCT	Primary	10	Generic questionnaire	EORTC QLQ-C30, EQ-5D-5L	
Silva et al., 2020 [35]	Quality of life in adults with sarcomas under conservative surgery or amputation	South America	Bone sarcoma	Cross-sectional	Primary	1	Generic and tumour-specific questionnaire	EORTC QLQ-C30	MSTS
Soomers et al., 2020 [36]	The Perceived Impact of Length of the Diagnostic Pathway Is Associated with Health-Related Quality of Life of Sarcoma Survivors: Results from the Dutch Nationwide SURVSARC Study	Europe	Soft tissues sarcoma; bone sarcoma	Cross-sectional	Primary	1	Generic questionnaire	EORTC QLQ-C30	
Srivastava et al., 2013 [37]	Quality of life in patients with chordomas/chondrosarcomas during treatment with proton beam therapy	Europe; Asia	Bone sarcoma	Longitudinal	Primary	2	Generic questionnaire	EORTC QLQ-C30	

Maggi et al., 2019 [38]	Sarcoma patients' quality of life from diagnosis to yearly follow-up: experience from an Italian tertiary care center	Europe	Soft tissues sarcoma; bone sarcoma	Longitudinal	Primary	8	Generic questionnaire	EORTC QLQ-C30	
Maggi et al., 2021 [39]	Symptoms and their implications on quality of life and psychological distress in sarcoma-patients	Europe	Soft tissue sarcoma; bone sarcoma	Observational	Primary	10	Generic questionnaire	EORTC QLQ-C30	
Malek et al., 2012 [40]	Does Limb-salvage Surgery Offer Patients Better Quality of Life and Functional Capacity than Amputation?	North America	Soft tissue sarcoma; bone sarcoma	Cohort	Primary	1	Generic and tumour-specific questionnaire	SF-36	TESS
Mason et al., 2013 [41]	Quality of life following amputation or limb preservation in patients with lower extremity bone sarcoma	North America	Bone sarcoma	Cross-sectional	Primary	1	Generic questionnaire	QLQ	
Reichardt et al., 2012 [42]	Quality of Life and Utility in Patients with metastatic Soft Tissue and Bone Sarcoma: The Sarcoma Treatment and Burden of Illness in North America and Europe (SABINE) Study	Europe; North America	Soft tissue sarcoma; bone sarcoma	Cross-sectional	Primary	1	Generic questionnaire	EORTC QLQ-C30, EQ-5D, 3-item cancer related symptoms questionnaire	
Reijers et al., 2021 [43]	Health-related quality of life after isolated limb perfusion compared to extended resection, or amputation for locally advanced extremity sarcoma: Is a limb salvage strategy worth the effort?	Europe	Soft tissue sarcoma	Cross-sectional	Primary	1	Generic and tumour-specific questionnaire	EORTC QLQ-C30	TESS
Rivard et al., 2015 [44]	Quality of Life, Functional Outcomes, and Wound Complications in Patients with Soft Tissue Sarcomas Treated with Preoperative Chemoradiation: A Prospective Study	North America	Soft tissue sarcoma	Cohort	Primary	3	Generic and tumour-specific questionnaire	SF-36	MSTS, TESS
Vade et al., 2021 [45]	Exploring Mentorship as a Novel Approach to Improving Quality of Life in Sarcoma Survivors: A Qualitative Pilot Study	Europe	GIST; bone sarcoma; soft tissue sarcoma	Qualitative	Primary	1	Interview		
Van Eck et al., 2020 [46]	Unraveling the Heterogeneity of Sarcoma Survivors' Health-Related Quality of Life Regarding Primary Sarcoma Location: Results from the SURVSARC Study	Europe	Soft tissue sarcoma; bone sarcoma	Cross-sectional	Primary	1	Generic questionnaire	EORTC QLQ-C30	
van Eck et al., 2021 [47]	Health-Related Quality of Life Issues Experienced by Thoracic and Breast Sarcoma Patients: A Rare and Understudied Group	Europe	Soft tissue sarcoma	Qualitative	Primary	1	Interview		
van Tine et al., 2022 [48]	Quality of life of patients with soft tissue sarcoma treated with doxorubicin in the ANNOUNCE phase III clinical trial	North America; Europe; Asia	Soft tissue sarcoma	RCT	Primary	16	Generic questionnaire	EORTC QLQ-C30	
Jing et al., 2022 [49]	Efficacy of Neoadjuvant Chemotherapy plus Limb-Sparing Surgery for Osteosarcoma and Its Impact on Long-Term Quality of Life	Asia	Bone sarcoma	RCT	Primary	1	Generic questionnaire	EORTC QLQ-C30, SF-36	
Obermair et al., 2011 [50]	Prospective, non-randomized phase 2 clinical trial of carboplatin plus paclitaxel with sequential	Oceania	Soft tissue sarcoma	RCT	Primary	6	Generic and tumour-specific questionnaire	FACT-G	FACT-En

	radical pelvic radiotherapy for uterine papillary serous carcinoma								
Ogura et al., 2021 [51]	The Critical Difference in the DASH Outcome Measure After Essential Upper Extremity Tumor Surgery	North America	Soft tissue sarcoma	Observational	Primary	At least 2	Tumour-specific questionnaire		DASH
Ostacoli et al., 2013 [52]	Quality of Life, Anxiety and Depression in Soft Tissue Sarcomas as Compared to More Common Tumours: An Observational Study	Europe	Soft tissue sarcoma	Observational	Primary	1	Generic questionnaire	FACT-G	
Zhuang et al., 2022 [53]	Does Aggressive Surgery Mean Worse Quality of Life and Functional Capacity in Retroperitoneal Sarcoma Patients?—A Retrospective Study of 161 Patients from China	Asia	Soft tissue sarcoma	Cohort	Primary	7	Generic questionnaire	EORTC QLQ-C30	
Götzl et al., 2019 [54]	Patient's quality of life after surgery and radiotherapy for extremity soft tissue sarcoma - a retrospective single-center study over ten years	Europe	Soft tissue sarcoma	Cohort	Primary	1	Generic questionnaire	EORTC QLQ-C30	
Gough et al., 2019 [55]	Does palliative chemotherapy really palliate and are we measuring it correctly? A mixed methods longitudinal study of health related quality of life in advanced soft tissue sarcoma	Europe	Soft tissue sarcoma	Mixed-method longitudinal	Primary	2	Interview + generic questionnaire	EORTC QLQ-C30	
Gounder et al., 2021 [56]	Health-related quality of life and pain with selinexor in patients with advanced dedifferentiated liposarcoma	North America; Europe	Soft tissue sarcoma	Longitudinal	Primary	15	Generic questionnaire	EORTC QLQ-C30	
Paredes et al., 2011 [57]	Quality of life of sarcoma patients from diagnosis to treatments: Predictors and longitudinal trajectories	Europe	Soft tissue sarcoma; bone sarcoma	Longitudinal	Primary	3	Generic questionnaire	EORTC QLQ-C30	
Paredes et al., 2012 [58]	Social Support and Adjustment in Patients with Sarcoma: The Moderator Effect of the Disease Phase	Europe	Soft tissue sarcoma; bone sarcoma	Cross-sectional	Primary	3	Generic questionnaire	WHOQOL-BREF	
Poole et al., 2014 [59]	Health utility of patients with advanced gastrointestinal stromal tumors (GIST) after failure of imatinib and sunitinib: findings from GRID, a randomized, double-blind, placebo-controlled phase III study of regorafenib versus placebo	Europe	GIST	RCT	Primary	4	Generic questionnaire	EQ-5D-3L	
Carbajal-López et al., 2022 [60]	Psychological Distress, Fatigue and Quality of Life in Patients with Gastrointestinal Stromal Tumors	Central America	GIST	Cross-sectional	Primary	1	Generic questionnaire	EORTC QLQ-C30	
Chuah et al., 2021 [61]	Assessment of Adherence to Imatinib and Health-Related Quality of Life Among Patients with Gastrointestinal Stromal Tumor: A Cross-Sectional Study in an Oncology Clinic in Malaysia	Asia	GIST	Cross-sectional	Primary	1	Generic questionnaire	EORTC QLQ-C30	
Coens et al., 2015 [62]	Health-Related Quality-of-Life Results From PALETTE: A Randomized, Double-Blind, Phase 3 Trial of Pazopanib Versus Placebo in Patients With Soft Tissue Sarcoma Whose Disease Has Progressed During or After Prior Chemotherapy—A European Organization for Research and Treatment of Cancer Soft Tissue and Bone	Europe	Soft tissue sarcoma; bone sarcoma	RCT	Primary	4	Generic questionnaire	EORTC QLQ-C30	

	Sarcoma Group Global Network Study (EORTC 62072)								
Custers et al., 2015 [63]	Fear of progression in patients with gastrointestinal stromal tumors (GIST): Is extended lifetime related to the Sword of Damocles?	Europe	GIST	Cross-sectional	Primary	1	Generic questionnaire	EORTC QLQ-C30	
Kask et al., 2021 [64]	Soft Tissue Sarcoma of Lower Extremity: Functional Outcome and Quality of Life	Europe	Soft tissue sarcoma	Cross-sectional	Primary	1	Generic and tumour-specific questionnaire	EORTC QLQ-C30,15D	TESS
Kokkali et al., 2022 [65]	A Multicenter, Prospective, Observational Study to Assess the Clinical Activity and Impact on Symptom Burden and Patients'Quality of Life in Patients with Advanced Soft Tissue Sarcomas Treated with Trabectedin in a Real-World Setting in Greece	Europe	Soft tissue sarcoma	Observational	Primary	2	Generic questionnaire	EQ-5D-3L, G-MDASI	
Barry et al., 2022 [66]	The Impact of Disease Progression on Health Related Quality of Life Outcomes in Patients With Oligometastatic Disease at 12 Months Post Stereotactic Body Radiation Therapy	North America; South America; Europe	Sarcoma: not specified	Longitudinal	Secondary	4	Generic questionnaire	EORTC QLQ-C30	

[*] RCT: Randomized Controlled Trial

[a] Generic questionnaire: EQ-5D-5L: EUroQoL-5 Dimensions; N-SAS: Nightingale Symptom Assessment Scale; FACT-G: Functional Assessment of Cancer Therapy-General; EORTC QLQ-C30: European Organization for Research and Treatment for Cancer Quality of Life Questionnaire-Core30; EORTC- QLQ-SBQ: European Organization for Research and Treatment for Cancer Quality of Life Questionnaire Symptom-based questionnaire; SF-36: Medical Outcome Study 36-item Short Form Health Survey; QoL-CS: Quality of Life – Cancer survivors scales; FACIT-Sp: Functional Assessment in Chronic Illness Therapy–Spiritual Well-being; EORTC ILC31: European Organization for Research and Treatment of Cancer Item Library 31; M-DASI: modified Duke Activity Status Index; WHO-5: The World Health Organization-FiveWell-Being Index; QLQ: Quality of Life Questionnaire; WHOQOL BREF; The World Health Organization Quality of Life questionnaire; 15D: generic, comprehensive, 15-dimensional, standardized, self-administered measure of HRQoL; G-MDASI: Greek version – M.D. Anderson Symptom Inventory

[b] Tumour/domain-specific questionnaire: FACT-Cog: Functional Assessment of Cancer Therapy - Cognitive Function; TESS: Toronto extremity salvage score; MSTS: Musculoskeletal tumour rating scale; DASH: Disabilities of the Arm, Shoulder and Hand; EORTC QLQ-EN24: EORTC Quality of Life Questionnaire Endometrial Cancer Module GIQLI: Gastrointestinal Quality of Life Index; EORTC QLQ-CR29: EORTC Quality of Life Questionnaire Colorectal Cancer Module; LEFS: Lower Extremity Functional Scale; SMFA: Short Musculoskeletal Function Assessment Questionnaire; FACT-En: Functional Assessment of Cancer Therapy – Endometrial; FACT-ES: Functional Assessment of Cancer Therapy – Endocrine Symptoms

Supplementary table 2.2. EURACAN G2: Rare GYN - Rare cancer of the female genial organs and placenta

Author, year [ref]	Article title	Continent	Subtypes of cancer	Study design	HRQoL end point	Number of HRQoL assessments	Type of HRQoL assessment	Generic questionnaire [a]	Tumour/domain-specific questionnaire [b]
Ferracini et al., 2021 [67]	Physical and functional well-being and symptoms of ovarian cancer in women undergoing first-line of chemotherapy: a one-year follow-up	South America	Ovarian	Longitudinal	Primary	12	Tumour-specific questionnaire		FACT-O, FACT-O-TOI
Forner et al., 2013 [68]	Can clitoris-conserving surgery for early vulvar cancer improve the outcome in terms of quality of life and sexual sensation?	Europe	Vulvar	Cohort	Primary	1	Generic and tumour-specific questionnaire	SF-12	FSFI
Forner et al., 2015 [69]	Quality of life and sexual function after surgery in early stage vulvar cancer	Europe	Vulvar	Cohort	Primary	1	Generic and tumour-specific questionnaire	SF-12	FSFI
van Dongen et al., 2022 [70]	Challenges and controversies patients and (health care) professionals experience in managing vaginal, vulvar, penile or anal cancer: The SILENCE study	Europe	Vaginal; vulvar	Qualitative exploratory	Primary	1	Interview		
Tsubamoto et al., 2019 [71]	Effects of leuprorelin for the treatment of recurrent gynecological cancer by assessment including self-administered quality-of-life questionnaire	Asia	Uterine; ovarian	Cohort	Primary	2	Generic questionnaire	Care Notebook QOL	
Zaid et al., 2014 [72]	Use of Social Media to Conduct a Cross-Sectional Epidemiologic and Quality of Life Survey of Patients with Neuroendocrine Carcinoma of the Cervix: A Feasibility Study	North America	Neuroendocrine carcinoma of the cervix	Cross-sectional	Primary	1	Tumour-specific questionnaire		PROMIS-sexual, FACT-Cx

[a] Generic questionnaire: SF-12: Short-form 12 questionnaire

[b] Tumour/domain-specific questionnaire: FACT-O: Functional Assessment of Cancer Therapy - Ovarian; FACT-O-TOI: Functional Assessment of Cancer Therapy – Ovarian Trial Outcome Index; FSFI: Female Sexual Function Index Questionnaire, PROMIS-sexual: Sexual Scale from PROMIS; FACT-Cx: Functional Assessment of Cancer Therapy – Cervix

Supplementary table 2.3. EURACAN G3: GU - Rare cancer of the male genital organs, and of the urinary tract

Author, year [ref]	Article title	Continent	Subtypes of cancer	Study design*	HRQoL end point	Number of HRQoL assessments	Type of HRQoL assessment	Generic questionnaire [a]	Tumour/domain-specific questionnaire [b]
Licht et al., 2021 [73]	Evaluation by electronic patient-reported outcomes of cancer survivors' needs and the efficacy of inpatient cancer rehabilitation in different tumour entities	Europe	Prostate cancer; Testicular cancer	Cohort	Primary	2	Generic questionnaire	EORTC QLQ-C30	
Hartung et al., 2016 [74]	Age-related variation and predictor of long-term quality of life in germ cell tumour survivors	Europe	Germ cell tumour	Cross-sectional	Primary	1	Generic questionnaire	SF-8	
Højer et al., 2022 [75]	Effect of Testosterone Replacement Therapy on Quality of Life and Sexual Function in Testicular Cancer Survivors With Mild Leydig Cell Insufficiency: Results From a Randomized Double-blind Trial	Europe	Testicular cancer	RCT	Primary	4	Generic and tumour-specific questionnaire	EORTC QLQ-C30	IIEF-15
Saoud et al., 2020 [76]	Impact of Non–guideline directed Care on Quality of Life in Testicular Cancer Survivors	North America	Testicular cancer	Cohort	Primary	1	Generic questionnaire	EORTC QLQ-C30	
Schmidt et al., 2018 [77]	Limited post-chemotherapy retroperitoneal resection of residual tumour in non seminomatous testicular cancer: complications, outcome and quality of life	Europe	Germ cell testicular cancer	Cross-sectional	Primary	1	Generic questionnaire	EORTC QLQ-C30	
Skøtt et al., 2018 [78]	Quality of life in long-term testicular cancer survivors with compensated Leydig cell dysfunction	Europe	Testicular cancer	Observational cohort	Primary	1	Generic and tumour-specific questionnaire	EORTC QLQ-C30	IIEF-15
van Dongen et al., 2022 [70]	Challenges and controversies patients and (health care) professionals experience in managing vaginal, vulvar, penile or anal cancer: The SILENCE study	Europe	Penile cancer	Qualitative exploratory	Primary	1	Interview		
Jovanovski et al., 2021 [79]	Quality of life among germ-cell testicular cancer survivors: The effect of time since cancer diagnosis	Europe	Germ cell testicular cancer	Case-control	Primary	1	Generic questionnaire	SF-12	
Nezu et al., 2022 [80]	Association of financial toxicity with quality of life in testicular cancer survivors	Asia	Testicular cancer	Cross-sectional	Primary	1	Generic and tumour-specific questionnaire	EORTC QLQ-C30	EORTC QLQ-TC26
Nicolai et al., 2015 [81]	Quality of life and pain control following laparoscopic retroperitoneal lymph node dissection in early-stage nonseminoma	Europe	Germ cell tumour	Non-RCT	Primary	3	Generic questionnaire	FACT-G, FACT-T-SG	
Witty et al., 2013 [82]	The impact of surgical treatment for penile cancer - Patients' perspectives	Europe	Penile cancer	Narrative history design	Primary	1	Interview		
Pérez et al., 2020 [83]	Oncological and Functional Outcomes After Organ-Sparing Plastic Reconstructive Surgery for Penile Cancer	South America	Penile cancer	Cross-sectional	Primary	1	Generic and tumour-specific questionnaire	EQ-5D-3L	IIEF-5, ICIQ-MLUTS

Chavarriaga et al., 2022 [84]	Inverted urethral flap reconstruction after partial penectomy: Long-term oncological and functional outcomes	South America	Penile cancer	Cross-sectional	Secondary	1	Generic and tumour-specific questionnaire	EQ-5D-3L	IIEF-5, ICIQ-MLUTS
Khanal et al., 2020 [85]	The effects of hypogonadism on quality of life in survivors of germ cell tumours treated with surgery alone versus surgery plus platinum-based chemotherapy	North America	Germ cell tumour	Non-RCT	Primary	1	Generic questionnaire	SF-36, PROMIS	AMS scale
Flechtner et al., 2015 [86]	Quality-of-Life Analysis of the German Prospective Multicentre Trial of Single-cycle Adjuvant BEP Versus Retroperitoneal Lymph Node Dissection in Clinical Stage I Nonseminomatous Germ Cell Tumours	Europe	Germ cell tumour	RCT	Primary	36	Generic questionnaire	EORTC QLQ-C30	

[*]RCT: Randomized Controlled Trial; Non-RCT: Nonrandomized Controlled Trial

[a] Generic Questionnaire: EORTC QLQ-C30: European Organization for Research and Treatment for Cancer Quality of Life Questionnaire-Core30; SF-8: Abbreviated Version of 36-Item Health Survey; SF-12: 12-Item Short Form Survey; FACT-G: Functional Assessment of Cancer Therapy – General; FACT-T-SG: Functional Assessment of Chronic Illness Therapy; EQ-5D-3L: EUroQoL-5 Dimension- 3 level; SF-36: Short form health survey; PROMIS: Patient-Reported Outcomes Management Information System

[b] Tumour/domain-specific questionnaire: IIEF-5: Index of Erectile Function; EORTC QLQ-TC26: EORTC Quality of Life Tumour-specific Testicular Questionnaire; ICIQ-MLUTS: International Consultation on Incontinence Modular Questionnaire for Male Lower Urinary Tract Symptoms; AMS: Aging Male’s symptoms scale

Supplementary table 2.4. EURACAN G4: NET - Rare cancer of the neuroendocrine system

Author, year [ref]	Article title	Continent	Subtypes of Cancer*	Study design**	HRQoL end point	Number of HRQoL assessments	Type of HRQoL assessment	Generic questionnaire [a]	Tumour/domain-specific questionnaire [b]
Ballal et al., 2019 [87]	Broadening horizons with 225Ac-DOTATATE targeted alpha therapy for gastroenteropancreatic neuroendocrine tumour patients stable or refractory to 177Lu-DOTATATE PRRT: first clinical experience on the efficacy and safety	Asia	GEP-NET	Cohort	Secondary	2	Tumour-specific questionnaire		EORTC QLQ-GI.NET21
Beesley et al., 2018 [88]	Perceptions of care and patient-reported outcomes in people living with neuroendocrine tumours	Oceania	Lung NET; GI-NET; P-NET	Cross-sectional	Primary	1	Generic and tumour-specific questionnaire	FACT-G	EORTC QLQ-GI.NET21
Begum et al., 2022 [89]	Anxiety, Depression and Quality of Life in Patients with Neuroendocrine Neoplasia After Surgery	Europe	GI-NET	Longitudinal	Primary	5	Generic and tumour-specific questionnaire	EORTC QLQ-C30	EORTC QLQ-GI.NET21
Laing et al., 2022 [90]	Prevalence of malnutrition and nutrition related complications in patients with gastroenteropancreatic neuroendocrine tumours	Oceania	GEP-NET	Cross-sectional	Secondary	12	Generic and tumour-specific questionnaire	EORTC QLQ-C30	EORTC QLQ-GI.NET21
Lamarca et al., 2018 [91]	Somatostatin analogue-induced pancreatic exocrine insufficiency in patients with neuroendocrine tumours: results of a prospective observational study	Europe	Lung NET; GI-NET; P-NET	Observational	Secondary	6	Generic and tumour-specific questionnaire	EORTC QLQ-C30	EORTC QLQ-GI.NET21
Lewis et al., 2018 [92]	Health-related quality of life, anxiety, depression and impulsivity in patients with advanced gastroenteropancreatic neuroendocrine tumours	Europe	GEP-NET	Cross-sectional	Primary	1	Generic and tumour-specific questionnaire	EORTC QLQ-C30	EORTC QLQ-GI.NET21
Hummelshøj et al., 2023 [93]	Fatigue and quality of life in patients with neuroendocrine neoplasia	Europe	GI-NET; lung NET; P-NET	Cross-sectional	Primary	1	Generic questionnaire	EQ-5D-5L	
Scandurra et al., 2021[94]	Quality of Life in Patients with Neuroendocrine Neoplasms: The Role of Severity, Clinical Heterogeneity, and Resilience	Europe	GI-NET; lung NET; thyroid; adrenal; paraganglia; oropharynx NET	Cross-sectional	Primary	1	Generic and tumour-specific questionnaire	EORTC QLQ-C30	EORTC QLQ-GI.NET21
Swinburn et al., 2012 [95]	Elicitation of health state utilities in neuroendocrine tumours	Europe	Neuroendocrine: not specified	Qualitative	Primary	1	Interview + health vignettes		
Martini et al., 2018 [96]	Quality of Life in Patients with Metastatic Gastroenteropancreatic Neuroendocrine Tumors Receiving Peptide Receptor Radionuclide Therapy: Information from a Monitoring Program in Clinical Routine	Europe	GEP-NET	Cohort	Secondary	4	Generic questionnaire	EORTC QLQ-C30	
Meng et al., 2017 [97]	Patient-reported health state utilities in metastatic gastroenteropancreatic neuroendocrine tumours – an analysis based on the CLARINET study	Europe	GEP-NET	RCT	Primary	6	Generic questionnaire	EORTC QLQ-C30	

Meyer et al., 2014 [98]	Capecitabine and streptozocin ± cisplatin in advanced gastroenteropancreatic neuroendocrine tumours	Europe	GI.NET; P-NET	RCT	Secondary	4	Generic questionnaire	EORTC QLQ-C30	
Mitry et al., 2014 [99]	Bevacizumab plus capecitabine in patients with progressive advanced well-differentiated neuroendocrine tumors of the gastro-intestinal (GI-NETs) tract (BETTER trial) – A phase II non-randomised trial	Europe	GI-NET	Non-RCT	Secondary	6	Generic questionnaire	EORTC QLQ-C30	
Modica et al., 2022 [100]	Health-related quality of life in patients with neuroendocrine neoplasms: a two-wave longitudinal study	Europe	GI.NET; P-NET	Longitudinal	Primary	2	Generic and tumour-specific questionnaire	EORTC QLQ-C30	EORTC QLQ-GI.NET21
van Leeuwaarde et al., 2022 [101]	A Multinational Pilot Study on Patients' Perceptions of Advanced Neuroendocrine Neoplasms on the EORTC QLQ-C30 and EORTC QLQ-GINET21 Questionnaires	Europe; North America; South America	GI.NET; P-NET	Cross-sectional	Primary	1	Generic and tumour-specific questionnaire	EORTC QLQ-C30	EORTC QLQ-GI.NET21
Ohlsson et al., 2022 [102]	Relationship between somatostatin receptor expressing tumour volume and health-related quality of life in patients with metastatic GEP-NET	Europe	GEP-NET	Cross-sectional	Primary	1	Generic and tumour-specific questionnaire	EORTC QLQ-C30	EORTC QLQ-GI.NET21
Adams et al., 2019 [103]	Living With Neuroendocrine Tumors: Assessment of Quality of Life Through a Mobile Application	North America	GI-NET; P-NET; lung NET	Observational	Primary	3	Generic and tumour-specific questionnaire	EORTC QLQ-C30, PROMIS-29	EORTC QLQ-GI.NET21
Zandee et al., 2019 [104]	Symptomatic and Radiological Response to 177Lu-DOTATATE for the Treatment of Functioning Pancreatic Neuroendocrine Tumors	Europe	P-NET	Cohort	Secondary	2	Generic questionnaire	EORTC QLQ-C30	
Pavel et al., 2017 [105]	Health-related quality of life for everolimus versus placebo in patients with advanced, non-functional, well-differentiated gastrointestinal or lung neuroendocrine tumours (RADIANT-4): a multicentre, randomised, double-blind, placebo-controlled, phase 3 trial	Europe North America	GI.NET; lung NET	RCT	Secondary	At least 7	Generic questionnaire	FACT-G	
Khan et al., 2011 [106]	Quality of Life in 265 Patients with gastroenteropancreatic or Bronchial Neuroendocrine Tumors Treated with [177Lu-DOTA0,Tyr3]Octreotate	Europe	GEP-NET	RCT	Primary	2	Generic questionnaire	EORTC QLQ-C30	
Traub-Weidinger et al., 2011 [107]	Improved Quality of Life in Patients Treated with Peptide Radionuclides	Europe	Lung NET	Longitudinal	Secondary	6	Generic questionnaire	VAS-5	

[*] GEP-NET: Gastroenteropancreatic neuroendocrine; P-NET: Neuroendocrine; NET: Neuroendocrine; GI-NET: Gastrointestinal neuroendocrine

** RCT: Randomized Controlled Trial; Non-RCT: Nonrandomized Controlled Trial

[a] Generic questionnaire: FACT-G: Functional Assessment of Cancer Therapy-General; EORTC QLQ-C30: European Organization for Research and Treatment for Cancer Quality of Life Questionnaire-Core30; EQ-5D-5L: EUroQoL-5 Dimension-5 level; PROMIS-29: Patient-Reported Outcome Measurement Information System; VAS-5: visual analogue scale

[b] Tumour/domain-specific questionnaire: EORTC QLQ-GI.NET21: EORTC Quality of Life Neuroendocrine Carcinoid Module; PROMIS-Sexual: Sexual scale of the Patient-Reported Outcome Measurement Information System; FACT-Cx: Functional Assessment of Cancer Therapy – Cervix

Supplementary table 2.5. EURACAN G5: Digestive Tract - Rare cancer of the digestive tract

Author, year [ref]	Article title	Continent	Subtypes of cancer*	Study design**	HRQoL end point	Number of HRQoL assessments	Type of HRQoL assessment	Generic questionnaire [a]	Tumour/domain -specific questionnaire [b]
Bentzen et al., 2013 [108]	Impaired health-related quality of life after chemoradiotherapy for anal cancer: Late effects in a national cohort of 128 survivors	Europe	Anal cancer	Cross-sectional	Primary	1	Generic and tumour-specific questionnaire	EORTC QLQ-C30	EORTC QLQ-CR29
Bourdais et al., 2021 [109]	Pulse-dose-rate interstitial brachytherapy in anal squamous cell carcinoma: clinical outcomes and patients' health quality perception	Europe	Anal cancer	Qualitative	Primary	1	Interview		
Bridgewater et al., 2016 [110]	Quality of life, long-term survivors and long-term outcome from the ABC-02 study	Europe	BTC	RCT	Primary	5	Generic and tumour-specific questionnaire	EORTC QLQ-C30	EORTC QLQ-PAN26
Goislard de Monsabert et al., 2021 [111]	Selective Internal Radiation Combined with Chemotherapy Maintains the Quality of Life in Intrahepatic Cholangiocarcinomas	Europe	Cholangiocarcinoma	RCT	Secondary	13	Generic questionnaire	EORTC QLQ-C30	
De et al., 2022 [112]	Patient-Reported Bowel and Urinary Function in Long-Term Survivors of Squamous Cell Carcinoma of the Anus Treated With Definitive Intensity Modulated Radiation Therapy And Concurrent Chemotherapy	North America	Anal cancer	Cross-sectional	Primary	1	Generic and tumour-specific questionnaire	FACT-G7	FIQoL, LARDS, ICIQ, MLUTS, FLUTS
Elberg Dengso et al., 2017 [113]	Health-related quality of life and anxiety and depression in patients diagnosed with cholangiocarcinoma: a prospective cohort study	Europe	Cholangiocarcinoma	Cohort	Primary	3	Generic questionnaire	EORTC QLQ-C30	
Fakhrian et al., 2013 [114]	Chronic adverse events and quality of life after radiochemotherapy in anal cancer patients	Europe	Anal cancer	Cross-sectional	Primary	1	Tumour-specific questionnaire		FACT-C
Lefevre et al., 2023 [115]	One-Year Treatment-Related Side Effects and Quality of Life After Chemoradiotherapy in Squamous Cell Carcinoma of the Anus	Europe	Anal cancer	Cross-sectional	Primary	2	Generic and tumour-specific questionnaire	EORTC QLQ-C30	EORTC QLQ-CR29
Liu et al., 2019 [116]	Implementation of comprehensive rehabilitation therapy in postoperative care of patients with cholangiocarcinoma and its impact on patients' quality of life	Asia	Cholangiocarcinoma	Experimental	Primary	1	Generic questionnaire	EORTC QLQ-C30	
Liu et al., 2022 [117]	Target nursing care on anxiety and depression in patients with gallbladder cancer during perioperative period	Asia	Gallbladder cancer	Cohort	Secondary	2	Generic questionnaire	SF-36	
Welzel et al., 2011 [118]	Quality of Life Outcomes in Patients with Anal Cancer after Combined Radiochemotherapy	Europe	Anal cancer	Cohort	Primary	1	Generic and tumour-specific questionnaire	EORTC QLQ-C30	EORTC QLQ-CR38

Woradet et al., 2015 [119]	Factors Affecting Health-Related Quality of Life in Patients With Cholangiocarcinoma in the Northeastern Region of Thailand	Asia; Europe	Cholangiocarcinoma	Longitudinal	Primary	2	Generic and tumour-specific questionnaire	FACT-G	FACT-Hep
Woradet et al., 2016 [120]	Health-Related Quality of Life and Survival of Cholangiocarcinoma Patients in Northeastern Region of Thailand	Asia; Europe	Cholangiocarcinoma	Longitudinal	Primary	3	Generic and tumour-specific questionnaire	FACT-G	FACT-Hep
Wu et al., 2017 [121]	Percutaneous Intraductal Radiofrequency Ablation for Extrahepatic Distal Cholangiocarcinoma: A Method for Prolonging Stent Patency and Achieving Better Functional Status and Quality of Life	Asia	Cholangiocarcinoma	Cohort	Secondary	5	Tumour-specific questionnaire		FACT-Hep
Edeline et al., 2019 [122]	Gemcitabine and Oxaliplatin Chemotherapy or Surveillance in Resected Biliary Tract Cancer (PRODIGE 12-ACCORD 18-UNICANCER GI): A Randomized Phase III Study	Europe	BTC	RCT	Primary	5	Generic questionnaire	EORTC QLQ-C30	
Han et al., 2014 [123]	Prospective Evaluation of Acute Toxicity and Quality of Life After IMRT and Concurrent Chemotherapy for Anal Canal and Perianal Cancer	North America	Anal Cancer; Perianal cancer	Cohort	Primary	At least 5	Generic and tumour-specific questionnaire	EORTC QLQ-C30	EORTC QLQ-CR29
Hosni et al., 2022 [124]	Impact of Definitive Chemoradiation on Quality of- Life Changes for Patients With Anal Cancer: Long-term Results of a Prospective Study	North America	Anal cancer	Cohort	Primary	7	Generic and tumour-specific questionnaire	EORTC QLQ-C30	EORTC QLQ-CR29
Sangruangake et al., 2022 [125]	The Relationship between Unmet Need, Physical Symptoms, Psychological Well-Being and Health Related Quality of Life in Cholangiocarcinoma Survivors	Asia	Cholangiocarcinoma	Cross-sectional	Primary	1	Generic and tumour-specific questionnaire	FACT-G, CaSUN, MSAS-SF	FACT-Hep
Sauter et al., 2022 [126]	Quality of life in patients treated with radiochemotherapy for primary diagnosis of anal cancer	Europe	Anal cancer	Cross-sectional	Primary	1	Generic and tumour-specific questionnaire	EORTC QLQ-C30	EORTC QLQ-ANL27
Shen et al., 2023 [127]	Efficacy of Systemic Chemotherapy in Patients With Low-grade Mucinous Appendiceal Adenocarcinoma A Randomized Crossover Trial	Europe	Appendiceal cancer	RCT	Secondary	2	Generic and tumour-specific questionnaire	EORTC QLQ-C30	EORTC QLQ-OV28
Sodergren et al., 2023 [128]	International Validation of the EORTC QLQ-ANL27, a Field Study to Test the Anal Cancer-Specific Health-Related Quality-of-Life Questionnaire	Europe	Anal cancer	Validation	Primary	1	Generic and tumour-specific questionnaire	EORTC QLQ-C30	EORTC QLQ-ANL27
Somjaivong et al., 2011 [129]	The Influence of Symptoms, Social Support, Uncertainty, and Coping on Health-Related Quality of Life Among Cholangiocarcinoma Patients in Northeast Thailand	Asia; Europe	Cholangiocarcinoma	Cross-sectional	Primary	1	Generic questionnaire	FACT-G	
Soni et al., 2021 [130]	Nivolumab in gastric/gastroesophageal junction cancer: real-world data from UK Early Access to Medicines Scheme	Europe	Gastro/gastroesophageal junction cancer	Observational	Primary	13	Generic questionnaire	EQ-5D-3L	

Stearns et al., 2018 [131]	Long-term Quality of Life After Cytoreductive Surgery and Heated Intraperitoneal Chemotherapy for Pseudomyxoma Peritonei: A Prospective Longitudinal Study	Europe	Pseudomyxoma peritonei	Cross-sectional	Primary	7	Generic questionnaire	EORTC QLQ-C30	
Subramani et al., 2022 [132]	Outcome Following Percutaneous Transhepatic Biliary Drainage (PTBD) in Carcinoma Gallbladder: a Prospective Observational Study	Asia	Gallbladder cancer	Observational	Primary	2	Generic questionnaire	SF-36	
Mortensen et al., 2015 [133]	Patients use normalisation techniques to cope with the quality-of-life effects of anal cancer	Europe	Anal cancer	Qualitative	Primary	1	Interview		
Darwish Murad et al., 2013 [134]	Excellent Quality of Life After Liver Transplantation for Patients With Perihilar Cholangiocarcinoma Who Have Undergone Neoadjuvant Chemoradiation	North America	Cholangiocarcin oma	Cross-sectional	Primary	11	Generic and tumour-specific questionnaire	SF-36, EuroQoL	NIDDK-QA
Tang et al., 2015 [135]	Quality of life after intensity-modulated radiation therapy for anal cancer	North America	Anal cancer	Cohort	Primary	3	Tumour-specific questionnaire		FACT-C, MOS-SPS
van Dongen et al., 2022 [70]	Challenges and controversies patients and (health care) professionals experience in managing vaginal, vulvar, penile or anal cancer: The SILENCE study	Europe	Anal cancer	Qualitative exploratory	Primary	1	Interview		
Joseph et al., 2016 [136]	Patient reported quality of life after helical IMRT based concurrent chemoradiation of locally advanced anal cancer	North America	Anal cancer	Cohort	Primary	9	Generic and tumour-specific questionnaire	EORTC QLQ-C30	EORTC QLQ-CR29
Aggarwal et al., 2013 [137]	Evaluation of high-dose-rate intraluminal brachytherapy by percutaneous transhepatic biliary drainage in the palliative management of malignant biliary obstruction: a pilot study	Asia	BTC	RCT	Primary	2	Generic questionnaire	EORTC QLQ-C30	
Artifon et al., 2015 [138]	Surgery or EUS-guided choledochoduodenostomy for malignant distal biliary obstruction after ERCP failure	North America; South America	BTC	RCT	Primary	1	Generic questionnaire	SF-36	
Axelsson et al., 2022 [139]	Patient-reported QoL in anal cancer survivors 3 and 6 years after treatment—results from the Swedish national ANCA study	Europe	Anal cancer	Cross-sectional	Primary	2	Generic questionnaire	EORTC QLQ-C30	
Atkinson et al., 2019 [140]	Reliability and between-group stability of a health-related quality of life symptom index for persons with anal high-grade squamous intraepithelial lesions: an AIDS Malignancy Consortium Study (AMC-A03)	North America	Anal cancer	RCT	Primary	2	Generic questionnaire	PGIC	A-HRSI;
Ginesi et al., 2023 [141]	Patients with Anal Cancer Have Low Functional and High Symptomatic Health-Related Quality of Life Scores After Chemoradiation	North America	Anal cancer	Cross sectional	Primary	1	Tumour-specific questionnaire		EORTC QLQ-ANL27
Patel et al., 2021 [142]	Understanding Patient Experience in Biliary Tract Cancer: A Qualitative Patient Interview Study	North America; Europe	BTC	Qualitative	Primary	1	Interview		

Pedersen et al., 2019 [143]	Quality of life following salvage surgery for squamous cell carcinoma of the anus	Europe	Anal cancer	Cross sectional	Primary	1	Generic and tumour-specific questionnaire	EORTC QLQ-C30	EORTC QLQ-CR29
Corrigan et al., 2022 [144]	Patient-Reported Outcomes After Chemoradiation in Patients With Anal Cancer: A Qualitative Analysis	North America	Anal cancer	Qualitative and cross-sectional	Primary	1	Interview + generic questionnaires	FACT-G7	
Kaupp-Roberts et al., 2016 [145]	Validation of the EORTC QLQ-BIL21 questionnaire for measuring quality of life in patients with cholangiocarcinoma and cancer of the gallbladder	Europe	Cholangiocarcinoma; gallbladder cancer	Validation	Primary	3	Generic and tumour-specific questionnaire	EORTC QLQ-C30	EORTC QLQ-BIL21
Keilson et al,. 2022 [146]	Patient reported outcomes: Financial toxicity is a barrier to clinical trials and personalized therapy in cholangiocarcinoma	North America	Cholangiocarcinoma	Cross-sectional	Primary	1	Tumour specific questionnaire		FACT-Hep
Knowles et al., 2015 [147]	Late effects and quality of life after chemo-radiation for the treatment of anal cancer	Europe	Anal cancer	Cross-sectional	Primary	1	Generic and tumour-specific questionnaire	EORTC QLQ-C30	EORTC QLQ-CR38, MSKCC

[*] BTC: Biliary Tract Cancer

[**] RCT: Randomized Controlled Trial; Validation: includes validation and questionnaire development studies

[a] Generic questionnaire: EORTC QLQ-C30: European Organization for Research and Treatment for Cancer Quality of Life Questionnaire-Core30; FACT-G7: Functional Assessment of Cancer Therapy-General 7-item version; SF-36: Medical Outcome Study 36-item Short Form Health Survey; FACT-G: Functional Assessment of Cancer Therapy-General; EQ-5D-3L: EUroQoL-5 Dimension-3 level; EuroQoL: EUroQoL-5 Dimension; CASUN: Cancer Survivor Unmet Needs Questionnaires; MSAS-SF: Memorial Symptom Assessment Scale short form; PGIC: Patient's Global Impression Of Change Scale

[b] Tumour/domain-specific questionnaire: EORTC QLQ-CR29: EORTC Quality of Life Questionnaire Colorectal Cancer Module; EORTC QLQ-PAN26: EORTC Quality of Life Questionnaire Pancreatic Module; FIQoL: Fecal Incontinence Quality of Life; LARS: Low Anterior Resection Syndrome; ICIQ-FLUTS: International Consultation on Incontinence Modular Questionnaire for Female Lower Urinary Tract Symptoms; ICIQ-MLUTS: International Consultation on Incontinence Modular Questionnaire for Male Lower Urinary Tract Symptoms; FACT-C: Functional Assessment of Cancer Therapy – Colorectal; EORTC QLQ-CR38: EORTC Quality of Life Questionnaire Colorectal Cancer Module; FACT-Hep: Functional Assessment of Cancer Therapy – Hepatobiliary; EORTC QLQ-ANL27: EORTC Quality of Life Questionnaire Anal Module; EORTC QLQ-OV28: EORTC Quality of Life Questionnaire Ovarian Module; NIDDK-QA: The National Institute of Diabetes and Digestive and Kidney Disease Liver Transplant Quality of Life Assessment; MOS-SPS: Medical Outcomes Study Social Support Survey; A-HRSI: ANCHOR Health-Related Symptom Index; EORTC QLQ-BIL21: EORTC Quality of Life Questionnaire Cholangiocarcinoma and Gallbladder Module; MSKCC: Memorial Sloan-Kettering Cancer Centre Bowel Function Instrument

Supplementary table 2.6. EURACAN G6: Endocrine - Rare cancer of the endocrine organs

Author, year [ref]	Article title	Continent	Subtypes of cancer	Study design*	HRQoL end point	Number of HRQoL assessments	Type of HRQoL assessment	Generic questionnaire [a]	Tumour/domain-specific questionnaire [b]
Banihashem et al., 2020 [148]	Psychological Status and Quality of Life Associated with Radioactive Iodine Treatment of Patients with Differentiated Thyroid Cancer: Results of Hospital Anxiety and Depression Scale and Short-Form (36) Health Survey	Asia	Thyroid	Observational	Primary	4	Generic questionnaire	SF-36	
Blefari et al., 2022 [149]	Long-Term Health-Related Quality of Life Outcomes Following Thyroid Surgery for Malignant or Benign Disease: Deficits Persist in Cancer Survivors Beyond Five Years	Oceania	Thyroid	Cohort	Primary	1	Tumour-specific questionnaire		CHO-QOL-THY
Bongers et al., 2020 [150]	Differences in long-term quality of life between hemithyroidectomy and total thyroidectomy in patients treated for low-risk differentiated thyroid carcinoma	North America; Europe	Thyroid	Cross-sectional	Primary	1	Generic and tumour-specific questionnaire	EORTC QLQ-C30	EORTC QLQ-THY34
Borget et al., 2015 [151]	Quality of Life and Cost-Effectiveness Assessment of Radioiodine Ablation Strategies in Patients With Thyroid Cancer: Results From the Randomized Phase III ESTIMABL Trial	Europe	Thyroid	RCT	Primary	4	Generic questionnaire	SF-36, EQ-5L-5D	
Buttner et al., 2020 [152]	Quality of Life in Patients with Hypoparathyroidism After Treatment for Thyroid Cancer	Europe	Thyroid	Cross-sectional	Primary	1	Generic questionnaire	EORTC QLQ-C30	
Buttner et al., 2020 [153]	Quality of life of patients more than 1 year after surgery for thyroid cancer	Europe	Thyroid	Cross-sectional	Primary	1	Generic and tumour-specific questionnaire	EORTC QLQ-C30	EORTC QLQ-THY34
Qu et al., 2022 [154]	Analysis of the Rehabilitation Efficacy and Nutritional Status of Patients After Endoscopic Radical Thyroidectomy by Fast Track Surgery Based on Nutritional Support	Asia	Thyroid	Non-RCT	Secondary	2	Generic questionnaire	GQOLI-74	
Dadu et al., 2015 [155]	Efficacy of the Natural Clay, Calcium Aluminosilicate Anti-Diarrheal, in Reducing Medullary Thyroid Cancer-Related Diarrhea and Its Effects on Quality of Life: A Pilot Study	North America	Thyroid	Pilot trial	Secondary	3	Tumour-specific questionnaire		MDASI-THY
de Rooij et al., 2021 [156]	Symptom clusters in 1330 survivors of 7 cancer types from the PROFILES registry: A network analysis	Europe	Thyroid	Cross-sectional	Primary	1	Generic questionnaire	EORTC QLQ-C30	
Diamond-Rossi et al., 2020 [157]	Looking under the hood of "the Cadillac of cancers:" radioactive iodine-related craniofacial side effects among patients with thyroid cancer	Europe; North America	Thyroid	Qualitative	Primary	1	Interview		
Dingle et al., 2013 [158]	Salivary morbidity and quality of life following radioactive iodine for well differentiated thyroid cancer	North America	Thyroid	Cross-sectional	Primary	1	Tumour-specific questionnaire		XeQOLS, UW-QOL; MDADI
Doubleday et al., 2021 [159]	What is the experience of our patients with transient hypoparathyroidism after total thyroidectomy?	North America	Thyroid	Cohort	Primary	5	Interview + generic questionnaire	SF-12, EORTC QLQ-C30	

Frey et al., 2021 [160]	Impact of Permanent Post-thyroidectomy Hypoparathyroidism on Self-evaluation of Quality of Life and Voice	Europe	Thyroid	Observational	Primary		Generic and tumour-specific questionnaire	SF-36	VHI
Yaniv et al., 2022 [161]	Quality of life following lobectomy versus total thyroidectomy is significantly related to hypothyroidism	Europe	Thyroid	Cross-sectional	Primary	1	Tumour-specific questionnaire		THYPRO
Yu et al., 2021 [162]	Initial Experience with Proton Beam Therapy for Differentiated Thyroid Cancer	North America	Thyroid	Observational	Primary	2	Tumour-specific questionnaire		EORTC QLQ-H&N35
Lan et al., 2020 [163]	Quality of Life in Papillary Thyroid Microcarcinoma Patients Undergoing Radiofrequency Ablation or Surgery: A Comparative Study	Asia	Thyroid	Cross-sectional	Primary	1	Generic and tumour-specific questionnaire	SF-36	THYCA-QOL
Lan et al., 2021 [164]	The quality of life in papillary thyroid microcarcinoma patients undergoing lobectomy or total thyroidectomy: A cross sectional study	Asia	Thyroid	Cross-sectional	Primary	1	Generic and tumour-specific questionnaire	SF-36	THYCA-QOL
Lee et al., 2013 [165]	Comparative Analysis of Oncological Outcomes and Quality of Life After Robotic versus Conventional Open Thyroidectomy With Modified Radical Neck Dissection in Patients With Papillary Thyroid Carcinoma and Lateral Neck Node Metastases	Asia	Thyroid	Cohort	Primary	1	Tumour-specific questionnaire		VHI-1, SIS-6, NDII
Lee et al., 2016 [166]	Comparison of quality of life between open and endoscopic thyroidectomy for papillary thyroid cancer	Asia	Thyroid	Non-RCT	Primary	4	Generic questionnaire	EORTC QLQ-C30	
Lee et al., 2022 [167]	Quality of Life of Survivors of Thyroid Cancer Is Not Inferior to That in Subjects without Cancer: Long-Term after Over 5 Years	Asia	Thyroid	Cohort	Primary	1	Generic questionnaire	EQ-5L-5D, EQ-VAS	
Li et al., 2019 [168]	Risk Factors of Deterioration in Quality of Life Scores in Thyroid Cancer Patients After Thyroidectomy	Asia	Thyroid	Observational	Primary	1	Generic questionnaire	EORTC QLQ-C30	
Li et al., 2020 [169]	Health-related quality of life analysis in differentiated thyroid carcinoma patients after thyroidectomy	Asia	Thyroid	Cross-sectional	Primary	1	Generic questionnaire	SF-36	
Li et al., 2022 [170]	Comparison of quality of life and cosmetic result between open and transaxillary endoscopic thyroid lobectomy for papillary thyroid microcarcinoma survivors: A single-center prospective cohort study	Asia	Thyroid	Cohort	Primary	5	Generic questionnaire	EORTC QLQ-C30	
Licht et al., 2021 [73]	Evaluation by electronic patient-reported outcomes of cancer survivors' needs and the efficacy of inpatient cancer rehabilitation in different tumor entities	Europe	Thyroid	Cohort	Primary	2	Generic questionnaire	EORTC QLQ-C30	
Liu et al., 2019 [171]	Mindfulness-based stress reduction in patients with differentiated thyroid cancer receiving radioactive iodine therapy: a randomized controlled trial	Asia	Thyroid	RCT	Primary	3	Generic questionnaire	EORTC QLQ-C30	
Liu et al., 2021 [172]	Appearance characteristics of incision, satisfaction with the aesthetic effect, and quality of life in of thyroid cancer patients after thyroidectomy	Asia	Thyroid	Cross sectional	Primary	1	Generic and tumour-specific questionnaire	EORTC QLQ-C30	POSAS
Lubitz et al., 2017 [173]	Measurement and Variation in Estimation of Quality of Life Effects of Patients Undergoing Treatment for Papillary Thyroid Carcinoma	North America	Thyroid	Observational	Primary	3	Generic questionnaire	SF-6D, HUI2, HUI3	
Luddy et al., 2021 [174]	Patient Reported Outcome Measures of Health-Related Quality of Life and Asthenia after Thyroidectomy	North America	Thyroid	Observational	Primary	4	Generic questionnaire	SF-36	

Lan et al., 2020 [175]	Factors associated with health-related quality of life in papillary thyroid microcarcinoma patients undergoing radiofrequency ablation: a crosssectional prevalence study	Asia	Thyroid	Cross-sectional	Primary	1	Generic and tumour-specific questionnaire	SF-36	THYCA-QOL
Lv et al., 2021 [176]	Study on the correlation between postoperative mental flexibility, negative emotions, and quality of life in patients with thyroid cancer	Asia	Thyroid	Observational	Primary	1	Generic questionnaire	FACT-G	
Wang et al., 2018 [177]	Health-Related Quality of Life of Community Thyroid Cancer Survivors in Hangzhou, China	Asia	Thyroid	Cross-sectional	Primary	1	Generic questionnaire	EORTC QLQ-C30, SF-36	
Wirth et al., 2022 [178]	Quality of life and surgical outcome of ABBA versus endocats endoscopic thyroid surgery: a single center experience	Europe	Thyroid	Cohort	Primary	1	Generic questionnaire	SF-12	
Wirth et al., 2021 [179]	Patient-Reported Outcomes with Selpercatinib Treatment Among Patients with RET-Mutant Medullary Thyroid Cancer in the Phase I/II LIBRETTO-001 Trial	Europe; North America; Oceania	Thyroid	RCT	Primary	6	Generic questionnaire	EORTC QLQ-C30	
Wongwattana et al., 2021 [180]	A comparison of efficacy and quality of life between transoral endoscopic thyroidectomy vestibular approach (TOETVA) and endoscopic thyroidectomy axillo-breast approach (ETABA) in thyroid surgery: non-randomized clinical trial	Asia	Thyroid	Non-RCT	Secondary	1	Generic questionnaire	SF-12	
Wu et al., 2016 [181]	Psychological and behavioral intervention improves the quality of life and mental health of patients suffering from differentiated thyroid cancer treated with postoperative radioactive iodine-1312	Asia	Thyroid	Longitudinal	Primary	2	Generic questionnaire	EORTC QLQ-C30	
Haraj et al., 2019 [182]	Evaluation of the quality of life in patients followed for differentiated cancer of the thyroid	Africa	Thyroid	Cross sectional	Primary	1	Generic questionnaire	SF-36	
Hedman et al., 2016 [183]	Determinants of long-term quality of life in patients with differentiated thyroid carcinoma – a population-based cohort study in Sweden	Europe	Thyroid	Cross-sectional	Primary	1	Generic questionnaire	SF-36	
Hedman et al., 2017 [184]	Effect of Thyroid-Related Symptoms on Long-Term Quality of Life in Patients with Differentiated Thyroid Carcinoma: Population-Based Study in Sweden	Europe	Thyroid	Cross-sectional	Primary	1	Generic and tumour-specific questionnaire	SF-36	Study-specific questionnaire
Hedman et al., 2018 [185]	Fear of Recurrence and View of Life Affect Health-Related Quality of Life in Patients with Differentiated Thyroid Carcinoma: A Prospective Swedish Population-Based Study	Europe	Thyroid	Longitudinal	Primary	2	Generic and tumour-specific questionnaire	SF-36	Study-specific questionnaire
Huang et al., 2016 [186]	Quality of life and cosmetic result of single-port access endoscopic thyroidectomy via axillary approach in patients with papillary thyroid carcinoma	Asia	Thyroid	Longitudinal	Primary	2	Tumour-specific questionnaire		UW-QOL
Huang et al., 2023 [187]	Health literacy and fatigue, anxiety, depression, and somatic symptoms in patients with differentiated thyroid carcinoma from West China: A cross-sectional study	Asia	Thyroid	Cross-sectional	Primary	1	Generic questionnaire	SOMS-7	
Husson et al., 2013 [188]	Health-related quality of life and disease specific symptoms in long-term thyroid cancer survivors: A study from the population-based PROFILES registry	Europe	Thyroid	Cross-sectional	Primary	1	Generic and tumour-specific questionnaire	EORTC QLQ-C30	THYCA-QOL

Husson et al., 2013 [189]	Development of a disease-specific health-related quality of life questionnaire (THYCA-QOL) for thyroid cancer survivors	Europe	Thyroid	Validation	Primary	1	Generic and tumour-specific questionnaire	EORTC QLQ-C30	THYCA-QOL
Husson et al., 2013[190]	Fatigue Among Short- and Long-Term Thyroid Cancer Survivors: Results from the Population-Based PROFILES Registry	Europe	Thyroid	Cross-sectional	Secondary	1	Tumour-specific questionnaire		THYCA-QOL
Schoormans et al., 2020 [191]	Negative illness perceptions are related to poorer health-related quality of life among thyroid cancer survivors: Results from the PROFILES registry	Europe	Thyroid	Cross-sectional	Primary	1	Generic questionnaire	EORTC QLQ-C30	
Shen et al., 2021 [192]	Comparing quality of life between patients undergoing trans-areola endoscopic thyroid surgery and trans-oral endoscopic thyroid surgery	Asia	Thyroid	Longitudinal	Primary	3	Generic questionnaire	SF-36	
Singer et al., 2012 [193]	Quality of Life in Patients with Thyroid Cancer Compared with the General Population	Europe	Thyroid	Cross-sectional	Primary	1	Generic questionnaire	EORTC QLQ-C30	
Singer et al., 2016 [194]	Quality-of-Life Priorities in Patients with Thyroid Cancer: A Multinational European Organisation for Research and Treatment of Cancer Phase I Study	Europe	Thyroid	Validation	Primary	1	Interview/item list		
Song et al., 2014 [195]	Quality of Life After Robotic Thyroidectomy by a Gasless Unilateral Axillary Approach	Asia	Thyroid	Cross-sectional	Primary	1	Tumour-specific questionnaire		UW-QOL, QOL-TV
Song et al., 2021 [196]	Radiofrequency ablation versus total thyroidectomy in patients with papillary thyroid microcarcinoma located in the isthmus: a retrospective cohort study	Asia	Thyroid	Cohort	Primary	1	Tumour-specific questionnaire		THYCA-QOL
Steenaard et al., 2020 [197]	Patient and Partner Perspectives on Health-Related Quality of Life in Adrenocortical Carcinoma	Europe	Adrenocortical carcinoma	Qualitative	Primary	2	Interview		
Steenaard et al., 2022 [198]	Health-Related Quality of Life in Adrenocortical Carcinoma: Development of the Disease-Specific Questionnaire ACC-QOL and Results from the PROFILES Registry	Europe	Adrenocortical carcinoma	Validation	Primary	1	Generic and tumour-specific questionnaire	EORTC QLQ-C30	ACC-QOL
Maki et al., 2022 [199]	Fatigue and quality of life among thyroid cancer survivors without persistent or recurrent disease	Asia	Thyroid	Cross-sectional	Secondary	1	Generic questionnaire	SF-36	
Ming et al., 2022 [200]	Effect of radioiodine therapy under thyroid hormone withdrawal on health-related quality of life in patients with differentiated thyroid cancer	Asia	Thyroid	Observational	Primary	3	Generic and tumour-specific questionnaire	EORTC QLQ-C30	EORTC QLQ-THY34
Missaoui et al., 2022 [201]	Health-related quality of life in long-term differentiated thyroid cancer survivors: A cross-sectional Tunisian-based study	Africa	Thyroid	Cross-sectional	Primary	1	Generic questionnaire	SF-36	
Mols et al., 2018 [202]	Age-related differences in health-related quality of life among thyroid cancer survivors compared with a normative sample: Results from the PROFILES Registry	Europe	Thyroid	Cross-sectional	Primary	1	Generic questionnaire	EORTC QLQ-C30	
Mongelli et al., 2020 [203]	Financial burden and quality of life among thyroid cancer survivors	North America	Thyroid	Cross-sectional	Primary	1	Generic questionnaire	PROMIS-29	
Moon et al., 2021 [204]	Effect of Initial Treatment Choice on 2-year Quality of Life in Patients with Low-risk Papillary Thyroid Microcarcinoma	Asia	Thyroid	Longitudinal	Primary	5	Tumour-specific questionnaire		KT-QOL

Moss et al., 2021 [205]	Medullary Thyroid Cancer Patient's Assessment of Quality of Life Tools: Results from the QALM Study	Europe	Thyroid	RCT	Primary	1	Generic and tumour-specific questionnaire	EORTC QLQ-C30, COH-QOL	MDASI, COH QOL-THY, EORTC QLQ-GINET21
Rajamanickam et al., 2022 [206]	Quality of life comparison in thyroxine hormone withdrawal versus triiodothyronine supplementation prior to radioiodine ablation in differentiated thyroid carcinoma: a prospective cohort study in the Indian population	Asia; Europe	Thyroid	Cohort	Primary	1	Generic and tumour-specific questionnaire	EORTC QLQ-C30	EORTC QLQ-H&N35, Billewicz scale
Ramin et al., 2020 [207]	Health-related quality of life of thyroid cancer patients undergoing radioiodine therapy: a cohort real-world study in a reference public cancer hospital in Brazil	South America	Thyroid	Cohort	Primary	3	Generic and tumour-specific questionnaire	EORTC QLQ-C30	EORTC QLQ-H&N35
Rani et al., 2014 [208]	Examining recombinant human TSH primed 131I therapy protocol in patients with metastatic differentiated thyroid carcinoma: comparison with the traditional thyroid hormone withdrawal protocol	Asia	Thyroid	Cohort	Secondary	1	Generic questionnaire	Adapted version of the EORTC-QLG-C30	
Rogers et al., 2017 [209]	Health-related quality of life, fear of recurrence, emotional distress in patients treated for thyroid cancer	Europe	Thyroid	Cross-sectional	Primary	1	Generic and tumour-specific questionnaire	EORTC QLQ-C30	THYCA-QoL
Ryu et al., 2018 [210]	Development and Evaluation of a Korean Version of a Thyroid-Specific Quality-of-Life Questionnaire Scale in Thyroid Cancer Patients	Asia	Thyroid	Longitudinal	Primary	4	Tumour-specific questionnaire		KT-QOL, VHI-30
Taieb et al., 2011 [211]	Heath-related quality of life in thyroid cancer patients following radioiodine ablation	Europe	Thyroid	Longitudinal	Primary	2	Generic questionnaire	FACIT	
Tang et al., 2020 [212]	Recombinant human thyrotropin versus thyroid hormone withdrawal in an Asian population	Asia	Thyroid	Cross sectional	Primary	1	Generic and tumour-specific questionnaire	SF-36	Billewicz scale
Taylor et al., 2023 [213]	Health-related quality-of- life analyses from a multicenter, randomized, double blind phase 2 study of patients with differentiated thyroid cancer treated with lenvatinib 18 or 24 mg/day	North America; Europe; Oceania	Thyroid	RCT	Secondary	7	Generic questionnaire	FACT-G; EQ-5D-3L, VAS	
Vaisman et al., 2015 [214]	Is there a role for peptide receptor radionuclide therapy inmedullary thyroid cancer?	South America	Thyroid	Observational	Secondary	2	Generic questionnaire	SF-36	
van Gelder et al., 2020 [215]	Assessing health-related quality of life in cancer survivors: factors impacting on EORTC QLU-C10D-derived utility values	Europe; Oceania	Thyroid	Cross-sectional	Primary	1	Generic questionnaire	EORTC QLQ-C30, EORTC QLU-C10D	
van Gerwen et al., 2022 [216]	Patient-reported outcomes following total thyroidectomy and lobectomy in thyroid cancer survivors: an analysis of the profiles registry data	North America	Thyroid	Cross-sectional	Primary	1	Generic questionnaire	EORTC QLQ-C30	
Vega-Vázquez et al., 2015 [217]	Quality of life in patients with differentiated thyroid cancer at the general endocrinology clinics of the university hospital of puerto rico	Central America	Thyroid	Cross-sectional	Primary	1	Tumour-specific questionnaire		UW-QOL
Vissers et al., 2013 [218]	The impact of comorbidity on health related quality of life among cancer survivors: analyses of data from the profiles registry	Europe	Thyroid	Cross-sectional	Primary	1	Generic questionnaire	EORTC QLQ-C30	

Jeon et al., 2019 [219]	Quality of life in patients with papillary thyroid microcarcinoma managed by active surveillance or lobectomy: a cross-sectional study	Asia	Thyroid	Cross-sectional	Primary	1	Generic and tumour-specific questionnaire	SF-12	THYCA-QOL
Juzwiszyn et al., 2020 [220]	Quality of life and acceptance of illness in patients who underwent total thyroidectomy	Europe	Thyroid	Cross-sectional	Primary	1	Generic questionnaire	WHOQOL-BREF	
Nervo et al., 2021 [221]	Quality of life during treatment with lenvatinib for thyroid cancer: the patients' perspective beyond the medical evaluation	Europe	Thyroid	Observational	Primary	4	Generic questionnaire	EQ-5D-3L	
Nickel et al., 2019 [222]	Health-related quality of life after diagnosis and treatment of differentiated thyroid cancer and association with type of surgical treatment	Oceania	Thyroid	Qualitative	Primary	1	Interview		
Nygaard et al., 2013 [223]	A placebo-controlled, blinded and randomised study on the effects of recombinant human thyrotropin on quality of life in the treatment of thyroid cancer	Europe	Thyroid	RCT	Primary	2	Generic questionnaire	SF-36, VAS	
Xue et al., 2021 [224]	Supraclavicular approach of lobectomy improves quality of life for patients with unilateral papillary thyroid microcarcinoma: a prospective cohort study	Asia	Thyroid	Cohort	Secondary	2	Generic and tumour-specific questionnaire	SF-12	THYCA-QOL
Ahn et al., 2020 [225]	Quality of life in patients with papillary thyroid microcarcinoma according to treatment: total thyroidectomy with or without radioactive iodine ablation	Asia	Thyroid	Cross-sectional	Primary	1	Generic and tumour-specific questionnaire	SF-12	THYCA-QOL
Alyousef et al., 2022 [226]	Long-Term Quality of Life (5-15 Years Post-Thyroidectomy) of Thyroid Carcinoma Patients in Two Tertiary Care Hospitals	Asia	Thyroid	Cross-sectional	Primary	1	Tumour-specific questionnaire		EORTC QLQ-H&N43
Aschebrook-Kilfoy et al., 2015 [227]	Risk Factors for Decreased Quality of Life in Thyroid Cancer Survivors: Initial Findings from the North American Thyroid Cancer Survivorship Study	North America	Thyroid	Longitudinal	Primary	1	Tumour-specific questionnaire		COH-QOL-THY
Roerink et al., 2017 [228]	High prevalence of self-reported shoulder complaints after thyroid carcinoma surgery	Europe	Thyroid	Cross-sectional	Primary	1	Generic and tumour-specific questionnaire	EORTC QLQ-C30	DASH
Gal et al., 2013 [229]	Quality of Life Impact of External Beam Radiotherapy for Advanced Thyroid Carcinoma	North America	Thyroid	Cross-sectional	Primary	1	Tumour-specific questionnaire		QOL-RTI, QOL-RTI/H&N module
Gallop et al., 2015 [230]	A qualitative evaluation of the validity of published health utilities and generic health utility measures for capturing health-related quality of life (HRQL) impact of differentiated thyroid cancer (DTC) at different treatment phases	Europe; North America	Thyroid	Qualitative	Primary	1	Interview		
Gamper et al., 2015 [231]	Persistent quality of life impairments in differentiated thyroid cancer patients: results from a monitoring programme	Europe	Thyroid	Cross-sectional	Primary	6	Generic questionnaire	EORTC QLQ-C30	
Giani et al., 2021 [232]	Safety and quality of life data from an Italian expanded access program of lenvatinib for treatment of thyroid cancer	Europe	Thyroid	RCT	Secondary	7	Generic questionnaire	EORTC QLQ-C30,VAS	
Giusti et al., 2011 [233]	Five-year longitudinal evaluation of quality of life in a cohort of patients with differentiated thyroid carcinoma	Europe	Thyroid	Longitudinal	Primary	3	Tumour-specific questionnaire		Billewicz scale
Giusti et al., 2020 [234]	Evaluation of Quality of Life in Patients with Differentiated Thyroid Cancer by Means of the	Europe	Thyroid	Longitudinal	Primary	5	Tumour-specific questionnaire		THYPRO

	Thyroid-Specific Patient-Reported Outcome Questionnaire: A 5-Year Longitudinal Study								
Gkatzia et al., 2021 [235]	Quality of Life Survey Following Radioiodine Ablation in Patients with Differentiated Thyroid Cancer	Europe	Thyroid	Longitudinal	Primary	3	Generic questionnaire	SF-36	
Goswami et al., 2018 [236]	Benchmarking health-related quality of life in thyroid cancer versus other cancers and United States normative data	North America	Thyroid	Survey	Primary	1	Generic questionnaire	PROMIS-29	
Goswami et al., 2019 [237]	Clinical factors associated with worse quality-of-life scores in United States thyroid cancer survivors	North America	Thyroid	Survey	Primary	1	Generic questionnaire	PROMIS-29	
Gou et al., 2017 [238]	Health-related quality-of-life assessment in surgical patients with papillary thyroid carcinoma	Asia	Thyroid	Observational	Primary	5	Generic questionnaire	SF-36	
Pak et al., 2017 [239]	Impact of age and sex on the quality of life following radioactive iodine ablation in patients with thyroid cancer	Asia	Thyroid	Cross-sectional	Secondary	1	Generic questionnaire	15-item self developed questionnaire	
Li et al., 2022 [240]	Total or Near-total Thyroidectomy in treatment of Thyroid Cancer	Asia	Thyroid	Longitudinal	Secondary	1	Generic questionnaire	EORTC QLQ-C30	
Chan et el., 2021 [241]	Health-Related Quality of Life in Asian Differentiated Thyroid Cancer Survivors	Asia	Thyroid	Cross-sectional	Primary	1	Generic and tumour-specific questionnaire	EORTC QLQ-C30	THYCA-QoL
Chen et al., 2022 [242]	Association of Total Thyroidectomy or Thyroid Lobectomy With the Quality of Life in Patients With Differentiated Thyroid Cancer With Low to Intermediate Risk of Recurrence	Asia	Thyroid	Observational	Primary	5	Generic and tumour-specific questionnaire	EORTC QLQ-C30	THYCA-QoL
Chen et al., 2022 [243]	Reminiscence therapy care program as a potential nursing intervention to relieve anxiety, depression, and quality of life in older papillary thyroid carcinoma patients: A randomized, controlled study	Asia	Thyroid	RCT	Primary	5	Generic questionnaire	EORTC QLQ-C30	
Chen et al., 2021 [244]	Postoperative quality of life and cosmetic outcome between minimally invasive video-assisted thyroidectomy and bilateral axillo-breast approach robotic thyroidectomy: a single center retrospective cohort study	Asia	Thyroid	Cohort	Primary	1	Generic questionnaire	SF-36	
Choi et al., 2014 [245]	Impact of Postthyroidectomy Scar on the Quality of Life of Thyroid Cancer Patients	Asia	Thyroid	Observational	Primary	1	Generic questionnaire	DLQI	
Chow et al., 2021 [246]	Association between quality of life and patient-reported complications from surgery and radioiodine in early-stage thyroid cancer survivors: A matched-pair analysis	North America	Thyroid	Matched pair-analysis	Primary	1	Generic questionnaire	PROMIS-29	
Kim et al., 2016 [247]	Comparison of the Quality of Life for Thyroid Cancer Survivors Who Had Open Versus Robotic Thyroidectomy	Asia	Thyroid	Longitudinal	Primary	1	Tumour-specific questionnaire		VHI-10, SIS-6
Kong et al., 2019 [248]	Longitudinal Assessment of Quality of Life According to Treatment Options in Low-Risk Papillary Thyroid Microcarcinoma Patients: Active Surveillance or Immediate Surgery	Asia	Thyroid	Cohort	Primary	2	Tumour-specific questionnaire		KT-QOL

Gulsoy Kirnap et al., 2020 [249]	The effect of iatrogenic subclinical hyperthyroidism on anxiety, depression and quality of life in differentiated thyroid carcinoma	Asia; Europe	Thyroid	Cross-sectional	Primary	1	Generic questionnaire	SF-36	
Kurumety et al., 2019 [250]	Post-thyroidectomy neck appearance and impact on quality of life in thyroid cancer survivors	North America	Thyroid	Cross-sectional	Primary	1	Generic questionnaire	PROMIS-29	
Kent et al., 2015 [251]	Health-related quality of life in older adult survivors of selected cancers: data from the SEER-MHOS linked data resource	North America	Thyroid	Cross-sectional	Primary	1	Generic questionnaire	SF-36	
Husson et al., 2020 [252]	The EORTC QLQ-C30 Summary Score as Prognostic Factor for Survival of Patients with Cancer in the "Real-World": Results from the Population-Based PROFILES Registry	Europe	Thyroid	Cross-sectional	Primary	1	Generic questionnaire	EORTC QLQ-C30	
Yang et al., 2019 [253]	Clinical Treatment Efficacy of Total Thyroidectomy Combined with Radioactive Iodine on Treatment of Thyroid Cancer and Its Effect on the Quality of Life of Patients	Asia	Thyroid	Cohort	Primary	1	Generic questionnaire	EORTC QLQ-C30	
Hamdan et al., 2022 [254]	A longitudinal investigation of posttraumatic growth and its associated factors among head and neck cancer survivors	Asia	Thyroid	Longitudinal	Primary	2	Tumour-specific questionnaire		EORTC QLQ-H&N35
Patterson et al., 2022 [255]	Associations between markers of social functioning and depression and quality of life in survivors of head and neck cancer: Findings from the Head and Neck Cancer 5000 study	Europe	Thyroid	Cross-sectional	Primary	1	Generic and tumour-specific questionnaire	EORTC QLQ-C30	EORTC QLQ-H&N35
Barry et al., 2022 [66]	The Impact of Disease Progression on Health Related Quality of Life Outcomes in Patients With Oligometastatic Disease at 12 Months Post Stereotactic Body Radiation Therapy	North America; South America; Europe	Thyroid	Longitudinal	Secondary	4	Generic questionnaire	EORTC QLQ-C30	

[*] RCT: Randomized Controlled Trial; Non-RCT: Non-Randomized Controlled Trial; Validation: includes validation and questionnaire development studies

[a] Generic questionnaire: SF-36: Medical Outcome Study 36-item Short Form Health Survey; EORTC QLQ-C30: European Organization for Research and Treatment for Cancer Quality of Life Questionnaire-Core30; EQ-5D-5L: EuroQol 5-Dimensional Instrument- 5 Level; GQOLI-74: General Quality of Life Inventory; SF-12: Short-Form 12 Questionnaire; VAS: Visual Analogue Scale; SF-6D: Short-Form Six-Dimension Health Index; HUI-12: Health Utilities Index-12 item; HUI-13: Health Utilities Index-13item; FACT-G: Functional Assessment of Cancer Therapy-General; SOMS-7: Screening for Somatoform Symptoms-7; PROMIS-29: Patient-Reported Outcomes Measurement Information System; COH-QOL: City of Hope Quality of Life–Ostomy Questionnaire; FACIT: Functional Assessment of Chronic Illness & Therapy; EORTC QLU-C10D: EORTC Quality Of Life Utility – Core 10 Dimensions; WHOQoL-BREF: World Health Organization Quality of Life Questionnaire – BREF; EQ-5D-3L: EuroQoL 5-Dimensional Instrument- 3 Level; DLQI: The Dermatology Life Quality Index

[b] Tumour/domain-specific questionnaire: COH-QOL-THY: City of Hope Quality of Life Questionnaire – Thyroid Version; EORTC QLQ-THY34: EORTC Quality of Life Questionnaire - Thyroid Cancer; MDASI-THY: MD Anderson Symptom Inventory-Thyroid questionnaire; XeQOLS: Xerostomia Quality of Life Scale questionnaire; UW-QOL: The University of Washington Head and Neck Cancer Quality of Life Questionnaire; MDADI: MD Anderson Dysphagia Inventory; VHI: The Voice Handicap Index; THYPRO: Thyroid-Specific Patient Reported Outcome; EORTC QLQ-H&N35: European Organization for Research and Treatment for Cancer Quality of Life Questionnaire-Head and Neck Cancer35; THYCA-QOL: THYroid CANcer-Quality of Life; VHI-10: Voice Handicap Index; SIS-6: Swallowing Impairment Score; NDII: Neck Dissection Impairment Index; POSAS: Patient and Observer Scar Assessment Scale; QOL-TV: The Quality of Life Questionnaire-Thyroid Version; ACC-QOL: Adrenocortical Carcinoma Health-Related Quality Of Life Questionnaire; KT-QoL: Korean Version Of The Self-Reported Thyroid-Specific Quality Of Life Questionnaire For Thyroid Cancer Patients; EORTC QLQ-GI.NET21: EORTC Quality of Life Neuroendocrine Carcinoid Module; VHI-30: Voice Handicap Index – 30item; DASH: Disabilities Of The Arm, Shoulder And Hand Questionnaire; QOL-RTI: Quality Of Life-Radiation Therapy Instrument; QOL-RTI/H&N: Quality of Life–Radiation Therapy Instrument Head and Neck Companion Module; SIS-6: Swallowing Impairment Score

Supplementary table 2.7. EURACAN G7: Head & Neck - Rare cancer of the head and neck

Author, year [ref]	Article title	Continent	Subtypes of cancer	Study design*	HRQoL end point	Number of HRQoL assessments	Type of HRQoL assessment	Generic questionnaire [a]	Tumour/domain-specific questionnaire [b]
Bachmann et al., 2018 [256]	Well-being and quality of life among oral cancer patients - Psychological vulnerability and coping responses upon entering initial treatment	Europe	Oral cancer	Mixed-method	Primary	3	Interview + generic and tumour-specific questionnaire	EORTC QLQ-C30	EORTC QLQ-H&N35
Bajwa et al., 2016 [257]	High-dose-rate interstitial brachytherapy in oral cancer - Its impact on quality of life	Asia	Oral cancer	Longitudinal	Primary	5	Generic and tumour-specific questionnaire	EORTC QLQ-C30	EORTC QLQ-H&N35
Becker et al., 2016 [258]	Health-related quality of life in patients with nasal prosthesis	Europe	Nasal cavity	Cross-sectional	Primary	1	Tumour-specific questionnaire		UW-QOL
Becker et al., 2018 [259]	Health-related quality of life in patients with major salivary gland carcinoma	Europe	Salivary grand	Cross-sectional	Primary	1	Tumour-specific questionnaire		UW-QOL
Li et al., 2020 [260]	Longitudinal Trend of Health-Related Quality of Life During Concurrent Chemoradiotherapy and Survival in Patients With Stage II–IVb Nasopharyngeal Carcinoma	Asia	Nasopharyngeal carcinoma	Longitudinal	Primary	6	Generic questionnaire	EORTC QLQ-C30	
Pan et al., 2017 [261]	Concurrent chemoradiotherapy degrades the quality of life of patients with stage II nasopharyngeal carcinoma as compared to radiotherapy	Asia	Nasopharyngeal carcinoma	Cross-sectional	Primary	1	Generic and tumour-specific questionnaire	EORTC QLQ-C30	EORTC QLQ-H&N35
Bozec et al., 2021 [262]	Long-term functional and quality of life outcomes in laryngectomized patients after successful voice restoration using tracheoesophageal prostheses	Europe	Laryngeal; hypopharyngeal cancer	Cross-sectional	Primary	1	Interview + generic and tumour-specific questionnaire	EORTC QLQ-C30	EORTC QLQ-H&N35, VHI-10, DOSS
Iriya et al., 2017 [263]	Health-related quality of life of patients with squamous cell carcinoma: a comparison according to tumor location	South America	Oral cavity; pharynx; larynx	Cross-sectional	Primary	1	Generic and tumour-specific questionnaire	EORTC QLQ-C30	EORTC QLQ-H&N35, UW-QOL
Daugaard et al., 2017 [264]	Association between late effects assessed by physicians and quality of life reported by head-and-neck cancer survivors	Europe	Oral cavity; Pharynx; Larynx; Salivary glands	Cross-sectional	Primary	1	Generic questionnaire	EORTC QLQ-C30	
Davudov et al., 2020 [265]	Quality of life in patients with oral cancer treated by different reconstruction methods as measured by the EORTC QLQ-H&N43	Asia	Oral cancer	Longitudinal	Primary	1	Generic and tumour-specific questionnaire	EORTC QLQ-C30	EORTC QLQ-H&N43
de Vries et al., 2020 [266]	Frailty is associated with decline in health-related quality of life of patients treated for head and neck cancer	Europe	Mucosal; salivary gland	Observational	Primary	4	Generic questionnaire	EORTC QLQ-C30	
Deckard et al., 2015 [267]	Comparative analysis of quality-of-life metrics after endoscopic surgery for sinonasal neoplasms	North America	Sinonasal cancer	Cross sectional	Primary	1	Generic and tumour-	EQ-5D	SNOT-20, ASBQ, LKE

							specific questionnaire		
Derousseau et al., 2015 [268]	Long-term changes in quality of life after endoscopic resection of sinonasal and skull-base tumors	North America	Sinonasal; skull-base tumour	Longitudinal	Primary	4	Tumour-specific questionnaire		SNOT-20
Deschuymer et al., 2021 [269]	Randomized Clinical Trial on Reduction of Radiotherapy Dose to the Elective Neck in Head and Neck Squamous Cell Carcinoma: Results on the Quality of Life	Europe	Oral cavity; oropharynx; hypopharynx; larynx	RCT	Primary	9	Generic and tumour-specific questionnaire	EORTC QLQ-C30	EORTC QLQ-H&N35
Dinescu et al., 2016 [270]	Evaluation of health-related quality of life with EORTC QLQ-C30 and QLQ-H&N35 in Romanian laryngeal cancer patients	Europe	Laryngeal; hypopharyngeal cancer	Case-control	Primary	1	Generic and tumour-specific questionnaire	EORTC QLQ-C30	EORTC QLQ-H&N35
Doss et al., 2017 [271]	Changes in health-related quality of life of oral cancer patients treated with curative intent: experience of a developing country	Asia	Oral cancer	Longitudinal	Primary	1	Generic and tumour-specific questionnaire	FACT-G	FACT-H&N, MAQ
Farrugia et al., 2021 [272]	A Principal Component of Quality of Life Measures Is Associated with Survival for Head and Neck Cancer Patients Treated with Radiation Therapy	North America	Pharyngeal cancer; oral cavity; laryngeal cancer; salivary gland	Longitudinal	Primary	2	Generic questionnaire	EORTC QLQ-C30	
Si et al., 2017 [273]	Influence of endoscopic sinus surgery on the quality of life of patients with early nasopharyngeal carcinoma and the analysis of prognosis-related factors	Asia	Nasopharyngeal cancer	Longitudinal	Primary	1	Tumour-specific questionnaire		FACT-H&N, QLQ-H&N35
Yang et al., 2021 [274]	The impact of induction chemotherapy on long-term quality of life in patients with locoregionally advanced nasopharyngeal carcinoma: Outcomes from a randomised phase 3 trial	Asia	Nasopharyngeal cancer	RCT	Primary	2	Generic and tumour-specific questionnaire	EORTC QLQ-C30	EORTC QLQ-H&N35
Yanwei et al., 2022 [275]	Traditional Chinese medicine formula 01 for nasopharyngeal carcinoma (NPC01) for head & neck cancer and health-related quality of life: a retrospective study	Asia	Nasopharyngeal cancer	Observational	Primary	2	Generic and tumour-specific questionnaire	EORTC QLQ-C30	EORTC QLQ-H&N35
Lastrucci et al., 2017 [276]	Late toxicity, evolving radiotherapy techniques, and quality of life in nasopharyngeal carcinoma	Europe	Nasopharyngeal cancer	Cross-sectional	Primary	1	Generic and tumour-specific questionnaire	FACT-G, EQ-5D-3L	FACT-NP, PSS-HN, xeQOLS
Lee et al., 2022 [277]	Comparison of quality of life outcomes in a de-intensification treatment regimen for p16 + oropharyngeal cancer	North America	Oropharyngeal cancer	Cross-sectional	Primary	1	Tumour-specific questionnaire		UW-QOL
Liao et al., 2021 [278]	A preliminary study on ultrasound-guided percutaneous microwave ablation for palliative treatment of advanced head and neck malignancies	Asia	Head and neck	Longitudinal observational	Secondary	1	Tumour-specific questionnaire		UW-QOL
Licht et al., 2021 [73]	Evaluation by electronic patient-reported outcomes of cancer survivors' needs and the efficacy of inpatient cancer rehabilitation in different tumor entities	Europe	Nasal cavity; middle ear; accessory sinus; laryngeal cancer	Cohort	Primary	2	Generic questionnaire	EORTC QLQ-C30	

Linsen et al., 2018 [279]	Age- and localization-dependent functional and psychosocial impairments and health related quality of life six months after OSCC therapy	Europe	Oral cancer	Cross-sectional	Primary	1	Generic questionnaire	Bochum questionnaire	
Locati et al., 2020 [280]	Patients With Adenoid Cystic Carcinomas of the Salivary Glands Treated With Lenvatinib: Activity and Quality of Life	Europe	Salivary glands	Observational	Secondary	5	Generic and tumour-specific questionnaire	EORTC QLQ-C30, EQ-5D-5L	EORTC QLQ-H&N35
Loimu et al., 2015 [281]	Health-related quality of life of head and neck cancer patients with successful oncological treatment	Europe	Oropharyngeal cancer; laryngeal cancer; hypopharyneal cancer; nasopharyngeal cancer; nasal cavity	Longitudinal	Primary	4	Generic questionnaire	15D	
Nascimento et al., 2019 [282]	Impact of xerostomia on the quality of life of patients submitted to head and neck radiotherapy	South America	Oral cavity; laryngeal cancer; nasopharyngeal cancer; oropharyngeal cancer	Cross-sectional	Primary	1	Tumour-specific questionnaire		OHIP, XI
Lopez-Jornet et al., 2012 [283]	Assessing quality of life in patients with head and neck cancer in Spain by means of EORTC QLQ-C30 and QLQ-H&N35	Europe	Oral cancer; salivary gland; oral cavity; tongue	Cross-sectional	Primary	1	Generic and tumour-specific questionnaire	EORTC QLQ-C30	EORTC QLQ-H&N35
Lu et al., 2022 [284]	Impact of changes in psychological resilience during treatment with intensity-modulated radiotherapy on nasopharyngeal carcinoma patients: a prospective study	Asia	Nasopharyngeal cancer	Longitudinal	Primary	1	Generic and tumour-specific questionnaire	EORTC QLQ-C30	EORTC QLQ-H&N35
Chan et al., 2012 [285]	Quality of Life of Patients After Salvage Nasopharyngectomy for Recurrent Nasopharyngeal Carcinoma	Asia	Nasopharyngeal cancer	Longitudinal	Primary	2	Generic and tumour-specific questionnaire	EORTC QLQ-C30	EORTC QLQ-H&N35
Williamson et al., 2011 [286]	Quality of life after treatment of laryngeal carcinoma: a single centre cross-sectional study	Europe	Laryngeal cancer	Cross-sectional	Primary	1	Tumour-specific questionnaire		UW-QOL
Hamdan et al., 2022 [254]	A longitudinal investigation of posttraumatic growth and its associated factors among head and neck cancer survivors	Asia	Nasopharyngeal cancer; oral cancer	Longitudinal	Primary	2	Tumour-specific questionnaire		EORTC QLQ-H&N35
Handschel et al., 2013 [287]	Psychological aspects affect quality of life in patients with oral squamous cell carcinomas	Europe	Oral cancer	Cross-sectional	Primary	1	Generic questionnaire	Self-developed questionnaire	
Hegde et al., 2018 [288]	Patient-reported quality-of-life outcomes after de-escalated chemoradiation for human papillomavirus-positive oropharyngeal carcinoma: Findings from a phase 2 trial	North America	Oropharynx cancer	RCT	Secondary	5	Generic and tumour-specific questionnaire	FACT-G	UW-QOL, FACT-H&N

Hong et al., 2015 [289]	Quality of life of nasopharyngeal cancer survivors in China	Asia	Nasopharyngeal cancer	Cross-sectional	Primary	1	Generic questionnaire	EORTC QLQ-C30	
Horn et al., 2020 [290]	Prospective feasibility analysis of salvage surgery in recurrent oral cancer in terms of quality of life	Europe	Oral cancer	Cohort	Primary	5	Generic and tumour-specific questionnaire	EORTC QLQ-C30	EORTC QLQ-H&N35
Hsing et al., 2011 [291]	Comparison between free flap and pectoralis major pedicled flap for reconstruction in oral cavity cancer patients – A quality of life analysis	Asia	Oral cavity	Cross-sectional	Secondary	1	Tumour-specific questionnaire		UW-QOL
Huang et al., 2020 [292]	Quality of life and survival outcome for patients with nasopharyngeal carcinoma treated by volumetric-modulated arc therapy versus intensity-modulated radiotherapy	Asia	Nasopharyngeal cancer	Longitudinal	Primary	4	Generic and tumour-specific questionnaire	EORTC QLQ-C30	EORTC QLQ-H&N35
Huang et al., 2023 [293]	Survival and long-term quality-of-life of resectable stage III/IV hypopharyngeal carcinoma treated between concurrent chemoradiotherapy and surgery followed by (concurrent chemo) radiotherapy	Asia	Hypopharyngeal cancer	Cohort	Primary	4	Generic and tumour-specific questionnaire	EORTC QLQ-C30	EORTC QLQ-H&N35, VHI-30
Scott et al., 2021 [294]	Long-term quality of life & functional outcomes after treatment of oropharyngeal cancer	Europe	Oropharyngeal cancer	Longitudinal	Primary	2	Generic and tumour-specific questionnaire	EORTC QLQ-C30	EORTC QLQ-H&N35, MDADI, NDII, OSS
Soares et al., 2018 [295]	Assessment of quality of life in patients with advanced oral cancer who underwent mandibulectomy with or without bone reconstruction	South America	Oral cancer	Cross-sectional	Primary	1	Tumour-specific questionnaire		UW-QOL
Spiegel et al., 2019 [296]	Quality of life in patients after reconstruction with the supraclavicular artery island flap (SCAIF) versus the radial free forearm flap (RFFF)	Europe	Oral cavity; oropharynx cancer	Cross-sectional	Primary	1	Generic and tumour-specific questionnaire	EORTC QLQ-C30	EORTC QLQ-H&N35
Spinato et al., 2017 [297]	Multicenter research into the quality of life of patients with advanced oropharyngeal carcinoma with long-term survival associated with human papilloma virus	Europe	Oropharyngeal cancer	Cross-sectional	Primary	1	Generic and tumour-specific questionnaire	EORTC QLQ-C30	EORTC QLQ-H&N35
McDonald et al., 2019 [298]	Health-related quality of life in patients with T1N0 oral squamous cell carcinoma: selective neck dissection compared with wait and watch surveillance	Europe	Oral cancer	Cross-sectional	Primary	1	Tumour-specific questionnaire		UW-QOL
Mucke et al., 2015 [299]	Quality of life after different oncologic interventions in head and neck cancer patients	Europe	Oral cancer	Cross-sectional	Primary	1	Tumour-specific questionnaire		UW-QOL
Rana et al., 2015 [300]	Prospective study of the influence of psychological and medical factors on quality of life and severity of symptoms among patients with oral squamous cell carcinoma	Europe	Oral cancer	Observational	Primary	1	Generic and tumour-specific questionnaire	BSI	UW-QOL
Ranta et al., 2021 [301]	Long-term Quality of Life After Treatment of Oropharyngeal Squamous Cell Carcinoma	Europe	Oropharyngeal cancer	Chart analysis	Primary	1	Generic and tumour-specific questionnaire	EORTC QLQ-C30	EORTC QLQ-H&N35, MDADI

Riva et al., 2021 [302]	Quality of Life in Electrochemotherapy for Cutaneous and Mucosal Head and Neck Tumors	Europe	Oral cavity; Parotid gland; Laryngeal cancer	Observational	Primary	4	Generic and tumour-specific questionnaire	EORTC QLQ-C30	EORTC QLQ-H&N35
Rogers et al., 2016 [303]	Health related quality of life following the treatment of oropharyngeal cancer by transoral laser	Europe	Oropharyngeal cancer	Cohort	Primary	1	Tumour-specific questionnaire		UW-QOL
Ruhle et al., 2021 [304]	Surviving Elderly Patients with Head-and-Neck Squamous Cell Carcinoma—What Is the Long-Term Quality of Life after Curative Radiotherapy?	Europe	Nasopharyngeal cancer; oropharyngeal; hypopharynegal; oral cavity; laryngeal cancer	Cross-sectional	Primary	1	Generic and tumour-specific questionnaire	EORTC QLQ-C30	EORTC QLQ-H&N35
Thomas et al., 2021 [305]	Longitudinal Assessment of Frailty and Quality of Life in Patients Undergoing Head and Neck Surgery	North America	Oral cavity; laryngeal cancer; hypopharyngeal cancer	Cohort	Primary	4	Generic and tumour-specific questionnaire	EORTC QLQ-C30	EORTC QLQ-H&N35, VES-13, Fried's Frailty Index
Hung et al., 2018 [306]	Oncologic Results and Quality of Life in Patients With Squamous Cell Carcinoma of Hypopharynx After Transoral Laser Microsurgery	Asia	Hypopharynx	Longitudinal	Primary	1	Generic and tumour-specific questionnaire	EORTC QLQ-C30	EORTC QLQ-H&N35, VHI-30, MDADI
Lee et al., 2011 [307]	Health-related quality of life outcome evaluation for intensity-modulated radiotherapy versus helical tomotherapy using EORTC QLQ-C30 and EORTC QLQ-H&N35 core questionnaires for nasopharyngeal carcinomas	Asia	Nasopharyngeal cancer	Cohort	Primary	1	Generic and tumour-specific questionnaire	EORTC QLQ-C30	EORTC QLQ-H&N35
Tuomi et al., 2015 [308]	Health-related quality of life and voice following radiotherapy for laryngeal cancer – a comparison between glottic and supraglottic tumours	Europe	Laryngeal cancer	Longitudinal	Primary	2	Generic and tumour-specific questionnaire	EORTC QLQ-C30	EORTC QLQ-H&N35, S-SECEL
Tyler et al., 2020 [309]	Long-Term Quality of Life After Definitive Treatment of Sinonasal and Nasopharyngeal Malignancies	North America	Sinonasal; nasopharyngeal cancer	Cross-sectional	Primary	1	Generic and tumour-specific questionnaire	EQ-5D-VAS	MDASI-HN, ASBQ
van Nieuwenhuizen et al., 2018 [310]	Patient-reported physical activity and the association with health-related quality of life in head and neck cancer survivors	Europe	Oral cavity; oropharyngeal, laryngeal; hypopharyngeal cancer	Cross-sectional	Primary	1	Generic and tumour-specific questionnaire	EORTC QLQ-C30	EORTC QLQ-H&N35
Jang-Chun et al., 2014 [311]	Comparisons of quality of life for patients with nasopharyngeal carcinoma after treatment with different RT technologies	Asia	Nasopharyngeal cancer	Cross-sectional	Primary	1	Generic and tumour-specific questionnaire	SF-36	EORTC QLQ-H&N35
Nemeth et al., 2017 [312]	Importance of chewing, saliva, and swallowing function in patients with advanced oral cancer undergoing preoperative chemoradiotherapy: a prospective study of quality of life	Europe	Oral cancer	Cross-sectional	Primary	1	Tumour-specific questionnaire		UW-QOL, FACT-H&N

Xu et al., 2023 [313]	Effect of Percutaneous Endoscopic Gastrostomy on Quality of Life after Chemoradiation for Locally Advanced Nasopharyngeal Carcinoma: A Cross-Sectional Study	Asia	Nasopharyngeal cancer	Cross-sectional	Primary	1	Generic questionnaire	EORTC QLQ-C30	
Abel et al., 2017 [314]	Impact on quality of life of IMRT versus 3-D conformal radiation therapy in head and neck cancer patients: A case control study	Europe	Pharyngeal cancer; oropharyngeal cancer; oral cancer	Longitudinal	Primary	6	Generic and tumour-specific questionnaire	EORTC QLQ-C30	EORTC QLQ-H&N35
Achim et al., 2018 [315]	Long-term Functional and Quality-of-Life Outcomes After Transoral Robotic Surgery in Patients With Oropharyngeal Cancer	North America	Oropharyngeal cancer	Longitudinal cohort	Primary	4	Tumour-specific questionnaire		EAT-10, HNQOL, PSS-HN
Aro et al., 2016 [316]	Trends in the 15D health-related quality of life over the first year following diagnosis of head and neck cancer	Europe	Oral cavity; nasopharynx; oropharynx; hypopharynx, larynx; parotid gland; submandibular gland; paranasal sinuses; mandible	Cohort	Primary	4	Generic questionnaire	15D	
Azevedo et al., 2012 [317]	Vocal Handicap and Quality of Life After Treatment of Advanced Squamous Carcinoma of the Larynx and/or Hypopharynx	South America	Laryngeal cancer	Cross-sectional	Primary	1	Tumour-specific questionnaire		UW-QoL; VHI
Zaoui et al., 2018 [318]	Quality of life after nasal cancer resection – surgical versus prosthetic rehabilitation	Europe	Nasal cavity	Cohort	Primary	1	Generic and tumour-specific questionnaire	SF-36	ROE, FROI-17
Hamilton et al., 2021 [319]	Patient-reported outcome measures in patients undergoing radiotherapy for head and neck cancer	North America	Oral cavity; nasopharynx; oropharynx; hypopharynx; larynx; ear; carotid body; paranasal sinus; salivary gland	Cohort	Primary	8	Tumour-specific questionnaire		VHNSS
Yue et al., 2018 [320]	Long-term quality of life measured by the University of Washington QoL questionnaire (version 4) in patients with oral cancer treated with or without reconstruction with a microvascular free flap	Asia	Oral cancer	Cross-sectional	Primary	1	Tumour-specific questionnaire		UW-QOL
You et al., 2020 [321]	Comparison of functional outcomes and health-related quality of life one year after treatment in patients with oral and oropharyngeal cancer treated with three different reconstruction methods	Asia	Oral; oropharyngeal cancer	Cross-sectional	Primary	1	Generic and tumour-specific questionnaire	EORTC QLQ-C30	EORTC QLQ-H&N35, UW-QoL
Gabriele et al., 2020 [322]	Quality of life, swallowing and speech outcomes after oncological treatment for mobile tongue carcinoma	Europe	Tongue cancer	Case control	Primary	1	Generic and tumour-specific questionnaire	EORTC QLQ-C30	EORTC QLQ-H&N35, PSS-HN, MDADI

Glicksman et al., 2018 [323]	Sinonasal Quality of Life After Endoscopic Resection of Malignant Sinonasal and Skull Base Tumors	North America	Sinonasal cancer	Cohort	Primary	5	Tumour-specific questionnaire		SNOT-22
Goetz et al., 2020 [324]	Hospital Based Quality of Life in Oral Cancer Surgery	Europe	Oral cancer	Cross-sectional	Primary	1	Generic and tumour-specific questionnaire	EORTC QLQ-C30	EORTC QLQ-H&N35
Govers et al., 2016 [325]	Quality of life after different procedures for regional control in oral cancer patients: cross-sectional survey	Europe	Oral cavity	Cross-sectional	Primary	1	Generic and tumour-specific questionnaire	EQ-5D-3L	SQD
Guibert et al., 2011 [326]	Quality of life in patients treated for advanced hypopharyngeal or laryngeal cancer	Europe	Hypopharyngeal; laryngeal cancer	Cohort	Primary	1	Generic and tumour-specific questionnaire	EORTC QLQ-C30	EORTC QLQ-H&N35
de Pauli Paglioni et al., 2020 [327]	The impact of radiation caries in the quality of life of head and neck cancer patients	South America	Head and neck squamous cell carcinoma (not specified); salivary gland cancer	Case-control	Primary	1	Tumour-specific questionnaire		UW-QOL
Pan et al., 2017 [328]	Intensity-modulated radiotherapy provides better quality of life than two-dimensional conventional radiotherapy for patients with stage II nasopharyngeal carcinoma	Asia	Nasopharyngeal carcinoma	Cross-sectional	Primary	1	Generic and tumour-specific questionnaire	EORTC QLQ-C30	EORTC QLQ-H&N35
Patterson et al., 2022 [255]	Associations between markers of social functioning and depression and quality of life in survivors of head and neck cancer: Findings from the Head and Neck Cancer 5000 study	Europe	Oral cavity; Oropharynx; larynx; salivary gland	Cross-sectional	Primary	1	Generic and tumour-specific questionnaire	EORTC QLQ-C30	EORTC QLQ-H&N35
Peisker et al., 2016 [329]	Longterm quality of life after oncologic surgery and microvascular free flap reconstruction in patients with oral squamous cell carcinoma	North American; Europe	Oral cancer	Cohort	Primary	1	Generic and tumour-specific questionnaire	EORTC QLQ-C30	EORTC QLQ-H&N35
Petrides et al., 2022 [330]	Health-related quality of life in maxillectomy patients undergoing dentoalveolar rehabilitation	Oceania	Oral Cavity; oropharynx; maxillary sinus	Cross-sectional	Primary	1	Tumour-specific questionnaire		FACE-Q, SHI, MDADI
Chen et al., 2013 [331]	Scintigraphic assessment of salivary function after intensity-modulated radiotherapy for head and neck cancer: Correlations with parotid dose and quality of life	Asia	Oral cavity; parotid; larynx; oropharynx; Nasopharyngeal cancer	Cohort	Primary	5	Generic and tumour-specific questionnaire	EORTC QLQ-C30	EORTC QLQ-H&N35
Chen et al., 2015 [332]	Comparison of functional outcomes and quality of life between transoral surgery and definitive chemoradiotherapy for oropharyngeal cancer	North America	Oropharyngeal cancer	Case-control	Primary	1	Tumour-specific questionnaire		UW-QOL
Chen et al., 2015 [333]	Influence of Intensity-Modulated Radiation Therapy on the Life Quality of Patients with Nasopharyngeal Carcinoma	Asia	Nasopharyngeal cancer	RCT	Primary	1	Generic and tumour-specific questionnaire	SF-36	EORTC QLQ-H&N35, NPC follow-up form

Ch'ng et al., 2014 [334]	Prospective quality of life assessment between treatment groups for oral cavity squamous cell carcinoma	Oceania	Oral cavity	Longitudinal	Primary	4	Generic and tumour-specific questionnaire	EORTC QLQ-C30	EORTC QLQ-H&N35
Liao et al., 2021 [335]	Quality of Life as a Mediator between Cancer Stage and Long-Term Mortality in Nasopharyngeal Cancer Patients Treated with Intensity-Modulated Radiotherapy	Asia	Nasopharyngeal cancer	Longitudinal	Primary	5	Generic and tumour-specific questionnaire	EORTC QLQ-C30	EORTC QLQ-H&N35
Choby et al., 2015 [336]	Transoral Robotic Surgery Alone for Oropharyngeal Cancer Quality-of-Life Outcomes	North America	Oropharyngeal cancer	Cohort	Primary	5	Tumour-specific questionnaire		UW-QOL
Crombie et al., 2014 [337]	Health-related quality of life of patients treated with primary chemoradiotherapy for oral cavity squamous cell carcinoma: a comparison with surgery	Oceania	Oral cavity	Cross-sectional	Primary	1	Generic and tumour-specific questionnaire	EORTC QLQ-C30	EORTC QLQ-H&N35, UW-QOL
Kang et al., 2023 [338]	Pre-treatment quality of life in patients with salivary gland cancer in comparison with those of head and neck cancer patients	Asia	Salivary gland; oral cavity; oropharyngeal cancer	Cross-sectional	Primary	1	Generic and tumour-specific questionnaire	EORTC QLQ-C30	EORTC QLQ-H&N35
Kao et al., 2022 [339]	Early quality of life outcomes after surgery in head and neck cancer survivors with EORTC QLQ-C30 and EORTC QLQ-H&N35 in an Asian tertiary centre	Asia and Europe	Oral cavity; nasopharynx; oropharynx; hypopharynx; larynx; sinofacial; ear; salivary gland	Longitudinal	Primary	2	Generic and tumour-specific questionnaire	EORTC QLQ-C30	EORTC QLQ-H&N35
Kent et al., 2015 [251]	Health-related quality of life in older adult survivors of selected cancers: data from the SEER-MHOS linked data resource	North America	Oral cavity; pharynx	Cross-sectional	Primary	1	Generic questionnaire	SF-36, VR-12	
Barry et al., 2022 [66]	The Impact of Disease Progression on Health Related Quality of Life Outcomes in Patients With Oligometastatic Disease at 12 Months Post Stereotactic Body Radiation Therapy	North America; South America; Europe	Head and neck: not specified	Longitudinal	Secondary	4	Generic questionnaire	EORTC QLQ-C30	

[*]RCT: Randomized Controlled Trial

[a] Generic questionnaire: EORTC QLQ-C30: European Organization for Research and Treatment for Cancer Quality of Life Questionnaire-Core30; EQ-5D-3L: EuroQoL 5-dimensional instrument- 3 Level; EQ-5D-5L: EuroQoL 5-dimensional instrument- 5 Level; FACT-G: Functional Assessment of Cancer Therapy-General; 15D: Quality of Life Questionnaire; BSI: Brief Symptom Inventory; SF-36: Medical Outcome Study 36-item Short Form Health Survey; VR-12: The Veterans Rand 12-Item Health Survey

[b] Tumour/domain-specific questionnaire: EORTC QLQ-H&N35: European Organization for Research and Treatment for Cancer Quality of Life Questionnaire-Head and Neck Cancer35; UW-QOL: The University of Washington Head and Neck Cancer Quality of Life Questionnaire; VHI-10: Voice Handicap Index; DOSS: Dysphagia Outcomes and Severity Scale; EORTC QLQ-H&N43: European Organization for Research and Treatment for Cancer Quality of Life Questionnaire-Head and Neck Cancer43; SNOT-20: Sino-Nasal Outcome Test; ASBQ: Anterior Skull Base Questionnaire; LKE: Lund-Kennedy Endoscopic Score; FACT-H&N: Functional Assessment of Cancer Therapy – Head & Neck; MAQ: Malaysian Added Questions; FACT-NP: Functional Assessment of Cancer Therapy Nasopharyngeal Cancer Questionnaire; PSS-HN: Performance Status Scale for Head & Neck Cancer Patients; xeQOLS: Xerostomia-Related Quality of Life Scale; OHIP: Oral Health Impact Profile Questionnaire; XI: Xerostomia Inventory; VHI-30: Voice handicap index-30; MDADI: MD Anderson Dysphagia Inventory; NDII: Neck Dissection Impairment Index; OSS: Oxford Shoulder Score; VES-13: Vulnerable Elders Survey-13; S-SECEL: Swedish Self-Evaluation of Communication Experiences after Laryngeal Cancer; MDASI-HN: M.D. Anderson Symptom Inventory- Head and Neck Module; EAT-10: The Eating Assessment Tool; HNQOL: The Head and Neck Quality of Life Instrument; ROE: Rhinoplasty Outcome Evaluation; FROI-17: Functional Rhinoplasty Outcome Inventory-17; VNHSS: Vanderbilt Head and Neck Symptom Survey; SNOT-22: Sino-Nasal Outcome Test-22 Questionnaire; SQD: Shoulder Disability Questionnaire; FACE-Q: The FACE-Q Head and Neck Cancer Module; SHI: The Speech Handicap Index

Supplementary table 2.8. EURACAN G8: Thoracic - Rare cancer of the thorax

Author, year [ref]	Article title	Continent	Subtypes of cancer	Study design	HRQoL end point	Number of HRQoL assessments	Type of HRQoL assessment	Generic questionnaire [a]	Tumour/domain-specific questionnaire [b]
Burkholder et al., 2015 [340]	Effects of Extended Pleurectomy and Decortication on Quality of Life and Pulmonary Function in Patients With Malignant Pleural Mesothelioma	North America	Mesothelioma	Cohort	Primary	6	Generic questionnaire	EORTC QLQ-C30	
Lauk et al., 2021 [341]	Quality of Life Is Not Deteriorated After Extrapleural Pneumonectomy vs. (Extended) Pleurectomy/Decortication in Patients With Malignant Pleural Mesothelioma	Europe	Mesothelioma	Longitudinal	Primary	3	Generic and tumour-specific questionnaire	SF-36, EORTC QLQ-C15-PAL	EORTC QLQ-LC13
Eberst et al., 2019 [342]	Health-Related Quality of Life Impact from Adding Bevacizumab to Cisplatin-Pemetrexed in Malignant Pleural Mesothelioma in theMAPSIFCTGFPC- 0701 Phase III Trial	Europe	Mesothelioma	RCT	Secondary	10	Generic and tumour-specific questionnaire	EORTC QLQ-C30	EORTC QLQ-LC13
Scherpereel et al., 2022 [343]	First-line nivolumab plus ipilimumab versus chemotherapy for the treatment of unresectable malignant pleural mesothelioma: patient-reported outcomes in CheckMate 743	Asia; Europe; North America; Central America	Mesothelioma	RCT	Primary	16	Generic and tumour-specific questionnaire	EQ-5D-3L-VAS	LCSS-Meso, ASBI, 3-IGI
Soldera et al., 2019 [344]	Favourable health-related quality of life reported in survivors of thymic malignancies	North America	Thymoma	Cross-sectional	Primary	1	Generic questionnaire	EQ-5D-3L-VAS, ESAS	
Mollberg et al., 2012 [345]	Quality of Life After Radical Pleurectomy Decortication for Malignant Pleural Mesothelioma	North America	Mesothelioma	Cohort	Primary	4	Generic questionnaire	EORTC QLQ-C30	
Moore et al., 20222 [346]	Malignant pleural mesothelioma: treatment patterns and humanistic burden of disease in Europe	Europe	Mesothelioma	Cross-sectional	Primary	1	Generic and tumour-specific questionnaire	EQ-5D-3L-VAS	LCSS-Meso
Rena et al., 2012 [347]	Extrapleural pneumonectomy for early stage malignant pleural mesothelioma: A harmful procedure	Europe	Mesothelioma	Non-RCT	Primary	3	Generic questionnaire	EORTC QLQ-C30	
Tanaka et al., 2017 [348]	Physical function and health-related quality of life in patients undergoing surgical treatment for malignant pleural mesothelioma	Asia	Mesothelioma	Observational	Primary	1	Generic questionnaire	SF-36	
Tanaka et al., 2019 [349]	Physical function and health-related quality of life in the convalescent phase in surgically treated patients with malignant pleural mesothelioma	Asia	Mesothelioma	Observational	Primary	3	Generic questionnaire	SF-36	
Vigneswaran et al., 2018 [350]	Influence of Pleurectomy and Decortication in Health-Related Quality of Life Among Patients with Malignant Pleural Mesothelioma	North America	Mesothelioma	Longitudinal	Primary	5	Generic questionnaire	EORTC QLQ-C30	
Nagamatsu et al., 2018 [351]	Quality of life of survivors of malignant pleural mesothelioma in Japan: a cross sectional study	Asia	Mesothelioma	Cross-sectional	Primary	1	Generic questionnaire	Adapted version of the EORTC	

								QLQ-C30; CoQoLo	
Nakamichi et al., 2021 [352]	Quality of life and lung function after pleurectomy/decortication for malignant pleural mesothelioma	Asia	Mesothelioma	Cohort	Primary	4	Generic questionnaire	SF-36	
Ambrogi et al., 2012 [353]	Clinical Impact of Extrapleural Pneumonectomy for Malignant Pleural Mesothelioma	Europe	Mesothelioma	Cohort	Secondary	6	Generic and tumour-specific questionnaire	SF-36	SGRQ
Arnold et al., 2015 [354]	The effect of chemotherapy on health-related quality of life in mesothelioma: results from the SWAMP trial	Europe	Mesothelioma	RCT	Primary	5	Generic and tumour-specific questionnaire	EORTC QLQ-C30, EQ-5D-5L	EORTC QLQ-LC13
Granieri et al., 2013 [355]	Quality of life and personality traits in patients with malignant pleural mesothelioma and their first-degree caregivers	Europe	Mesothelioma	Cross-sectional	Primary	1	Generic questionnaire	WHOQOL-BREF	
Kao et al., 2012 [356]	Health-related quality of life and inflammatory markers in malignant pleural mesothelioma	Oceania	Mesothelioma	Cohort	Primary	1	Tumour-specific questionnaire		LCSS
Kirby et al., 2013 [357]	Quality of Life Study following Cyto reductive Surgery and Intraperitoneal Chemotherapy for pseudomyxoma Peritonei including Redo Procedures	Oceania	Pseudomyxoma Peritonei	Cross-sectional	Primary	1	Generic questionnaire	FACIT-TS-G	FACT-C

*RCT: Randomized Controlled trial; Non-RCT: Nonrandomized Controlled Trial

[a] Generic questionnaire: EORTC QLQ-C30: European Organization for Research and Treatment for Cancer Quality of Life Questionnaire-Core30; SF-36: Medical Outcome Study 36-item Short Form Health Survey; EORTC QLQ-C15-PAL: European Organization for Research and Treatment for Cancer Quality of Life Core Questionnaire for Palliative Care; EQ-5D-3L-VAS: EuroQoL 5-Dimensional Instrument- 3 Level Visual Analog Scale; ESAS: Edmonton Symptom Assessment Scale; CoQoLo: Comprehensive Quality of Life Outcome Questionnaire; EQ-5D-5L: EuroQoL 5-Dimensional Instrument- 5 Level; WHOQOL-BREF: World Health Organization Quality of Life–BREF; FACIT-TS-G: Functional Assessment of Chronic Illness Therapy - Treatment Satisfaction – General

[b] Tumour/domain-specific questionnaire: EORTC QLQ-LC13: EORTC Lung Module; LCSS Meso: Lung Cancer Symptom Scale-Mesothelioma; ASBI: Average Symptom Burden Index; 3-IGI: 3-Item Global Index; SGRQ: St. George’s Respiratory Questionnaire; LCSS: Lung Cancer Symptom Scale; FACT-C: Functional Assessment of Cancer Therapy-Colorectal

Supplementary table 2.9. EURACAN G9: Skin & Eye Melanoma - Rare cancer of the skin and eye melanoma

Author, year [ref]	Article title	Continent	Subtypes of cancer	Study design*	HRQoL end point	Number of HRQoL assessments	Type of HRQoL assessment	Generic questionnaire [a]	Tumour/domain-specific questionnaire [b]
Barker et al., 2020 [358]	Quality of Life Concerns in Patients with Uveal Melanoma after Initial Diagnosis	North America	Uveal Melanoma	Cross-sectional	Primary	1	Generic and tumour-specific questionnaire	EORTC QLQ-C30	EORTC QLQ-OPT30
Bharmal et al., 2020 [359]	Health-related quality of life trajectory of treatment-naive patients with Merkel cell carcinoma receiving avelumab	Europe; North America	Merkel cell carcinoma	RCT	Primary	12	Generic and tumour-specific questionnaire	EQ-5D-5L	FACT-M
Bharmal et al., 2017 [360]	Psychometric properties of the FACT-M questionnaire in patients with Merkel cell carcinoma	Europe; North America	Merkel cell carcinoma	RCT	Primary	4	Interview + Generic and tumour-specific questionnaire	EQ-5D-5L	FACT-M
Bharmal et al., 2019 [361]	Psychometric Properties of EQ-5D-5L Scoring Algorithms for the United Kingdom in Metastatic Merkel Cell Carcinoma	Europe	Merkel cell carcinoma	RCT	Primary	22	Generic questionnaire	EQ-5D-5L, FACT-G	FACT-M
Bharmal et al., 2020 [362]	Update on the psychometric properties and minimal important difference (MID) thresholds of the FACT-M questionnaire for use in treatment-naïve and previously treated patients with metastatic Merkel cell carcinoma	Europe; North America	Merkel cell carcinoma	RCT	Primary	1	Generic and tumour-specific questionnaire	EQ-5D-5L	FACT-M
Brown et al., 2022 [363]	Prediction of all-cause mortality from 24 month trajectories in patient-reported psychological, clinical and quality of life outcomes in uveal melanoma patients	Europe	Uveal Melanoma	Longitudinal	Primary	3	Generic and tumour-specific questionnaire	FACT-G	EORTC QLQ-OPT30
Damato et al., 2018 [364]	Patient-reported Outcomes and Quality of Life After Treatment of Choroidal Melanoma: A Comparison of Enucleation Versus Radiotherapy in 1596 Patients	Europe; North America	Choroidal Melanoma	Longitudinal	Primary		Generic and tumour-specific questionnaire	FACT-G	EORTC QLQ-OPT30
Damato et al., 2019 [365]	Patient-Reported Outcomes and Quality of Life after Treatment for Choroidal Melanoma.	Europe; North America	Choroidal Melanoma	Longitudinal	Primary	9	Generic and tumour-specific questionnaire	FACT-G	EORTC QLQ-OPT30
Frenkel et al., 2018 [366]	Long-term uveal melanoma survivors: measuring their quality of life	Asia	Uveal Melanoma	Cross-sectional	Primary	1	Generic and tumour-specific questionnaire	EORTC QLQ-C30	EORTC QLQ-OPT30
Lieb et al., 2020 [367]	Psychosocial impact of prognostic genetic testing in uveal melanoma patients: a controlled prospective clinical observational study	Europe	Uveal Melanoma	Non-RCT	Primary	4	Generic questionnaire	SF-12	
Wiley et al., 2013 [368]	Quality of Life and Cancer-Related Needs in Patients with Choroidal Melanoma	North America	Choroidal Melanoma	Cross-sectional	Primary	1	Generic and tumour-specific questionnaire	CNQSF	VFQ-25
Hope-Stone et al., 2016 [369]	Two-year patient reported outcomes following treatment of uveal melanoma	Europe	Uveal Melanoma	Longitudinal	Primary	3	Generic questionnaire	FACT-G	
Hope-Stone et al., 2019 [370]	Comparison between patient-reported outcomes after enucleation and proton beam radiotherapy for uveal melanomas: a 2-year cohort study	Europe	Uveal melanoma	Cohort	Primary	3	Tumour-specific questionnaire		EORTC QLQ-OPT30

Scannell et al, 2020 [371]	Quality of Life in Uveal Melanoma Patients in Ireland: A Single-Centre Survey	Europe	Uveal melanoma	Survey	Primary	1	Generic and tumour-specific questionnaire	EORTC QLQ-C30	EORTC QLQ-OPT30
Mouriaux et al., 2016 [372]	Sorafenib in metastatic uveal melanoma: efficacy, toxicity and health-related quality of life in a multicentre phase II study	Europe	Uveal melanoma	RCT	Secondary	5	Generic questionnaire	FACT-G	
van Beek et al., 2018 [373]	Quality of life: fractionated stereotactic radiotherapy versus enucleation treatment in uveal melanoma patients	Europe	Uveal melanoma	Cohort	Primary	7	Generic and tumour-specific questionnaire	EORTC QLQ-C30	VFQ-25
Vogl et al., 2017 [374]	Percutaneous Isolated Hepatic Perfusion as a Treatment for Isolated Hepatic Metastases of Uveal Melanoma: Patient Outcome and Safety in a Multi-centre Study	Europe	Uveal melanoma	Cohort	Secondary	1	Generic questionnaire	CTCAE, EORTC QLQ-C30	
Atkinson et al., 2017 [375]	Relationship between Physician-Adjudicated Adverse Events and Patient-Reported Health-Related Quality of Life in a Phase II Clinical Trial (NCT01143402) of Patients with Metastatic Uveal Melanoma	North America	Uveal melanoma	RCT	Primary	3	Generic and tumour-specific questionnaire	CTCAE	FACT-M
Gollrad et al., 2021 [376]	Quality of life and treatment-related burden during ocular proton therapy: a prospective trial of 131 patients with uveal melanoma	Europe	Uveal melanoma	Longitudinal cohort	Primary	4	Generic and tumour-specific questionnaire	EORTC QLQ-C30	EORTC QLQ-OPT30
Klingenstein et al., 2020 [377]	The national comprehensive cancer network distress thermometer as a screening tool for the evaluation of quality of life in uveal melanoma patients	Europe	Uveal melanoma	Cross-sectional	Primary	1	Generic questionnaire	NCCN	
Kaufman et al., 2018 [378]	Non progression with avelumab treatment associated with gains in quality of life in metastatic Merkel cell carcinoma	North America	Uveal melanoma	RCT	Primary	6	Generic and tumour-specific questionnaire	FACT-G, EQ-5D-5L	FACT-M
Klingenstein et al., 2013 [379]	Quality of life in the follow-up of uveal melanoma patients after CyberKnife treatment	Europe	Uveal melanoma	Cohort	Primary	3	Generic questionnaire	SF-12	
Klingenstein et al., 2016 [380]	Quality of life in the follow-up of uveal melanoma patients after enucleation in comparison to CyberKnife treatment	Europe	Uveal melanoma	Cross-sectional	Primary	1	Generic questionnaire	SF-12	
Kopp et al., 2017 [381]	The use of semi structured interviews to assess quality of life impacts for patients with uveal melanoma	North America	Uveal melanoma	Qualitative	Primary	1	Interview		
Gollrad et al., 2022 [382]	Impact of Adjuvant Ocular Interventions on the Quality of Life of Patients with Uveal Melanoma after Proton Beam Therapy	Europe	Uveal melanoma	Longitudinal	Primary	3	Generic and tumour-specific questionnaire	EORTC QLQ-C30	EORTC QLQ-OPT30

*RCT: Randomized Controlled Trial; Non-RCT: Nonrandomized Controlled Trial

[a] Generic questionnaire: EORTC QLQ-C30: European Organization for Research and Treatment for Cancer Quality of Life Questionnaire-Core30; EQ-5D- 5L: EuroQoL 5-dimensional 5-level version; FACT-G: Functional Assessment of Cancer Therapy-General; SF-12: Medical Outcome Study 12-item Short Form Health; CNQSF: Cancer Needs Questionnaire –Short form; CTCAE: Common Terminology Criteria for Adverse Events; NCCN: NCCN distress thermometer and problem List;

[B] Tumour/Domain-Specific Questionnaire: EORTC QLQ-OPT30: European Organization For Research And Treatment For Cancer Quality Of Life Questionnaire-Melanoma; FACT-M: Functional Assessment Of Cancer Therapy – Melanoma; VFQ-25: 25-item National Eye Institute Visual Function Questionnaire

Supplementary table 2.10. EURACAN G10 Brain – Rare cancer of the brain and spinal cord

Author, year [ref]	Article title	Continent	Subtypes of cancer	Study design*	HRQoL end point	Number of HRQoL assessments	Type of HRQoL assessment	Generic questionnaire [a]	Tumour/domain-specific questionnaire [b]
Bitterlich et al., 2017 [383]	Analysis of health-related quality of life in patients with brain tumors prior and subsequent to radiotherapy	Europe	Low grade; high grade glioma: 8 subtypes	Longitudinal	Primary	4	Generic and tumour-specific questionnaire	EORTC QLQ-C30	EORTC-BN20
Blonski et al., 2012 [384]	Combination of neoadjuvant chemotherapy followed by surgical resection as a new strategy for WHO grade II gliomas: a study of cognitive status and quality of life	Europe	Low grade glioma: 3 subtypes	Feasibility	Primary	1	Generic and tumour-specific questionnaire	EORTC QLQ-C30	EORTC-BN20
Boele et al., 2014 [385]	The association between cognitive functioning and health-related quality of life in low-grade glioma patients	Europe	Low-grade gliomas: 3 subtypes	Cross-sectional	Primary	1	Generic and tumour-specific questionnaire	SF-36	EORTC-BN20
Boele et al., 2015 [386]	Health-Related Quality of Life in Stable, Long-Term Survivors of Low-Grade Glioma	Europe	Low grade glioma	Longitudinal	Primary	2	Generic and tumour-specific questionnaire	SF-36	EORTC-BN20
Boele et al., 2022 [387]	Long-term wellbeing and neurocognitive functioning of diffuse low-grade glioma patients and their caregivers: A longitudinal study spanning two decades	Europe	Low grade glioma: 3 subtypes	Longitudinal	Primary	3	Generic and tumour-specific questionnaire	SF-36	EORTC-BN20
Bunevicius et al., 2014 [388]	Predictors of health-related quality of life in neurosurgical brain tumor patients: focus on patient-centered perspective	Europe	High grade; low grade glioma: not specified	Cross-sectional	Primary	1	Generic questionnaire	SF-36	
Bunevicius et al., 2017 [389]	Reliability and validity of the SF-36 Health Survey Questionnaire in patients with brain tumors: a cross-sectional study	Europe	High grade; low grade glioma: not specified	Cross-sectional	Primary	1	Generic questionnaire	SF-36	
Buvarp et al., 2021 [390]	Preoperative Patient-Reported Outcomes in Suspected Low-Grade Glioma: Markers of Disease Severity and Correlations with Molecular Subtypes	Europe	Low-grade glioma: 2 subtypes	Cohort	Primary	1	Generic and tumour-specific questionnaire	EORTC QLQ-C30, EQ-5D-3L	EORTC-BN20
Umezaki et al., 2020 [391]	Factors associated with health-related quality of life in patients with glioma: impact of symptoms and implications for rehabilitation	Asia	High grade; low grade glioma: 8 subtypes	Cross-sectional	Primary	1	Generic and tumour-specific questionnaire	EORTC QLQ-C30	EORTC-BN20
Daigle et al., 2013 [392]	Effects of surgical resection on the evolution of quality of life in newly diagnosed patients with glioblastoma: a report on 19 patients surviving to follow-up	North America	Glioblastoma	Longitudinal	Primary	2	Tumour-specific questionnaire		SNAS
Dirven et al., 2015 [393]	The impact of bevacizumab on health-related quality of life in patients treated for recurrent glioblastoma: results of the randomised controlled phase 2 BELOB trial	Europe	Glioblastoma	RCT	Primary	7	Generic and tumour-specific questionnaire	EORTC QLQ-C30	EORTC-BN20

Dirven et al., 2019 [394]	Impact of Radiation Target Volume on Health-Related Quality of Life in Patients With Low-Grade Glioma in the 2-Year Period Post Treatment: A Secondary Analysis of the EORTC 22033-26033	Europe	Low grade glioma: 3 subtypes	RCT	Primary	5	Generic and tumour-specific questionnaire	EORTC QLQ-C30	EORTC-BN20
Dirven et al., 2020 [395]	Neurocognitive functioning and health-related quality of life in adult medulloblastoma patients: long-term outcomes of the NOA-07 study	Europe	Medulloblastoma	Longitudinal	Secondary	16	Generic and tumour-specific questionnaire	EORTC QLQ-C30	EORTC-BN20
Drewes et al., 2016 [396]	Quality of life in patients with intracranial tumors: does tumor laterality matter?	Europe	High grade; low grade glioma: not specified	Cross-sectional	Primary	2	Generic questionnaire	EQ-5D-3L	
Drewes et al., 2018 [397]	Perioperative and Postoperative Quality of Life in Patients with Glioma-A Longitudinal Cohort Study	Europe	High grade; low grade glioma: not specified	Longitudinal	Primary	3	Generic questionnaire	EQ-5D-3L	
Dutzmann et al., 2013 [398]	A multi-center retrospective analysis of treatment effects and quality of life in adult patients with cranial ependymomas	Europe	Ependymomas	Cohort	Primary	1	Generic and tumour-specific questionnaire	EORTC QLQ-C30	EORTC-BN20
Field et al., 2017 [399]	Health-related quality of life outcomes from CABARET: a randomized phase 2 trial of carboplatin and bevacizumab in recurrent glioblastoma	Oceania	Glioblastoma	RCT	Secondary	16	Generic and tumour-specific questionnaire	EORTC QLQ-C30	EORTC-BN20
Flechl et al., 2012 [400]	Neurocognitive and sociodemographic functioning of glioblastoma long-term survivors	Europe	Glioblastoma	Cross-sectional	Primary	1	Generic and tumour-specific questionnaire	EORTC QLQ-C30	EORTC-BN20
Yavas et al., 2012 [401]	Health-related quality of life in high-grade glioma patients: a prospective single-center study	Asia	High grade glioma: not specified	Cohort	Primary	5	Generic and tumour-specific questionnaire	EORTC QLQ-C30	EORTC-BN20
Langegard et al., 2019 [402]	Evaluation of quality of care in relation to health-related quality of life of patients diagnosed with brain tumor: a novel clinic for proton beam therapy	Europe	Brain tumour: not specified	Longitudinal	Primary	3	Generic questionnaire	EORTC QLQ-C30, QPP	
Leonetti et al., 2021 [403]	Factors Influencing Mood Disorders and Health Related Quality of Life in Adults With Glioma: A Longitudinal Study	Europe	High grade; low grade gliomas: 6 subtypes	Longitudinal	Primary	5	Generic questionnaire	SF-36	
Licht et al., 2021 [73]	Evaluation by electronic patient-reported outcomes of cancer survivors' needs and the efficacy of inpatient cancer rehabilitation in different tumor entities	Europe	Brain tumour: not specified	Observational	Primary	2	Generic questionnaire	EORTC QLQ-C30	
Liu et al., 2020 [404]	Impact of neurosurgical enhanced recovery after surgery (ERAS) program on health-related quality of life in glioma patients: a secondary analysis of a randomized controlled trial	Asia	High grade; low grade gliomas: 5 subtypes	RCT	Primary	4	Generic and tumour-specific questionnaire	EORTC QLQ-C30	EORTC-BN20
Liu et al., 2018 [405]	Improvement of health related quality of life in patients with recurrent glioma treated with bevacizumab plus daily temozolomide as the salvage therapy	Asia	High grade; low grade gliomas: 3 subtypes	Longitudinal	Primary	3	Generic and tumour-specific questionnaire	EORTC QLQ-C30	EORTC-BN20

Lombardi et al., 2018 [406]	Quality of Life Perception, Cognitive Function, and Psychological Status in a Real-world Population, of Glioblastoma Patients Treated With Radiotherapy and Temozolomide	Europe	Glioblastoma	Longitudinal	Primary	5	Generic and tumour-specific questionnaire	EORTC QLQ-C30	EORTC-BN20
Waddle et al., 2019 [407]	Impacts of Surgery on Symptom Burden and Quality of Life in Pituitary Tumor Patients in the Subacute Post-operative Period	North America	Pituitary tumour	Longitudinal	Primary	2	Generic and tumour-specific questionnaire	EORTC QLQ-C30	EORTC-BN20
Wang et al., 2021 [408]	Comprehensive ability evaluation and trend analysis of patients with malignant intracranial tumors in the perisurgery period	Asia	High grade; low grade gliomas: not specified	Non-RCT	Primary	6	Generic and tumour-specific questionnaire	EORTC QLQ-C30	EORTC-BN20, ADL
Watanabe et al., 2022 [409]	Characteristics of health-related quality of life and related factors in patients with brain tumors treated with rehabilitation therapy	Asia	High grade; low grade gliomas: 9 subtypes	Cross-sectional	Primary	1	Generic and tumour-specific questionnaire	EORTC QLQ-C30, EQ-5D-5L	EORTC-BN20
Wefel et al., 2021 [410]	Neurocognitive, symptom, and health-related quality of life outcomes of a randomized trial of bevacizumab for newly diagnosed glioblastoma (NRG/RTOG 0825)	North America	Glioblastoma	RCT	Primary	14	Generic and tumour-specific questionnaire	EORTC QLQ-C30	EORTC-BN20
Svedung Wettervik et al., 2022 [411]	Patient-reported quality of life in grade 2 and 3 gliomas after surgery, can we do more?	Europe	High grade; low grade gliomas: 2 subtypes	Longitudinal	Primary	2	Generic questionnaire	RAND-36	
Wolf et al., 2016 [412]	Evaluation of neuropsychological outcome and quality of life after glioma surgery	Europe	High grade glioma: 5 subtypes	Non-RCT	Primary	3	Generic and tumour-specific questionnaire	EORTC QLQ-C30	EORTC-BN20
Habets et al., 2014 [413]	Health-related quality of life and cognitive functioning in long term anaplastic oligodendroglioma and oligoastrocytoma survivors	Europe	Low grade gliomas: 2 subtypes	Longitudinal	Primary	2	Generic and tumour-specific questionnaire	EORTC QLQ-C30	EORTC-BN20
Hickmann et al., 2017 [414]	Evaluating patients for psychosocial distress and supportive care needs based on health-related quality of life in primary brain tumors: a prospective multicenter analysis of patients with gliomas in an outpatient setting	Europe	High grade; low grade gliomas: 3 subtypes	Cross-sectional	Primary	1	Generic and tumour-specific questionnaire	EORTC QLQ-C30	EORTC-BN20, SCNS-SF34
Sagberg et al., 2016 [415]	Quality of survival the 1st year with glioblastoma: a longitudinal study of patient-reported quality of life	Europe	Glioblastoma	Longitudinal	Primary	8	Generic questionnaire	EQ-5D-3L	
Sagberg et al., 2019 [416]	Brain atlas for assessing the impact of tumor location on perioperative quality of life in patients with high-grade glioma: A prospective population based cohort study	Europe	High grade glioma: 3 subtypes	Cross-sectional	Primary	1	Interview + generic questionnaire	EQ-5D-3L	
Scartoni et al., 2020 [417]	Proton therapy re-irradiation preserves health-related quality of life in large recurrent glioblastoma	Europe	Glioblastoma	Longitudinal	Primary	4	Generic and tumour-specific questionnaire	EORTC QLQ-C30	EORTC-BN20

Seekatz et al., 2017 [418]	Screening for symptom burden and supportive needs of patients with glioblastoma and brain metastases and their caregivers in relation to their use of specialized palliative care	Europe	Glioblastoma	Longitudinal	Primary	2	Generic questionnaire	EORTC QLQ-C15-PAL	
Solanki et al., 2017 [419]	Impairments in Quality of Life and Cognitive Functions in Long-term Survivors of Glioblastoma	Asia	Glioblastoma	Cross-sectional	Primary	1	Generic questionnaire	WHOQOL-BREF	
Stahl et al., 2020 [420]	Health-related quality of life and emotional well-being in patients with glioblastoma and their relatives	Europe	Glioblastoma	Cohort	Primary	1	Generic questionnaire	SF-36	
Stahl et al., 2022 [421]	Quality of life in patients with glioblastoma and their relatives	Europe	Glioblastoma	Longitudinal	Primary	6	Generic questionnaire	SF-36	
Stockelmaier et al., 2017[422]	Therapy for recurrent high-grade gliomas: Results of a prospective multicenter study on health-related quality of life	Europe	High-grade glioma: not specified	Cross-sectional	Primary	1	Generic and tumour-specific questionnaire	EORTC QLQ-C30	EORTC-BN20
Suen et al., 2021 [423]	Health-related quality of life of glioblastoma patients receiving post-operative concomitant chemoradiotherapy plus adjuvant chemotherapy: A longitudinal study	Europe; Asia	Glioblastoma	Longitudinal	Primary	4	Generic and tumour-specific questionnaire	EORTC QLQ-C30	EORTC-BN20
Maitre et al., 2020 [424]	Prospective Longitudinal Assessment of Quality of Life and Activities of Daily Living as Patient-Reported Outcome Measures in Recurrent/Progressive Glioma Treated with High-dose Salvage Re-irradiation	Asia	High-grade glioma: 4 subtypes	Longitudinal	Primary	9	Generic and tumour-specific questionnaire	EORTC QLQ-C30	EORTC-BN20
Minniti et al., 2013 [425]	Health-Related Quality of Life in Elderly Patients With Newly Diagnosed Glioblastoma Treated With Short-Course Radiation Therapy Plus Concomitant and Adjuvant Temozolomide	Europe	Glioblastoma	Longitudinal	Primary	4	Generic and tumour-specific questionnaire	EORTC QLQ-C30	EORTC-BN20
Randazzo et al., 2019 [426]	Complementary and integrative health interventions and their association with health-related quality of life in the primary brain tumor population	North America	High grade; low grade gliomas: not specified	Cross-sectional	Primary	1	Generic and tumour-specific questionnaire	FACT-G	FACT-Br
Reddy et al., 2013 [427]	Prospective evaluation of health-related quality of life in patients with glioblastoma multiforme treated on a phase II trial of hypofractionated IMRT with temozolomide	North America	Glioblastoma	RCT	Primary	5	Generic and tumour-specific questionnaire	EORTC QLQ-C30	EORTC-BN20
Reijneveld et al., 2016 [428]	Health-related quality of life in patients with high-risk low-grade glioma (EORTC 22033-26033): a randomised, open-label, phase 3 intergroup study	Europe; North America; Oceania	Low-grade glioma: 3 subtypes	RCT	Primary	13	Generic and tumour-specific questionnaire	EORTC QLQ-C30	EORTC-BN20
Renovanz et al., 2018 [429]	Supportive Care Needs in Glioma Patients and Their Caregivers in Clinical Practice: Results of a Multicenter Cross-Sectional Study	Europe	High grade; low grade gliomas: not specified	Cross sectional	Primary	1	Generic and tumour-specific questionnaire	EORTC QLQ-C30, PQQ	EORTC-BN20
Renovanz et al., 2020 [430]	Health-related quality of life and distress in elderly vs. younger patients with high-grade glioma—results of a multicenter study	Europe	High-grade glioma:1 subtype	Cross-sectional	Primary	1	Generic and tumour-specific questionnaire	EORTC QLQ-C30	EORTC-BN20

Rubin et al., 2022 [431]	Primary versus recurrent surgery for glioblastoma—a prospective cohort study	Europe	Glioblastoma	Cohort	Primary	2	Generic questionnaire	EQ-5D-3L	
Taphoorn et al., 2015 [432]	Health-Related Quality of Life in a Randomized Phase III Study of Bevacizumab, Temozolomide, and Radiotherapy in Newly Diagnosed Glioblastoma	Europe	Glioblastoma	RCT	Secondary	17	Generic and tumour-specific questionnaire	EORTC QLQ-C30	EORTC-BN20
Taphoorn et al., 2018 [433]	Influence of Treatment With Tumor-Treating Fields on Health-Related Quality of Life of Patients With Newly Diagnosed Glioblastoma	Europe	Glioblastoma	RCT	Secondary	5	Generic and tumour-specific questionnaire	EORTC QLQ-C30	EORTC-BN20
Teng et al., 2021 [434]	Life after surgical resection of a low-grade glioma: A prospective cross sectional study evaluating health-related quality of life	Oceania	Low-grade glioma: 2 subtypes	Cross-sectional	Primary	11	Generic questionnaire	EORTC QLQ-C30	
Vaitkiene et al., 2019 [435]	Association of miR-34a Expression with Quality of life of Glioblastoma Patients: A Prospective Study	Europe	Glioblastoma	Cross-sectional	Primary	1	Generic and tumour-specific questionnaire	EORTC QLQ-C30, PHQ-9	EORTC-BN20
Valiulyte et al., 2022 [436]	Associations of miR-181a with Health-Related Quality of Life, Cognitive Functioning, and Clinical Data of Patients with Different Grade Glioma Tumors	Europe	High grade; low grade gliomas: 1 subtype	Cross-sectional	Primary	1	Generic and tumour-specific questionnaire	EORTC QLQ-C30, PHQ-9	EORTC-BN20
Jakola et al., 2011 [437]	Postoperative Deterioration in Health Related Quality of Life as Predictor for Survival in Patients with Glioblastoma: A Prospective Study	Europe	Glioblastoma	Longitudinal	Primary	2	Generic questionnaire	EQ-5D-5L	
Jakola et al., 2012 [438]	Low Grade Gliomas in Eloquent Locations – Implications for Surgical Strategy, Survival and Long Term Quality of Life	Europe	Low-grade glioma: 3 subtypes	Cohort	Secondary	1	Generic and tumour-specific questionnaire	EORTC QLQ-C30, EQ-5D	EORTC-BN20
Jakola et al., 2014 [439]	Surgical strategies in low-grade gliomas and implications for long-term quality of life	Europe	Low grade glioma: 3 subtypes	Cohort	Primary	1	Generic and tumour-specific questionnaire	EORTC QLQ-C30, EQ-5D	EORTC-BN20
Jakola et al., 2022 [440]	The impact of resection in IDH-mutant WHO grade 2 gliomas: a retrospective population-based parallel cohort study	Europe	Low grade glioma: not specified	Cohort	Secondary	1	Generic and tumour-specific questionnaire	EORTC QLQ-C30, EQ-5D	EORTC-BN20
Nickel et al., 2018 [441]	The patients' view: impact of the extent of resection, intraoperative imaging, and awake surgery on health-related quality of life in high-grade glioma patients—results of a multicenter cross-sectional study	Europe	High grade glioma: 4 subtypes	Cross-sectional	Primary	1	Generic and tumour-specific questionnaire	EORTC QLQ-C30	EORTC-BN20
Noll et al., 2017 [442]	Relationships between neurocognitive functioning, mood, and quality of life in patients with temporal lobe glioma	North America	High grade glioma: 3 subtypes	Cross-sectional	Primary	1	Generic and tumour-specific questionnaire	FACT-G	FACT-Br
Okita et al., 2015 [443]	Health-related quality of life in long-term survivors with Grade II gliomas: the contribution of disease recurrence and Karnofsky Performance Status	Asia	Low grade Glioma: 3 subtypes	Cross sectional	Primary	1	Generic and tumour-specific questionnaire	EORTC QLQ-C30	EORTC-BN20

Aaronson et al., 2011 [444]	Compromised Health-Related Quality of Life in Patients with low-grade glioma	Europe	Low grade glioma: not specified	Cross sectional	Primary	1	Generic and tumour-specific questionnaire	SF-36	EORTC-BN20
Ahn et al., 2022 [445]	Influence of Concurrent and Adjuvant Temozolomide on Health-Related Quality of Life of Patients with Grade III Gliomas: A Secondary Analysis of a Randomized Clinical Trial (KNOG-1101 Study)	Asia	High grade glioma: 1 subtype	RCT	Primary	5	Generic and tumour-specific questionnaire	EORTC QLQ-C30	EORTC-BN20
Yavas et al., 2012 [446]	Prospective assessment of health-related quality of life in patients with low-grade glioma	Asia	Low grade glioma: not specified	Non-RCT	Primary	8	Generic and tumour-specific questionnaire	EORTC QLQ-C30	EORTC-BN20
Armstrong et al., 2013 [447]	Net Clinical Benefit Analysis of Radiation Therapy Oncology Group 0525: A Phase III Trial Comparing Conventional Adjuvant Temozolomide With Dose- Intensive Temozolomide in Patients With Newly Diagnosed Glioblastoma	North America; Europe	Glioblastoma	RTC	Primary	9	Generic and tumour-specific questionnaire	EORTC QLQ-C30	EORTC-BN20, MDASI-BT
Zhu et al., 2017 [448]	Health-related quality of life, cognitive screening, and functional status in a randomized phase III trial (EF-14) of tumor treating fields with temozolomide compared to temozolomide alone in newly diagnosed glioblastoma	North America; Europe	Glioblastoma	RCT	Secondary	4	Generic and tumour-specific questionnaire	EORTC QLQ-C30	EORTC-BN20
Wick et al., 2019 [449]	Longitudinal analysis of quality of life following treatment with Asunercept plus reirradiation versus reirradiation in progressive glioblastoma patients	Europe	Glioblastoma	Longitudinal	Primary	2	Generic and tumour-specific questionnaire	EORTC QLQ-C15-PAL	EORTC-BN20
Gabel et al., 2019 [450]	Health Related Quality of Life in Adult Low and High-Grade Glioma Patients Using the National Institutes of Health Patient Reported Outcomes Measurement Information System (PROMIS) and Neuro-QOL Assessments	North America	High grade and low grade glioma: not specified	Mixed-method	Primary	1	Interview + generic and tumour-specific questionnaire	PROMIS	Neuro-QOL
Giovagnoli et al., 2014 [451]	Quality of life and brain tumors: what beyond the clinical burden?	Europe	High grade and low grade glioma: 5 subtypes	Cross-sectional	Primary	1	Generic questionnaire	FLIC	
Palmer et al., 2021 [452]	Health-Related Quality of Life for Patients Receiving Tumor Treating Fields for Glioblastoma	North America; Asia	Glioblastoma	Cross sectional	Primary	1	Generic questionnaire	EQ-5D-5L	
Park et al., 2022 [453]	Quality of life following concurrent temozolomide-based chemoradiation therapy or observation in low-grade glioma	North America	Low grade glioma: not specified	Cross-sectional	Primary	1	Generic questionnaire	EQ-5D-5L	
Peters et al., 2014 [454]	Impact of Health-Related Quality of Life and Fatigue on Survival of Recurrent High-Grade Glioma Patients	North America	High grade Glioma: not specified	Observational	Primary	1	Generic and tumour-specific questionnaire	FACT-G	FACT-Br

Peters et al., 2022 [455]	Effects of low-dose naltrexone on quality of life in high-grade glioma patients: a placebo-controlled, double-blind randomized trial	North America	High grade glioma: not specified	RTC	Primary	1	Tumour-specific questionnaire		FACT-Br
Piil et al., 2015 [456]	Health-related quality of life in patients with high-grade gliomas: a quantitative longitudinal study	Europe	High grade glioma: 7 subtypes	Longitudinal	Primary	5	Generic and tumour-specific questionnaire	FACT-G	FACT-Br
Piil et al., 2019[457]	Health-related quality of life and caregiver perspectives in glioblastoma survivors: a mixed methods study	Europe	Glioblastoma	Mixed method	Primary	1	Interview + generic and tumour-specific questionnaire	FACT-G	FACT-Br
Pollom et al., 2017 [458]	Phase 1/2 Trial of 5-Fraction Stereotactic Radiosurgery With 5-mm Margins With Concurrent and Adjuvant Temozolomide in Newly Diagnosed Supratentorial Glioblastoma: Health-Related Quality of Life Results	North America	Glioblastoma	Longitudinal	Secondary	8	Generic and tumour-specific questionnaire	EORTC QLQ-C30	EORTC-BN20, MDASI-BT
Porter et al., 2015 [459]	Assessment of clinical and nonclinical characteristics associated with health-related quality of life in patients with high-grade gliomas: a feasibility study	North America	High grade glioma: 5 subtypes	Cross sectional	Primary	1	Generic and tumour-specific questionnaire	FPQLI-C	FACT-Br
Clement et al., 2021 [460]	Impact of depatuxizumab mafodotin on health-related quality of life and neurological functioning in the phase II EORTC 1410/INTELLANCE 2 trial for EGFR-amplified recurrent glioblastoma	Europe	Glioblastoma	RCT	Primary	4	Generic and tumour-specific questionnaire	EORTC QLQ-C30	EORTC-BN20
Coomans et al., 2022 [461]	Factors associated with health-related quality of life (HRQoL) deterioration in glioma patients during the progression-free survival period	Europe	Glioblastoma	Longitudinal	Primary	4	Generic and tumour-specific questionnaire	EORTC QLQ-C30	EORTC-BN20
Kaminska et al., 2017 [462]	Quality of life in patients with brain tumors in the course of alpha therapy	Europe	Glioblastoma	Cross-sectional	Primary	1	Generic questionnaire	WHOQOL-BREF	

* RCT: Randomized Controlled Trial; Non-RCT: Nonrandomized controlled trial

[a] Generic questionnaire: EORTC QLQ-C30: European Organization for Research and Treatment for Cancer Quality of Life Questionnaire-Core30; SF-36: Medical Outcome Study 36-item Short Form Health Survey; EQ-5D-5L: EUroQoL-5 Dimensions 5-Level Version; EQ-5D-3L: EUroQoL-5 Dimensions 3-level version; QPP: Quality from the Patient's Perspective questionnaire; RAND-36: RAND 36-Item Health Survey; EORTC QLQ-C15-PAL: European Organization for Research and Treatment for Cancer Quality of Life Questionnaire Core 15 Palliative Care; WHOQOL-BREF: The World Health Organization Quality of Life Questionnaire; FACT-G: Functional Assessment of Cancer Therapy-General; PPQ: Patient's Perspective Questionnaire; PHQ-9: Patient Health Questionnaire-9; PROMIS: Patient-Reported Outcome Measurement Information System; FLIC: Functional Living Index Cancer; FPQLI-C: The Ferrans and Powers Quality of Life Index Cancer version III

[b] Tumour/domain-specific questionnaire: EORTC QLQ-BN20: European Organization for Research and Treatment for Cancer Quality of Life Questionnaire Brain module; SNAS: Sherbrooke Neuro-Oncology Assessment Scale; ADL: Katz Activities of Daily Living; SCNS-SF34: 34-Item Short-Form Supportive Care Need Survey; FACT-Br: Functional Assessment of Cancer Therapy-Brain; Neuro-QOL: Quality of Life in Neurological Disorders; MDASI-BT: MD Anderson Symptom Inventory Brain Tumor

1. Bonvalot, S., et al., *Final Safety and Health-Related Quality of LIfe Results of the Phase 2/3 Act.In.Sarc Study With Preoperative NBTXR3 Plus Radiation Therapy Versus Radiation Therapy in Locally Advanced Soft-Tissue Sarcoma.* Int J Radiat Oncol Biol Phys, 2022. **114**(3): p. 422-432.
2. Ustundag, S. and A.D. Zencirci, *Factors affecting the quality of life of cancer patients undergoing chemotherapy: A questionnaire study.* Asia Pac J Oncol Nurs, 2015. **2**(1): p. 17-25.
3. Davidson, D., et al., *Health-related quality of life following treatment for extremity soft tissue sarcoma.* J Surg Oncol, 2016. **114**(7): p. 821-827.
4. Day, J.R., et al., *Patient reported quality of life in young adults with sarcoma receiving care at a sarcoma center.* Front Psychol, 2022. **13**: p. 871254.
5. den Hollander, D., et al., *Symptoms reported by gastrointestinal stromal tumour (GIST) patients on imatinib treatment: combining questionnaire and forum data.* Support Care Cancer, 2022. **30**(6): p. 5137-5146.
6. den Hollander, D., et al., *'I thought I had fibroids, and now I don't': a mixed method study on health-related quality of life in uterine sarcoma patients.* Health Qual Life Outcomes, 2022. **20**(1): p. 65.
7. Dong, S., et al., *Quality of life and Q-TWiST were not adversely affected in Ewing sarcoma patients treated with combined anlotinib, irinotecan, and vincristine: (Peking University People's Hospital Ewing sarcoma trial-02, PKUPH-EWS-02).* Medicine, 2021. **100**(51): p. e28078.
8. Drabbe, C., et al., *The age-related impact of surviving sarcoma on health-related quality of life: data from the SURVSARC study.* ESMO Open, 2021. **6**(1): p. 100047.
9. Dressler, J.A., et al., *Long-term functional outcomes of laparoscopic resection for gastric gastrointestinal stromal tumors.* Surg Endosc, 2016. **30**(4): p. 1592-8.
10. Fauske, L., et al., *Striving towards Normality in Daily Life: A Qualitative Study of Patients Living with Metastatic Gastrointestinal Stromal Tumour in Long-Term Clinical Remission.* Sarcoma, 2020. **2020**: p. 1814394.
11. Ferguson, R.J., et al., *Self-reported cognitive impairments and quality of life in patients with gastrointestinal stromal tumor: Results of a multinational survey.* Cancer, 2022. **128**(22): p. 4017-4026.
12. Fiore, M., et al., *A Prospective Observational Study of Multivisceral Resection for Retroperitoneal Sarcoma: Clinical and Patient-Reported Outcomes 1 Year After Surgery.* Ann Surg Oncol, 2021. **28**(7): p. 3904-3916.
13. Furtado, S., et al., *Physical functioning, pain and quality of life after amputation for musculoskeletal tumours: a national survey.* Bone Joint J, 2015. **97-B**(9): p. 1284-90.
14. Yoo, C., et al., *Impact of imatinib rechallenge on health-related quality of life in patients with TKI-refractory gastrointestinal stromal tumours: Sub-analysis of the placebo-controlled, randomised phase III trial (RIGHT).* Eur J Cancer, 2016. **52**: p. 201-8.
15. Younger, E., et al., *Health-Related Quality of Life and Experiences of Sarcoma Patients during the COVID-19 Pandemic.* Cancers (Basel), 2020. **12**(8).
16. Lazenby, M. and J. Khatib, *Associations among Patient Characteristics, Health-Related Quality of Life, and Spiritual Well-Being among Arab Muslim Cancer Patients.* Journal of Palliative Medicine, 2012. **15**(12): p. 1321-1324.
17. Le Cesne, A., et al., *A randomized phase III trial comparing trabectedin to best supportive care in patients with pre-treated soft tissue sarcoma: T-SAR, a French Sarcoma Group trial.* Ann Oncol, 2021. **32**(8): p. 1034-1044.
18. Lim, H.J., et al., *Retrospective quality of life study in patients with retroperitoneal sarcoma in an Asian population.* Health Qual Life Outcomes, 2020. **18**(1): p. 270.
19. Lopez-Guerra, J.L., et al., *Health related quality of life and late side effects of long-term survivors of Ewing's sarcoma of bone.* J BUON, 2011. **16**(3): p. 528-36.
20. Weidema, M.E., et al., *Health-related quality of life and symptom burden of epithelioid hemangioendothelioma patients: a global patient-driven Facebook study in a very rare malignancy.* Acta Oncol, 2020. **59**(8): p. 975-982.
21. Weschenfelder, W., et al., *Factors influencing quality of life, function, reintegration and participation after musculoskeletal tumour operations.* BMC Cancer, 2020. **20**(1): p. 351.
22. Wong, P., et al., *Long-Term Quality of Life of Retroperitoneal Sarcoma Patients Treated with Pre-Operative Radiotherapy and Surgery.* Cureus, 2017. **9**(10): p. e1764.
23. Eliason, L., et al., *Qualitative study to characterize patient experience and relevance of patient-reported outcome measures for patients with metastatic synovial sarcoma.* J Patient Rep Outcomes, 2022. **6**(1): p. 43.
24. Eichler, M., et al., *The association of Health-Related Quality of Life and 1-year-survival in sarcoma patients-results of a Nationwide Observational Study (PROSa).* Br J Cancer, 2022. **126**(9): p. 1346-1354.
25. Eichler, M., et al., *The Health-Related Quality of Life of Sarcoma Patients and Survivors in Germany-Cross-Sectional Results of a Nationwide Observational Study (PROSa).* Cancers (Basel), 2020. **12**(12).
26. Eichler, M., et al., *Quality of life of GIST patients with and without current tyrosine kinase inhibitor treatment: Cross-sectional results of a German multicentre observational study (PROSa).* Eur J Cancer Care (Engl), 2021. **30**(6): p. e13484.
27. Edmondson, R.J., et al., *Phase 2 study of anastrozole in rare cohorts of patients with estrogen receptor/progesterone receptor positive leiomyosarcomas and carcinosarcomas of the uterine corpus: The PARAGON trial (ANZGOG 0903).* Gynecol Oncol, 2021. **163**(3): p. 524-530.
28. Hentschel, L., et al., *Quality of life and added value of a tailored palliative care intervention in patients with soft tissue sarcoma undergoing treatment with trabectedin: a multicentre, cluster-randomised trial within the German Interdisciplinary Sarcoma Group (GISG).* BMJ Open, 2020. **10**(8): p. e035546.
29. Holzer, L.A., et al., *Body image, self-esteem, and quality of life in patients with primary malignant bone tumors.* Arch Orthop Trauma Surg, 2020. **140**(1): p. 1-10.
30. Hudgens, S., et al., *Evaluation of Quality of Life at Progression in Patients with Soft Tissue Sarcoma.* Sarcoma, 2017. **2017**: p. 2372135.
31. Sachsenmaier, S.M., I. Ipach, and T. Kluba, *Quality of Life, Physical and Mental Status and Contentment of Patients with Localized Soft Tissue or Bone Sarcoma: A Questionnaire Analysis.* Orthop Rev (Pavia), 2015. **7**(2): p. 5920.
32. Saebye, C., et al., *Factors associated with reduced functional outcome and quality of life in patients having limb-sparing surgery for soft tissue sarcomas - a national multicenter study of 128 patients.* Acta Oncol, 2017. **56**(2): p. 239-244.
33. Saebye, C., et al., *Changes in Functional Outcome and Quality of Life in Soft Tissue Sarcoma Patients within the First Year after Surgery: A Prospective Observational Study.* Cancers (Basel), 2020. **12**(2).

34. Schoffski, P., et al., *Patient-reported outcomes in individuals with advanced gastrointestinal stromal tumor treated with ripretinib in the fourth-line setting: analysis from the phase 3 INVICTUS trial.* BMC Cancer, 2022. **22**(1): p. 1302.
35. Silva, R.S., et al., *Quality of Life in Adults with Sarcomas under Conservative Surgery or Amputation.* Acta Ortop Bras, 2020. **28**(5): p. 236-242.
36. Soomers, V., et al., *The Perceived Impact of Length of the Diagnostic Pathway Is Associated with Health-Related Quality of Life of Sarcoma Survivors: Results from the Dutch Nationwide SURVSARC Study.* Cancers (Basel), 2020. **12**(8).
37. Srivastava, A., et al., *Quality of life in patients with chordomas/chondrosarcomas during treatment with proton beam therapy.* J Radiat Res, 2013. **54 Suppl 1**(Suppl 1): p. i43-8.
38. Maggi, G., et al., *Sarcoma patients' quality of life from diagnosis to yearly follow-up: experience from an Italian tertiary care center.* Future Oncol, 2019. **15**(27): p. 3125-3134.
39. Maggi, G., et al., *Symptoms and Their Implications on Quality of Life and Psychological Distress in Sarcoma Patients.* Future Oncology, 2021. **17**(7): p. 817-823.
40. Malek, F., et al., *Does Limb-salvage Surgery Offer Patients Better Quality of Life and Functional Capacity than Amputation?* Clinical Orthopaedics and Related Research®, 2012. **470**(7): p. 2000-2006.
41. Mason, G.E., et al., *Quality of life following amputation or limb preservation in patients with lower extremity bone sarcoma.* Front Oncol, 2013. **3**: p. 210.
42. Reichardt, P., et al., *Quality of Life and Utility in Patients with Metastatic Soft Tissue and Bone Sarcoma: The Sarcoma Treatment and Burden of Illness in North America and Europe (SABINE) Study.* Sarcoma, 2012. **2012**: p. 740279.
43. Reijers, S.J.M., et al., *Health-related quality of life after isolated limb perfusion compared to extended resection, or amputation for locally advanced extremity sarcoma: Is a limb salvage strategy worth the effort?* Eur J Surg Oncol, 2022. **48**(3): p. 500-507.
44. Rivard, J.D., et al., *Quality of life, functional outcomes, and wound complications in patients with soft tissue sarcomas treated with preoperative chemoradiation: a prospective study.* Ann Surg Oncol, 2015. **22**(9): p. 2869-75.
45. Vade, L., I. Hompland, and L. Fauske, *Exploring Mentorship as a Novel Approach to Improving Quality of Life in Sarcoma Survivors: A Qualitative Pilot Study.* Sarcoma, 2021. **2021**: p. 2042785.
46. van Eck, I., et al., *Unraveling the Heterogeneity of Sarcoma Survivors' Health-Related Quality of Life Regarding Primary Sarcoma Location: Results from the SURVSARC Study.* Cancers (Basel), 2020. **12**(11).
47. van Eck, I., et al., *Health-Related Quality of Life Issues Experienced by Thoracic and Breast Sarcoma Patients: A Rare and Understudied Group.* J Clin Med, 2021. **10**(22).
48. Van Tine, B.A., et al., *Quality of life of patients with soft tissue sarcoma treated with doxorubicin in the ANNOUNCE phase III clinical trial.* Rare Tumors, 2022. **14**: p. 20363613221100033.
49. Jing, S., et al., *Efficacy of Neoadjuvant Chemotherapy plus Limb-Sparing Surgery for Osteosarcoma and Its Impact on Long-Term Quality of Life.* Evid Based Complement Alternat Med, 2022. **2022**: p. 1693824.
50. Obermair, A., et al., *Prospective, non-randomized phase 2 clinical trial of carboplatin plus paclitaxel with sequential radical pelvic radiotherapy for uterine papillary serous carcinoma.* Gynecol Oncol, 2011. **120**(2): p. 179-84.
51. Ogura, K., et al., *The critical difference in the DASH (Disabilities of the Arm, Shoulder, and Hand) outcome measure after essential upper extremity tumor surgery.* J Shoulder Elbow Surg, 2021. **30**(9): p. e602-e609.
52. Ostacoli, L., et al., *Quality of Life, Anxiety and Depression in Soft Tissue Sarcomas as Compared to More Common Tumours: An Observational Study.* Applied Research in Quality of Life, 2014. **9**(1): p. 123-131.
53. Zhuang, A., et al., *Does Aggressive Surgery Mean Worse Quality of Life and Functional Capacity in Retroperitoneal Sarcoma Patients?—A Retrospective Study of 161 Patients from China.* Cancers, 2022. **14**(20): p. 5126.
54. Gotzl, R., et al., *Patient's quality of life after surgery and radiotherapy for extremity soft tissue sarcoma - a retrospective single-center study over ten years.* Health Qual Life Outcomes, 2019. **17**(1): p. 170.
55. Gough, N., et al., *Does palliative chemotherapy really palliate and are we measuring it correctly? A mixed methods longitudinal study of health related quality of life in advanced soft tissue sarcoma.* PLOS ONE, 2019. **14**(9): p. e0210731.
56. Gounder, M., et al., *Health-related quality of life and pain with selinexor in patients with advanced dedifferentiated liposarcoma.* Future Oncol, 2021. **17**(22): p. 2923-2939.
57. Paredes, T., et al., *Quality of life of sarcoma patients from diagnosis to treatments: predictors and longitudinal trajectories.* Eur J Oncol Nurs, 2011. **15**(5): p. 492-9.
58. Paredes, T.F., M.C. Canavarro, and M.R. Simoes, *Social support and adjustment in patients with sarcoma: the moderator effect of the disease phase.* J Psychosoc Oncol, 2012. **30**(4): p. 402-25.
59. Poole, C.D., et al., *Health utility of patients with advanced gastrointestinal stromal tumors (GIST) after failure of imatinib and sunitinib: findings from GRID, a randomized, double-blind, placebo-controlled phase III study of regorafenib versus placebo.* Gastric Cancer, 2015. **18**(3): p. 627-34.
60. Carbajal-Lopez, E.B., et al., *Psychological Distress, Fatigue and Quality of Life in Patients with Gastrointestinal Stromal Tumors.* Psychol Russ, 2022. **15**(2): p. 3-13.
61. Chuah, P.L., et al., *Assessment of Adherence to Imatinib and Health-Related Quality of Life Among Patients with Gastrointestinal Stromal Tumor: A Cross-Sectional Study in an Oncology Clinic in Malaysia.* Patient Prefer Adherence, 2021. **15**: p. 2175-2184.
62. Coens, C., et al., *Health-related quality-of-life results from PALETTE: A randomized, double-blind, phase 3 trial of pazopanib versus placebo in patients with soft tissue sarcoma whose disease has progressed during or after prior chemotherapy-a European Organization for research and treatment of cancer soft tissue and bone sarcoma group global network study (EORTC 62072).* Cancer, 2015. **121**(17): p. 2933-41.
63. Custers, J.A., et al., *Fear of progression in patients with gastrointestinal stromal tumors (GIST): Is extended lifetime related to the Sword of Damocles?* Acta Oncol, 2015. **54**(8): p. 1202-8.
64. Kask, G., et al., *Soft Tissue Sarcoma of Lower Extremity: Functional Outcome and Quality of Life.* Ann Surg Oncol, 2021. **28**(11): p. 6892-6905.
65. Kokkali, S., et al., *A Multicenter, Prospective, Observational Study to Assess the Clinical Activity and Impact on Symptom Burden and Patients' Quality of Life in Patients with Advanced Soft Tissue Sarcomas Treated with Trabectedin in a Real-World Setting in Greece.* Cancers (Basel), 2022. **14**(8).

66. Barry, A.S., et al., *The Impact of Disease Progression on Health-Related Quality of Life Outcomes in Patients With Oligometastatic Disease at 12 Months Post Stereotactic Body Radiation Therapy.* Int J Radiat Oncol Biol Phys, 2022. **114**(5): p. 989-999.
67. Ferracini, A.C., et al., *Physical and functional well-being and symptoms of ovarian cancer in women undergoing first-line of chemotherapy: a one-year follow-up.* Support Care Cancer, 2021. **29**(12): p. 7421-7430.
68. Forner, D.M., R. Dakhil, and B. Lampe, *Can clitoris-conserving surgery for early vulvar cancer improve the outcome in terms of quality of life and sexual sensation?* Eur J Obstet Gynecol Reprod Biol, 2013. **171**(1): p. 150-3.
69. Forner, D.M., R. Dakhil, and B. Lampe, *Quality of life and sexual function after surgery in early stage vulvar cancer.* Eur J Surg Oncol, 2015. **41**(1): p. 40-5.
70. van Dongen, J., et al., *Challenges and controversies patients and (health care) professionals experience in managing vaginal, vulvar, penile or anal cancer: The SILENCE study.* Eur J Cancer Care (Engl), 2022. **31**(6): p. e13676.
71. Tsubamoto, H., et al., *Effects of leuprorelin for the treatment of recurrent gynecological cancer by assessment including self-administered quality-of-life questionnaire.* J Obstet Gynaecol Res, 2019. **45**(1): p. 203-209.
72. Zaid, T., et al., *Use of social media to conduct a cross-sectional epidemiologic and quality of life survey of patients with neuroendocrine carcinoma of the cervix: a feasibility study.* Gynecol Oncol, 2014. **132**(1): p. 149-53.
73. Licht, T., et al., *Evaluation by electronic patient-reported outcomes of cancer survivors' needs and the efficacy of inpatient cancer rehabilitation in different tumor entities.* Support Care Cancer, 2021. **29**(10): p. 5853-5864.
74. Hartung, T.J., et al., *Age-related variation and predictors of long-term quality of life in germ cell tumor survivors.* Urol Oncol, 2016. **34**(2): p. 60 e1-6.
75. Hojer, E.G., et al., *Effect of Testosterone Replacement Therapy on Quality of Life and Sexual Function in Testicular Cancer Survivors With Mild Leydig Cell Insufficiency: Results From a Randomized Double-blind Trial.* Clin Genitourin Cancer, 2022. **20**(4): p. 334-343.
76. Saoud, R.M., et al., *Impact of Non-guideline-directed Care on Quality of Life in Testicular Cancer Survivors.* Eur Urol Focus, 2021. **7**(5): p. 1137-1142.
77. Schmidt, A.H., et al., *Limited post-chemotherapy retroperitoneal resection of residual tumour in non-seminomatous testicular cancer: complications, outcome and quality of life.* Acta Oncol, 2018. **57**(8): p. 1084-1093.
78. Skott, J.W., et al., *Quality of Life in Long-Term Testicular Cancer Survivors With Compensated Leydig Cell Dysfunction.* Clin Genitourin Cancer, 2019. **17**(1): p. e65-e71.
79. Jovanovski, A., et al., *Quality of life among germ-cell testicular cancer survivors: The effect of time since cancer diagnosis.* PLoS One, 2021. **16**(10): p. e0258257.
80. Nezu, K., et al., *Association of financial toxicity with quality of life in testicular cancer survivors.* Int J Urol, 2022. **29**(12): p. 1526-1534.
81. Nicolai, N., et al., *Quality of life and pain control following laparoscopic retroperitoneal lymph node dissection in early-stage nonseminoma.* Tumori, 2015. **101**(6): p. 650-6.
82. Witty, K., et al., *The impact of surgical treatment for penile cancer -- patients' perspectives.* Eur J Oncol Nurs, 2013. **17**(5): p. 661-7.
83. Perez, J., et al., *Oncological and Functional Outcomes After Organ-Sparing Plastic Reconstructive Surgery for Penile Cancer.* Urology, 2020. **142**: p. 161-165 e1.
84. Chavarriaga, J., et al., *Inverted urethral flap reconstruction after partial penectomy: Long-term oncological and functional outcomes.* Urol Oncol, 2022. **40**(4): p. 169 e13-169 e20.
85. Khanal, N., et al., *The effects of hypogonadism on quality of life in survivors of germ cell tumors treated with surgery alone versus surgery plus platinum-based chemotherapy.* Support Care Cancer, 2020. **28**(7): p. 3165-3170.
86. Flechtner, H.H., et al., *Quality-of-Life Analysis of the German Prospective Multicentre Trial of Single-cycle Adjuvant BEP Versus Retroperitoneal Lymph Node Dissection in Clinical Stage I Nonseminomatous Germ Cell Tumours.* Eur Urol, 2016. **69**(3): p. 518-25.
87. Ballal, S., et al., *Broadening horizons with (225)Ac-DOTATATE targeted alpha therapy for gastroenteropancreatic neuroendocrine tumour patients stable or refractory to (177)Lu-DOTATATE PRRT: first clinical experience on the efficacy and safety.* Eur J Nucl Med Mol Imaging, 2020. **47**(4): p. 934-946.
88. Beesley, V.L., et al., *Perceptions of care and patient-reported outcomes in people living with neuroendocrine tumours.* Support Care Cancer, 2018. **26**(9): p. 3153-3161.
89. Begum, N., et al., *Anxiety, Depression and Quality of Life in Patients with Neuroendocrine Neoplasia After Surgery.* World J Surg, 2022. **46**(6): p. 1408-1419.
90. Laing, E., et al., *Prevalence of malnutrition and nutrition-related complications in patients with gastroenteropancreatic neuroendocrine tumours.* J Neuroendocrinol, 2022. **34**(6): p. e13116.
91. Lamarca, A., et al., *Somatostatin analogue-induced pancreatic exocrine insufficiency in patients with neuroendocrine tumors: results of a prospective observational study.* Expert Rev Gastroenterol Hepatol, 2018. **12**(7): p. 723-731.
92. Lewis, A.R., et al., *Health-related quality of life, anxiety, depression and impulsivity in patients with advanced gastroenteropancreatic neuroendocrine tumours.* World J Gastroenterol, 2018. **24**(6): p. 671-679.
93. Hummelshoj, N.E., et al., *Fatigue and quality of life in patients with neuroendocrine neoplasia.* Scand J Gastroenterol, 2023. **58**(1): p. 45-53.
94. Scandurra, C., et al., *Quality of Life in Patients with Neuroendocrine Neoplasms: The Role of Severity, Clinical Heterogeneity, and Resilience.* J Clin Endocrinol Metab, 2021. **106**(1): p. e316-e327.
95. Swinburn, P., et al., *Elicitation of health state utilities in neuroendocrine tumours.* J Med Econ, 2012. **15**(4): p. 681-7.
96. Martini, C., et al., *Quality of Life in Patients with Metastatic Gastroenteropancreatic Neuroendocrine Tumors Receiving Peptide Receptor Radionuclide Therapy: Information from a Monitoring Program in Clinical Routine.* J Nucl Med, 2018. **59**(10): p. 1566-1573.
97. Meng, Y., et al., *Patient-reported health state utilities in metastatic gastroenteropancreatic neuroendocrine tumours - an analysis based on the CLARINET study.* Health Qual Life Outcomes, 2017. **15**(1): p. 131.
98. Meyer, T., et al., *Capecitabine and streptozocin +/- cisplatin in advanced gastroenteropancreatic neuroendocrine tumours.* Eur J Cancer, 2014. **50**(5): p. 902-11.

99. Mitry, E., et al., *Bevacizumab plus capecitabine in patients with progressive advanced well-differentiated neuroendocrine tumors of the gastro-intestinal (GI-NETs) tract (BETTER trial)--a phase II non-randomised trial.* Eur J Cancer, 2014. **50**(18): p. 3107-15.
100. Modica, R., et al., *Health-related quality of life in patients with neuroendocrine neoplasms: a two-wave longitudinal study.* J Endocrinol Invest, 2022. **45**(11): p. 2193-2200.
101. van Leeuwaarde, R.S., et al., *A Multinational Pilot Study on Patients' Perceptions of Advanced Neuroendocrine Neoplasms on the EORTC QLQ-C30 and EORTC QLQ-GINET21 Questionnaires.* J Clin Med, 2022. **11**(5).
102. Ohlsson, H., et al., *Relationship between somatostatin receptor expressing tumour volume and health-related quality of life in patients with metastatic GEP-NET.* J Neuroendocrinol, 2022. **34**(6): p. e13139.
103. Adams, J.R., et al., *Living With Neuroendocrine Tumors: Assessment of Quality of Life Through a Mobile Application.* JCO Clin Cancer Inform, 2019. **3**: p. 1-10.
104. Zandee, W.T., et al., *Symptomatic and Radiological Response to 177Lu-DOTATATE for the Treatment of Functioning Pancreatic Neuroendocrine Tumors.* J Clin Endocrinol Metab, 2019. **104**(4): p. 1336-1344.
105. Pavel, M.E., et al., *Health-related quality of life for everolimus versus placebo in patients with advanced, non-functional, well-differentiated gastrointestinal or lung neuroendocrine tumours (RADIANT-4): a multicentre, randomised, double-blind, placebo-controlled, phase 3 trial.* Lancet Oncol, 2017. **18**(10): p. 1411-1422.
106. Khan, S., et al., *Quality of life in 265 patients with gastroenteropancreatic or bronchial neuroendocrine tumors treated with [177Lu-DOTA0,Tyr3]octreotate.* J Nucl Med, 2011. **52**(9): p. 1361-8.
107. Traub-Weidinger, T., et al., *Improved quality of life in patients treated with Peptide radionuclides.* World J Nucl Med, 2011. **10**(2): p. 115-21.
108. Bentzen, A.G., et al., *Impaired health-related quality of life after chemoradiotherapy for anal cancer: late effects in a national cohort of 128 survivors.* Acta Oncol, 2013. **52**(4): p. 736-44.
109. Bourdais, R., et al., *Pulse-dose-rate interstitial brachytherapy in anal squamous cell carcinoma: clinical outcomes and patients' health quality perception.* J Contemp Brachytherapy, 2021. **13**(3): p. 263-272.
110. Bridgewater, J., et al., *Quality of life, long-term survivors and long-term outcome from the ABC-02 study.* Br J Cancer, 2016. **114**(9): p. 965-71.
111. Goislard de Monsabert, C., et al., *Selective Internal Radiation Combined with Chemotherapy Maintains the Quality of Life in Intrahepatic Cholangiocarcinomas.* Curr Oncol, 2021. **28**(6): p. 4530-4541.
112. De, B., et al., *Patient-Reported Bowel and Urinary Function in Long-Term Survivors of Squamous Cell Carcinoma of the Anus Treated With Definitive Intensity Modulated Radiation Therapy And Concurrent Chemotherapy.* Int J Radiat Oncol Biol Phys, 2022. **114**(1): p. 78-88.
113. Elberg Dengso, K., et al., *Health-related quality of life and anxiety and depression in patients diagnosed with cholangiocarcinoma: a prospective cohort study.* Acta Oncol, 2017. **56**(2): p. 198-204.
114. Fakhrian, K., et al., *Chronic adverse events and quality of life after radiochemotherapy in anal cancer patients. A single institution experience and review of the literature.* Strahlenther Onkol, 2013. **189**(6): p. 486-94.
115. Lefevre, A.C., et al., *One-Year Treatment-Related Side Effects and Quality of Life After Chemoradiotherapy in Squamous Cell Carcinoma of the Anus.* Int J Radiat Oncol Biol Phys, 2023. **115**(5): p. 1165-1177.
116. Liu, S., L. Zhou, and L. An, *Implementation of comprehensive rehabilitation therapy in postoperative care of patients with cholangiocarcinoma and its impact on patients' quality of life.* Exp Ther Med, 2019. **17**(4): p. 2703-2707.
117. Liu, S., et al., *Target nursing care on anxiety and depression in patients with gallbladder cancer during perioperative period.* Medicine (Baltimore), 2022. **101**(31): p. e29883.
118. Welzel, G., et al., *Quality of life outcomes in patients with anal cancer after combined radiochemotherapy.* Strahlenther Onkol, 2011. **187**(3): p. 175-82.
119. Woradet, S., et al., *Factors Affecting Health-Related Quality of Life in Patients With Cholangiocarcinoma in the Northeastern Region of Thailand.* Cancer Nurs, 2015. **38**(6): p. E46-51.
120. Woradet, S., et al., *Health-Related Quality of Life and Survival of Cholangiocarcinoma Patients in Northeastern Region of Thailand.* PLoS One, 2016. **11**(9): p. e0163448.
121. Wu, T.T., et al., *Percutaneous Intraductal Radiofrequency Ablation for Extrahepatic Distal Cholangiocarcinoma: A Method for Prolonging Stent Patency and Achieving Better Functional Status and Quality of Life.* Cardiovasc Intervent Radiol, 2017. **40**(2): p. 260-269.
122. Edeline, J., et al., *Gemcitabine and Oxaliplatin Chemotherapy or Surveillance in Resected Biliary Tract Cancer (PRODIGE 12-ACCORD 18-UNICANCER GI): A Randomized Phase III Study.* J Clin Oncol, 2019. **37**(8): p. 658-667.
123. Han, K., et al., *Prospective evaluation of acute toxicity and quality of life after IMRT and concurrent chemotherapy for anal canal and perianal cancer.* Int J Radiat Oncol Biol Phys, 2014. **90**(3): p. 587-94.
124. Hosni, A., et al., *Impact of Definitive Chemoradiation on Quality-of-Life Changes for Patients With Anal Cancer: Long-term Results of a Prospective Study.* Dis Colon Rectum, 2022. **65**(5): p. 642-653.
125. Sangruangake, M., et al., *The Relationship between Unmet Need, Physical Symptoms, Psychological Well-Being and Health-Related Quality of Life in Cholangiocarcinoma Survivors.* Asian Pac J Cancer Prev, 2022. **23**(8): p. 2821-2828.
126. Sauter, C., et al., *Quality of life in patients treated with radiochemotherapy for primary diagnosis of anal cancer.* Sci Rep, 2022. **12**(1): p. 4416.
127. Shen, J.P., et al., *Efficacy of Systemic Chemotherapy in Patients With Low-grade Mucinous Appendiceal Adenocarcinoma: A Randomized Crossover Trial.* JAMA Netw Open, 2023. **6**(6): p. e2316161.
128. Sodergren, S.C., et al., *International Validation of the EORTC QLQ-ANL27, a Field Study to Test the Anal Cancer-Specific Health-Related Quality-of-Life Questionnaire.* Int J Radiat Oncol Biol Phys, 2023. **115**(5): p. 1155-1164.
129. Somjaivong, B., et al., *The influence of symptoms, social support, uncertainty, and coping on health-related quality of life among cholangiocarcinoma patients in northeast Thailand.* Cancer Nurs, 2011. **34**(6): p. 434-42.
130. Soni, M., et al., *Nivolumab in gastric/gastroesophageal junction cancer: real-world data from UK Early Access to Medicines Scheme.* Future Oncol, 2021. **17**(24): p. 3163-3174.
131. Stearns, A.T., et al., *Long-term Quality of Life After Cytoreductive Surgery and Heated Intraperitoneal Chemotherapy for Pseudomyxoma Peritonei: A Prospective Longitudinal Study.* Ann Surg Oncol, 2018. **25**(4): p. 965-973.
132. Subramani, V.N., et al., *Outcome Following Percutaneous Transhepatic Biliary Drainage (PTBD) in Carcinoma Gallbladder: a Prospective Observational Study.* J Gastrointest Cancer, 2022. **53**(3): p. 543-548.

133. Mortensen, G.L. and L. Lundby, *Patients use normalisation techniques to cope with the quality-of-life effects of anal cancer.* Dan Med J, 2015. **62**(3).
134. Darwish Murad, S., et al., *Excellent quality of life after liver transplantation for patients with perihilar cholangiocarcinoma who have undergone neoadjuvant chemoradiation.* Liver Transpl, 2013. **19**(5): p. 521-8.
135. Tang, Y., et al., *Quality of life after intensity-modulated radiation therapy for anal cancer.* Journal of Radiation Oncology, 2015. **4**(3): p. 291-298.
136. Joseph, K., et al., *Patient reported quality of life after helical IMRT based concurrent chemoradiation of locally advanced anal cancer.* Radiother Oncol, 2016. **120**(2): p. 228-33.
137. Aggarwal, R., et al., *Evaluation of high-dose-rate intraluminal brachytherapy by percutaneous transhepatic biliary drainage in the palliative management of malignant biliary obstruction--a pilot study.* Brachytherapy, 2013. **12**(2): p. 162-70.
138. Artifon, E.L., et al., *Surgery or EUS-guided choledochoduodenostomy for malignant distal biliary obstruction after ERCP failure.* Endosc Ultrasound, 2015. **4**(3): p. 235-43.
139. Axelsson, A., et al., *Patient-reported QoL in anal cancer survivors 3 and 6 years after treatment-results from the Swedish national ANCA study.* Support Care Cancer, 2022. **30**(5): p. 4169-4178.
140. Atkinson, T.M., et al., *Reliability and between-group stability of a health-related quality of life symptom index for persons with anal high-grade squamous intraepithelial lesions: an AIDS Malignancy Consortium Study (AMC-A03).* Qual Life Res, 2019. **28**(5): p. 1265-1269.
141. Ginesi, M., et al., *Patients with Anal Cancer Have Low Functional and High Symptomatic Health-Related Quality of Life Scores After Chemoradiation.* J Gastrointest Surg, 2023. **27**(2): p. 416-418.
142. Patel, N., et al., *Understanding Patient Experience in Biliary Tract Cancer: A Qualitative Patient Interview Study.* Oncol Ther, 2021. **9**(2): p. 557-573.
143. Pedersen, T.B., et al., *Quality of life following salvage surgery for squamous cell carcinoma of the anus.* Eur J Surg Oncol, 2019. **45**(6): p. 995-998.
144. Corrigan, K.L., et al., *Patient-Reported Outcomes After Chemoradiation in Patients With Anal Cancer: A Qualitative Analysis.* Adv Radiat Oncol, 2022. **7**(4): p. 100986.
145. Kaupp-Roberts, S.D., et al., *Validation of the EORTC QLQ-BIL21 questionnaire for measuring quality of life in patients with cholangiocarcinoma and cancer of the gallbladder.* Br J Cancer, 2016. **115**(9): p. 1032-1038.
146. Keilson, J.M., et al., *Patient reported outcomes: Financial toxicity is a barrier to clinical trials and personalized therapy in cholangiocarcinoma.* J Surg Oncol, 2022. **126**(6): p. 1003-1010.
147. Knowles, G., et al., *Late effects and quality of life after chemo-radiation for the treatment of anal cancer.* Eur J Oncol Nurs, 2015. **19**(5): p. 479-85.
148. Banihashem, S., et al., *Psychological Status and Quality of Life Associated with Radioactive Iodine Treatment of Patients with Differentiated Thyroid Cancer: Results of Hospital Anxiety and Depression Scale and Short-Form (36) Health Survey.* Indian J Nucl Med, 2020. **35**(3): p. 216-221.
149. Blefari, N.D.A., et al., *Long-Term Health-Related Quality of Life Outcomes Following Thyroid Surgery for Malignant or Benign Disease: Deficits Persist in Cancer Survivors Beyond Five Years.* World J Surg, 2022. **46**(10): p. 2423-2432.
150. Bongers, P.J., et al., *Differences in long-term quality of life between hemithyroidectomy and total thyroidectomy in patients treated for low-risk differentiated thyroid carcinoma.* Surgery, 2020. **167**(1): p. 94-101.
151. Borget, I., et al., *Quality of Life and Cost-Effectiveness Assessment of Radioiodine Ablation Strategies in Patients With Thyroid Cancer: Results From the Randomized Phase III ESTIMABL Trial.* J Clin Oncol, 2015. **33**(26): p. 2885-92.
152. Buttner, M., et al., *Quality of Life in Patients With Hypoparathyroidism After Treatment for Thyroid Cancer.* J Clin Endocrinol Metab, 2020. **105**(12).
153. Buttner, M., et al., *Quality of life of patients more than 1 year after surgery for thyroid cancer.* Hormones (Athens), 2020. **19**(2): p. 233-243.
154. Qu, F., et al., *Analysis of the Rehabilitation Efficacy and Nutritional Status of Patients After Endoscopic Radical Thyroidectomy by Fast Track Surgery Based on Nutritional Support.* Front Surg, 2022. **9**: p. 897616.
155. Dadu, R., et al., *Efficacy of the Natural Clay, Calcium Aluminosilicate Anti-Diarrheal, in Reducing Medullary Thyroid Cancer-Related Diarrhea and Its Effects on Quality of Life: A Pilot Study.* Thyroid, 2015. **25**(10): p. 1085-90.
156. de Rooij, B.H., et al., *Symptom clusters in 1330 survivors of 7 cancer types from the PROFILES registry: A network analysis.* Cancer, 2021. **127**(24): p. 4665-4674.
157. Diamond-Rossi, S.A., et al., *Looking under the hood of "the Cadillac of cancers:" radioactive iodine-related craniofacial side effects among patients with thyroid cancer.* J Cancer Surviv, 2020. **14**(6): p. 847-857.
158. Dingle, I.F., et al., *Salivary morbidity and quality of life following radioactive iodine for well-differentiated thyroid cancer.* Otolaryngol Head Neck Surg, 2013. **148**(5): p. 746-52.
159. Doubleday, A.R., et al., *What is the experience of our patients with transient hypoparathyroidism after total thyroidectomy?* Surgery, 2021. **169**(1): p. 70-76.
160. Frey, S., et al., *Impact of Permanent Post-thyroidectomy Hypoparathyroidism on Self-evaluation of Quality of Life and Voice: Results From the National QoL-Hypopara Study.* Ann Surg, 2021. **274**(5): p. 851-858.
161. Yaniv, D., et al., *Quality of life following lobectomy versus total thyroidectomy is significantly related to hypothyroidism.* J Surg Oncol, 2022. **126**(4): p. 640-648.
162. Yu, N.Y., et al., *Initial Experience with Proton Beam Therapy for Differentiated Thyroid Cancer.* Int J Part Ther, 2021. **8**(1): p. 311-318.
163. Lan, Y., et al., *Quality of Life in Papillary Thyroid Microcarcinoma Patients Undergoing Radiofrequency Ablation or Surgery: A Comparative Study.* Front Endocrinol (Lausanne), 2020. **11**: p. 249.
164. Lan, Y., et al., *The quality of life in papillary thyroid microcarcinoma patients undergoing lobectomy or total thyroidectomy: A cross-sectional study.* Cancer Med, 2021. **10**(6): p. 1989-2002.
165. Lee, J., et al., *Comparative analysis of oncological outcomes and quality of life after robotic versus conventional open thyroidectomy with modified radical neck dissection in patients with papillary thyroid carcinoma and lateral neck node metastases.* J Clin Endocrinol Metab, 2013. **98**(7): p. 2701-8.
166. Lee, M.C., et al., *Comparison of quality of life between open and endoscopic thyroidectomy for papillary thyroid cancer.* Head & Neck, 2016. **38**(S1): p. E827-E831.
167. Lee, J., et al., *Quality of Life of Survivors of Thyroid Cancer Is Not Inferior to That in Subjects without Cancer: Long-Term after Over 5 Years.* Endocrinol Metab (Seoul), 2022. **37**(4): p. 664-673.
168. Li, J., et al., *Risk Factors of Deterioration in Quality of Life Scores in Thyroid Cancer Patients After Thyroidectomy.* Cancer Manag Res, 2019. **11**: p. 10593-10598.

169. Li, J., et al., *Health-related quality of life analysis in differentiated thyroid carcinoma patients after thyroidectomy.* Sci Rep, 2020. **10**(1): p. 5765.
170. Li, T., et al., *Comparison of quality of life and cosmetic result between open and transaxillary endoscopic thyroid lobectomy for papillary thyroid microcarcinoma survivors: A single-center prospective cohort study.* Cancer Med, 2022. **11**(22): p. 4146-4156.
171. Liu, T., et al., *Mindfulness-based stress reduction in patients with differentiated thyroid cancer receiving radioactive iodine therapy: a randomized controlled trial.* Cancer Manag Res, 2019. **11**: p. 467-474.
172. Liu, Y.H., et al., *Appearance characteristics of incision, satisfaction with the aesthetic effect, and quality of life in of thyroid cancer patients after thyroidectomy.* Int J Health Plann Manage, 2021. **36**(3): p. 784-792.
173. Lubitz, C.C., et al., *Measurement and Variation in Estimation of Quality of Life Effects of Patients Undergoing Treatment for Papillary Thyroid Carcinoma.* Thyroid, 2017. **27**(2): p. 197-206.
174. Luddy, M.K., et al., *Patient Reported Outcome Measures of Health-Related Quality of Life and Asthenia after Thyroidectomy.* J Surg Res, 2021. **264**: p. 394-401.
175. Lan, Y., et al., *Factors associated with health-related quality of life in papillary thyroid microcarcinoma patients undergoing radiofrequency ablation: a cross-sectional prevalence study.* Int J Hyperthermia, 2020. **37**(1): p. 1174-1181.
176. Lv, J., et al., *Study on the correlation between postoperative mental flexibility, negative emotions, and quality of life in patients with thyroid cancer.* Gland Surgery, 2021. **10**(8): p. 2471-2476.
177. Wang, T., et al., *Health-Related Quality of Life of Community Thyroid Cancer Survivors in Hangzhou, China.* Thyroid, 2018. **28**(8): p. 1013-1023.
178. Wirth, U., et al., *Quality of life and surgical outcome of ABBA versus EndoCATS endoscopic thyroid surgery: a single center experience.* Surg Endosc, 2022. **36**(2): p. 968-979.
179. Wirth, L.J., et al., *Patient-Reported Outcomes with Selpercatinib Treatment Among Patients with RET-Mutant Medullary Thyroid Cancer in the Phase I/II LIBRETTO-001 Trial.* The Oncologist, 2021. **27**(1): p. 13-21.
180. Wongwattana, P., P. Laoveerakul, and A. Santeerapharp, *A comparison of efficacy and quality of life between transoral endoscopic thyroidectomy vestibular approach (TOETVA) and endoscopic thyroidectomy axillo-breast approach (ETABA) in thyroid surgery: non-randomized clinical trial.* European Archives of Oto-Rhino-Laryngology, 2021. **278**.
181. Wu, H.X., et al., *Psychological and behavioral intervention improves the quality of life and mental health of patients suffering from differentiated thyroid cancer treated with postoperative radioactive iodine-131.* Neuropsychiatr Dis Treat, 2016. **12**: p. 1055-60.
182. Haraj, N.E., et al., *Evaluation of the quality of life in patients followed for differentiated cancer of the thyroid.* Ann Endocrinol (Paris), 2019. **80**(1): p. 26-31.
183. Hedman, C., et al., *Determinants of long-term quality of life in patients with differentiated thyroid carcinoma - a population-based cohort study in Sweden.* Acta Oncol, 2016. **55**(3): p. 365-9.
184. Hedman, C., et al., *Effect of Thyroid-Related Symptoms on Long-Term Quality of Life in Patients with Differentiated Thyroid Carcinoma: A Population-Based Study in Sweden.* Thyroid, 2017. **27**(8): p. 1034-1042.
185. Hedman, C., et al., *Fear of Recurrence and View of Life Affect Health-Related Quality of Life in Patients with Differentiated Thyroid Carcinoma: A Prospective Swedish Population-Based Study.* Thyroid, 2018.
186. Huang, J.K., et al., *Quality of life and cosmetic result of single-port access endoscopic thyroidectomy via axillary approach in patients with papillary thyroid carcinoma.* Onco Targets Ther, 2016. **9**: p. 4053-9.
187. Huang, M., et al., *Health literacy and fatigue, anxiety, depression, and somatic symptoms in patients with differentiated thyroid carcinoma from West China: A cross-sectional study.* Health Sci Rep, 2023. **6**(1): p. e1018.
188. Husson, O., et al., *Health-related quality of life and disease specific symptoms in long-term thyroid cancer survivors: a study from the population-based PROFILES registry.* Acta Oncol, 2013. **52**(2): p. 249-58.
189. Husson, O., et al., *Development of a disease-specific health-related quality of life questionnaire (THYCA-QoL) for thyroid cancer survivors.* Acta Oncol, 2013. **52**(2): p. 447-54.
190. Husson, O., et al., *Fatigue among short- and long-term thyroid cancer survivors: results from the population-based PROFILES registry.* Thyroid, 2013. **23**(10): p. 1247-55.
191. Schoormans, D., et al., *Negative illness perceptions are related to poorer health-related quality of life among thyroid cancer survivors: Results from the PROFILES registry.* Head Neck, 2020. **42**(9): p. 2533-2541.
192. Shen, S., et al., *Comparing quality of life between patients undergoing trans-areola endoscopic thyroid surgery and trans-oral endoscopic thyroid surgery.* BMC Surgery, 2021. **21**(1): p. 277.
193. Singer, S., et al., *Quality of life in patients with thyroid cancer compared with the general population.* Thyroid, 2012. **22**(2): p. 117-24.
194. Singer, S., et al., *Quality-of-Life Priorities in Patients with Thyroid Cancer: A Multinational European Organisation for Research and Treatment of Cancer Phase I Study.* Thyroid, 2016. **26**(11): p. 1605-1613.
195. Song, C.M., et al., *Quality of life after robotic thyroidectomy by a gasless unilateral axillary approach.* Ann Surg Oncol, 2014. **21**(13): p. 4188-94.
196. Song, Q., et al., *Radiofrequency ablation versus total thyroidectomy in patients with papillary thyroid microcarcinoma located in the isthmus: a retrospective cohort study.* Int J Hyperthermia, 2021. **38**(1): p. 708-714.
197. Steenaard, R.V., et al., *Patient and Partner Perspectives on Health-Related Quality of Life in Adrenocortical Carcinoma.* J Endocr Soc, 2020. **4**(5): p. bvaa040.
198. Steenaard, R.V., et al., *Health-Related Quality of Life in Adrenocortical Carcinoma: Development of the Disease-Specific Questionnaire ACC-QOL and Results from the PROFILES Registry.* Cancers (Basel), 2022. **14**(6).
199. Maki, Y., K. Horiuchi, and T. Okamoto, *Fatigue and quality of life among thyroid cancer survivors without persistent or recurrent disease.* Endocr Connect, 2022. **11**(2).
200. Ming, H., et al., *Effect of radioiodine therapy under thyroid hormone withdrawal on health-related quality of life in patients with differentiated thyroid cancer.* Jpn J Clin Oncol, 2022. **52**(10): p. 1159-1166.
201. Missaoui, A.M., et al., *Health-related quality of life in long-term differentiated thyroid cancer survivors: A cross-sectional Tunisian-based study.* Front Endocrinol (Lausanne), 2022. **13**: p. 999331.

202. Mols, F., et al., *Age-related differences in health-related quality of life among thyroid cancer survivors compared with a normative sample: Results from the PROFILES Registry.* Head Neck, 2018. **40**(10): p. 2235-2245.
203. Mongelli, M.N., et al., *Financial burden and quality of life among thyroid cancer survivors.* Surgery, 2020. **167**(3): p. 631-637.
204. Moon, J.H., et al., *Effect of Initial Treatment Choice on 2-year Quality of Life in Patients with Low-risk Papillary Thyroid Microcarcinoma.* J Clin Endocrinol Metab, 2021. **106**(3): p. 724-735.
205. Moss, L., et al., *Medullary Thyroid Cancer Patient's Assessment of Quality of Life Tools: Results from the QaLM Study.* Eur Thyroid J, 2021. **10**(1): p. 72-78.
206. Rajamanickam, S., et al., *Quality of life comparison in thyroxine hormone withdrawal versus triiodothyronine supplementation prior to radioiodine ablation in differentiated thyroid carcinoma: a prospective cohort study in the Indian population.* Eur Arch Otorhinolaryngol, 2022. **279**(4): p. 2011-2018.
207. Ramim, J.E., et al., *Health-related quality of life of thyroid cancer patients undergoing radioiodine therapy: a cohort real-world study in a reference public cancer hospital in Brazil.* Support Care Cancer, 2020. **28**(8): p. 3771-3779.
208. Rani, D., et al., *Examining recombinant human TSH primed (1)(3)(1)I therapy protocol in patients with metastatic differentiated thyroid carcinoma: comparison with the traditional thyroid hormone withdrawal protocol.* Eur J Nucl Med Mol Imaging, 2014. **41**(9): p. 1767-80.
209. Rogers, S.N., et al., *Health-related quality of life, fear of recurrence, and emotional distress in patients treated for thyroid cancer.* Br J Oral Maxillofac Surg, 2017. **55**(7): p. 666-673.
210. Ryu, C.H., et al., *Development and Evaluation of a Korean Version of a Thyroid-Specific Quality-of-Life Questionnaire Scale in Thyroid Cancer Patients.* Cancer Res Treat, 2018. **50**(2): p. 405-415.
211. Taieb, D., et al., *Heath-related quality of life in thyroid cancer patients following radioiodine ablation.* Health Qual Life Outcomes, 2011. **9**: p. 33.
212. Tang, C.Y.L., et al., *Recombinant human thyrotropin versus thyroid hormone withdrawal in an Asian population.* Endocrine, 2020. **69**(1): p. 126-132.
213. Taylor, M.H., et al., *Health-related quality-of-life analyses from a multicenter, randomized, double-blind phase 2 study of patients with differentiated thyroid cancer treated with lenvatinib 18 or 24 mg/day.* Cancer Med, 2023. **12**(4): p. 4332-4342.
214. Vaisman, F., et al., *Is There a Role for Peptide Receptor Radionuclide Therapy in Medullary Thyroid Cancer?* Clinical Nuclear Medicine, 2015. **40**(2): p. 123-127.
215. van Gelder, T., et al., *Assessing health-related quality of life in cancer survivors: factors impacting on EORTC QLU-C10D-derived utility values.* Qual Life Res, 2020. **29**(6): p. 1483-1494.
216. van Gerwen, M., et al., *Patient-reported outcomes following total thyroidectomy and lobectomy in thyroid cancer survivors: an analysis of the PROFILES Registry data.* Support Care Cancer, 2022. **30**(1): p. 687-693.
217. Vega-Vázquez, M.A., et al., *Quality of life-in patients with differentiated thyroid cancer at the general endocrinology clinics of the University Hospital of Puerto Rico.* Bol Asoc Med P R, 2015. **107**(1): p. 25-31.
218. Vissers, P.A., et al., *The impact of comorbidity on Health-Related Quality of Life among cancer survivors: analyses of data from the PROFILES registry.* J Cancer Surviv, 2013. **7**(4): p. 602-13.
219. Jeon, M.J., et al., *Quality of Life in Patients with Papillary Thyroid Microcarcinoma Managed by Active Surveillance or Lobectomy: A Cross-Sectional Study.* Thyroid, 2019. **29**(7): p. 956-962.
220. Juzwiszyn, J., A. Dolczewska, and M. Chabowski, *Quality of Life and acceptance of illness in patients who underwent total thyroidectomy.* Ann Ital Chir, 2020. **91**: p. 352-358.
221. Nervo, A., et al., *Quality of Life during Treatment with Lenvatinib for Thyroid Cancer: The Patients' Perspective beyond the Medical Evaluation.* Eur Thyroid J, 2021. **10**(1): p. 65-71.
222. Nickel, B., et al., *Health-Related Quality of Life After Diagnosis and Treatment of Differentiated Thyroid Cancer and Association With Type of Surgical Treatment.* JAMA Otolaryngol Head Neck Surg, 2019. **145**(3): p. 231-238.
223. Nygaard, B., et al., *A placebo-controlled, blinded and randomised study on the effects of recombinant human thyrotropin on quality of life in the treatment of thyroid cancer.* Eur Thyroid J, 2013. **2**(3): p. 195-202.
224. Xue, S., et al., *Supraclavicular Approach of Lobectomy Improves Quality of Life for Patients With Unilateral Papillary Thyroid Microcarcinoma: A Prospective Cohort Study.* Front Endocrinol (Lausanne), 2021. **12**: p. 766444.
225. Ahn, J., et al., *Quality of Life in Patients with Papillary Thyroid Microcarcinoma According to Treatment: Total Thyroidectomy with or without Radioactive Iodine Ablation.* Endocrinol Metab (Seoul), 2020. **35**(1): p. 115-121.
226. Alyousef, M.Y., et al., *Long-Term Quality of Life (5-15 Years Post-Thyroidectomy) of Thyroid Carcinoma Patients in Two Tertiary Care Hospitals.* Cureus, 2022. **14**(2): p. e22005.
227. Aschebrook-Kilfoy, B., et al., *Risk Factors for Decreased Quality of Life in Thyroid Cancer Survivors: Initial Findings from the North American Thyroid Cancer Survivorship Study.* Thyroid, 2015. **25**(12): p. 1313-21.
228. Roerink, S.H., et al., *High prevalence of self-reported shoulder complaints after thyroid carcinoma surgery.* Head Neck, 2017. **39**(2): p. 260-268.
229. Gal, T.J., et al., *Quality of life impact of external beam radiotherapy for advanced thyroid carcinoma.* Thyroid, 2013. **23**(1): p. 64-9.
230. Gallop, K., et al., *A qualitative evaluation of the validity of published health utilities and generic health utility measures for capturing health-related quality of life (HRQL) impact of differentiated thyroid cancer (DTC) at different treatment phases.* Quality of Life Research, 2015. **24**(2): p. 325-338.
231. Gamper, E.M., et al., *Persistent quality of life impairments in differentiated thyroid cancer patients: results from a monitoring programme.* Eur J Nucl Med Mol Imaging, 2015. **42**(8): p. 1179-88.
232. Giani, C., et al., *Safety and Quality-of-Life Data from an Italian Expanded Access Program of Lenvatinib for Treatment of Thyroid Cancer.* Thyroid, 2021. **31**(2): p. 224-232.
233. Giusti, M., et al., *Five-year longitudinal evaluation of quality of life in a cohort of patients with differentiated thyroid carcinoma.* J Zhejiang Univ Sci B, 2011. **12**(3): p. 163-73.
234. Giusti, M., et al., *Evaluation of Quality of Life in Patients with Differentiated Thyroid Cancer by Means of the Thyroid-Specific Patient-Reported Outcome Questionnaire: A 5-Year Longitudinal Study.* Eur Thyroid J, 2020. **9**(5): p. 247-255.
235. Gkatzia, N., et al., *Quality of Life Survey Following Radioiodine Ablation in Patients with Differentiated Thyroid Cancer.* SN Comprehensive Clinical Medicine, 2021. **3**(1): p. 158-165.
236. Goswami, S., et al., *Benchmarking health-related quality of life in thyroid cancer versus other cancers and United States normative data.* Surgery, 2018. **164**(5): p. 986-992.

237. Goswami, S., et al., *Clinical factors associated with worse quality-of-life scores in United States thyroid cancer survivors.* Surgery, 2019. **166**(1): p. 69-74.
238. Gou, J., et al., *Health-related quality-of-life assessment in surgical patients with papillary thyroid carcinoma: A single-center analysis from Mainland China.* Medicine (Baltimore), 2017. **96**(38): p. e8070.
239. Pak, K., et al., *Impact of age and sex on the quality of life following radioactive iodine ablation in patients with thyroid cancer.* Nuklearmedizin, 2017. **56**(5): p. 177-183.
240. Li, G. and L. Wu, *Total or Near-total Thyroidectomy in treatment of Thyroid Cancer.* Pak J Med Sci, 2022. **38**(6): p. 1662-1667.
241. Chan, W.L., et al., *Health-Related Quality of Life in Asian Differentiated Thyroid Cancer Survivors.* Cancer Control, 2021. **28**: p. 10732748211029726.
242. Chen, W., et al., *Association of Total Thyroidectomy or Thyroid Lobectomy With the Quality of Life in Patients With Differentiated Thyroid Cancer With Low to Intermediate Risk of Recurrence.* JAMA Surg, 2022. **157**(3): p. 200-209.
243. Chen, L., et al., *Reminiscence therapy care program as a potential nursing intervention to relieve anxiety, depression, and quality of life in older papillary thyroid carcinoma patients: A randomized, controlled study.* Frontiers in Psychology, 2022. **13**.
244. Chen, W.-H. and C.-Y. Chen, *Postoperative quality of life and cosmetic outcome between minimally invasive video-assisted thyroidectomy and bilateral axillo-breast approach robotic thyroidectomy: a single center retrospective cohort study.* Updates in Surgery, 2021. **73**(4): p. 1459-1465.
245. Choi, Y., et al., *Impact of postthyroidectomy scar on the quality of life of thyroid cancer patients.* Ann Dermatol, 2014. **26**(6): p. 693-9.
246. Chow, K.Y., et al., *Association between quality of life and patient-reported complications from surgery and radioiodine in early-stage thyroid cancer survivors: A matched-pair analysis.* Surgery, 2021. **170**(2): p. 462-468.
247. Kim, W.W., et al., *Comparison of the Quality of Life for Thyroid Cancer Survivors Who Had Open Versus Robotic Thyroidectomy.* J Laparoendosc Adv Surg Tech A, 2016. **26**(8): p. 618-24.
248. Kong, S.H., et al., *Longitudinal Assessment of Quality of Life According to Treatment Options in Low-Risk Papillary Thyroid Microcarcinoma Patients: Active Surveillance or Immediate Surgery (Interim Analysis of MAeSTro).* Thyroid, 2019. **29**(8): p. 1089-1096.
249. Gulsoy Kirnap, N., et al., *The effect of iatrogenic subclinical hyperthyroidism on anxiety, depression and quality of life in differentiated thyroid carcinoma.* Turk J Med Sci, 2020. **50**(4): p. 870-876.
250. Kurumety, S.K., et al., *Post-thyroidectomy neck appearance and impact on quality of life in thyroid cancer survivors.* Surgery, 2019. **165**(6): p. 1217-1221.
251. Kent, E.E., et al., *Health-related quality of life in older adult survivors of selected cancers: data from the SEER-MHOS linkage.* Cancer, 2015. **121**(5): p. 758-65.
252. Husson, O., et al., *The EORTC QLQ-C30 Summary Score as Prognostic Factor for Survival of Patients with Cancer in the "Real-World": Results from the Population-Based PROFILES Registry.* Oncologist, 2020. **25**(4): p. e722-e732.
253. Yang, Y., et al., *Clinical Treatment Efficacy of Total Thyroidectomy Combined with Radioactive Iodine on Treatment of Thyroid Cancer and Its Effect on the Quality of Life of Patients.* Iran J Public Health, 2019. **48**(8): p. 1461-1468.
254. Hamdan, N.A., N. Abd Hamid, and M.F.I. Leong Bin Abdullah, *A longitudinal investigation of posttraumatic growth and its associated factors among head and neck cancer survivors.* Psycho-Oncology, 2022. **31**(3): p. 504-511.
255. Patterson, J.M., et al., *Associations between markers of social functioning and depression and quality of life in survivors of head and neck cancer: Findings from the Head and Neck Cancer 5000 study.* Psycho-Oncology, 2022. **31**(3): p. 478-485.
256. Bachmann, A.S., et al., *Well-being and quality of life among oral cancer patients – Psychological vulnerability and coping responses upon entering initial treatment.* Journal of Cranio-Maxillofacial Surgery, 2018. **46**(9): p. 1637-1644.
257. Bajwa, H.K., R. Singareddy, and K.R. Alluri, *High-dose-rate interstitial brachytherapy in oral cancer-Its impact on quality of life.* Brachytherapy, 2016. **15**(3): p. 381-386.
258. Becker, C., A.M. Becker, and J. Pfeiffer, *Health-related quality of life in patients with nasal prosthesis.* J Craniomaxillofac Surg, 2016. **44**(1): p. 75-9.
259. Becker, C., et al., *Health-related quality of life in patients with major salivary gland carcinoma.* Eur Arch Otorhinolaryngol, 2018. **275**(4): p. 997-1003.
260. Li, J.B., et al., *Longitudinal Trend of Health-Related Quality of Life During Concurrent Chemoradiotherapy and Survival in Patients With Stage II-IVb Nasopharyngeal Carcinoma.* Front Oncol, 2020. **10**: p. 579292.
261. Pan, X.-B., et al., *Concurrent chemoradiotherapy degrades the quality of life of patients with stage II nasopharyngeal carcinoma as compared to radiotherapy.* Oncotarget, 2017. **8**(8).
262. Bozec, A., et al., *Long-term functional and quality of life outcomes in laryngectomized patients after successful voice restoration using tracheoesophageal prostheses.* Surg Oncol, 2021. **38**: p. 101580.
263. Iriya, P.M.O., et al., *Health-related quality of life of patients with squamous cell carcinoma: a comparison according to tumor location.* Braz Oral Res, 2017. **31**: p. e105.
264. Daugaard, R., et al., *Association between late effects assessed by physicians and quality of life reported by head-and-neck cancer survivors.* Acta Oncologica, 2017. **56**(2): p. 342-347.
265. Davudov, M.M., et al., *Quality of life in patients with oral cancer treated by different reconstruction methods as measured by the EORTC QLQ-H&N43.* Br J Oral Maxillofac Surg, 2020. **58**(9): p. e67-e74.
266. de Vries, J., et al., *Frailty is associated with decline in health-related quality of life of patients treated for head and neck cancer.* Oral Oncol, 2020. **111**: p. 105020.
267. Deckard, N.A., et al., *Comparative analysis of quality-of-life metrics after endoscopic surgery for sinonasal neoplasms.* Am J Rhinol Allergy, 2015. **29**(2): p. 151-5.
268. Derousseau, T., et al., *Long-term changes in quality of life after endoscopic resection of sinonasal and skull-base tumors.* Int Forum Allergy Rhinol, 2015. **5**(12): p. 1129-35.
269. Deschuymer, S., et al., *Randomized Clinical Trial on Reduction of Radiotherapy Dose to the Elective Neck in Head and Neck Squamous Cell Carcinoma: Results on the Quality of Life.* Qual Life Res, 2021. **30**(1): p. 117-127.
270. Dinescu, F.V., et al., *Evaluation of health-related quality of life with EORTC QLQ-C30 and QLQ-H&N35 in Romanian laryngeal cancer patients.* Eur Arch Otorhinolaryngol, 2016. **273**(9): p. 2735-40.
271. Doss, J.G., et al., *Changes in health-related quality of life of oral cancer patients treated with curative intent: experience of a developing country.* Int J Oral Maxillofac Surg, 2017. **46**(6): p. 687-698.
272. Farrugia, M., et al., *A Principal Component of Quality of Life Measures Is Associated with Survival for Head and Neck Cancer Patients Treated with Radiation Therapy.* Cancers (Basel), 2021. **13**(5).

273. Si, Y.F., et al., *Influence of endoscopic sinus surgery on the quality of life of patients with early nasopharyngeal carcinoma and the analysis of prognosis-related factors.* Tumour Biol, 2017. **39**(7): p. 1010428317707435.
274. Yang, Q., et al., *The impact of induction chemotherapy on long-term quality of life in patients with locoregionally advanced nasopharyngeal carcinoma: Outcomes from a randomised phase 3 trial.* Oral Oncol, 2021. **121**: p. 105494.
275. Yanwei, L., et al., *Traditional Chinese medicine formula 01 for nasopharyngeal carcinoma (NPC01) for head & neck cancer and health-related quality of life: a retrospective study.* BMC Complement Med Ther, 2022. **22**(1): p. 216.
276. Lastrucci, L., et al., *Late toxicity, evolving radiotherapy techniques, and quality of life in nasopharyngeal carcinoma.* Radiol Med, 2017. **122**(4): p. 303-308.
277. Lee, E., et al., *Comparison of quality of life outcomes in a de-intensification treatment regimen for p16 + oropharyngeal cancer.* Eur Arch Otorhinolaryngol, 2022. **279**(9): p. 4533-4540.
278. Liao, J., et al., *A preliminary study on ultrasound-guided percutaneous microwave ablation for palliative treatment of advanced head and neck malignancies.* Int J Hyperthermia, 2021. **38**(1): p. 479-487.
279. Linsen, S.S., N.C. Gellrich, and G. Kruskemper, *Age- and localization-dependent functional and psychosocial impairments and health related quality of life six months after OSCC therapy.* Oral Oncol, 2018. **81**: p. 61-68.
280. Locati, L.D., et al., *Patients with adenoid cystic carcinomas of the salivary glands treated with lenvatinib: Activity and quality of life.* Cancer, 2020. **126**(9): p. 1888-1894.
281. Loimu, V., et al., *Health-related quality of life of head and neck cancer patients with successful oncological treatment.* Eur Arch Otorhinolaryngol, 2015. **272**(9): p. 2415-23.
282. Nascimento, M.L., et al., *Impact of xerostomia on the quality of life of patients submitted to head and neck radiotherapy.* Med Oral Patol Oral Cir Bucal, 2019. **24**(6): p. e770-e775.
283. Lopez-Jornet, P., et al., *Assessing quality of life in patients with head and neck cancer in Spain by means of EORTC QLQ-C30 and QLQ-H&N35.* J Craniomaxillofac Surg, 2012. **40**(7): p. 614-20.
284. Lu, N., T. Qin, and W. Hu, *Impact of changes in psychological resilience during treatment with intensity-modulated radiotherapy on nasopharyngeal carcinoma patients: a prospective study.* Ann Palliat Med, 2022. **11**(1): p. 123-134.
285. Chan, Y.W., V.L. Chow, and W.I. Wei, *Quality of life of patients after salvage nasopharyngectomy for recurrent nasopharyngeal carcinoma.* Cancer, 2012. **118**(15): p. 3710-8.
286. Williamson, J.S., D. Ingrams, and H. Jones, *Quality of life after treatment of laryngeal carcinoma: a single centre cross-sectional study.* Ann R Coll Surg Engl, 2011. **93**(8): p. 591-5.
287. Handschel, J., et al., *Psychological aspects affect quality of life in patients with oral squamous cell carcinomas.* Psychooncology, 2013. **22**(3): p. 677-82.
288. Hegde, J.V., et al., *Patient-reported quality-of-life outcomes after de-escalated chemoradiation for human papillomavirus-positive oropharyngeal carcinoma: Findings from a phase 2 trial.* Cancer, 2018. **124**(3): p. 521-529.
289. Hong, J.S., et al., *Quality of life of nasopharyngeal cancer survivors in China.* Curr Oncol, 2015. **22**(3): p. e142-7.
290. Horn, D., et al., *Prospective feasibility analysis of salvage surgery in recurrent oral cancer in terms of quality of life.* Oral Oncol, 2020. **102**: p. 104580.
291. Hsing, C.-Y., et al., *Comparison between free flap and pectoralis major pedicled flap for reconstruction in oral cavity cancer patients – A quality of life analysis.* Oral Oncology, 2011. **47**(6): p. 522-527.
292. Huang, T.L., et al., *Quality of life and survival outcome for patients with nasopharyngeal carcinoma treated by volumetric-modulated arc therapy versus intensity-modulated radiotherapy.* Radiat Oncol, 2020. **15**(1): p. 84.
293. Huang, Z., et al., *Survival and long-term quality-of-life of concurrent chemoradiotherapy versus surgery followed by radiotherapy with or without concurrent chemotherapy for the treatment of resectable stage III/IV hypopharyngeal carcinoma.* Asian Journal of Surgery, 2023. **46**(9): p. 3693-3699.
294. Scott, S.I., et al., *Long-term quality of life & functional outcomes after treatment of oropharyngeal cancer.* Cancer Med, 2021. **10**(2): p. 483-495.
295. Soares, J.R.N., et al., *Assessment of quality of life in patients with advanced oral cancer who underwent mandibulectomy with or without bone reconstruction.* Rev Assoc Med Bras (1992), 2018. **64**(8): p. 710-716.
296. Spiegel, J.L., et al., *Quality of life in patients after reconstruction with the supraclavicular artery island flap (SCAIF) versus the radial free forearm flap (RFFF).* Eur Arch Otorhinolaryngol, 2019. **276**(8): p. 2311-2318.
297. Spinato, G., et al., *Multicenter research into the quality of life of patients with advanced oropharyngeal carcinoma with long-term survival associated with human papilloma virus.* Oncol Lett, 2017. **14**(1): p. 185-193.
298. McDonald, C., et al., *Health-related quality of life in patients with T1N0 oral squamous cell carcinoma: selective neck dissection compared with wait and watch surveillance.* Br J Oral Maxillofac Surg, 2019. **57**(7): p. 649-654.
299. Mucke, T., et al., *Quality of life after different oncologic interventions in head and neck cancer patients.* J Craniomaxillofac Surg, 2015. **43**(9): p. 1895-8.
300. Rana, M., et al., *Prospective study of the influence of psychological and medical factors on quality of life and severity of symptoms among patients with oral squamous cell carcinoma*. Br J Oral Maxillofac Surg, 2015. **53**(4): p. 364-70.
301. Ranta, P., et al., *Long-term Quality of Life After Treatment of Oropharyngeal Squamous Cell Carcinoma.* Laryngoscope, 2021. **131**(4): p. E1172-E1178.
302. Riva, G., et al., *Quality of Life in Electrochemotherapy for Cutaneous and Mucosal Head and Neck Tumors.* J Clin Med, 2021. **10**(19).
303. Rogers, S.N., et al., *Health related quality of life following the treatment of oropharyngeal cancer by transoral laser.* Eur Arch Otorhinolaryngol, 2016. **273**(11): p. 3913-3920.
304. Ruhle, A., et al., *Surviving Elderly Patients with Head-and-Neck Squamous Cell Carcinoma-What Is the Long-Term Quality of Life after Curative Radiotherapy?* Cancers (Basel), 2021. **13**(6).
305. Thomas, C.M., et al., *Longitudinal Assessment of Frailty and Quality of Life in Patients Undergoing Head and Neck Surgery.* Laryngoscope, 2021. **131**(7): p. E2232-E2242.
306. Hung, L.T., et al., *Oncologic results and quality of life in patients with squamous cell carcinoma of hypopharynx after transoral laser microsurgery.* Lasers Surg Med, 2018. **50**(2): p. 117-124.
307. Lee, T.-F., et al., *Health-related quality of life outcome evaluation for intensity-modulated radiotherapy versus helical tomotherapy using EORTC QLQ-C30 and EORTC QLQ-HN35 core questionnaires for nasopharyngeal carcinomas.* Scientific Research and Essays, 2011. **6**: p. 3389-3398.

308. Tuomi, L., et al., *Health-related quality of life and voice following radiotherapy for laryngeal cancer--a comparison between glottic and supraglottic tumours.* Acta Oncol, 2015. **54**(1): p. 73-9.
309. Tyler, M.A., et al., *Long-term quality of life after definitive treatment of sinonasal and nasopharyngeal malignancies.* Laryngoscope, 2020. **130**(1): p. 86-93.
310. van Nieuwenhuizen, A.J., et al., *Patient-reported physical activity and the association with health-related quality of life in head and neck cancer survivors.* Support Care Cancer, 2018. **26**(4): p. 1087-1095.
311. Jang-Chun, L., et al., *Comparisons of quality of life for patients with nasopharyngeal carcinoma after treatment with different RT technologies.* Acta Otorhinolaryngol Ital, 2014. **34**(4): p. 241-6.
312. Nemeth, D., et al., *Importance of chewing, saliva, and swallowing function in patients with advanced oral cancer undergoing preoperative chemoradiotherapy: a prospective study of quality of life.* Int J Oral Maxillofac Surg, 2017. **46**(10): p. 1229-1236.
313. Xu, Y., et al., *Effect of Percutaneous Endoscopic Gastrostomy on Quality of Life after Chemoradiation for Locally Advanced Nasopharyngeal Carcinoma: A Cross-Sectional Study.* Curr Oncol, 2023. **30**(1): p. 1000-1009.
314. Abel, E., et al., *Impact on quality of life of IMRT versus 3-D conformal radiation therapy in head and neck cancer patients: A case control study.* Adv Radiat Oncol, 2017. **2**(3): p. 346-353.
315. Achim, V., et al., *Long-term Functional and Quality-of-Life Outcomes After Transoral Robotic Surgery in Patients With Oropharyngeal Cancer.* JAMA Otolaryngol Head Neck Surg, 2018. **144**(1): p. 18-27.
316. Aro, K., et al., *Trends in the 15D health-related quality of life over the first year following diagnosis of head and neck cancer.* Eur Arch Otorhinolaryngol, 2016. **273**(8): p. 2141-50.
317. Azevedo, E.H., et al., *Vocal handicap and quality of life after treatment of advanced squamous carcinoma of the larynx and/or hypopharynx.* J Voice, 2012. **26**(2): p. e63-71.
318. Zaoui, K., et al., *Quality of life after nasal cancer resection - surgical versus prosthetic rehabilitation.* Rhinology, 2018. **56**(4): p. 400-406.
319. Hamilton, S.N., et al., *Patient-reported outcome measures in patients undergoing radiotherapy for head and neck cancer.* Support Care Cancer, 2021. **29**(5): p. 2537-2547.
320. Yue, J., et al., *Long-term quality of life measured by the University of Washington QoL questionnaire (version 4) in patients with oral cancer treated with or without reconstruction with a microvascular free flap.* Br J Oral Maxillofac Surg, 2018. **56**(6): p. 475-481.
321. You, Q., et al., *Comparison of functional outcomes and health-related quality of life one year after treatment in patients with oral and oropharyngeal cancer treated with three different reconstruction methods.* Br J Oral Maxillofac Surg, 2020. **58**(7): p. 759-765.
322. Gabriele, M., et al., *Quality of life, swallowing and speech outcomes after oncological treatment for mobile tongue carcinoma.* European Journal of Plastic Surgery, 2020. **43**(3): p. 247-256.
323. Glicksman, J.T., et al., *Sinonasal quality of life after endoscopic resection of malignant sinonasal and skull base tumors.* Laryngoscope, 2018. **128**(4): p. 789-793.
324. Goetz, C., et al., *Hospital Based Quality of Life in Oral Cancer Surgery.* Cancers (Basel), 2020. **12**(8).
325. Govers, T.M., et al., *Quality of life after different procedures for regional control in oral cancer patients: cross-sectional survey.* Clin Otolaryngol, 2016. **41**(3): p. 228-33.
326. Guibert, M., et al., *Quality of life in patients treated for advanced hypopharyngeal or laryngeal cancer.* Eur Ann Otorhinolaryngol Head Neck Dis, 2011. **128**(5): p. 218-23.
327. de Pauli Paglioni, M., et al., *The impact of radiation caries in the quality of life of head and neck cancer patients.* Support Care Cancer, 2020. **28**(6): p. 2977-2984.
328. Pan, X.B., et al., *Intensity-modulated radiotherapy provides better quality of life than two-dimensional conventional radiotherapy for patients with stage II nasopharyngeal carcinoma.* Oncotarget, 2017. **8**(28): p. 46211-46218.
329. Peisker, A., et al., *Longterm quality of life after oncologic surgery and microvascular free flap reconstruction in patients with oral squamous cell carcinoma.* Med Oral Patol Oral Cir Bucal, 2016. **21**(4): p. e420-4.
330. Petrides, G.A., et al., *Health-related quality of life in maxillectomy patients undergoing dentoalveolar rehabilitation.* Oral Oncol, 2022. **126**: p. 105757.
331. Chen, W.C., et al., *Scintigraphic assessment of salivary function after intensity-modulated radiotherapy for head and neck cancer: correlations with parotid dose and quality of life.* Oral Oncol, 2013. **49**(1): p. 42-8.
332. Chen, A.M., et al., *Comparison of functional outcomes and quality of life between transoral surgery and definitive chemoradiotherapy for oropharyngeal cancer.* Head & Neck, 2015. **37**(3): p. 381-385.
333. Chen, J., et al., *Influence of Intensity-Modulated Radiation Therapy on the Life Quality of Patients with Nasopharyngeal Carcinoma.* Cell Biochem Biophys, 2015. **73**(3): p. 731-6.
334. Ch'ng, S., et al., *Prospective quality of life assessment between treatment groups for oral cavity squamous cell carcinoma.* Head Neck, 2014. **36**(6): p. 834-40.
335. Liao, K.-C., et al., *Quality of Life as a Mediator between Cancer Stage and Long-Term Mortality in Nasopharyngeal Cancer Patients Treated with Intensity-Modulated Radiotherapy.* Cancers, 2021. **13**(20): p. 5063.
336. Choby, G.W., et al., *Transoral robotic surgery alone for oropharyngeal cancer: quality-of-life outcomes.* JAMA Otolaryngol Head Neck Surg, 2015. **141**(6): p. 499-504.
337. Crombie, A.K., C.S. Farah, and M.D. Batstone, *Health-related quality of life of patients treated with primary chemoradiotherapy for oral cavity squamous cell carcinoma: a comparison with surgery.* Br J Oral Maxillofac Surg, 2014. **52**(2): p. 111-7.
338. Kang, D., et al., *Pre-treatment quality of life in patients with salivary gland cancer in comparison with those of head and neck cancer patients.* Qual Life Res, 2023. **32**(5): p. 1493-1506.
339. Kao, N.H., et al., *Early quality of life outcomes after surgery in head and neck cancer survivors with EORTC QLQ-C30 and EORTC QLQ-HN35 in an Asian tertiary centre.* Support Care Cancer, 2022. **30**(5): p. 4537-4546.
340. Burkholder, D., et al., *Effects of extended pleurectomy and decortication on quality of life and pulmonary function in patients with malignant pleural mesothelioma.* Ann Thorac Surg, 2015. **99**(5): p. 1775-80.
341. Lauk, O., et al., *Quality of Life Is Not Deteriorated After Extrapleural Pneumonectomy vs. (Extended) Pleurectomy/Decortication in Patients With Malignant Pleural Mesothelioma.* Front Surg, 2021. **8**: p. 766033.
342. Eberst, G., et al., *Health-Related Quality of Life Impact from Adding Bevacizumab to Cisplatin-Pemetrexed in Malignant Pleural Mesothelioma in the MAPS IFCT-GFPC-0701 Phase III Trial.* Clin Cancer Res, 2019. **25**(19): p. 5759-5765.

343. Scherpereel, A., et al., *First-line nivolumab plus ipilimumab versus chemotherapy for the treatment of unresectable malignant pleural mesothelioma: patient-reported outcomes in CheckMate 743.* Lung Cancer, 2022. **167**: p. 8-16.
344. Soldera, S.V., et al., *Favourable health-related quality of life reported in survivors of thymic malignancies.* Eur J Cardiothorac Surg, 2019. **55**(2): p. 292-299.
345. Mollberg, N.M., et al., *Quality of life after radical pleurectomy decortication for malignant pleural mesothelioma.* Ann Thorac Surg, 2012. **94**(4): p. 1086-92.
346. Moore, A., et al., *Malignant pleural mesothelioma: treatment patterns and humanistic burden of disease in Europe.* BMC Cancer, 2022. **22**(1): p. 693.
347. Rena, O. and C. Casadio, *Extrapleural pneumonectomy for early stage malignant pleural mesothelioma: a harmful procedure.* Lung Cancer, 2012. **77**(1): p. 151-5.
348. Tanaka, T., et al., *Physical function and health-related quality of life in patients undergoing surgical treatment for malignant pleural mesothelioma.* Support Care Cancer, 2017. **25**(8): p. 2569-2575.
349. Tanaka, T., et al., *Physical function and health-related quality of life in the convalescent phase in surgically treated patients with malignant pleural mesothelioma.* Support Care Cancer, 2019. **27**(11): p. 4107-4113.
350. Vigneswaran, W.T., et al., *Influence of Pleurectomy and Decortication in Health-Related Quality of Life Among Patients with Malignant Pleural Mesothelioma.* World J Surg, 2018. **42**(4): p. 1036-1045.
351. Nagamatsu, Y., et al., *Quality of life of survivors of malignant pleural mesothelioma in Japan: a cross sectional study.* BMC Cancer, 2018. **18**(1): p. 350.
352. Nakamichi, T., et al., *Quality of life and lung function after pleurectomy/decortication for malignant pleural mesothelioma.* Interact Cardiovasc Thorac Surg, 2021. **33**(4): p. 572-579.
353. Ambrogi, V., et al., *Clinical impact of extrapleural pneumonectomy for malignant pleural mesothelioma.* Ann Surg Oncol, 2012. **19**(5): p. 1692-9.
354. Arnold, D.T., et al., *The effect of chemotherapy on health-related quality of life in mesothelioma: results from the SWAMP trial.* Br J Cancer, 2015. **112**(7): p. 1183-9.
355. Granieri, A., et al., *Quality of life and personality traits in patients with malignant pleural mesothelioma and their first-degree caregivers.* Neuropsychiatr Dis Treat, 2013. **9**: p. 1193-202.
356. Kao, S.C., et al., *Health-related quality of life and inflammatory markers in malignant pleural mesothelioma.* Support Care Cancer, 2013. **21**(3): p. 697-705.
357. Kirby, R., et al., *Quality of life study following cytoreductive surgery and intraperitoneal chemotherapy for pseudomyxoma peritonei including redo procedures.* Int J Surg Oncol, 2013. **2013**: p. 461041.
358. Barker, C.A., et al., *Quality of Life Concerns in Patients with Uveal Melanoma after Initial Diagnosis.* Ocul Oncol Pathol, 2020. **6**(3): p. 184-195.
359. Bharmal, M., et al., *Health-related quality of life trajectory of treatment-naive patients with Merkel cell carcinoma receiving avelumab.* Future Oncol, 2020. **16**(27): p. 2089-2099.
360. Bharmal, M., et al., *Psychometric properties of the FACT-M questionnaire in patients with Merkel cell carcinoma.* Health Qual Life Outcomes, 2017. **15**(1): p. 247.
361. Bharmal, M., M. Hunger, and M. Schlichting, *Psychometric Properties of EQ-5D-5L Scoring Algorithms for the United Kingdom in Metastatic Merkel Cell Carcinoma.* Value Health, 2019. **22**(10): p. 1170-1177.
362. Bharmal, M., et al., *Update on the psychometric properties and minimal important difference (MID) thresholds of the FACT-M questionnaire for use in treatment-naive and previously treated patients with metastatic Merkel cell carcinoma.* Health Qual Life Outcomes, 2020. **18**(1): p. 145.
363. Brown, S.L., et al., *Prediction of all-cause mortality from 24 month trajectories in patient-reported psychological, clinical and quality of life outcomes in uveal melanoma patients.* J Behav Med, 2022. **45**(1): p. 115-123.
364. Damato, B., et al., *Patient-reported Outcomes and Quality of Life After Treatment of Choroidal Melanoma: A Comparison of Enucleation Versus Radiotherapy in 1596 Patients.* Am J Ophthalmol, 2018. **193**: p. 230-251.
365. Damato, B., et al., *Patient-Reported Outcomes and Quality of Life after Treatment for Choroidal Melanoma.* Ocul Oncol Pathol, 2019. **5**(6): p. 402-411.
366. Frenkel, S., et al., *Long-term uveal melanoma survivors: measuring their quality of life.* Acta Ophthalmol, 2018. **96**(4): p. e421-e426.
367. Lieb, M., et al., *Psychosocial impact of prognostic genetic testing in uveal melanoma patients: a controlled prospective clinical observational study.* BMC Psychol, 2020. **8**(1): p. 8.
368. Wiley, J.F., et al., *Quality of life and cancer-related needs in patients with choroidal melanoma.* Br J Ophthalmol, 2013. **97**(11): p. 1471-4.
369. Hope-Stone, L., et al., *Two-year patient-reported outcomes following treatment of uveal melanoma.* Eye (Lond), 2016. **30**(12): p. 1598-1605.
370. Hope-Stone, L., et al., *Comparison between patient-reported outcomes after enucleation and proton beam radiotherapy for uveal melanomas: a 2-year cohort study.* Eye, 2019. **33**(9): p. 1478-1484.
371. Scannell, O., et al., *Quality of Life in Uveal Melanoma Patients in Ireland: A Single-Centre Survey.* Ocul Oncol Pathol, 2020. **6**(2): p. 99-106.
372. Mouriaux, F., et al., *Sorafenib in metastatic uveal melanoma: efficacy, toxicity and health-related quality of life in a multicentre phase II study.* Br J Cancer, 2016. **115**(1): p. 20-4.
373. van Beek, J.G.M., et al., *Quality of life: fractionated stereotactic radiotherapy versus enucleation treatment in uveal melanoma patients.* Acta Ophthalmol, 2018. **96**(8): p. 841-848.
374. Vogl, T.J., et al., *Percutaneous Isolated Hepatic Perfusion as a Treatment for Isolated Hepatic Metastases of Uveal Melanoma: Patient Outcome and Safety in a Multi-centre Study.* Cardiovasc Intervent Radiol, 2017. **40**(6): p. 864-872.
375. Atkinson, T.M., et al., *Relationship between physician-adjudicated adverse events and patient-reported health-related quality of life in a phase II clinical trial (NCT01143402) of patients with metastatic uveal melanoma.* J Cancer Res Clin Oncol, 2017. **143**(3): p. 439-445.
376. Gollrad, J., et al., *Quality of life and treatment-related burden during ocular proton therapy: a prospective trial of 131 patients with uveal melanoma.* Radiat Oncol, 2021. **16**(1): p. 174.
377. Klingenstein, A., et al., *The national comprehensive cancer network distress thermometer as a screening tool for the evaluation of quality of life in uveal melanoma patients.* Acta Ophthalmol, 2020. **98**(3): p. e381-e387.
378. Kaufman, H.L., et al., *Nonprogression with avelumab treatment associated with gains in quality of life in metastatic Merkel cell carcinoma.* Future Oncol, 2018. **14**(3): p. 255-266.
379. Klingenstein, A., et al., *Quality of life in the follow-up of uveal melanoma patients after CyberKnife treatment.* Melanoma Res, 2013. **23**(6): p. 481-8.
380. Klingenstein, A., et al., *Quality of life in the follow-up of uveal melanoma patients after enucleation in comparison to CyberKnife treatment.* Graefes Arch Clin Exp Ophthalmol, 2016. **254**(5): p. 1005-12.
381. Kopp, B.C., R.T. Crump, and E. Weis, *The use of semistructured interviews to assess quality of life impacts for patients with uveal melanoma.* Can J Ophthalmol, 2017. **52**(2): p. 181-185.
382. Gollrad, J., et al., *Impact of Adjuvant Ocular Interventions on the Quality of Life of Patients with Uveal Melanoma after Proton Beam Therapy.* Ocul Oncol Pathol, 2022. **8**(2): p. 110-119.

383. Bitterlich, C. and D. Vordermark, *Analysis of health-related quality of life in patients with brain tumors prior and subsequent to radiotherapy.* Oncol Lett, 2017. **14**(2): p. 1841-1846.
384. Blonski, M., et al., *Combination of neoadjuvant chemotherapy followed by surgical resection as a new strategy for WHO grade II gliomas: a study of cognitive status and quality of life.* J Neurooncol, 2012. **106**(2): p. 353-66.
385. Boele, F.W., et al., *The association between cognitive functioning and health-related quality of life in low-grade glioma patients.* Neurooncol Pract, 2014. **1**(2): p. 40-46.
386. Boele, F.W., et al., *Health-related quality of life in stable, long-term survivors of low-grade glioma.* J Clin Oncol, 2015. **33**(9): p. 1023-9.
387. Boele, F.W., et al., *Long-term wellbeing and neurocognitive functioning of diffuse low-grade glioma patients and their caregivers: A longitudinal study spanning two decades.* Neuro Oncol, 2023. **25**(2): p. 351-364.
388. Bunevicius, A., et al., *Predictors of health-related quality of life in neurosurgical brain tumor patients: focus on patient-centered perspective.* Acta Neurochir (Wien), 2014. **156**(2): p. 367-74.
389. Bunevicius, A., *Reliability and validity of the SF-36 Health Survey Questionnaire in patients with brain tumors: a cross-sectional study.* Health Qual Life Outcomes, 2017. **15**(1): p. 92.
390. Buvarp, D., et al., *Preoperative Patient-Reported Outcomes in Suspected Low-Grade Glioma: Markers of Disease Severity and Correlations with Molecular Subtypes.* J Clin Med, 2021. **10**(4).
391. Umezaki, S., et al., *Factors associated with health-related quality of life in patients with glioma: impact of symptoms and implications for rehabilitation.* Jpn J Clin Oncol, 2020. **50**(9): p. 990-998.
392. Daigle, K., et al., *Effects of surgical resection on the evolution of quality of life in newly diagnosed patients with glioblastoma: a report on 19 patients surviving to follow-up.* Curr Med Res Opin, 2013. **29**(10): p. 1307-13.
393. Dirven, L., et al., *The impact of bevacizumab on health-related quality of life in patients treated for recurrent glioblastoma: results of the randomised controlled phase 2 BELOB trial.* Eur J Cancer, 2015. **51**(10): p. 1321-30.
394. Dirven, L., et al., *Impact of Radiation Target Volume on Health-Related Quality of Life in Patients With Low-Grade Glioma in the 2-Year Period Post Treatment: A Secondary Analysis of the EORTC 22033-26033.* Int J Radiat Oncol Biol Phys, 2019. **104**(1): p. 90-100.
395. Dirven, L., et al., *Neurocognitive functioning and health-related quality of life in adult medulloblastoma patients: long-term outcomes of the NOA-07 study.* J Neurooncol, 2020. **148**(1): p. 117-130.
396. Drewes, C., et al., *Quality of life in patients with intracranial tumors: does tumor laterality matter?* J Neurosurg, 2016. **125**(6): p. 1400-1407.
397. Drewes, C., et al., *Perioperative and Postoperative Quality of Life in Patients with Glioma-A Longitudinal Cohort Study.* World Neurosurg, 2018. **117**: p. e465-e474.
398. Dutzmann, S., et al., *A multi-center retrospective analysis of treatment effects and quality of life in adult patients with cranial ependymomas.* J Neurooncol, 2013. **114**(3): p. 319-27.
399. Field, K.M., et al., *Health-related quality of life outcomes from CABARET: a randomized phase 2 trial of carboplatin and bevacizumab in recurrent glioblastoma.* J Neurooncol, 2017. **133**(3): p. 623-631.
400. Flechl, B., et al., *Neurocognitive and sociodemographic functioning of glioblastoma long-term survivors.* J Neurooncol, 2012. **109**(2): p. 331-9.
401. Yavas, C., et al., *Health-related quality of life in high-grade glioma patients: a prospective single-center study.* Support Care Cancer, 2012. **20**(10): p. 2315-25.
402. Langegard, U., et al., *Evaluation of quality of care in relation to health-related quality of life of patients diagnosed with brain tumor: a novel clinic for proton beam therapy.* Support Care Cancer, 2019. **27**(7): p. 2679-2691.
403. Leonetti, A., et al., *Factors Influencing Mood Disorders and Health Related Quality of Life in Adults With Glioma: A Longitudinal Study.* Front Oncol, 2021. **11**: p. 662039.
404. Liu, B., et al., *Impact of neurosurgical enhanced recovery after surgery (ERAS) program on health-related quality of life in glioma patients: a secondary analysis of a randomized controlled trial.* J Neurooncol, 2020. **148**(3): p. 555-567.
405. Liu, Y., et al., *Improvement of health related quality of life in patients with recurrent glioma treated with bevacizumab plus daily temozolomide as the salvage therapy.* Clin Neurol Neurosurg, 2018. **169**: p. 64-70.
406. Lombardi, G., et al., *Quality of Life Perception, Cognitive Function, and Psychological Status in a Real-world Population of Glioblastoma Patients Treated With Radiotherapy and Temozolomide: A Single-center Prospective Study.* Am J Clin Oncol, 2018. **41**(12): p. 1263-1271.
407. Waddle, M.R., et al., *Impacts of Surgery on Symptom Burden and Quality of Life in Pituitary Tumor Patients in the Subacute Post-operative Period.* Front Oncol, 2019. **9**: p. 299.
408. Wang, Y., et al., *Comprehensive ability evaluation and trend analysis of patients with malignant intracranial tumors in the perisurgery period.* Brain Behav, 2021. **11**(11): p. e02192.
409. Watanabe, T., et al., *Characteristics of health-related quality of life and related factors in patients with brain tumors treated with rehabilitation therapy.* J Patient Rep Outcomes, 2022. **6**(1): p. 94.
410. Wefel, J.S., et al., *Neurocognitive, symptom, and health-related quality of life outcomes of a randomized trial of bevacizumab for newly diagnosed glioblastoma (NRG/RTOG 0825).* Neuro Oncol, 2021. **23**(7): p. 1125-1138.
411. Svedung Wettervik, T., et al., *Patient-reported quality of life in grade 2 and 3 gliomas after surgery, can we do more?* Clin Neurol Neurosurg, 2022. **214**: p. 107175.
412. Wolf, J., et al., *Evaluation of neuropsychological outcome and "quality of life" after glioma surgery.* Langenbecks Arch Surg, 2016. **401**(4): p. 541-9.
413. Habets, E.J., et al., *Health-related quality of life and cognitive functioning in long-term anaplastic oligodendroglioma and oligoastrocytoma survivors.* J Neurooncol, 2014. **116**(1): p. 161-8.
414. Hickmann, A.K., et al., *Evaluating patients for psychosocial distress and supportive care needs based on health-related quality of life in primary brain tumors: a prospective multicenter analysis of patients with gliomas in an outpatient setting.* J Neurooncol, 2017. **131**(1): p. 135-151.
415. Sagberg, L.M., O. Solheim, and A.S. Jakola, *Quality of survival the 1st year with glioblastoma: a longitudinal study of patient-reported quality of life.* J Neurosurg, 2016. **124**(4): p. 989-97.
416. Sagberg, L.M., et al., *Brain atlas for assessing the impact of tumor location on perioperative quality of life in patients with high-grade glioma: A prospective population-based cohort study.* Neuroimage Clin, 2019. **21**: p. 101658.
417. Scartoni, D., et al., *Proton therapy re-irradiation preserves health-related quality of life in large recurrent glioblastoma.* J Cancer Res Clin Oncol, 2020. **146**(6): p. 1615-1622.
418. Seekatz, B., et al., *Screening for symptom burden and supportive needs of patients with glioblastoma and brain metastases and their caregivers in relation to their use of specialized palliative care.* Supportive Care in Cancer, 2017. **25**(9): p. 2761-2770.

419. Solanki, C., et al., *Impairments in Quality of Life and Cognitive Functions in Long-term Survivors of Glioblastoma.* J Neurosci Rural Pract, 2017. **8**(2): p. 228-235.
420. Ståhl, P., et al., *Health-related quality of life and emotional well-being in patients with glioblastoma and their relatives.* Journal of Neuro-Oncology, 2020. **149**(2): p. 347-356.
421. Ståhl, P., et al., *Quality of life in patients with glioblastoma and their relatives.* Acta Neurologica Scandinavica, 2022. **146**(1): p. 82-91.
422. Stockelmaier, L., et al., *Therapy for Recurrent High-Grade Gliomas: Results of a Prospective Multicenter Study on Health-Related Quality of Life.* World Neurosurg, 2017. **102**: p. 383-399.
423. Kevin Suen, K.-F., et al., *Health-related quality of life of glioblastoma patients receiving post-operative concomitant chemoradiotherapy plus adjuvant chemotherapy: A longitudinal study.* Interdisciplinary Neurosurgery, 2021. **26**: p. 101339.
424. Maitre, P., et al., *Prospective Longitudinal Assessment of Quality of Life and Activities of Daily Living as Patient-Reported Outcome Measures in Recurrent/Progressive Glioma Treated with High-dose Salvage Re-irradiation.* Clinical Oncology, 2021. **33**(3): p. e155-e165.
425. Minniti, G., et al., *Health-related quality of life in elderly patients with newly diagnosed glioblastoma treated with short-course radiation therapy plus concomitant and adjuvant temozolomide.* Int J Radiat Oncol Biol Phys, 2013. **86**(2): p. 285-91.
426. Randazzo, D.M., et al., *Complementary and integrative health interventions and their association with health-related quality of life in the primary brain tumor population.* Complement Ther Clin Pract, 2019. **36**: p. 43-48.
427. Reddy, K., et al., *Prospective evaluation of health-related quality of life in patients with glioblastoma multiforme treated on a phase II trial of hypofractionated IMRT with temozolomide.* J Neurooncol, 2013. **114**(1): p. 111-6.
428. Reijneveld, J.C., et al., *Health-related quality of life in patients with high-risk low-grade glioma (EORTC 22033-26033): a randomised, open-label, phase 3 intergroup study.* Lancet Oncol, 2016. **17**(11): p. 1533-1542.
429. Renovanz, M., et al., *Supportive Care Needs in Glioma Patients and Their Caregivers in Clinical Practice: Results of a Multicenter Cross-Sectional Study.* Front Neurol, 2018. **9**: p. 763.
430. Renovanz, M., et al., *Health-related quality of life and distress in elderly vs. younger patients with high-grade glioma-results of a multicenter study.* Support Care Cancer, 2020. **28**(11): p. 5165-5175.
431. Rubin, M.C., et al., *Primary versus recurrent surgery for glioblastoma-a prospective cohort study.* Acta Neurochir (Wien), 2022. **164**(2): p. 429-438.
432. Taphoorn, M.J., et al., *Health-Related Quality of Life in a Randomized Phase III Study of Bevacizumab, Temozolomide, and Radiotherapy in Newly Diagnosed Glioblastoma.* J Clin Oncol, 2015. **33**(19): p. 2166-75.
433. Taphoorn, M.J.B., et al., *Influence of Treatment With Tumor-Treating Fields on Health-Related Quality of Life of Patients With Newly Diagnosed Glioblastoma: A Secondary Analysis of a Randomized Clinical Trial.* JAMA Oncol, 2018. **4**(4): p. 495-504.
434. Teng, K.X., et al., *Life after surgical resection of a low-grade glioma: A prospective cross-sectional study evaluating health-related quality of life.* J Clin Neurosci, 2021. **88**: p. 259-267.
435. Vaitkiene, P., et al., *Association of miR-34a Expression with Quality of Life of Glioblastoma Patients: A Prospective Study.* Cancers (Basel), 2019. **11**(3).
436. Valiulyte, I., et al., *Associations of miR-181a with Health-Related Quality of Life, Cognitive Functioning, and Clinical Data of Patients with Different Grade Glioma Tumors.* Int J Mol Sci, 2022. **23**(19).
437. Jakola, A.S., et al., *Postoperative deterioration in health related quality of life as predictor for survival in patients with glioblastoma: a prospective study.* PLoS One, 2011. **6**(12): p. e28592.
438. Jakola, A.S., et al., *Low Grade Gliomas in Eloquent Locations – Implications for Surgical Strategy, Survival and Long Term Quality of Life.* PLOS ONE, 2012. **7**(12): p. e51450.
439. Jakola, A.S., et al., *Surgical strategies in low-grade gliomas and implications for long-term quality of life.* J Clin Neurosci, 2014. **21**(8): p. 1304-9.
440. Jakola, A.S., et al., *The impact of resection in IDH-mutant WHO grade 2 gliomas: a retrospective population-based parallel cohort study.* J Neurosurg, 2022. **137**(5): p. 1321-1328.
441. Nickel, K., et al., *The patients' view: impact of the extent of resection, intraoperative imaging, and awake surgery on health-related quality of life in high-grade glioma patients-results of a multicenter cross-sectional study.* Neurosurg Rev, 2018. **41**(1): p. 207-219.
442. Noll, K.R., et al., *Relationships between neurocognitive functioning, mood, and quality of life in patients with temporal lobe glioma.* Psycho-Oncology, 2017. **26**(5): p. 617-624.
443. Okita, Y., et al., *Health-related quality of life in long-term survivors with Grade II gliomas: the contribution of disease recurrence and Karnofsky Performance Status.* Jpn J Clin Oncol, 2015. **45**(10): p. 906-13.
444. Aaronson, N.K., et al., *Compromised health-related quality of life in patients with low-grade glioma.* J Clin Oncol, 2011. **29**(33): p. 4430-5.
445. Ahn, G.S., et al., *Influence of Concurrent and Adjuvant Temozolomide on Health-Related Quality of Life of Patients with Grade III Gliomas: A Secondary Analysis of a Randomized Clinical Trial (KNOG-1101 Study).* Cancer Res Treat, 2022. **54**(2): p. 396-405.
446. Yavas, C., et al., *Prospective assessment of health-related quality of life in patients with low-grade glioma: a single-center experience.* Support Care Cancer, 2012. **20**(8): p. 1859-68.
447. Armstrong, T.S., et al., *Net clinical benefit analysis of radiation therapy oncology group 0525: a phase III trial comparing conventional adjuvant temozolomide with dose-intensive temozolomide in patients with newly diagnosed glioblastoma.* J Clin Oncol, 2013. **31**(32): p. 4076-84.
448. Zhu, J.J., et al., *Health-related quality of life, cognitive screening, and functional status in a randomized phase III trial (EF-14) of tumor treating fields with temozolomide compared to temozolomide alone in newly diagnosed glioblastoma.* J Neurooncol, 2017. **135**(3): p. 545-552.
449. Wick, W., et al., *Longitudinal analysis of quality of life following treatment with Asunercept plus reirradiation versus reirradiation in progressive glioblastoma patients.* J Neurooncol, 2019. **145**(3): p. 531-540.
450. Gabel, N., et al., *Health Related Quality of Life in Adult Low and High-Grade Glioma Patients Using the National Institutes of Health Patient Reported Outcomes Measurement Information System (PROMIS) and Neuro-QOL Assessments.* Front Neurol, 2019. **10**: p. 212.
451. Giovagnoli, A.R., et al., *Quality of life and brain tumors: what beyond the clinical burden?* J Neurol, 2014. **261**(5): p. 894-904.
452. Palmer, J.D., et al., *Health-Related Quality of Life for Patients Receiving Tumor Treating Fields for Glioblastoma.* Front Oncol, 2021. **11**: p. 772261.

453. Park, D.Y., et al., *Quality of life following concurrent temozolomide-based chemoradiation therapy or observation in low-grade glioma.* J Neurooncol, 2022. **156**(3): p. 499-507.
454. Peters, K.B., et al., *Impact of health-related quality of life and fatigue on survival of recurrent high-grade glioma patients.* J Neurooncol, 2014. **120**(3): p. 499-506.
455. Peters, K.B., et al., *Effects of low-dose naltrexone on quality of life in high-grade glioma patients: a placebo-controlled, double-blind randomized trial.* Support Care Cancer, 2022. **30**(4): p. 3463-3471.
456. Piil, K., et al., *Health-related quality of life in patients with high-grade gliomas: a quantitative longitudinal study.* J Neurooncol, 2015. **124**(2): p. 185-95.
457. Piil, K., et al., *Health-related quality of life and caregiver perspectives in glioblastoma survivors: a mixed-methods study.* BMJ Support Palliat Care, 2022. **12**(e6): p. e846-e854.
458. Pollom, E.L., et al., *Phase 1/2 Trial of 5-Fraction Stereotactic Radiosurgery With 5-mm Margins With Concurrent and Adjuvant Temozolomide in Newly Diagnosed Supratentorial Glioblastoma: Health-Related Quality of Life Results.* Int J Radiat Oncol Biol Phys, 2017. **98**(1): p. 123-130.
459. Porter, K.R., et al., *Assessment of clinical and nonclinical characteristics associated with health-related quality of life in patients with high-grade gliomas: a feasibility study.* Support Care Cancer, 2014. **22**(5): p. 1349-62.
460. Clement, P.M.J., et al., *Impact of depatuxizumab mafodotin on health-related quality of life and neurological functioning in the phase II EORTC 1410/INTELLANCE 2 trial for EGFR-amplified recurrent glioblastoma.* Eur J Cancer, 2021. **147**: p. 1-12.
461. Coomans, M.B., et al., *Factors associated with health-related quality of life (HRQoL) deterioration in glioma patients during the progression-free survival period.* Neuro Oncol, 2022. **24**(12): p. 2159-2169.
462. Kaminska, M., et al., *Quality of life in patients with brain tumors in the course of alpha therapy*. J Psychosoc Oncol, 2017. **35**(5): p. 631-644.
