# Supplementary material for: Health-Related Quality of Life (HRQoL) Assessments in Research on Patients with Adult Rare Solid Cancers: A State-of-the-Art Review
Source: Cancers (Basel). 2025 Jan 24;17(3):387. doi: 10.3390/cancers17030387 (PMC11816368; doi:10.3390/cancers17030387)
Supplement: Supplementary file 1 [file cancers-17-00387-s001.zip › Supplementary File1_ search string.pdf]

Numbers FLOW chart:

Domain 0

Database	Numbers
Medline via Ovid	1066
Embase.com	1127
Scopus	891
**Total**	3084
Duplicates	1731
**Unique references**	1353

Domain 1

Database	Numbers
Medline via Ovid	1792
Embase.com	2612
Scopus	1815
**Total**	6219
Duplicates	3124
**Unique references**	3095

Domain 2

Database	Numbers
Medline via Ovid	437
Embase.com	1104
Scopus	386
**Total**	1927
Duplicates	671
**Unique references**	1256

Domain 3

Database	Numbers
Medline via Ovid	379
Embase.com	614
Scopus	308
**Total**	1301
Duplicates	535
**Unique references**	766

Domain 4

Database	Numbers
Medline via Ovid	526
Embase.com	890
Scopus	520
**Total**	1936
Duplicates	898

**Unique references**	1038

Domain 5

Database	Numbers
Medline via Ovid	3242
Embase.com	4307
Scopus	3128
**Total**	10677
Duplicates	5124
**Unique references**	5553

Domain 6

Database	Numbers
Medline via Ovid	1065
Embase.com	1333
Scopus	1050
**Total**	3448
Duplicates	1840
**Unique references**	1608

Domain 7

Database	Numbers
Medline via Ovid	1164
Embase.com	1532
Scopus	1195
**Total**	3891
Duplicates	1963
**Unique references**	1928

Domain 8

Database	Numbers
Medline via Ovid	555
Embase.com	855
Scopus	569
**Total**	1979
Duplicates	997
**Unique references**	982

Domain 9

Database	Numbers
Medline via Ovid	245
Embase.com	539
Scopus	268
**Total**	1052
Duplicates	441

**Unique references**	611

Domain 10

Database	Numbers
Medline via Ovid	2128
Embase.com	2587
Scopus	2137
**Total**	6852
Duplicates	3882
**Unique references**	2970

Search history:

Medline via Ovid:

Domain 0 – General Terms

1	"rare diseases"/ and exp neoplasms/	3971
2	(rare adj5 (cancer? or tumor* or tumour* or malignan* or neoplas*)).ti,ab,kf.	95552
3	or/1-2 [Domain 0 - General terms]	98245
4	*"quality of life"/ or ("Quality Of Life" or HRQOL or QOL).ti,ab,kf. [QoL]	385875
5	3 and 4 [Domain 0 AND QoL]	1063

Domain 1 – Sarcoma

1	exp sarcoma/	149050
2	sarcoma?.ti,ab,kf.	105774
3	adenosarcoma/	377
4	adenosarcoma?.ti,ab,kf.	646
5	carcinosarcoma/	3397
6	carcinosarcoma?.ti,ab,kf.	4713
7	"desmoplastic small round cell tumor"/	240
8	"desmoplastic small round cell tumo?r?".ti,ab,kf.	778
9	"desmoplastic small cell tumo?r?".ti,ab,kf.	52
10	DSRCT?.ti,ab,kf.	405
11	"endometrial stromal tumor"/	192
12	"endometrial stromal tumo?r?".ti,ab,kf.	206
13	"endolymphatic stromal myos?s".ti,ab,kf.	59
14	fibrosarcoma/	12358
15	fibrosarcoma?.ti,ab,kf.	12071
16	dermatofibrosarcoma/	1458
17	dermatofibrosarcoma?.ti,ab,kf.	2242
18	DFSP.ti,ab,kf.	880
19	neurofibrosarcoma/	414
20	neurofibrosarcoma?.ti,ab,kf.	425
21	hemangiosarcoma/	7603
22	hemangiosarcoma?.ti,ab,kf.	1212
23	angiosarcoma?.ti,ab,kf.	7221
24	"histiocytoma, malignant fibrous"/	990
25	"malignant fibrous histiocytoma?".ti,ab,kf.	4184

26	"malignant fibrohistiocytic tumo?r?".ti,ab,kf.	16
27	leiomyosarcoma/	9284
28	leiomyosarcoma?.ti,ab,kf.	11433
29	exp liposarcoma/	5376
30	liposarcoma?.ti,ab,kf.	7512
31	"atypical lipomatous tumo?r?".ti,ab,kf.	390
32	lymphangiosarcoma?.ti,ab,kf.	328
33	"mixed tumor, mesodermal"/	122
34	"mesodermal mixed tumo?r?".ti,ab,kf.	98
35	myosarcoma/	326
36	myosarcoma?.ti,ab,kf.	226
37	exp rhabdomyosarcoma/	11220
38	rhabdomyosarcoma?.ti,ab,kf.	13133
39	myxosarcoma/	431
40	myxosarcoma?.ti,ab,kf.	265
41	"phyllodes tumor"/	2038
42	"phyllodes tumo?r?".ti,ab,kf.	1795
43	fibromyxosarcoma?.ti,ab,kf.	55
44	gliosarcoma?.ti,ab,kf.	1227
45	leukosarcoma?.ti,ab,kf.	129
46	"stewart-treves syndrome".ti,ab,kf.	264
47	lymphosarcoma?.ti,ab,kf.	5166
48	reticulosarcoma?.ti,ab,kf.	1159
49	"rhabdoid tumo?r?".ti,ab,kf.	2393
50	"fibroblastic sarcoma?".ti,ab,kf.	186
51	"hemangioendothelioma, epithelioid"/	1122
52	"epithelioid hemangioendothelioma?".ti,ab,kf.	1335
53	"malignant peripheral nerve sheath tumo?r?".ti,ab,kf.	2797
54	glomangiosarcoma?.ti,ab,kf.	46
55	"malignant glomus tumo?r?".ti,ab,kf.	120
56	"perivascular epithelioid cell neoplasms"/	528
57	"perivascular epithelioid cell tumo?r?".ti,ab,kf.	658
58	PEComa?.ti,ab,kf.	818
59	"ewing sarcoma"/	7665
60	"ewing sarcoma?".ti,ab,kf.	4581
61	ESFT.ti,ab,kf.	314
62	"neuroectodermal tumors, primitive, peripheral"/	1746
63	"peripheral neuroectodermal tumo?r?".ti,ab,kf.	242
64	"primitive neuroectodermal tumo?r?".ti,ab,kf.	3572
65	pPNET.ti,ab,kf.	244
66	medulloepithelioma?.ti,ab,kf.	396
67	"gastrointestinal stromal tumors"/	7533
68	((gastrointestinal or gastric or GI) adj1 stroma? adj1 (tumo?r? or neoplas* or sarcoma?)).ti,ab,kf.	11306
69	GIST?.ti,ab,kf.	9453
70	"carney triad?".ti,ab,kf.	124
71	"Carney-Stratakis syndrome".ti,ab,kf.	63
72	"Carney-Stratakis dyad?".ti,ab,kf.	3
73	exp osteosarcoma/	31784
74	osteosarcoma?.ti,ab,kf.	28201

75	"osteogenic sarcoma?".ti,ab,kf.	2773
76	"bone sarcoma?".ti,ab,kf.	1969
77	exp chondrosarcoma/	7464
78	chondrosarcoma?.ti,ab,kf.	8897
79	"giant cell tumor of bone"/	2113
80	("giant cell tumo?r?" adj3 bone?).ti,ab,kf.	2516
81	GCTB.ti,ab,kf.	448
82	osteoclastoma?.ti,ab,kf.	382
83	chordoma/	3797
84	chordoma?.ti,ab,kf.	4663
85	"notochordal sarcoma?".ti,ab,kf.	3
86	or/1-85 [Domain 1 - Sarcomas]	250921
87	*"quality of life"/ or ("Quality Of Life" or HRQOL or QOL).ti,ab,kf. [QoL]	385875
88	86 and 87 [Domain 1 AND QoL]	1784

Domain 2 - Female genital organs and placenta

1	(rare adj3 ("female genital" or fallopian or uterine or vaginal or vulvar) adj3 (cancer? or tumo?r? or malignan*)).ti,ab,kf.	432
2	("non epithelial" adj2 (ovarian or ovary) adj2 (cancer? or tumo?r? or malignan*)).ti,ab,kf.	82
3	exp "sex cord-gonadal stromal tumors"/	7030
4	"sex cord stromal tumo?r?".ti,ab,kf.	1069
5	("sex cord tumo?r?" adj3 ovar*).ti,ab,kf.	282
6	SCST.ti,ab,kf.	134
7	SCCO.ti,ab,kf.	228
8	SCCOHT.ti,ab,kf.	102
9	dysgerminoma/	5662
10	dysgerminoma?.ti,ab,kf.	1465
11	"endodermal sinus tumor"/	1115
12	"endodermal sinus tumo?r?".ti,ab,kf.	834
13	"yolk sac tumo?r?".ti,ab,kf.	2214
14	orchioblastoma?.ti,ab,kf.	29
15	"carcinoma, embryonal"/	1164
16	((embryo or embryon*) adj1 (carcinoma? or adenocarcinoma? or "cell cancer?")).ti,ab,kf.	5224
17	"choriocarcinoma, non-gestational"/	138
18	"non-gestational choriocarcinoma?".ti,ab,kf.	61
19	"nongestational choriocarcinoma?".ti,ab,kf.	82
20	teratoma/	17193
21	"mature teratoma?".ti,ab,kf.	1899
22	teratosarcoma?.ti,ab,kf.	1
23	"immature teratoma?".ti,ab,kf.	1330
24	"mixed germ cell tumo?r?".ti,ab,kf.	816
25	exp fibroma/	17786
26	fibroma?.ti,ab,kf.	9837
27	thecoma/	938
28	thecoma?.ti,ab,kf.	456
29	hemangiofibroma?.ti,ab,kf.	16
30	exp fibrosarcoma/	14095

31	fibrosarcoma?.ti,ab,kf.	12071
32	"fibroblastic sarcoma".ti,ab,kf.	176
33	"sclerosing stromal tumo?r?".ti,ab,kf.	209
34	"signet ring stromal tumo?r?".ti,ab,kf.	36
35	"microcystic stromal tumo?r?".ti,ab,kf.	49
36	"leydig cell tumor"/	1957
37	"leydig cell tumo?r?".ti,ab,kf.	1814
38	"steroid cell tumo?r?".ti,ab,kf.	204
39	"granulosa cell tumor"/	2471
40	"granulosa cell tumo?r?".ti,ab,kf.	2519
41	"granulosa cell carcinoma?".ti,ab,kf.	33
42	"granulosa cancer?".ti,ab,kf.	9
43	"sertoli cell tumor"/	847
44	"sertoli cell tumo?r?".ti,ab,kf.	755
45	"sertoli-leydig cell tumor"/	850
46	"sertoli-leydig cell tumo?r?".ti,ab,kf.	564
47	androblastoma?.ti,ab,kf.	97
48	"carcinoma, endometrioid"/ and exp uterus/	491
49	"endometrioid adenocarcinoma?".ti,ab,kf. and exp uterus/	289
50	("endometrioid adenocarcinoma?" adj3 uter*).ti,ab,kf.	218
51	"carcinoma, adenosquamous"/ and exp uterus/	80
52	("adenosquamous carcinoma?" adj3 uter*).ti,ab,kf.	68
53	"adenocarcinoma, mucinous"/ and exp uterus/	96
54	("mucinous adenocarcinoma?" adj3 uter*).ti,ab,kf.	23
55	"mixed cell adenocarcinoma?".ti,ab,kf. and exp uterus/	0
56	("mixed cell adenocarcinoma?" adj3 uter*).ti,ab,kf.	0
57	"villous adenocarcinoma?".ti,ab,kf.	22
58	"brenner tumor"/	672
59	"brenner tumo?r?".ti,ab,kf.	710
60	"carcinoma, transitional cell"/ and exp uterus/	19
61	("transitional cell carcinoma?" adj3 uter*).ti,ab,kf.	13
62	"basaloid carcinoma?".ti,ab,kf. and exp uterus/	2
63	("basaloid carcinoma?" adj3 uter*).ti,ab,kf.	2
64	exp "carcinoma, squamous cell"/ and exp uterus/	2011
65	(("squamous cell carcinoma?" or SCC) adj3 uter*).ti,ab,kf.	1103
66	"carcinoma, adenoid cystic"/ and exp uterus/	28
67	("adenoid cystic carcinoma?" adj3 uter*).ti,ab,kf.	27
68	"adenocarcinoma, clear cell"/ and exp uterus/	137
69	("clear cell adenocarcinoma?" adj3 uter*).ti,ab,kf.	40
70	(serous adj3 carcinoma?).ti,ab,kf.	5498
71	"mixed tumor, mullerian"/	530
72	"mullerian mixed tumo?r?".ti,ab,kf.	81
73	exp "carcinoma, squamous cell"/ and "cervix uteri"/	1704
74	(("squamous cell carcinoma?" or SCC) adj3 cervi*).ti,ab,kf.	3927
75	CSCC.ti,ab,kf.	1727
76	"Carcinoma, Verrucous"/ and "cervix uteri"/	2
77	((verrucous or warty) adj1 carcinoma? adj3 cervi*).ti,ab,kf.	28
78	"basaloid carcinoma?".ti,ab,kf. and "cervix uteri"/	2
79	("basaloid carcinoma?" adj3 cervi*).ti,ab,kf.	4
80	("spindle cell" adj3 carcinoma?).ti,ab,kf. and "cervix uteri"/	0

81	("spindle cell" adj3 carcinoma? adj3 cervi*).ti,ab,kf.	2
82	"lymphoepithelial carcinoma?".ti,ab,kf. and "cervix uteri"/	0
83	("lymphoepithelial carcinoma?" adj3 cervi*).ti,ab,kf.	3
84	"carcinoma, transitional cell"/ and "cervix uteri"/	10
85	("transitional cell carcinoma?" adj3 cervi*).ti,ab,kf.	7
86	"glassy cell carcinoma of the cervix".rs.	12
87	"glassy cell carcinoma?".ti,ab,kf. and "cervix uteri"/	11
88	("glassy cell carcinoma?" adj3 cervi*).ti,ab,kf.	44
89	exp adenocarcinoma/ and "cervix uteri"/	1231
90	(adenocarcinoma? adj3 cervi*).ti,ab,kf.	2927
91	"cystadenocarcinoma, serous"/ and "cervix uteri"/	12
92	("serous cystadenocarcinoma?" adj3 cervi*).ti,ab,kf.	1
93	"undifferentiated carcinoma?".ti,ab,kf. and "cervix uteri"/	9
94	("undifferentiated carcinoma?" adj3 cervi*).ti,ab,kf.	22
95	"carcinoma, signet ring cell"/ and "cervix uteri"/	4
96	("signet ring cell carcinoma?" and cervi*).ti,ab,kf.	48
97	mesonephroma/ and "cervix uteri"/	23
98	(mesonephroma? and cervi*).ti,ab,kf.	16
99	exp adenocarcinoma/ and ovary/	1414
100	(adenocarcinoma? adj3 ovar*).ti,ab,kf.	2792
101	"cystadenocarcinoma, serous"/ and ovary/	243
102	("serous cystadenocarcinoma?" adj3 ovar*).ti,ab,kf.	368
103	(endometrioid* adj1 adenofibroma?).ti,ab,kf. and ovary/	1
104	(endometrioid* adj1 adenofibroma? adj3 ovar*).ti,ab,kf.	5
105	adenocarcinofibroma?.ti,ab,kf.	4
106	"carcinoma, transitional cell"/ and ovary/	14
107	("transitional cell carcinoma?" adj3 ovar*).ti,ab,kf.	69
108	"basaloid carcinoma?".ti,ab,kf. and ovary/	0
109	("basaloid carcinoma?" adj3 ovar*).ti,ab,kf.	3
110	"adenocarcinoma, mucinous"/ and ovary/	165
111	("mucinous adenocarcinoma?" adj3 ovar*).ti,ab,kf.	143
112	"adenocarcinoma, clear cell"/ and ovary/	105
113	("clear cell adenocarcinoma?" adj3 ovar*).ti,ab,kf.	205
114	(peritoneal adj3 (serous or papillary) adj3 carcinoma?).ti,ab,kf.	294
115	exp adenocarcinoma/ and "fallopian tubes"/	519
116	(adenocarcinoma? adj3 ("fallopian tube?" or oviduct?)).ti,ab,kf.	182
117	"cystadenocarcinoma, serous"/ and "fallopian tubes"/	177
118	("serous cystadenocarcinoma?" adj3 ("fallopian tube?" or oviduct?)).ti,ab,kf.	0
119	"struma ovarii"/	586
120	"struma ovarii".ti,ab,kf.	649
121	"neoplasms, germ cell and embryonal"/ and ovary/	173
122	("germ cell tumo?r?" adj3 ovar*).ti,ab,kf.	936
123	"gestational trophoblastic disease"/	1188
124	"gestational trophoblastic disease?".ti,ab,kf.	1811
125	GTD.ti,ab,kf.	731
126	"gestational trophoblastic neoplas*".ti,ab,kf.	1240
127	"invasive mole?".ti,ab,kf.	527
128	exp "hydatidiform mole"/	5238
129	"hydatidiform mole?".ti,ab,kf.	3724
130	"molar pregnanc*".ti,ab,kf.	1480

131	"partial mole?".ti,ab,kf.	526
132	exp choriocarcinoma/	7526
133	choriocarcinoma?.ti,ab,kf.	7427
134	"placental site trophoblastic tumo?r?".ti,ab,kf.	563
135	PSTT.ti,ab,kf.	268
136	"epithelioid trophoblastic tumo?r?".ti,ab,kf.	252
137	polyembryoma?.ti,ab,kf. and ovary/	3
138	(polyembryoma? adj3 ovar*).ti,ab,kf.	4
139	"epithelial tumo?r?".ti,ab,kf. and (vulva/ or vagina/)	11
140	("epithelial tumo?r?" adj3 (vulva or vagina?)).ti,ab,kf.	6
141	exp "carcinoma, squamous cell"/ and (vulva/ or vagina/)	630
142	("squamous cell carcinoma?" adj3 (vulva or vagina?)).ti,ab,kf.	750
143	adenocarcinoma/ and (vulva/ or vagina/)	377
144	(adenocarcinoma? adj3 (vulva or vagina?)).ti,ab,kf.	473
145	"paget disease, extramammary"/ and (vulva/ or vagina/)	135
146	("paget disease" adj3 (vulva or vagina?)).ti,ab,kf.	47
147	"undifferentiated carcinoma?".ti,ab,kf. and (vulva/ or vagina/)	2
148	("undifferentiated carcinoma?" adj3 (vulva or vagina?)).ti,ab,kf.	4
149	or/1-148 [Domain 2 - Female genital organs and placenta]	117645
150	*"quality of life"/ or ("Quality Of Life" or HRQOL or QOL).ti,ab,kf. [QoL]	385875
151	149 and 150 [domain 2 AND QoL]	437

Domain 3 - Male genital organs and the urinary tract

1	(rare adj3 ("male genital" or testicular or "urinary tract" or penile or prostat*) adj3 (cancer? or tumo?r? or malignan*)).ti,ab,kf.	739
2	"carcinoma, acinar cell"/ and prostate/	39
3	("acinar cell carcinoma?" adj3 prostate?).ti,ab,kf.	0
4	"adenocarcinoma, mucinous"/ and prostate/	38
5	("mucinous adenocarcinoma?" adj3 prostate?).ti,ab,kf.	71
6	"carcinoma, signet ring cell"/ and prostate/	4
7	("signet ring cell carcinoma?" and prostate?).ti,ab,kf.	72
8	(adenocarcinoma? adj3 "neuroendocrine differentiation").ti,ab,kf. and prostate/	7
9	(adenocarcinoma? adj3 "neuroendocrine differentiation" adj3 prostate?).ti,ab,kf.	23
10	"oxyphilic adenocarcinoma?".ti,ab,kf. and prostate/	0
11	("oxyphilic adenocarcinoma?" adj3 prostate?).ti,ab,kf.	0
12	("spindle cell" adj3 carcinoma?).ti,ab,kf. and prostate/	0
13	("spindle cell" adj3 carcinoma? adj3 prostate?).ti,ab,kf.	2
14	"lymphoepithelial carcinoma?".ti,ab,kf. and prostate/	0
15	("lymphoepithelial carcinoma?" adj3 prostate?).ti,ab,kf.	0
16	exp "carcinoma, squamous cell"/ and prostate/	50
17	("squamous cell carcinoma?" adj3 prostate?).ti,ab,kf.	154
18	("squamous carcinoma?" adj3 prostate?).ti,ab,kf.	12
19	"carcinoma, adenosquamous"/ and prostate/	5
20	("adenosquamous carcinoma?" adj3 prostate?).ti,ab,kf.	24
21	"basaloid carcinoma?".ti,ab,kf. and prostate/	3
22	("basaloid carcinoma?" adj3 prostate?).ti,ab,kf.	6
23	"infiltrating duct carcinoma?".ti,ab,kf. and prostate/	1
24	("infiltrating duct carcinoma?" adj3 prostate?).ti,ab,kf.	0
25	"cribriform carcinoma?".ti,ab,kf. and prostate/	7

26	("cribriform carcinoma?" adj3 prostate?).ti,ab,kf.	6
27	"solid carcinoma?".ti,ab,kf. and prostate/	2
28	("solid carcinoma?" adj3 prostate?).ti,ab,kf.	0
29	"adenocarcinoma, papillary"/ and prostate/	3
30	("papillary adenocarcinoma?" adj3 prostate).ti,ab,kf.	15
31	"carcinoma, transitional cell"/ and prostate/	138
32	("transitional cell carcinoma?" adj3 prostate?).ti,ab,kf.	158
33	"basal cell adenocarcinoma?".ti,ab,kf. and prostate/	0
34	("basal cell adenocarcinoma?" adj3 prostate?).ti,ab,kf.	0
35	"carcinoma, adenoid cystic"/ and prostate/	10
36	("adenoid cystic carcinoma?" adj3 prostate?).ti,ab,kf.	30
37	"paratesticular adenocarcinoma?".ti,ab,kf.	2
38	"endometrioid adenocarcinoma?".ti,ab,kf. and prostate/	2
39	("endometrioid adenocarcinoma?" adj3 prostate?).ti,ab,kf.	11
40	"adenocarcinoma, clear cell"/ and prostate/	8
41	("clear cell adenocarcinoma?" adj3 prostate?).ti,ab,kf.	5
42	"cystadenocarcinoma, serous"/ and prostate/	0
43	("serous cystadenocarcinoma?" adj3 prostate?).ti,ab,kf.	0
44	"adenocarcinoma, mucinous"/ and prostate/	38
45	("mucinous adenocarcinoma?" adj3 prostate?).ti,ab,kf.	71
46	"rete testis"/	430
47	"rete testis".ti,ab,kf.	1119
48	"collecting duct carcinoma?".ti,ab,kf. and prostate/	0
49	("collecting duct carcinoma?" adj3 prostate?).ti,ab,kf.	0
50	seminoma/	3239
51	seminoma*.ti,ab,kf.	7893
52	"non-seminoma*".ti,ab,kf.	1981
53	"neoplasms, germ cell and embryonal"/ and "testicular neoplasms"/	4933
54	"testicular germ cell tumo?r?".ti,ab,kf.	3023
55	"mixed germ cell tumo?r?".ti,ab,kf.	816
56	teratocarcinoma/	733
57	teratocarcinoma?.ti,ab,kf.	2596
58	"malignant teratoma?".ti,ab,kf.	666
59	"sex cord cancer?".ti,ab,kf. and exp testis/	0
60	("sex cord cancer?" adj3 (testes or testi*)).ti,ab,kf.	0
61	"gonadal stromal tumo?r?".ti,ab,kf. and exp testis/	19
62	("gonadal stromal tumo?r?" adj3 (testes or testi*)).ti,ab,kf.	27
63	"sertoli cell tumor"/	847
64	"Sertoli cell carcinoma?".ti,ab,kf.	4
65	"epithelial tumo?r?".ti,ab,kf. and exp penis/	7
66	("epithelial tumo?r?" adj3 peni*).ti,ab,kf.	3
67	exp "carcinoma, squamous cell"/ and exp penis/	466
68	(("squamous cell carcinoma?" or SCC) adj3 peni*).ti,ab,kf.	1080
69	"carcinoma, adenosquamous"/ and exp penis/	2
70	("adenosquamous carcinoma?" adj3 peni*).ti,ab,kf.	7
71	"Carcinoma, Verrucous"/ and exp penis/	16
72	("verrucous carcinoma?" adj3 peni*).ti,ab,kf.	89
73	"basaloid carcinoma?".ti,ab,kf. and exp penis/	3
74	("basaloid carcinoma?" adj3 peni*).ti,ab,kf.	3
75	exp adenocarcinoma/ and exp penis/	348

76	(adenocarcinoma? adj3 peni*).ti,ab,kf.	41
77	"paget disease, extramammary"/ and exp penis/	46
78	("paget disease" adj3 peni*).ti,ab,kf.	4
79	"mixed tumo?r?".ti,ab,kf. and exp penis/	2
80	("mixed tumo?r?" adj3 peni*).ti,ab,kf.	1
81	"renal cell adenocarcinoma?".ti,ab,kf.	126
82	"adenocarcinoma, clear cell"/ and exp kidney/	105
83	("clear cell adenocarcinoma?" adj3 (kidney or renal)).ti,ab,kf.	36
84	"adenocarcinoma, papillary"/ and exp kidney/	48
85	("papillary adenocarcinoma?" adj3 (kidney or renal)).ti,ab,kf.	31
86	("renal cell carcinoma?" adj3 chromophobe?).ti,ab,kf.	938
87	"collecting duct carcinoma?".ti,ab,kf. and exp kidney/	148
88	("collecting duct carcinoma?" adj3 (kidney or renal)).ti,ab,kf.	166
89	(xp* adj3 "translocation carcinoma?").ti,ab,kf.	31
90	carcinoma/ and neuroblastoma/	154
91	(carcinoma? adj3 neuroblastoma?).ti,ab,kf.	421
92	"carcinoma, medullary"/ and exp kidney/	25
93	("medullary carcinoma?" adj3 (kidney or renal)).ti,ab,kf.	234
94	(tubular adj3 carcinoma? adj3 (kidney or renal)).ti,ab,kf.	71
95	"spindle cell carcinoma?".ti,ab,kf. and exp kidney/	38
96	("spindle cell carcinoma?" adj3 (kidney or renal)).ti,ab,kf.	86
97	exp "Carcinoma, Squamous Cell"/ adj3 exp kidney/	647
98	("squamous cell carcinoma?" adj3 kidney).ti,ab,kf.	109
99	("epithelial tumo?r?" adj3 ("renal pelvis" or ureter?)).ti,ab,kf.	18
100	"epithelial tumo?r?".ti,ab,kf. and ("kidney pelvis"/ or ureter/)	33
101	exp carcinoma/ and ("kidney pelvis"/ or ureter/)	2941
102	(carcinoma? adj3 ("kindey pelvis" or "renal pelvis" or ureter?)).ti,ab,kf.	1400
103	"carcinoma, squamous cell"/ and ("kidney pelvis"/ or ureter/)	344
104	("squamous cell carcinoma?" adj3 ("kindey pelvis" or "renal pelvis" or ureter?)).ti,ab,kf.	149
105	"basaloid carcinoma?".ti,ab,kf. and ("kidney pelvis"/ or ureter/)	0
106	("basaloid carcinoma?" adj3 ("kindey pelvis" or "renal pelvis" or ureter?)).ti,ab,kf.	0
107	"Carcinoma, Verrucous"/ and ("kidney pelvis"/ or ureter/)	3
108	("verrucous carcinoma?" adj3 ("kindey pelvis" or "renal pelvis" or ureter?)).ti,ab,kf.	3
109	exp adenocarcinoma/ and ("kidney pelvis"/ or ureter/)	693
110	(adenocarcinoma? adj3 ("kindey pelvis" or "renal pelvis" or ureter?)).ti,ab,kf.	152
111	"carcinoma, signet ring cell"/ and ("kidney pelvis"/ or ureter/)	6
112	("signet ring cell carcinoma?" and ("kindey pelvis" or "renal pelvis" or ureter?)).ti,ab,kf.	17
113	"carcinoma, adenoid cystic"/ and ("kidney pelvis"/ or ureter/)	1
114	("adenoid cystic carcinoma?" adj3 ("kindey pelvis" or "renal pelvis" or ureter?)).ti,ab,kf.	0
115	"epithelial tumo?r?".ti,ab,kf. adj3 (urethra?.ti,ab,kf. or urethra/)	6
116	exp carcinoma/ and urethra/	704
117	(carcinoma? adj3 urethra?).ti,ab,kf.	572
118	("squamous cell carcinoma?" adj3 urethra?).ti,ab,kf.	59
119	"basaloid carcinoma?".ti,ab,kf. and urethra/	0
120	("basaloid carcinoma?" adj3 urethra?).ti,ab,kf.	0
121	"Carcinoma, Verrucous"/ and urethra/	2
122	("verrucous carcinoma?" adj3 urethra?).ti,ab,kf.	0

123	exp adenocarcinoma/ and urethra/	204
124	(adenocarcinoma? adj3 urethra?).ti,ab,kf.	171
125	"carcinoma, signet ring cell"/ and urethra/	1
126	("signet ring cell carcinoma?" and urethra?).ti,ab,kf.	7
127	"carcinoma, adenoid cystic"/ and urethra/	0
128	("adenoid cystic carcinoma?" adj3 urethra?).ti,ab,kf.	1
129	"undifferentiated carcinoma?".ti,ab,kf. and "urinary bladder"/	14
130	("undifferentiated carcinoma?" adj3 bladder).ti,ab,kf.	20
131	"carcinoma, transitional cell"/ and "urinary bladder"/	2152
132	("transitional cell carcinoma?" adj3 bladder).ti,ab,kf.	3320
133	"lymphoepithelial carcinoma?".ti,ab,kf. and "urinary bladder"/	0
134	("lymphoepithelial carcinoma?" adj3 bladder).ti,ab,kf.	1
135	"carcinoma, giant cell"/ and "urinary bladder"/	5
136	("giant cell carcinoma?" adj3 bladder).ti,ab,kf.	8
137	("squamous cell carcinoma?" adj3 bladder).ti,ab,kf.	377
138	"basaloid carcinoma?".ti,ab,kf. and "urinary bladder"/	0
139	("basaloid carcinoma?" adj3 bladder).ti,ab,kf.	0
140	"Carcinoma, Verrucous"/ and "urinary bladder"/	4
141	("verrucous carcinoma?" adj3 bladder).ti,ab,kf.	14
142	exp adenocarcinoma/ and "urinary bladder"/	709
143	(adenocarcinoma? adj3 bladder).ti,ab,kf.	695
144	"carcinoma, signet ring cell"/ and "urinary bladder"/	24
145	("signet ring cell carcinoma?" adj3 bladder).ti,ab,kf.	67
146	"carcinoma, adenoid cystic"/ and "urinary bladder"/	0
147	("adenoid cystic carcinoma?" adj3 bladder).ti,ab,kf.	2
148	"carcinoma, mucoepidermoid"/ and "urinary bladder"/	0
149	("mucoepidermoid carcinoma?" adj3 bladder).ti,ab,kf.	0
150	"extragonadal germ cell tumo?r?".ti,ab,kf.	501
151	"embryonal adenocarcinoma?".ti,ab,kf.	11
152	"germ cell tumo?r?".ti,ab,kf. and exp "central nervous system"/	492
153	("germ cell tumo?r?" adj3 ("central nervous system" or CNS)).ti,ab,kf.	236
154	exp "wilms tumor"/	9401
155	nephroblastoma?.ti,ab,kf.	2712
156	or/1-155 [Domain 3 - Male genital organs and the urinary tract]	45832
157	*"quality of life"/ or ("Quality Of Life" or HRQOL or QOL).ti,ab,kf. [QoL]	385875
158	156 and 157 [Domain 3 AND QoL]	379

Domain 4 – Neuroendocrine

1	(rare adj3 (neuroendocrine or lung or thymic or thymus) adj3 (cancer? or tumo?r? or malignan*)).ti,ab,kf.	2270
2	"lung neoplasms"/ and "carcinoid tumor"/	1881
3	(lung? adj3 carcinoid?).ti,ab,kf.	688
4	"pulmonary carcinoid?".ti,ab,kf.	583
5	"pulmonary neuroendocrine tumo?r?".ti,ab,kf.	254
6	"thymus neoplasms"/ and "carcinoid tumor"/	482
7	"thymic carcinoid?".ti,ab,kf.	445
8	(thymus adj3 carcinoid?).ti,ab,kf.	59
9	"neuroendocrine tumors"/ and exp lung/	122
10	(("neuroendocrine tumo?r?" or NET) adj3 lung?).ti,ab,kf.	682

11	"neuroendocrine tumors"/ and (gastroenteropancreatic or "gastro entero pancreatic" or "GEP").ti,ab,kf.	1440
12	(("neuroendocrine tumo?r?" or "NET") adj3 (gastroenteropancreatic or "gastro entero pancreatic" or "GEP")).ti,ab,kf.	1237
13	"endocrine carcinoma?".ti,ab,kf. and (exp pancreas/ or exp "gastrointestinal tract"/)	59
14	("endocrine carcinoma?" adj3 (pancrea* or digestive or gastrointestinal)).ti,ab,kf.	103
15	"carcinoid tumor"/ and (exp pancreas/ or exp "gastrointestinal tract"/)	2207
16	(carcinoid? adj3 (pancrea* or digestive or gastrointestinal)).ti,ab,kf.	898
17	"carcinoma, islet cell"/ and (exp pancreas/ or exp "gastrointestinal tract"/)	93
18	("islet cell carcinoma?" adj3 (pancrea* or digestive or gastrointestinal)).ti,ab,kf.	190
19	(insulinoma? adj3 malignan*).ti,ab,kf.	353
20	glucagonoma/	774
21	glucagonoma?.ti,ab,kf.	983
22	(somatostatinoma? adj3 malignan*).ti,ab,kf.	25
23	gastrinoma/	995
24	gastrinoma?.ti,ab,kf.	1787
25	vipoma/	483
26	(vipoma? or "vip oma?" or "vasoactive intestinal peptide oma?").ti,ab,kf.	423
27	("mixed pancreatic" adj5 tumo?r?).ti,ab,kf.	7
28	paraganglioma/	5251
29	paraganglioma?.ti,ab,kf.	8659
30	"carcinoma, medullary"/ and "thyroid gland"/	289
31	("medullary carcinoma?" adj3 thyroid*).ti,ab,kf.	1290
32	("follicular carcinoma?" adj3 thyroid*).ti,ab,kf.	755
33	"endocrine gland neoplasms"/ and "thyroid gland"/	9
34	("endocrine tumo?r?" adj3 thyroid*).ti,ab,kf.	47
35	"carcinoma, neuroendocrine"/	5631
36	"neuroendocrine carcinoma?".ti,ab,kf.	6295
37	("well differentiated" adj3 "endocrine tumo?r?" adj3 carcinoid?).ti,ab,kf.	9
38	("poorly differentiated" adj3 "endocrine carcinoma?").ti,ab,kf.	72
39	neuroblastoma/	30263
40	neuroblastoma?.ti,ab,kf.	40443
41	ganglioneuroblastoma/	419
42	ganglioneuroblastoma?.ti,ab,kf.	940
43	or/1-42 [Domain 4 - Neuroendocrine]	79767
44	*"quality of life"/ or ("Quality Of Life" or HRQOL or QOL).ti,ab,kf. [QoL]	385875
45	43 and 44 [Domain 4 AND QoL]	526

Domain 5 - Digestive Tract

1	(rare adj3 "digestive tract" adj3 (cancer? or tumo?r? or malignan*)).ti,ab,kf.	22
2	exp "carcinoma, squamous cell"/ and exp esophagus/	2274
3	(("squamous cell carcinoma?" or SCC) adj3 (esophagus or oesophagus)).ti,ab,kf.	1950
4	"squamous carcinoma?".ti,ab,kf. adj3 ((esophagus or oesophagus).ti,ab,kf. or exp esophagus/)	191
5	"carcinoma, adenosquamous"/ and exp esophagus/	18
6	("adenosquamous carcinoma?" adj3 (esophagus or oesophagus)).ti,ab,kf.	19
7	"Carcinoma, Verrucous"/ and exp esophagus/	12
8	("verrucous carcinoma?" adj3 (esophagus or oesophagus)).ti,ab,kf.	34
9	"adenocarcinoma"/ and exp esophagus/	3783

10	(adenocarcinoma? adj3 (esophagus or oesophagus)).ti,ab,kf.	2525
11	"carcinoma, mucoepidermoid"/ and exp esophagus/	8
12	("mucoepidermoid carcinoma?" adj3 (esophagus or oesophagus)).ti,ab,kf.	20
13	"carcinoma, adenoid cystic"/ and exp esophagus/	19
14	("adenoid cystic carcinoma?" adj3 (esophagus or oesophagus)).ti,ab,kf.	40
15	"undifferentiated carcinoma?".ti,ab,kf. and exp esophagus/	20
16	("undifferentiated carcinoma?" adj3 (esophagus or oesophagus)).ti,ab,kf.	17
17	"adenocarcinoma"/ and exp stomach/	6526
18	(adenocarcinoma? adj3 (stomach or gastric)).ti,ab,kf.	12504
19	(adenocarcinoma? adj3 "intestinal type").ti,ab,kf.	724
20	"carcinoma, signet ring cell"/ and exp stomach/	263
21	("signet ring cell carcinoma?" and (stomach or gastric)).ti,ab,kf.	1136
22	"adenocarcinoma, mucinous"/ and exp stomach/	285
23	("mucinous adenocarcinoma?" adj3 (stomach or gastric)).ti,ab,kf.	71
24	"carcinoma, adenosquamous"/ and exp stomach/	24
25	("adenosquamous carcinoma?" adj3 (stomach or gastric)).ti,ab,kf.	70
26	exp "carcinoma, squamous cell"/ and exp stomach/	1303
27	(("squamous cell carcinoma?" or SCC) adj3 (stomach or gastric)).ti,ab,kf.	451
28	"squamous carcinoma?".ti,ab,kf. and exp stomach/	56
29	("squamous carcinoma?" adj3 (stomach or gastric)).ti,ab,kf.	25
30	"basaloid carcinoma?".ti,ab,kf. and exp stomach/	0
31	("basaloid carcinoma?" adj3 (stomach or gastric)).ti,ab,kf.	0
32	"Carcinoma, Verrucous"/ and exp stomach/	2
33	("verrucous carcinoma?" adj3 (stomach or gastric)).ti,ab,kf.	1
34	"carcinoma, mucoepidermoid"/ and exp stomach/	2
35	("mucoepidermoid carcinoma?" adj3 (stomach or gastric)).ti,ab,kf.	4
36	"carcinoma, adenoid cystic"/ and exp stomach/	6
37	("adenoid cystic carcinoma?" adj3 (stomach or gastric)).ti,ab,kf.	3
38	"undifferentiated carcinoma?".ti,ab,kf. and exp stomach/	73
39	("undifferentiated carcinoma?" adj3 (stomach or gastric)).ti,ab,kf.	29
40	"epithelial tumo?r?".ti,ab,kf. and exp "intestine, small"/	80
41	("epithelial tumo?r?" adj3 "small intestine?").ti,ab,kf.	4
42	"adenocarcinoma"/ and exp "intestine, small"/	3260
43	(adenocarcinoma? adj3 "small intestine?").ti,ab,kf.	266
44	"adenocarcinoma, mucinous"/ and exp "intestine, small"/	181
45	("mucinous adenocarcinoma?" adj3 "small intestine?").ti,ab,kf.	2
46	"carcinoma, signet ring cell"/ and exp "intestine, small"/	63
47	("signet ring cell carcinoma?" and "small intestine?").ti,ab,kf.	14
48	"carcinoma, adenosquamous"/ and exp "intestine, small"/	24
49	("adenosquamous carcinoma?" adj3 "small intestine?").ti,ab,kf.	3
50	"carcinoma, medullary"/ and exp "intestine, small"/	6
51	("medullary carcinoma?" adj3 "small intestine?").ti,ab,kf.	1
52	"undifferentiated carcinoma?".ti,ab,kf. and exp "intestine, small"/	21
53	("undifferentiated carcinoma?" adj3 "small intestine?").ti,ab,kf.	2
54	exp "carcinoma, squamous cell"/ and exp "intestine, small"/	429
55	(("squamous cell carcinoma?" or SCC) adj3 "small intestine?").ti,ab,kf.	12
56	"adenocarcinoma"/ and exp colon/	2411
57	(adenocarcinoma? adj3 colon*).ti,ab,kf.	10354
58	"carcinoma, signet ring cell"/ and exp colon/	42
59	("signet ring cell carcinoma?" and colon*).ti,ab,kf.	324

60	"adenocarcinoma, mucinous"/ and exp colon/	171
61	("mucinous adenocarcinoma?" adj3 colon*).ti,ab,kf.	119
62	"carcinoma, adenosquamous"/ and exp colon/	8
63	("adenosquamous carcinoma?" adj3 colon*).ti,ab,kf.	41
64	"undifferentiated carcinoma?".ti,ab,kf. and exp colon/	14
65	("undifferentiated carcinoma?" adj3 colon*).ti,ab,kf.	20
66	"carcinoma, medullary"/ and exp colon/	13
67	("medullary carcinoma?" adj3 colon*).ti,ab,kf.	23
68	exp "carcinoma, squamous cell"/ and exp colon/	237
69	(("squamous cell carcinoma?" or SCC) adj3 colon*).ti,ab,kf.	260
70	"adenocarcinoma, mucinous"/ and exp appendix/	102
71	("mucinous adenocarcinoma?" adj3 append*).ti,ab,kf.	167
72	(fibromyxoma? adj3 append*).ti,ab,kf.	0
73	exp adenocarcinoma/ and exp pancreas/	6284
74	(adenocarcinoma? adj3 pancrea*).ti,ab,kf.	24155
75	"adenocarcinoma, mucinous"/ and exp pancreas/	526
76	("mucinous adenocarcinoma?" adj3 pancrea*).ti,ab,kf.	33
77	"undifferentiated carcinoma?".ti,ab,kf. and exp pancreas/	51
78	("undifferentiated carcinoma?" adj3 pancrea*).ti,ab,kf.	74
79	"carcinoma, signet ring cell"/ and exp pancreas/	49
80	("signet ring cell carcinoma?" and pancrea*).ti,ab,kf.	99
81	"carcinoma, adenosquamous"/ and exp pancreas/	42
82	("adenosquamous carcinoma?" adj3 pancrea*).ti,ab,kf.	150
83	(("squamous cell carcinoma?" or SCC) adj3 pancrea*).ti,ab,kf.	169
84	"carcinoma, acinar cell"/ and exp pancreas/	131
85	("acinar cell carcinoma?" adj3 pancrea*).ti,ab,kf.	462
86	"cystadenocarcinoma, mucinous"/ and exp pancreas/	78
87	("mucinous cystadenocarcinoma" adj3 pancrea*).ti,ab,kf.	85
88	("intraductal papillary mucinous carcinoma?" adj3 pancrea*).ti,ab,kf.	55
89	("solid pseudopapillary carcinoma?" adj3 pancrea*).ti,ab,kf.	8
90	"cystadenocarcinoma, serous"/ and exp pancreas/	17
91	("serous cystadenocarcinoma?" adj3 pancrea*).ti,ab,kf.	30
92	("osteoclast-like giant cell" adj3 carcinoma? adj3 pancrea*).ti,ab,kf.	8
93	pancreatoblastoma.rs.	88
94	pancreatoblastoma?.ti,ab,kf.	322
95	exp "biliary tract neoplasms"/	32815
96	(biliary adj3 (neoplas* or cancer? or tumo?r? or cystadenocarcinoma?)).ti,ab,kf.	5582
97	("bile duct" adj3 (neoplas* or cancer? or tumo?r? or carcinoma? or adenocarcinoma?)).ti,ab,kf.	4938
98	cholangiocarcinoma/	11172
99	cholangiocarcinoma?.ti,ab,kf.	17391
100	"klatskin tumor"/	998
101	"klatskin tumo?r?".ti,ab,kf.	545
102	"gallbladder neoplasms"/	9682
103	"gallbladder neoplas*".ti,ab,kf.	797
104	"gallbladder cancer?".ti,ab,kf.	4657
105	"gallbladder carcinoma?".ti,ab,kf.	2672
106	"gallbladder sarcoma?".ti,ab,kf.	8
107	"gallbladder adenocarcinoma?".ti,ab,kf.	393
108	"ampullary cancer?".ti,ab,kf.	484

109	"vater papilla carcinoma?".ti,ab,kf.	0
110	"carcinoma, hepatocellular"/	101925
111	"hepatocellular carcinoma?".ti,ab,kf.	115435
112	exp adenocarcinoma/ and exp liver/	21629
113	(adenocarcinoma? adj3 (liver or "intrahepatic bil*")).ti,ab,kf.	634
114	"undifferentiated carcinoma?".ti,ab,kf. and exp liver/	29
115	("undifferentiated carcinoma?" adj3 (liver or "intrahepatic bil*")).ti,ab,kf.	16
116	(("squamous cell carcinoma?" or SCC) adj3 (liver or "intrahepatic bil*")).ti,ab,kf.	150
117	"bile duct cystadenocarcinoma?".ti,ab,kf.	10
118	hepatoblastoma/	2048
119	hepatoblastoma?.ti,ab,kf.	4035
120	"epithelial tumo?r?".ti,ab,kf. and (gallbladder/ or exp "extrahepatic bile ducts"/)	32
121	("epithelial tumo?r?" adj3 (gallbladder or "extrahepatic bil*")).ti,ab,kf.	13
122	"adenocarcinoma"/ and "extrahepatic bile ducts"/	154
123	(adenocarcinoma? adj3 "extrahepatic bil*").ti,ab,kf.	58
124	exp "carcinoma, squamous cell"/ and "extrahepatic bile ducts"/	13
125	(("squamous cell carcinoma?" or SCC) adj3 "extrahepatic bil*").ti,ab,kf.	9
126	"adenocarcinoma"/ and gallbladder/	306
127	(adenocarcinoma? adj3 gallbladder?).ti,ab,kf.	747
128	"adenocarcinoma, mucinous"/ and gallbladder/	23
129	("mucinous adenocarcinoma?" adj3 gallbladder?).ti,ab,kf.	15
130	"adenocarcinoma, clear cell"/ and gallbladder/	1
131	("clear cell adenocarcinoma?" adj3 gallbladder?).ti,ab,kf.	2
132	"carcinoma, adenosquamous"/ and gallbladder/	15
133	("adenosquamous carcinoma?" adj3 gallbladder?).ti,ab,kf.	42
134	"undifferentiated carcinoma?".ti,ab,kf. and gallbladder/	3
135	("undifferentiated carcinoma?" adj3 gallbladder?).ti,ab,kf.	18
136	exp "anus neoplasms"/	7086
137	((anal or anus) adj3 (cancer? or neoplas* or carcinoma? or adenocarcinoma?)).ti,ab,kf.	6203
138	SSCA.ti,ab,kf.	162
139	"epithelial tumo?r?".ti,ab,kf. and exp rectum/	16
140	("epithelial tumo?r?" adj3 rect*).ti,ab,kf.	12
141	"adenocarcinoma"/ and exp rectum/	1732
142	(adenocarcinoma? adj3 rect*).ti,ab,kf.	3910
143	"carcinoma, signet ring cell"/ and exp rectum/	21
144	("signet ring cell carcinoma?" and rect*).ti,ab,kf.	164
145	"adenocarcinoma, mucinous"/ and exp rectum/	130
146	("mucinous adenocarcinoma?" adj3 rect*).ti,ab,kf.	76
147	"carcinoma, adenosquamous"/ and exp rectum/	10
148	("adenosquamous carcinoma?" adj3 rect*).ti,ab,kf.	10
149	"undifferentiated carcinoma".ti,ab,kf. and exp rectum/	3
150	("undifferentiated carcinoma" adj3 rect*).ti,ab,kf.	5
151	"carcinoma, medullary"/ and exp rectum/	5
152	("medullary carcinoma?" adj3 rect*).ti,ab,kf.	0
153	exp "carcinoma, squamous cell"/ and exp rectum/	218
154	(("squamous cell carcinoma?" or SCC) adj3 rect*).ti,ab,kf.	189
155	"Carcinoma, Verrucous"/ and exp rectum/	0
156	("verrucous carcinoma?" adj3 rect*).ti,ab,kf.	1
157	"basaloid carcinoma?".ti,ab,kf. and exp rectum/	3

158	("basaloid carcinoma?" adj3 rect*).ti,ab,kf.	4
159	"epithelial tumo?r?".ti,ab,kf. and exp "anal canal"/	2
160	("epithelial tumo?r?" adj3 "anal canal").ti,ab,kf.	1
161	(("squamous cell carcinoma?" or SCC) adj3 "anal canal").ti,ab,kf.	383
162	"Carcinoma, Verrucous"/ and exp "anal canal"/	4
163	("verrucous carcinoma?" adj3 "anal canal").ti,ab,kf.	0
164	"basaloid carcinoma?".ti,ab,kf. and exp "anal canal"/	8
165	("basaloid carcinoma?" adj3 "anal canal").ti,ab,kf.	10
166	"adenocarcinoma"/ and exp "anal canal"/	532
167	(adenocarcinoma? adj3 "anal canal").ti,ab,kf.	97
168	("mucinous adenocarcinoma?" adj3 "anal canal").ti,ab,kf.	9
169	"carcinoma, adenosquamous"/ and exp "anal canal"/	0
170	("adenosquamous carcinoma?" adj3 "anal canal").ti,ab,kf.	2
171	"paget disease, extramammary"/ and exp "anal canal"/	69
172	("paget disease" adj3 "anal canal").ti,ab,kf.	0
173	"pseudomyxoma peritonei"/	1149
174	"pseudomyxoma peritonei".ti,ab,kf.	1670
175	"peritoneum pseudomyxoma".ti,ab,kf.	5
176	"gelatinous ascites".ti,ab,kf.	44
177	"peritoneal neoplasms"/ and exp mesothelioma/	2223
178	"peritoneal mesothelioma?".ti,ab,kf.	1648
179	"peritoneum mesothelioma?".ti,ab,kf.	11
180	"primary peritoneal serous carcinoma?".ti,ab,kf.	80
181	PPSC.ti,ab,kf.	89
182	"peritoneal desmoplastic small round cell tumo?r?".ti,ab,kf.	1
183	"peritoneal desmoplastic small cell tumo?r?".ti,ab,kf.	0
184	"peritoneal neoplasms"/ and leiomyomatosis/	131
185	"diffuse peritoneal leiomyomatosis".ti,ab,kf.	11
186	"leiomyomatosis peritonealis disseminata".ti,ab,kf.	202
187	"carcinomatous peritonitis".ti,ab,kf.	156
188	or/1-187 [Domain 5 - Digestive Tract]	273888
189	*"quality of life"/ or ("Quality Of Life" or HRQOL or QOL).ti,ab,kf. [QoL]	385875
190	188 and 189 [Domain 5 AND QoL]	3239

Domain 6 – Endocrine

1	(rare adj3 endocrine adj3 (cancer? or tumo?r? or malignan*)).ti,ab,kf.	351
2	exp "thyroid neoplasms"/	60307
3	(thyroid* adj3 (cancer? or neoplas* or adenoma? or lymphoma? or sarcoma? or carcinoma?)).ti,ab,kf.	59140
4	"thyroid carcinoma, anaplastic"/	931
5	"anaplastic thyroid carcinoma?".ti,ab,kf.	1573
6	"hurthle cell carcinoma?".ti,ab,kf.	553
7	"adenocarcinoma, papillary"/ and "thyroid gland"/	161
8	("papillary adenocarcinoma?" adj3 thyroid*).ti,ab,kf.	115
9	"undifferentiated carcinoma?".ti,ab,kf. and "thyroid gland"/	54
10	("undifferentiated carcinoma?" adj3 thyroid*).ti,ab,kf.	52
11	"carcinoma, mucoepidermoid"/ and "thyroid gland"/	22
12	("mucoepidermoid carcinoma?" adj3 thyroid*).ti,ab,kf.	63
13	"adenocarcinoma, mucinous"/ and "thyroid gland"/	9

14	("mucinous adenocarcinoma?" adj3 thyroid*).ti,ab,kf.	4
15	"spindle cell tumo?r? with thymus like differentiation?".ti,ab,kf.	3
16	(carcinoma? adj3 "thymus like differentiation").ti,ab,kf.	77
17	carcinoma/ and "parathyroid glands"/	246
18	(carcinoma? adj3 parathyroid*).ti,ab,kf.	1738
19	"adrenocortical carcinoma"/	2010
20	"adrenocortical carcinoma?".ti,ab,kf.	3638
21	"adrenal cortex carcionoma?".ti,ab,kf.	0
22	"adrenal cortical carcinoma?".ti,ab,kf.	613
23	"suprarenal carcinoma?".ti,ab,kf.	12
24	"pheochromocytoma"/ and malignan*.ti,ab,kf.	2070
25	(malignant adj3 (ph?eochromocytoma? or phaeochromoblastoma or "chromaffin cell tumo?r?" or "chromaffin paraganglioma?")).ti,ab,kf.	1271
26	(ph?eochromocytoma? adj3 paraganglioma?).ti,ab,kf.	2261
27	PPGL.ti,ab,kf.	432
28	carcinoma/ and exp "adrenal glands"/	410
29	(carcinoma? adj3 "adrenal gland?").ti,ab,kf.	154
30	carcinoma/ and exp "pituitary glands"/	172
31	(carcinoma? adj3 ("pituitar*" or hypophys?s)).ti,ab,kf.	676
32	or/1-31 [Domain 6 - Endocrine]	87510
33	*"quality of life"/ or ("Quality Of Life" or HRQOL or QOL).ti,ab,kf. [QoL]	385875
34	32 and 33 [Domain 6 AND QoL]	1063

Domain 7 - Head and Neck

1	(rare adj3 (head or neck) adj3 (cancer? or tumo?r? or malignan*)).ti,ab,kf.	343
2	(sinonasal adj3 (carcinoma? or SCC or melanoma? or sarcoma? or cancer? or tumo?r? or malignan* or adenocarcinoma?)).ti,ab,kf.	2713
3	(paranasal adj3 (carcinoma? or SCC or melanoma? or sarcoma? or cancer? or tumo?r? or malignan* or adenocarcinoma?)).ti,ab,kf.	1131
4	(nasal adj3 (carcinoma? or SCC or melanoma? or sarcoma? or cancer? or tumo?r? or malignan* or adenocarcinoma?)).ti,ab,kf.	3835
5	(sinus adj3 (carcinoma? or SCC or melanoma? or sarcoma? or cancer? or tumo?r? or malignan* or adenocarcinoma?)).ti,ab,kf.	3458
6	"inverting papilloma?".ti,ab,kf.	94
7	"midline granuloma?".ti,ab,kf.	276
8	"olfactory neuroblastoma?".ti,ab,kf.	870
9	"esthesioneuroblastoma?".ti,ab,kf.	925
10	"nose cavity cancer?".ti,ab,kf.	0
11	"epithelial tumo?r?".ti,ab,kf. and ("nasal cavity"/ or exp "paranasal sinuses"/)	35
12	("epithelial tumo?r?" adj3 ("nasal cavit*" or "nasal sinus*" or "paranasal sinus*")).ti,ab,kf.	23
13	exp "carcinoma, squamous cell"/ and ("nasal cavity"/ or exp "paranasal sinuses"/)	764
14	(("squamous cell carcinoma?" or SCC) adj3 ("nasal cavit*" or "nasal sinus*" or "paranasal sinus*")).ti,ab,kf.	131
15	("squamous carcinoma?" adj3 ("nasal cavit*" or "nasal sinus*" or "paranasal sinus*")).ti,ab,kf.	10
16	"Carcinoma, Verrucous"/ and ("nasal cavity"/ or exp "paranasal sinuses"/)	8
17	("verrucous carcinoma?" adj3 ("nasal cavit*" or "nasal sinus*" or "paranasal sinus*")).ti,ab,kf.	6

18	"basaloid carcinoma?".ti,ab,kf. and ("nasal cavity"/ or exp "paranasal sinuses"/)	1
19	("basaloid carcinoma?" adj3 ("nasal cavit*" or "nasal sinus*" or "paranasal sinus*")).ti,ab,kf.	0
20	"carcinoma, adenosquamous"/ and ("nasal cavity"/ or exp "paranasal sinuses"/)	5
21	("adenosquamous carcinoma?" adj3 ("nasal cavit*" or "nasal sinus*" or "paranasal sinus*")).ti,ab,kf.	2
22	"lymphoepithelial carcinoma?".ti,ab,kf. and ("nasal cavity"/ or exp "paranasal sinuses"/)	5
23	("lymphoepithelial carcinoma?" adj3 ("nasal cavit*" or "nasal sinus*" or "paranasal sinus*")).ti,ab,kf.	2
24	"undifferentiated carcinoma?".ti,ab,kf. and ("nasal cavity"/ or exp "paranasal sinuses"/)	111
25	("undifferentiated carcinoma?" adj3 ("nasal cavit*" or "nasal sinus*" or "paranasal sinus*")).ti,ab,kf.	8
26	exp "salivary gland neoplasms"/	18968
27	("salivary gland?" adj3 (cancer? or tumo?r? or malignan* or carcinoma?)).ti,ab,kf.	8402
28	"salivary gland lymphadenoma?".ti,ab,kf.	2
29	"non-sebaceous lymphadenoma?".ti,ab,kf.	11
30	"sebaceous lymphadenoma?".ti,ab,kf.	80
31	(parotid adj3 (cancer? or tumo?r? or malignan* or carcinoma?)).ti,ab,kf.	5392
32	("sublingual gland?" adj3 (cancer? or tumo?r? or malignan* or carcinoma?)).ti,ab,kf.	77
33	("submandibular gland?" adj3 (cancer? or tumo?r? or malignan* or carcinoma?)).ti,ab,kf.	513
34	"warthin tumo?r?".ti,ab,kf.	502
35	"squamous carcinoma?".ti,ab,kf. and exp "salivary glands"/	29
36	("squamous carcinoma?" adj3 "salivary gland?").ti,ab,kf.	5
37	"lymphoepithelial carcinoma?".ti,ab,kf. and exp "salivary glands"/	35
38	("lymphoepithelial carcinoma?" adj3 "salivary gland?").ti,ab,kf.	36
39	exp adenocarcinoma/ and exp "salivary glands"/	1773
40	(adenocarcinoma? adj3 "salivary gland?").ti,ab,kf.	357
41	"adenocarcinoma, mucinous"/ and "salivary glands"/	6
42	("mucinous adenocarcinoma?" adj3 "salivary gland?").ti,ab,kf.	11
43	"cystadenocarcinoma, papillary"/ and exp "salivary glands"/	7
44	("papillary cystadenocarcinoma?" adj3 "salivary gland?").ti,ab,kf.	11
45	"carcinoma, adenoid cystic"/ and exp "salivary glands"/	683
46	("adenoid cystic carcinoma?" adj3 "salivary gland?").ti,ab,kf.	495
47	"carcinoma, mucoepidermoid"/ and exp "salivary glands"/	391
48	("mucoepidermoid carcinoma?" adj3 "salivary gland?").ti,ab,kf.	298
49	"carcinoma, acinar cell"/ and exp "salivary glands"/	147
50	("acinar cell carcinoma?" adj3 "salivary gland?").ti,ab,kf.	3
51	(myoepithelioma? adj3 malignan* adj3 "salivary gland?").ti,ab,kf.	17
52	carcinoma/ and "adenoma, pleomorphic"/	494
53	(carcinoma? adj3 "pleomorphic adenoma?").ti,ab,kf.	863
54	"mixed tumor, malignant"/ and exp "salivary glands"/	13
55	("malignant mixed tumo?r?" adj3 "salivary gland?").ti,ab,kf.	43
56	(epithelial adj1 myoepithelial adj1 carcinoma? adj3 "salivary gland?").ti,ab,kf.	57
57	"adenocarcinoma, sebaceous"/ and exp "salivary glands"/	10
58	("sebaceous adenocarcinoma?" adj3 "salivary gland?").ti,ab,kf.	1
59	exp cystadenocarcinoma/ and exp "salivary glands"/	28

60	(cystadenocarcinoma? adj3 "salivary gland?").ti,ab,kf.	31
61	"carcinoma, ductal"/ and exp "salivary glands"/	87
62	("ductal carcinoma?" adj3 "salivary gland?").ti,ab,kf.	9
63	"oncocytic carcinoma?".ti,ab,kf. and exp "salivary glands"/	20
64	("oncocytic carcinoma?" adj3 "salivary gland?").ti,ab,kf.	14
65	"nasopharyngeal carcinoma"/	5851
66	((nasopharyn* or rhinopharyn*) adj3 (carcinoma? or adenocarcinoma? or cancer?)).ti,ab,kf.	18588
67	(nasopharyn* adj3 SCQ).ti,ab,kf.	0
68	"lymphoepithelioma?".ti,ab,kf.	1120
69	"epithelial tumo?r?".ti,ab,kf. and exp nasopharynx/	7
70	("epithelial tumo?r?" adj3 nasopharyn*).ti,ab,kf.	31
71	exp "carcinoma, squamous cell"/ and exp nasopharynx/	147
72	(("squamous cell carcinoma?" or SCC) adj3 nasopharyn*).ti,ab,kf.	231
73	"squamous carcinoma?".ti,ab,kf. and exp nasopharynx/	6
74	("squamous carcinoma?" adj3 nasopharyn*).ti,ab,kf.	27
75	"basaloid carcinoma?".ti,ab,kf. and exp nasopharynx/	2
76	("basaloid carcinoma?" adj3 nasopharyn*).ti,ab,kf.	0
77	"lymphoepithelial carcinoma?".ti,ab,kf. and exp nasopharynx/	2
78	("lymphoepithelial carcinoma?" adj3 nasopharyn*).ti,ab,kf.	21
79	"undifferentiated carcinoma?".ti,ab,kf. and exp nasopharynx/	23
80	("undifferentiated carcinoma?" adj3 nasopharyn*).ti,ab,kf.	167
81	"adenocarcinoma, papillary"/ and exp nasopharynx/	11
82	("papillary adenocarcinoma?" adj3 nasopharyn*).ti,ab,kf.	63
83	("middle ear" adj3 (cancer? or tumo?r? or malignan* or schwannoma? or meningioma? or adenoma? or paraganglioma? or carcinoma?)).ti,ab,kf.	806
84	exp "carcinoma, squamous cell"/ and exp hypopharynx/	392
85	(("squamous cell carcinoma?" or SCC) adj3 hypophary*).ti,ab,kf.	894
86	"squamous carcinoma?".ti,ab,kf. and exp hypopharynx/	14
87	("squamous carcinoma?" adj3 hypophary*).ti,ab,kf.	44
88	"lymphoepithelial carcinoma?".ti,ab,kf. and exp hypopharynx/	3
89	("lymphoepithelial carcinoma?" adj3 hypophary*).ti,ab,kf.	4
90	"Carcinoma, Verrucous"/ and exp hypopharynx/	0
91	("verrucous carcinoma?" adj3 hypophary*).ti,ab,kf.	0
92	"carcinoma, giant cell"/ and exp hypopharynx/	0
93	("giant cell carcinoma?" adj3 hypophary*).ti,ab,kf.	1
94	"carcinoma, adenosquamous"/ and exp hypopharynx/	1
95	("adenosquamous carcinoma?" adj3 hypophary*).ti,ab,kf.	4
96	"undifferentiated carcinoma?".ti,ab,kf. and exp hypopharynx/	1
97	("undifferentiated carcinoma?" adj3 hypophary*).ti,ab,kf.	1
98	exp "carcinoma, squamous cell"/ and exp larynx/	2761
99	(("squamous cell carcinoma?" or SCC) adj3 laryn*).ti,ab,kf.	3993
100	"squamous carcinoma?".ti,ab,kf. and exp larynx/	89
101	("squamous carcinoma?" adj3 laryn*).ti,ab,kf.	276
102	"Carcinoma, Verrucous"/ and exp larynx/	29
103	("verrucous carcinoma?" adj3 laryn*).ti,ab,kf.	75
104	"carcinoma, adenosquamous"/ and exp larynx/	6
105	("adenosquamous carcinoma?" adj3 laryn*).ti,ab,kf.	13
106	"undifferentiated carcinoma?".ti,ab,kf. and exp larynx/	10
107	("undifferentiated carcinoma?" adj3 laryn*).ti,ab,kf.	4

108	"lymphoepithelial carcinoma?".ti,ab,kf. and exp larynx/	8
109	("lymphoepithelial carcinoma?" adj3 laryn*).ti,ab,kf.	16
110	"carcinoma, giant cell"/ and exp larynx/	1
111	("giant cell carcinoma?" adj3 laryn*).ti,ab,kf.	3
112	"epithelial tumo?r?".ti,ab,kf. and oropharynx/	5
113	("epithelial tumo?r?" adj3 oropharyn*).ti,ab,kf.	2
114	exp "carcinoma, squamous cell"/ and exp oropharynx/	458
115	(("squamous cell carcinoma?" or SCC) adj3 oropharyn*).ti,ab,kf.	3357
116	"squamous carcinoma?".ti,ab,kf. and oropharynx/	10
117	("squamous carcinoma?" adj3 oropharyn*).ti,ab,kf.	66
118	"lymphoepithelial carcinoma?".ti,ab,kf. and oropharynx/	0
119	("lymphoepithelial carcinoma?" adj3 oropharyn*).ti,ab,kf.	1
120	"Carcinoma, Verrucous"/ and exp oropharynx/	0
121	("verrucous carcinoma?" adj3 oropharyn*).ti,ab,kf.	0
122	"carcinoma, adenosquamous"/ and exp oropharynx/	1
123	("adenosquamous carcinoma?" adj3 oropharyn*).ti,ab,kf.	2
124	"undifferentiated carcinoma?".ti,ab,kf. and oropharynx/	1
125	("undifferentiated carcinoma?" adj3 oropharyn*).ti,ab,kf.	3
126	"epithelial tumo?r?".ti,ab,kf. and exp mouth/	150
127	("epithelial tumo?r?" adj3 ("oral cavit*" or mouth? or "lip*")).ti,ab,kf.	20
128	exp "carcinoma, squamous cell"/ and mouth/	688
129	(("squamous cell carcinoma?" or SCC) adj3 ("oral cavit*" or mouth?)).ti,ab,kf.	2762
130	"squamous carcinoma?".ti,ab,kf. and mouth/	14
131	("squamous carcinoma?" adj3 ("oral cavit*" or mouth?)).ti,ab,kf.	88
132	"Carcinoma, Verrucous"/ and mouth/	12
133	("verrucous carcinoma?" adj3 ("oral cavit*" or mouth?)).ti,ab,kf.	69
134	"carcinoma, adenosquamous"/ and mouth/	1
135	("adenosquamous carcinoma?" adj3 ("oral cavit*" or mouth?)).ti,ab,kf.	4
136	"lymphoepithelial carcinoma?".ti,ab,kf. and mouth/	1
137	("lymphoepithelial carcinoma?" adj3 ("oral cavit*" or mouth?)).ti,ab,kf.	2
138	"undifferentiated carcinoma?".ti,ab,kf. and mouth/	1
139	("undifferentiated carcinoma?" adj3 ("oral cavit*" or mouth?)).ti,ab,kf.	3
140	exp "carcinoma, squamous cell"/ and lip/	432
141	(("squamous cell carcinoma?" or SCC) adj3 lip?).ti,ab,kf.	443
142	"epithelial tumo?r?".ti,ab,kf. and exp "ear, middle"/	9
143	("epithelial tumo?r?" adj3 "middle ear?").ti,ab,kf.	3
144	exp "carcinoma, squamous cell"/ and exp "ear, middle"/	198
145	(("squamous cell carcinoma?" or SCC) adj3 "middle ear?").ti,ab,kf.	40
146	exp adenocarcinoma/ and exp "ear, middle"/	189
147	(adenocarcinoma? adj3 "middle ear?").ti,ab,kf.	37
148	"carcinoma, adenoid cystic"/ and exp "ear, middle"/	33
149	("adenoid cystic carcinoma?" adj3 "middle ear?").ti,ab,kf.	4
150	"odontogenic malignant tumo?r?".ti,ab,kf.	8
151	"clear cell odontogenic carcinoma?".ti,ab,kf.	104
152	"ghost cell odontogenic carcinoma?".ti,ab,kf.	45
153	"esthesioneuroblastoma, olfactory"/	822
154	"olfactory neuroblastoma?".ti,ab,kf.	870
155	or/1-154 [Domain 7 - Head and Neck]	70178
156	*"quality of life"/ or ("Quality Of Life" or HRQOL or QOL).ti,ab,kf. [QoL]	385875
157	155 and 156 [Domain 7 AND QoL]	1162

Domain 8 – Thorax

1	(rare adj3 (thorax or thoracic) adj3 (cancer? or tumo?r? or malignan*)).ti,ab,kf.	81
2	"thymus neoplasms"/	9993
3	((thymic or thymus) adj3 (cancer? or tumo?r? or malignan* or carcinoma?)).ti,ab,kf.	4860
4	thymoma?.ti,ab,kf.	10267
5	"epithelial tumo?r?".ti,ab,kf. and "thymus gland"/	120
6	("epithelial tumo?r?" adj3 thym*).ti,ab,kf.	1062
7	thymolipoma?.ti,ab,kf.	243
8	"good syndrome".ti,ab,kf.	123
9	"good's syndrome".ti,ab,kf.	198
10	exp "carcinoma, squamous cell"/ and "thymus gland"/	62
11	(("squamous cell carcinoma?" or SCC) adj3 thym*).ti,ab,kf.	194
12	exp adenocarcinoma/ and "thymus gland"/	277
13	(adenocarcinoma? adj3 thym*).ti,ab,kf.	110
14	"undifferentiated carcinoma?".ti,ab,kf. and "thymus gland"/	3
15	("undifferentiated carcinoma?" adj3 thym*).ti,ab,kf.	11
16	"lung neoplasms"/ and mesothelioma/	4831
17	"malignant mesothelioma"/	2858
18	"pleura? mesothelioma?".ti,ab,kf.	6237
19	("squamous cell carcinoma?" adj3 "small cell" adj3 (lung? or pleura?)).ti,ab,kf.	250
20	("squamous cell carcinoma?" adj3 "clear cell" adj3 (lung? or pleura?)).ti,ab,kf.	7
21	"adenocarcinoma"/ and exp lung/	2944
22	(adenocarcinoma? adj3 (lung? or pleura?)).ti,ab,kf.	27791
23	"adenocarcinoma, mucinous"/ and exp lung/	80
24	("mucinous adenocarcinoma?" adj3 (lung? or pleura?)).ti,ab,kf.	108
25	"solid carcinoma?".ti,ab,kf. and exp lung/	3
26	("solid carcinoma?" adj3 (lung? or pleura?)).ti,ab,kf.	1
27	"adenocarcinoma, clear cell"/ and exp lung/	8
28	("clear cell adenocarcinoma?" adj3 (lung? or pleura?)).ti,ab,kf.	14
29	"carcinoma, acinar cell"/ and exp lung/	9
30	("acinar cell carcinoma?" adj3 (lung? or pleura?)).ti,ab,kf.	1
31	"carcinoma, signet ring cell"/ and exp lung/	18
32	("signet ring cell carcinoma?" adj3 (lung? or pleura?)).ti,ab,kf.	28
33	"cystadenocarcinoma, mucinous"/ and exp lung/	4
34	("mucinous cystadenocarcinoma" adj3 (lung? or pleura?)).ti,ab,kf.	6
35	"adenocarcinoma, bronchiolo-alveolar"/	2651
36	"bronchiolo-alveolar adenocarcinoma?".ti,ab,kf.	16
37	"bronchioloalveolar adenocarcinoma?".ti,ab,kf.	46
38	"carcinoma, adenosquamous"/ and exp lung/	56
39	("adenosquamous carcinoma?" adj3 (lung? or pleura?)).ti,ab,kf.	157
40	"carcinoma, large cell"/ and exp lung/	190
41	("large cell carcinoma?" adj3 (lung? or pleura?)).ti,ab,kf.	368
42	("poorly differentiated" adj3 "endocrine carcinoma?" adj3 (lung? or pleura?)).ti,ab,kf.	0

43	(("small cell" or "large cell") adj3 "endocrine carcionoma?" adj3 (lung? or pleura?)).ti,ab,kf.	0
44	"carcinoma, mucoepidermoid"/ and exp lung/	52
45	("mucoepidermoid carcinoma?" adj3 (lung? or pleura?)).ti,ab,kf.	99
46	"carcinoma, adenoid cystic"/ and exp lung/	180
47	("adenoid cystic carcinoma?" adj3 (lung? or pleura?)).ti,ab,kf.	96
48	(epithelial adj1 myoepithelial adj1 carcinoma? adj3 (lung? or pleura?)).ti,ab,kf.	21
49	"sarcomatoid carcinoma?".ti,ab,kf. and exp lung/	30
50	("sarcomatoid carcinoma?" adj3 (lung? or pleura?)).ti,ab,kf.	129
51	"pleomorphic carcinoma?".ti,ab,kf. and exp lung/	29
52	("pleomorphic carcinoma?" adj3 (lung? or pleura?)).ti,ab,kf.	123
53	"carcinoma, giant cell"/ and exp lung/	13
54	("giant cell carcinoma?" adj3 (lung? or pleura?)).ti,ab,kf.	146
55	"pulmonary blastoma"/	510
56	"pulmonary blastoma?".ti,ab,kf.	420
57	"epithelial tumo?r?".ti,ab,kf. and trachea/	6
58	("epithelial tumo?r?" adj3 trachea?).ti,ab,kf.	4
59	exp "carcinoma, squamous cell"/ and trachea/	375
60	(("squamous cell carcinoma?" or SCC) adj3 trachea?).ti,ab,kf.	97
61	"squamous carcinoma?".ti,ab,kf. and trachea/	9
62	("squamous carcinoma?" adj3 trachea?).ti,ab,kf.	8
63	"carcinoma, adenosquamous"/ and trachea/	3
64	("adenosquamous carcinoma?" adj3 trachea?).ti,ab,kf.	0
65	"Carcinoma, Verrucous"/ and trachea/	0
66	("verrucous carcinoma?" adj3 trachea?).ti,ab,kf.	0
67	"undifferentiated carcinoma?".ti,ab,kf. and trachea/	10
68	("undifferentiated carcinoma?" adj3 trachea?).ti,ab,kf.	4
69	"spindle cell carcinoma?".ti,ab,kf. and trachea/	4
70	("spindle cell carcinoma?" adj3 trachea?).ti,ab,kf.	1
71	"lymphoepithelial carcinoma?".ti,ab,kf. and trachea/	1
72	("lymphoepithelial carcinoma?" adj3 trachea?).ti,ab,kf.	1
73	"carcinoma, giant cell"/ and trachea/	0
74	("giant cell carcinoma?" adj3 trachea?).ti,ab,kf.	0
75	exp adenocarcinoma/ and trachea/	422
76	(adenocarcinoma? adj3 trachea?).ti,ab,kf.	33
77	"adenocarcinoma, mucinous"/ and trachea/	1
78	("mucinous adenocarcinoma?" adj3 trachea?).ti,ab,kf.	2
79	"adenocarcinoma, papillary"/ and trachea/	18
80	("papillary adenocarcinoma?" adj3 trachea?).ti,ab,kf.	1
81	"solid carcinoma?".ti,ab,kf. and trachea/	0
82	("solid carcinoma?" adj3 trachea?).ti,ab,kf.	0
83	"adenocarcinoma, clear cell"/ and trachea/	2
84	("clear cell adenocarcinoma?" adj3 trachea?).ti,ab,kf.	0
85	"carcinoma, signet ring cell"/ and trachea/	0
86	("signet ring cell carcinoma?" and trachea?).ti,ab,kf.	3
87	"cystadenocarcinoma, mucinous"/ and trachea/	0
88	("mucinous cystadenocarcinoma" adj3 trachea?).ti,ab,kf.	0
89	"carcinoma, adenoid cystic"/ and trachea/	180
90	("adenoid cystic carcinoma?" adj3 trachea?).ti,ab,kf.	196
91	"carcinoma, mucoepidermoid"/ and trachea/	15

92	("mucoepidermoid carcinoma?" adj3 trachea?).ti,ab,kf.	30
93	(myoepithelial adj1 carcinoma? adj3 trachea?).ti,ab,kf.	5
94	(classic adj3 "invasive lobular carcinoma?" adj3 (breast? or mamma*)).ti,ab,kf.	4
95	"pleomorphic carcinoma?".ti,ab,kf. and exp breast/	1
96	("pleomorphic carcinoma?" adj3 (breast? or mamma*)).ti,ab,kf.	9
97	"paget's disease, mammary"/	802
98	("paget's disease" adj3 (breast? or mamma*)).ti,ab,kf.	572
99	("tubular adenocarcinoma?" adj3 (breast? or mamma*)).ti,ab,kf.	6
100	"mucinous carcinoma?".ti,ab,kf. and exp breast/	96
101	("mucinous carcinoma?" adj3 (breast? or mamma*)).ti,ab,kf.	213
102	"carcinoma, medullary"/ and exp breast/	47
103	("medullary carcinoma?" adj3 (breast? or mamma*)).ti,ab,kf.	155
104	"adenocarcinoma, papillary"/ and exp breast/	12
105	("papillary adenocarcinoma?" adj3 (breast? or mamma*)).ti,ab,kf.	11
106	"cribriform carcinoma?".ti,ab,kf. and exp breast/	11
107	("cribriform carcinoma?" adj3 (breast? or mamma*)).ti,ab,kf.	27
108	"apocrine adenocarcinoma?".ti,ab,kf. and exp breast/	2
109	("apocrine adenocarcinoma?" adj3 (breast? or mamma*)).ti,ab,kf.	3
110	"secretory breast carcinoma".rs.	107
111	"secretory carcinoma?".ti,ab,kf. adj3 (exp breast/ or (breast? or mamma*).ti,ab,kf.)	373
112	"apocrine adenocarcinoma?".ti,ab,kf. and exp breast/	2
113	("apocrine adenocarcinoma?" adj3 (breast? or mamma*)).ti,ab,kf.	3
114	"glycogen-rich carcinoma?".ti,ab,kf. and exp breast/	0
115	("glycogen-rich carcinoma?" adj3 (breast? or mamma*)).ti,ab,kf.	5
116	"lipid-rich carcinoma?".ti,ab,kf. and exp breast/	3
117	("lipid-rich carcinoma?" adj3 (breast? or mamma*)).ti,ab,kf.	22
118	"oncocytic carcinoma?".ti,ab,kf. and exp breast/	1
119	("oncocytic carcinoma?" adj3 (breast? or mamma*)).ti,ab,kf.	6
120	"metaplastic carcinoma?".ti,ab,kf. and exp breast/	83
121	("metaplastic carcinoma?" adj3 (breast? or mamma*)).ti,ab,kf.	230
122	"squamous carcinoma?".ti,ab,kf. and exp breast/	15
123	("squamous carcinoma?" adj3 (breast? or mamma*)).ti,ab,kf.	41
124	"carcinoma, adenosquamous"/ and exp breast/	20
125	("adenosquamous carcinoma?" adj3 (breast? or mamma*)).ti,ab,kf.	41
126	(adenocarcinoma? adj3 (cartil* or osseous) adj3 metaplas* adj3 (breast? or mamma*)).ti,ab,kf.	0
127	"carcinoma, mucoepidermoid"/ and exp breast/	7
128	("mucoepidermoid carcinoma?" adj3 (breast? or mamma*)).ti,ab,kf.	35
129	"carcinoma, adenoid cystic"/ and exp breast/	65
130	("adenoid cystic carcinoma?" adj3 (breast? or mamma*)).ti,ab,kf.	261
131	(myoepithelial adj1 carcinoma? adj3 (breast? or mamma*)).ti,ab,kf.	37
132	"carcinoma, acinar cell"/ and exp breast/	11
133	("acinar cell carcinoma?" adj3 (breast? or mamma*)).ti,ab,kf.	6
134	"mesothelioma, malignant"/	2858
135	(malignan* adj3 mesothelioma?).ti,ab,kf.	10133
136	mesothelioma?.ti,ab,kf. and (pleura/ or exp pericardium/)	1340
137	(mesothelioma? adj3 (pleura or pericardium)).ti,ab,kf.	714
138	mesothelioma?.ti,ab,kf. and peritoneum/	255
139	(mesothelioma? adj3 (peritoneum or "tunica vaginalis")).ti,ab,kf.	520

140	"pleuropulmonary blastoma".rs.	149
141	(pleuropulmonary adj3 blastoma?).ti,ab,kf.	464
142	or/1-141 [Domain 8 - Thorax]	68728
143	*"quality of life"/ or ("Quality Of Life" or HRQOL or QOL).ti,ab,kf. [QoL]	385875
144	142 and 143 [Domain 8 AND QoL]	554

Domain 9 - Skin and eye melanoma

1	(rare adj3 (skin or cutan*) adj3 (cancer? or tumo?r? or malignan* or carcinoma?)).ti,ab,kf.	1346
2	"uveal melanoma".rs.	1762
3	(uvea? adj3 melanoma?).ti,ab,kf.	5516
4	"iridociliary melanoma?".ti,ab,kf.	23
5	"irido-ciliary melanoma?".ti,ab,kf.	3
6	(melanoma? adj3 eye?).ti,ab,kf.	635
7	"choroid melanoma?".ti,ab,kf.	142
8	"choroidal melanoma?".ti,ab,kf.	2567
9	(melanoma? adj3 ciliary).ti,ab,kf.	435
10	(melanoma? adj3 iris).ti,ab,kf.	505
11	(melanoma? adj3 conjunctiva).ti,ab,kf.	176
12	"merkel cell carcinoma"/	3041
13	("merkel cell" adj1 (carcinoma? or tumo?r? or cancer?)).ti,ab,kf.	4016
14	"carcinoma, neuroendocrine"/ and exp skin/	12
15	"neuroendocrine carcinoma of the skin".ti,ab,kf.	190
16	"cutaneous neuroendocrine carcinoma?".ti,ab,kf.	136
17	"trabecular cancer?".ti,ab,kf.	6
18	"cutaneous apudoma?".ti,ab,kf.	3
19	exp "mucous membrane"/ and melanoma/	955
20	"mucosal melanoma?".ti,ab,kf.	1288
21	(mucous adj3 melanoma?).ti,ab,kf.	66
22	(extracutaneous adj3 melanoma?).ti,ab,kf.	56
23	"carcinoma, basal cell"/ and exp skin/	1896
24	("basal cell carcinoma?" adj3 (skin or cutan* or dermal)).ti,ab,kf.	1463
25	"basosquamous carcinoma?".ti,ab,kf. and exp skin/	6
26	("basosquamous carcinoma?" adj3 (skin or cutan* or dermal)).ti,ab,kf.	13
27	"squamous carcinoma?".ti,ab,kf. and exp skin/	154
28	("squamous carcinoma?" adj3 (skin or cutan* or dermal)).ti,ab,kf.	145
29	"Carcinoma, Verrucous"/ and exp skin/	30
30	("verrucous carcinoma?" adj3 (skin or cutan* or dermal)).ti,ab,kf.	49
31	"spindle cell carcinoma?".ti,ab,kf. and exp skin/	13
32	("spindle cell carcinoma?" adj3 (skin or cutan* or dermal)).ti,ab,kf.	7
33	(adenoid adj3 "squamous cell carcinoma?" adj3 (skin or cutan* or dermal)).ti,ab,kf.	7
34	((pseudovascular or "pseudo vascular") adj3 "squamous cell carcinoma?" adj3 (skin or cutan* or dermal)).ti,ab,kf.	6
35	"carcinoma, adenosquamous"/ and exp skin/	4
36	("adenosquamous carcinoma?" adj3 (skin or cutan* or dermal)).ti,ab,kf.	20
37	("adnexal carcinoma?" adj3 (skin or cutan* or dermal)).ti,ab,kf.	79
38	("nodular hidradenoma" adj3 malignant adj3 (skin or cutan* or dermal)).ti,ab,kf.	1
39	"adenocarcinoma, sebaceous"/	532
40	"sebaceous adenocarcinoma?".ti,ab,kf.	46

41	"carcinoma, adenoid cystic"/ and exp skin/	106
42	("adenoid cystic carcinoma?" adj3 (skin or cutan* or dermal)).ti,ab,kf.	125
43	"paget disease, extramammary"/ and exp skin/	195
44	("paget disease" adj3 (skin or cutan* or dermal)).ti,ab,kf.	27
45	"apocrine adenocarcinoma?".ti,ab,kf. and exp skin/	42
46	("apocrine adenocarcinoma?" adj3 (skin or cutan* or dermal)).ti,ab,kf.	18
47	"adenocarcinoma, mucinous"/ and exp skin/	79
48	("mucinous adenocarcinoma?" adj3 (skin or cutan* or dermal)).ti,ab,kf.	23
49	"pillomatrix carcinoma?".ti,ab,kf. and exp skin/	0
50	("pillomatrix carcinoma?" adj3 (skin or cutan* or dermal)).ti,ab,kf.	0
51	"eccrine porocarcinoma"/	179
52	"eccrine porocarcinoma".ti,ab,kf.	290
53	("mixed tumo?r?" adj3 malignant).ti,ab,kf. and exp skin/	9
54	("mixed tumo?r?" adj3 malignant adj3 (skin or cutan* or dermal)).ti,ab,kf.	22
55	(sclerosing adj3 "sweat duct?" adj3 carcinoma?).ti,ab,kf.	41
56	("eccrine spiradenoma" adj3 malignant).ti,ab,kf.	58
57	("tubular adenocarcinoma?" adj3 (skin or cutan* or dermal)).ti,ab,kf.	2
58	(eccrine adj3 papillary adj3 adenocarcinoma?).ti,ab,kf.	1
59	"superficial spreading".ti,ab,kf. and exp melanoma/	965
60	("superficial spreading" adj3 melanoma?).ti,ab,kf.	895
61	nodular.ti,ab,kf. and exp melanoma/	1434
62	(nodular adj3 melanoma?).ti,ab,kf.	1006
63	"lentigo maligna".ti,ab,kf. and exp melanoma/	1079
64	("lentigo maligna" adj3 melanoma?).ti,ab,kf.	717
65	("acral lentiginous" adj3 malignan*).ti,ab,kf. and exp melanoma/	22
66	("acral lentiginous" adj3 malignan* adj3 melanoma?).ti,ab,kf.	27
67	"epithelial tumo?r?".ti,ab,kf. and exp eye/	114
68	("epithelial tumo?r?" adj3 (eye? or adnexa?)).ti,ab,kf.	3
69	exp "carcinoma, squamous cell"/ and exp eye/	776
70	(("squamous cell carcinoma?" or SCC) adj3 (eye? or adnexa?)).ti,ab,kf.	85
71	"squamous carcinoma?".ti,ab,kf. and exp eye/	22
72	("squamous carcinoma?" adj3 (eye? or adnexa?)).ti,ab,kf.	4
73	"carcinoma, basal cell"/ and exp eye/	608
74	("basal cell carcinoma?" adj3 (eye? or adnexa?)).ti,ab,kf.	43
75	exp adenocarcinoma/ and exp eye/	1081
76	(adenocarcinoma? adj3 (eye? or adnexa?)).ti,ab,kf.	61
77	"carcinoma, adenoid cystic"/ and exp eye/	148
78	("adenoid cystic carcinoma?" adj3 (eye? or adnexa?)).ti,ab,kf.	7
79	"carcinoma, mucoepidermoid"/ and exp eye/	31
80	("mucoepidermoid carcinoma?" adj3 (eye? or adnexa?)).ti,ab,kf.	2
81	"embryonal tumo?r?".ti,ab,kf. and exp eye/	3
82	("embryonal tumo?r?" adj3 eye?).ti,ab,kf.	3
83	retinoblastoma/	8093
84	retinoblastoma?.ti,ab,kf.	17944
85	or/1-84 [Domain 9 - Skin and eye melanoma]	44640
86	*"quality of life"/ or ("Quality Of Life" or HRQOL or QOL).ti,ab,kf. [QoL]	386175
87	85 and 86 [Domain 9 AND QoL]	245

Domain 10 – Brain and Spinal Cords

1	(rare adj3 (brain or "spinal cord" or "nervous system") adj3 (cancer? or tumo?r? or malignan* or carcinoma?)).ti,ab,kf.	870
2	exp glioma/	97116
3	(neuroglial adj3 tumo?r?).ti,ab,kf.	96
4	"glioneural tumo?r?".ti,ab,kf.	26
5	"neuronal-glial tumo?r?".ti,ab,kf.	85
6	glioma?.ti,ab,kf.	68983
7	(glial adj3 tumo?r?).ti,ab,kf.	3077
8	astrocytoma?.ti,ab,kf.	17709
9	astroglioma?.ti,ab,kf.	243
10	oligoastrocytoma?.ti,ab,kf.	932
11	glioblastoma?.ti,ab,kf.	48224
12	"pleomorphic xanthoastrocytoma?".ti,ab,kf.	632
13	ependymoma?.ti,ab,kf.	6003
14	ependymoblastoma?.ti,ab,kf.	296
15	subependymoma?.ti,ab,kf.	423
16	"ependymal tumo?r?".ti,ab,kf.	215
17	ganglioglioma?.ti,ab,kf.	1567
18	gliosarcoma?.ti,ab,kf.	1227
19	oligodendroglioma?.ti,ab,kf.	4616
20	oligodendrocytoma?.ti,ab,kf.	16
21	(astrocytic adj3 tumo?r?).ti,ab,kf.	1677
22	astroblastoma?.ti,ab,kf.	212
23	"gliomatosis cerebri".ti,ab,kf.	395
24	(oligodendroglioma? adj3 tumo?r?).ti,ab,kf.	426
25	"choroid plexus carcinoma".rs.	82
26	"choroid plexus carcinoma?".ti,ab,kf.	433
27	("meningioma?" adj3 malignan*).ti,ab,kf.	1097
28	pinealoma/	1967
29	pinealoma?.ti,ab,kf.	363
30	(tumo?r? adj3 pineal).ti,ab,kf.	1621
31	pineoblastoma?.ti,ab,kf.	488
32	"embryonal tumo?r?".ti,ab,kf. and exp "central nervous system"/	106
33	("embryonal tumo?r?" adj3 ("central nervous system" or CNS)).ti,ab,kf.	167
34	"teratoid tumor, atypical".rs.	100
35	"atypical teratoid tumo?r?".ti,ab,kf.	15
36	neuroepithelioma?.ti,ab,kf.	621
37	medulloblastoma/ and exp adult/	1940
38	((medulloblastoma? or "medullo blastoma?") and (adult? or aged or elderly or postpubertal or "post pubertal")).ti,ab,kf.	1347
39	or/1-38 [Domain 10 - Brain and spinal cords]	139224
40	*"quality of life"/ or ("Quality Of Life" or HRQOL or QOL).ti,ab,kf. [QoL]	385875
41	39 and 40 [domain 10 AND QoL]	2123

Embase.com

Domain 0 – General Terms

No.	Query	Results

1	'rare cancer'/exp OR ('rare diseases'/exp AND 'malignant neoplasm'/exp) OR ((rare NEAR/5 (cancer$ OR tumor* OR tumour* OR malignan* OR neoplas*)):ti,ab,kw)	142085
2	'quality of life'/exp/mj OR 'quality of life':ti,ab,kw OR hrqol:ti,ab,kw OR qol:ti,ab,kw	574615
3	#1 AND #2	1854
4	#3 NOT ('conference abstract'/it OR 'conference review'/it)	1127
5	#3 AND ('conference abstract'/it OR 'conference review'/it)	727

Domain 1 – Sarcoma

No.	Query	Results
1	'sarcoma'/exp OR sarcoma$:ti,ab,kw	272890
2	'adenosarcoma'/de OR adenosarcoma$:ti,ab,kw OR 'carcinosarcoma'/de OR carcinosarcoma$:ti,ab,kw OR 'desmoplastic small round cell tumor'/de OR 'desmoplastic small round cell tumo$r$':ti,ab,kw OR 'desmoplastic small cell tumo$r$':ti,ab,kw OR dsrct$:ti,ab,kw OR 'endometrial stromal tumor'/de OR 'endometrial stromal tumo$r$':ti,ab,kw OR 'endolymphatic stromal myos$s':ti,ab,kw OR 'fibrosarcoma'/de OR fibrosarcoma$:ti,ab,kw OR 'dermatofibrosarcoma protuberans'/exp OR dermatofibrosarcoma$:ti,ab,kw OR dfsp:ti,ab,kw OR 'neurofibrosarcoma'/de OR neurofibrosarcoma$:ti,ab,kw OR 'angiosarcoma'/exp OR hemangiosarcoma$:ti,ab,kw OR angiosarcoma$:ti,ab,kw OR 'malignant fibrous histiocytoma'/exp OR 'malignant fibrous histiocytoma$':ti,ab,kw OR 'malignant fibrohistiocytic tumo$r$':ti,ab,kw OR 'leiomyosarcoma'/exp OR leiomyosarcoma$:ti,ab,kw OR 'liposarcoma'/exp OR liposarcoma$:ti,ab,kw OR 'atypical lipomatous tumo$r$':ti,ab,kw OR lymphangiosarcoma$:ti,ab,kw OR 'mesodermal mixed tumo$r$':ti,ab,kw OR 'myosarcoma'/exp OR myosarcoma$:ti,ab,kw OR 'rhabdomyosarcoma'/exp OR rhabdomyosarcoma$:ti,ab,kw OR 'myxosarcoma'/de OR myxosarcoma$:ti,ab,kw OR 'phyllodes tumor'/de OR 'phyllodes tumo$r$':ti,ab,kw OR fibromyxosarcoma$:ti,ab,kw OR gliosarcoma$:ti,ab,kw OR leukosarcoma$:ti,ab,kw OR 'stewart-treves syndrome':ti,ab,kw OR lymphosarcoma$:ti,ab,kw OR reticulosarcoma$:ti,ab,kw OR 'rhabdoid tumo$r$':ti,ab,kw OR 'fibroblastic sarcoma$':ti,ab,kw OR 'epithelioid hemangioendothelioma'/exp OR 'epithelioid hemangioendothelioma$':ti,ab,kw OR 'malignant peripheral nerve sheath tumo$r$':ti,ab,kw OR glomangiosarcoma$:ti,ab,kw OR 'malignant glomus tumo$r$':ti,ab,kw OR 'perivascular epithelioid cell tumor'/de OR 'perivascular epithelioid cell tumo$r$':ti,ab,kw OR pecoma$:ti,ab,kw OR 'ewing sarcoma'/de OR 'ewing sarcoma$':ti,ab,kw OR esft:ti,ab,kw OR 'peripheral neuroectodermal tumo$r$':ti,ab,kw OR 'primitive neuroectodermal tumo$r$':ti,ab,kw OR ppnet:ti,ab,kw OR medulloepithelioma$:ti,ab,kw	142187
3	'gastrointestinal stromal tumor'/exp OR (((gastrointestinal OR gastric OR gi) NEAR/1 stroma$ NEAR/1 (tumo$r$ OR neoplas* OR sarcoma$)):ti,ab,kw) OR gist$:ti,ab,kw OR 'carney triad$':ti,ab,kw OR 'carney-stratakis syndrome':ti,ab,kw OR 'carney-stratakis dyad$':ti,ab,kw	26139
4	'osteosarcoma'/de OR osteosarcoma$:ti,ab,kw OR 'osteogenic sarcoma$':ti,ab,kw OR 'bone sarcoma$':ti,ab,kw OR 'chondrosarcoma'/exp OR chondrosarcoma$:ti,ab,kw OR 'osteoclastoma'/de OR (('giant cell tumo$r$' NEAR/3 bone$):ti,ab,kw) OR gctb:ti,ab,kw OR osteoclastoma$:ti,ab,kw OR 'chordoma'/de OR chordoma$:ti,ab,kw OR 'notochordal sarcoma$':ti,ab,kw	74766

5	#1 OR #2 OR #3 OR #4	340515
6	'quality of life'/exp/mj OR 'quality of life':ti,ab,kw OR hrqol:ti,ab,kw OR qol:ti,ab,kw	574615
7	#5 AND #6	3604
8	#7 NOT ('conference abstract'/it OR 'conference review'/it)	2612
9	#7 AND ('conference abstract'/it OR 'conference review'/it)	992

Domain 2 - Female genital organs and placenta

No.	Query	Results
1	(rare NEAR/3 ('female genital' OR fallopian OR uterine OR vaginal OR vulvar) NEAR/3 (cancer$ OR tumo$r$ OR malignan*)):ti,ab,kw	902
2	(('non epithelial' NEAR/2 (ovarian OR ovary) NEAR/2 (cancer$ OR tumo$r$ OR malignan*)):ti,ab,kw) OR 'ovarian sex cord stromal tumor'/exp OR 'sex cord stromal tumo$r$':ti,ab,kw OR (('sex cord tumo$r$' NEAR/3 ovar*):ti,ab,kw) OR scst:ti,ab,kw OR scco:ti,ab,kw OR sccoht:ti,ab,kw OR 'dysgerminoma'/de OR dysgerminoma$:ti,ab,kw OR 'yolk sac tumor'/de OR 'endodermal sinus tumo$r$':ti,ab,kw OR 'yolk sac tumo$r$':ti,ab,kw OR orchioblastoma$:ti,ab,kw OR 'embryonal carcinoma'/de OR (((embryo OR embryon*) NEAR/1 (carcinoma$ OR adenocarcinoma$ OR 'cell cancer$')):ti,ab,kw) OR 'non-gestational choriocarcinoma'/de OR 'non-gestational choriocarcinoma$':ti,ab,kw OR 'nongestational choriocarcinoma$':ti,ab,kw OR 'teratoma'/exp OR 'mature teratoma$':ti,ab,kw OR teratosarcoma$:ti,ab,kw OR 'immature teratoma$':ti,ab,kw OR 'mixed germ cell tumo$r$':ti,ab,kw OR 'fibroma'/exp OR fibroma$:ti,ab,kw OR 'thecoma'/de OR thecoma$:ti,ab,kw OR hemangiofibroma$:ti,ab,kw OR 'fibrosarcoma'/exp OR fibrosarcoma$:ti,ab,kw OR 'fibroblastic sarcoma':ti,ab,kw OR 'sclerosing stromal tumo$r$':ti,ab,kw OR 'signet ring stromal tumo$r$':ti,ab,kw OR 'microcystic stromal tumo$r$':ti,ab,kw OR 'leydig cell tumor'/de OR 'leydig cell tumo$r$':ti,ab,kw OR 'steroid cell tumo$r$':ti,ab,kw OR 'granulosa cell tumor'/exp OR 'granulosa cell tumo$r$':ti,ab,kw OR 'granulosa cell carcinoma$':ti,ab,kw OR 'granulosa cancer$':ti,ab,kw OR 'sertoli cell tumor'/de OR 'sertoli cell tumo$r$':ti,ab,kw OR 'androblastoma'/de OR 'sertoli-leydig cell tumo$r$':ti,ab,kw OR androblastoma$:ti,ab,kw OR ('endometrioid carcinoma'/de AND 'uterus'/exp) OR (('endometrioid adenocarcinoma$' NEAR/3 uter*):ti,ab,kw) OR ('endometrioid adenocarcinoma$':ti,ab,kw AND 'uterus'/exp) OR ('adenosquamous carcinoma'/de AND 'uterus'/exp) OR (('adenosquamous carcinoma$' NEAR/3 uter*):ti,ab,kw) OR ('colloid carcinoma'/de AND 'uterus'/exp) OR (('mucinous adenocarcinoma$' NEAR/3 uter*):ti,ab,kw) OR (('mixed cell adenocarcinoma$' NEAR/3 uter*):ti,ab,kw) OR ('mixed cell adenocarcinoma$':ti,ab,kw AND 'uterus'/exp) OR 'villous adenocarcinoma$':ti,ab,kw OR 'brenner tumor'/exp OR 'brenner tumo$r$':ti,ab,kw OR ('transitional cell carcinoma'/exp AND 'uterus'/exp) OR (('transitional cell carcinoma$' NEAR/3 uter*):ti,ab,kw) OR (('basaloid carcinoma$' NEAR/3 uter*):ti,ab,kw) OR ('basaloid carcinoma$':ti,ab,kw AND 'uterus'/exp) OR ('squamous cell carcinoma'/exp AND 'uterus'/exp) OR ((('squamous cell carcinoma$' OR scc) NEAR/3 uter*):ti,ab,kw) OR ('adenoid cystic carcinoma'/exp AND 'uterus'/exp) OR (('adenoid cystic carcinoma$' NEAR/3 uter*):ti,ab,kw) OR (('clear cell adenocarcinoma$' NEAR/3 uter*):ti,ab,kw) OR ((serous NEAR/3 carcinoma$):ti,ab,kw) OR 'mixed mullerian	183107

	tumor'/de OR 'mullerian mixed tumo$r$':ti,ab,kw OR ('squamous cell carcinoma'/exp AND 'uterine cervix'/exp) OR ((('squamous cell carcinoma$' OR scc) NEAR/3 cervi*):ti,ab,kw) OR cscc:ti,ab,kw OR ('verrucous carcinoma'/de AND 'uterine cervix'/exp) OR (((verrucous OR warty) NEAR/1 carcinoma$ NEAR/3 cervi*):ti,ab,kw) OR (('basaloid carcinoma$' NEAR/3 cervi*):ti,ab,kw) OR ('basaloid carcinoma$':ti,ab,kw AND 'uterine cervix'/exp) OR (('spindle cell' NEAR/3 carcinoma$ NEAR/3 cervi*):ti,ab,kw) OR ('spindle cell carcinoma'/exp AND 'uterine cervix'/exp) OR (('lymphoepithelial carcinoma$' NEAR/3 cervi*):ti,ab,kw) OR ('lymphoepithelioma'/de AND 'uterine cervix'/exp) OR ('transitional cell carcinoma'/de AND 'uterine cervix'/exp) OR (('transitional cell carcinoma$' NEAR/3 cervi*):ti,ab,kw) OR (('glassy cell carcinoma$' NEAR/3 cervi*):ti,ab,kw) OR ('glassy cell carcinoma$':ti,ab,kw AND 'uterine cervix'/exp) OR 'uterine cervix adenocarcinoma'/exp OR ((adenocarcinoma$ NEAR/3 cervi*):ti,ab,kw) OR (('serous cystadenocarcinoma$' NEAR/3 cervi*):ti,ab,kw) OR ('serous cystadenocarcinoma$':ti,ab,kw AND 'uterine cervix'/exp) OR (('undifferentiated carcinoma$' NEAR/3 cervi*):ti,ab,kw) OR ('anaplastic carcinoma'/de AND 'uterine cervix'/exp) OR ('signet ring carcinoma'/de AND 'uterine cervix'/exp) OR ('signet ring cell carcinoma$':ti,ab,kw AND cervi*:ti,ab,kw) OR ('mesonephroma'/de AND 'uterine cervix'/exp) OR (mesonephroma$:ti,ab,kw AND cervi*:ti,ab,kw) OR 'ovary adenocarcinoma'/exp OR ((adenocarcinoma$ NEAR/3 ovar*):ti,ab,kw) OR (('serous cystadenocarcinoma$' NEAR/3 ovar*):ti,ab,kw) OR ('serous cystadenocarcinoma$':ti,ab,kw AND 'ovary'/exp) OR ((endometrioid* NEAR/1 adenofibroma$ NEAR/3 ovar*):ti,ab,kw) OR (endometrioid*:ti,ab,kw AND 'adenofibroma'/de AND 'ovary'/exp) OR adenocarcinofibroma$:ti,ab,kw OR ('transitional cell carcinoma'/de AND 'ovary'/exp) OR (('transitional cell carcinoma$' NEAR/3 ovar*):ti,ab,kw) OR (('basaloid carcinoma$' NEAR/3 ovar*):ti,ab,kw) OR ('basaloid carcinoma$':ti,ab,kw AND 'ovary'/exp) OR 'ovarian mucinous adenocarcinoma'/exp OR (('mucinous adenocarcinoma$' NEAR/3 ovar*):ti,ab,kw) OR ('colloid carcinoma'/de AND 'ovary'/exp) OR (('clear cell adenocarcinoma$' NEAR/3 ovar*):ti,ab,kw) OR ((peritoneal NEAR/3 (serous OR papillary) NEAR/3 carcinoma$):ti,ab,kw) OR ('adenocarcinoma'/exp AND 'fallopian tube'/exp) OR ((adenocarcinoma$ NEAR/3 ('fallopian tube$' OR oviduct$)):ti,ab,kw) OR (('serous cystadenocarcinoma$' NEAR/3 ('fallopian tube$' OR oviduct$)):ti,ab,kw) OR ('serous cystadenocarcinoma$':ti,ab,kw AND 'fallopian tube'/exp) OR 'struma ovarii'/de OR 'struma ovarii':ti,ab,kw OR ('germ cell and embryonal neoplasms'/exp AND 'ovary'/exp) OR (('germ cell tumo$r$' NEAR/3 ovar*):ti,ab,kw)	
3	'gestational trophoblastic neoplasia'/exp OR 'gestational trophoblastic disease$':ti,ab,kw OR gtd:ti,ab,kw OR 'gestational trophoblastic neoplas*':ti,ab,kw OR 'invasive mole$':ti,ab,kw OR 'hydatidiform mole'/exp OR 'hydatidiform mole$':ti,ab,kw OR 'molar pregnanc*':ti,ab,kw OR 'partial mole$':ti,ab,kw OR 'choriocarcinoma'/exp OR choriocarcinoma$:ti,ab,kw OR 'placental site trophoblastic tumo$r$':ti,ab,kw OR pstt:ti,ab,kw OR 'epithelioid trophoblastic tumo$r$':ti,ab,kw OR ((polyembryoma$ NEAR/3 ovar*):ti,ab,kw) OR (polyembryoma$:ti,ab,kw AND 'ovary'/exp) OR (('epithelial tumo$r$' NEAR/3 (vulva OR vagina$)):ti,ab,kw) OR ('epithelial tumo$r$':ti,ab,kw AND 'vagina'/exp) OR ('squamous cell carcinoma'/exp AND 'vagina'/exp) OR (('squamous cell carcinoma$' NEAR/3 (vulva OR vagina$)):ti,ab,kw) OR ('adenocarcinoma'/exp AND 'vagina'/exp) OR ((adenocarcinoma$ NEAR/3 (vulva OR vagina$)):ti,ab,kw) OR ('paget skin disease'/de AND 'vagina'/exp) OR (('paget disease' NEAR/3 (vulva OR	25363

	vagina$)):ti,ab,kw) OR (('undifferentiated carcinoma$' NEAR/3 (vulva OR vagina$)):ti,ab,kw) OR ('anaplastic carcinoma'/de AND 'vagina'/exp)	
4	#1 OR #2 OR #3	205525
5	'quality of life'/exp/mj OR 'quality of life':ti,ab,kw OR hrqol:ti,ab,kw OR qol:ti,ab,kw	574615
6	#4 AND #5	1567
7	#6 NOT ('conference abstract'/it OR 'conference review'/it)	1104
8	#6 AND ('conference abstract'/it OR 'conference review'/it)	463

Domain 3 - Male genital organs and the urinary tract

No.	Query	Results
1	(rare NEAR/3 ('male genital' OR testicular OR 'urinary tract' OR penile OR prostat*) NEAR/3 (cancer$ OR tumo$r$ OR malignan*)):ti,ab,kw	1559
2	'acinar cell carcinoma'/de AND 'prostate'/exp OR (('acinar cell carcinoma$' NEAR/3 prostate$):ti,ab,kw) OR ('signet ring carcinoma'/de AND 'prostate'/exp) OR ('signet ring cell carcinoma$':ti,ab,kw AND prostate$:ti,ab,kw) OR ((adenocarcinoma$ NEAR/3 'neuroendocrine differentiation' NEAR/3 prostate$):ti,ab,kw) OR (((adenocarcinoma$ NEAR/3 'neuroendocrine differentiation'):ti,ab,kw) AND 'prostate'/exp) OR (('oxyphilic adenocarcinoma$' NEAR/3 prostate$):ti,ab,kw) OR ('oxyphilic adenocarcinoma$' AND 'prostate'/exp) OR (('spindle cell' NEAR/3 carcinoma$ NEAR/3 prostate$):ti,ab,kw) OR ('spindle cell carcinoma'/exp AND 'prostate'/exp) OR (('lymphoepithelial carcinoma$' NEAR/3 prostate$):ti,ab,kw) OR ('lymphoepithelioma'/de AND 'prostate'/exp) OR ('squamous cell carcinoma'/exp AND 'prostate'/exp) OR (('squamous cell carcinoma$' NEAR/3 prostate$):ti,ab,kw) OR (('squamous carcinoma$' NEAR/3 prostate$):ti,ab,kw) OR ('adenosquamous carcinoma'/de AND 'prostate'/exp) OR (('adenosquamous carcinoma$' NEAR/3 prostate$):ti,ab,kw) OR (('basaloid carcinoma$' NEAR/3 prostate$):ti,ab,kw) OR ('basaloid carcinoma$':ti,ab,kw AND 'prostate'/exp) OR (('infiltrating duct carcinoma$' NEAR/3 prostate$):ti,ab,kw) OR ('invasive ductal carcinoma'/exp AND 'prostate'/exp) OR (('cribriform carcinoma$' NEAR/3 prostate$):ti,ab,kw) OR ('cribriform carcinoma$':ti,ab,kw AND 'prostate'/exp) OR (('solid carcinoma$' NEAR/3 prostate$):ti,ab,kw) OR ('solid carcinoma$':ti,ab,kw AND 'prostate'/exp) OR (('papillary adenocarcinoma$' NEAR/3 prostate):ti,ab,kw) OR ('papillary adenocarcinoma$':ti,ab,kw AND 'prostate'/exp) OR ('transitional cell carcinoma'/exp AND 'prostate'/exp) OR (('transitional cell carcinoma$' NEAR/3 prostate$):ti,ab,kw) OR (('basal cell adenocarcinoma$' NEAR/3 prostate$):ti,ab,kw) OR ('basal cell adenocarcinoma$':ti,ab,kw AND 'prostate'/exp) OR ('adenoid cystic carcinoma'/de AND 'prostate'/exp) OR (('adenoid cystic carcinoma$' NEAR/3 prostate$):ti,ab,kw) OR 'paratesticular adenocarcinoma$':ti,ab,kw OR (('endometrioid adenocarcinoma$' NEAR/3 prostate$):ti,ab,kw) OR ('endometrioid adenocarcinoma$':ti,ab,kw AND 'prostate'/exp) OR (('clear cell adenocarcinoma$' NEAR/3 prostate$):ti,ab,kw) OR ('clear cell adenocarcinoma$':ti,ab,kw AND 'prostate'/exp) OR ('cystadenocarcinoma'/de AND 'prostate'/exp) OR (('serous cystadenocarcinoma$' NEAR/3 prostate$):ti,ab,kw) OR ('colloid carcinoma'/de AND 'prostate'/exp) OR (('mucinous adenocarcinoma$' NEAR/3 prostate$):ti,ab,kw) OR 'rete testis'/exp	38636

	OR 'rete testis':ti,ab,kw OR (('collecting duct carcinoma$' NEAR/3 prostate$):ti,ab,kw) OR ('collecting duct carcinoma$':ti,ab,kw AND 'prostate'/exp)	
3	'seminoma'/exp OR seminoma*:ti,ab,kw OR 'non-seminoma*':ti,ab,kw OR ('germ cell and embryonal neoplasms'/exp AND 'testis tumor'/exp) OR 'testicular germ cell tumo$r$':ti,ab,kw OR 'mixed germ cell tumo$r$':ti,ab,kw OR 'teratocarcinoma'/de OR teratocarcinoma$:ti,ab,kw OR 'malignant teratoma$':ti,ab,kw OR (('sex cord cancer$' NEAR/3 (testes OR testi*)):ti,ab,kw) OR ('sex cord cancer$':ti,ab,kw AND 'testis'/exp) OR (('gonadal stromal tumo$r$' NEAR/3 (testes OR testi*)):ti,ab,kw) OR ('gonadal stromal tumo$r$':ti,ab,kw AND 'testis'/exp) OR 'sertoli cell tumor'/de OR 'sertoli cell carcinoma$':ti,ab,kw OR (('epithelial tumo$r$' NEAR/3 peni*):ti,ab,kw) OR ('epithelium tumor'/exp AND 'penis'/exp) OR ('carcinoma, squamous cell'/exp AND 'penis'/exp) OR ((('squamous cell carcinoma$' OR scc) NEAR/3 peni*):ti,ab,kw) OR ('carcinoma, adenosquamous'/de AND 'penis'/exp) OR (('adenosquamous carcinoma$' NEAR/3 peni*):ti,ab,kw) OR ('verrucous carcinoma'/de AND 'penis'/exp) OR (('verrucous carcinoma$' NEAR/3 peni*):ti,ab,kw) OR (('basaloid carcinoma$' NEAR/3 peni*):ti,ab,kw) OR ('basaloid carcinoma$':ti,ab,kw AND 'penis'/exp) OR ('adenocarcinoma'/exp AND 'penis'/exp) OR ((adenocarcinoma$ NEAR/3 peni*):ti,ab,kw) OR ('paget skin disease'/de AND 'penis'/exp) OR (('paget disease' NEAR/3 peni*):ti,ab,kw) OR (('mixed tumo$r$' NEAR/3 peni*):ti,ab,kw) OR ('mixed tumo$r$' AND 'penis'/exp) OR 'renal cell adenocarcinoma$':ti,ab,kw OR ('clear cell adenocarcinoma$' AND 'kidney'/exp) OR (('clear cell adenocarcinoma$' NEAR/3 (kidney OR renal)):ti,ab,kw) OR ('papillary adenocarcinoma$':ti,ab,kw AND 'kidney'/exp) OR (('papillary adenocarcinoma$' NEAR/3 (kidney OR renal)):ti,ab,kw) OR 'chromophobe renal cell carcinoma'/exp OR (('renal cell carcinoma$' NEAR/3 chromophobe$):ti,ab,kw) OR (('collecting duct carcinoma$' NEAR/3 (kidney OR renal)):ti,ab,kw) OR ('collecting duct carcinoma'/exp AND 'kidney'/exp) OR ('carcinoma'/exp AND 'neuroblastoma'/exp) OR ((carcinoma$ NEAR/3 neuroblastoma$):ti,ab,kw) OR ('medullary carcinoma'/de AND 'kidney'/exp) OR (('medullary carcinoma$' NEAR/3 (kidney OR renal)):ti,ab,kw) OR ((tubular NEAR/3 carcinoma$ NEAR/3 (kidney OR renal)):ti,ab,kw) OR (('spindle cell carcinoma$' NEAR/3 (kidney OR renal)):ti,ab,kw) OR ('spindle cell carcinoma'/exp AND 'kidney'/exp) OR ('squamous cell carcinoma'/exp AND 'kidney'/exp) OR (('squamous cell carcinoma$' NEAR/3 kidney):ti,ab,kw) OR (('epithelial tumo$r$' NEAR/3 ('renal pelvis' OR ureter$)):ti,ab,kw) OR ('epithelium tumor'/exp AND ('kidney pelvis'/exp OR 'ureter'/exp)) OR ('carcinoma'/exp AND ('kidney pelvis'/exp OR 'ureter'/exp)) OR ((carcinoma$ NEAR/3 ('kindey pelvis' OR 'renal pelvis' OR ureter$)):ti,ab,kw) OR ('squamous cell carcinoma'/exp AND ('kidney pelvis'/exp OR 'ureter'/exp)) OR (('squamous cell carcinoma$' NEAR/3 ('kindey pelvis' OR 'renal pelvis' OR ureter$)):ti,ab,kw) OR (('basaloid carcinoma$' NEAR/3 ('kindey pelvis' OR 'renal pelvis' OR ureter$)):ti,ab,kw) OR ('basaloid carcinoma$':ti,ab,kw AND ('kidney pelvis'/exp OR 'ureter'/exp)) OR ('verrucous carcinoma'/de AND ('kidney pelvis'/exp OR 'ureter'/exp)) OR (('verrucous carcinoma$' NEAR/3 ('kindey pelvis' OR 'renal pelvis' OR ureter$)):ti,ab,kw) OR ('adenocarcinoma'/exp AND ('kidney pelvis'/exp OR 'ureter'/exp)) OR ((adenocarcinoma$ NEAR/3 ('kindey pelvis' OR 'renal pelvis' OR ureter$)):ti,ab,kw) OR ('signet ring carcinoma'/de AND ('kidney pelvis'/exp OR 'ureter'/exp)) OR ('signet ring cell carcinoma$':ti,ab,kw AND ('kindey pelvis':ti,ab,kw OR 'renal pelvis':ti,ab,kw OR ureter$:ti,ab,kw)) OR ('adenoid cystic carcinoma'/de AND ('kidney pelvis'/exp OR 'ureter'/exp)) OR (('adenoid cystic carcinoma$' NEAR/3 ('kindey pelvis' OR 'renal pelvis' OR ureter$)):ti,ab,kw) OR (('epithelial tumo$r$' NEAR/3 urethra$):ti,ab,kw) OR ('epithelium tumor'/exp	70933

	AND 'urethra'/exp) OR ('carcinoma'/exp AND 'urethra'/exp) OR ((carcinoma$ NEAR/3 urethra$):ti,ab,kw) OR (('squamous cell carcinoma$' NEAR/3 urethra$):ti,ab,kw) OR (('basaloid carcinoma$' NEAR/3 urethra$):ti,ab,kw) OR ('basaloid carcinoma$':ti,ab,kw AND 'urethra'/exp) OR ('verrucous carcinoma'/de AND 'urethra'/exp) OR (('verrucous carcinoma$' NEAR/3 urethra$):ti,ab,kw) OR ('adenocarcinoma'/exp AND 'urethra'/exp) OR ((adenocarcinoma$ NEAR/3 urethra$):ti,ab,kw) OR ('signet ring carcinoma'/de AND 'urethra'/exp) OR ('signet ring cell carcinoma$':ti,ab,kw AND urethra$:ti,ab,kw) OR ('adenoid cystic carcinoma'/de AND 'urethra'/exp) OR (('adenoid cystic carcinoma$' NEAR/3 urethra$):ti,ab,kw) OR (('undifferentiated carcinoma$' NEAR/3 bladder):ti,ab,kw) OR ('anaplastic carcinoma'/de AND 'bladder'/exp) OR 'transitional cell carcinoma of the bladder'/de OR (('transitional cell carcinoma$' NEAR/3 bladder):ti,ab,kw) OR (('lymphoepithelial carcinoma$' NEAR/3 bladder):ti,ab,kw) OR ('lymphoepithelioma'/exp AND 'bladder'/exp) OR ('giant cell carcinoma'/de AND 'bladder'/exp) OR (('giant cell carcinoma$' NEAR/3 bladder):ti,ab,kw) OR (('squamous cell carcinoma$' NEAR/3 bladder):ti,ab,kw) OR (('basaloid carcinoma$' NEAR/3 bladder):ti,ab,kw) OR ('basaloid carcinoma$':ti,ab,kw AND 'bladder'/exp) OR ('verrucous carcinoma'/de AND 'bladder'/exp) OR (('verrucous carcinoma$' NEAR/3 bladder):ti,ab,kw) OR 'adenocarcinoma of the bladder'/exp OR ((adenocarcinoma$ NEAR/3 bladder):ti,ab,kw) OR ('signet ring carcinoma'/de AND 'bladder'/exp) OR (('signet ring cell carcinoma$' NEAR/3 bladder):ti,ab,kw) OR ('adenoid cystic carcinoma'/exp AND 'bladder'/exp) OR (('adenoid cystic carcinoma$' NEAR/3 bladder):ti,ab,kw) OR ('mucoepidermoid tumor'/exp AND 'bladder'/exp) OR (('mucoepidermoid carcinoma$' NEAR/3 bladder):ti,ab,kw) OR 'extragonadal germ cell tumo$r$':ti,ab,kw OR 'embryonal adenocarcinoma$':ti,ab,kw OR (('germ cell tumo$r$' NEAR/3 ('central nervous system' OR cns)):ti,ab,kw) OR ('germ cell tumo$r$':ti,ab,kw AND 'central nervous system'/exp) OR 'nephroblastoma'/exp OR nephroblastoma$:ti,ab,kw	
4	#1 OR #2 OR #3	109230
5	'quality of life'/exp/mj OR 'quality of life':ti,ab,kw OR hrqol:ti,ab,kw OR qol:ti,ab,kw	574615
6	#4 AND #5	917
7	#6 NOT ('conference abstract'/it OR 'conference review'/it)	613
8	#6 AND ('conference abstract'/it OR 'conference review'/it)	304

Domain 4 - Neuroendocrine

No.	Query	Results
1	(rare NEAR/3 (neuroendocrine OR lung OR thymic OR thymus) NEAR/3 (cancer$ OR tumo$r$ OR malignan$)):ti,ab,kw	4849
2	'pulmonary carcinoid'/exp OR ((lung$ NEAR/3 carcinoid$):ti,ab,kw) OR 'pulmonary carcinoid$':ti,ab,kw OR 'pulmonary neuroendocrine tumo$r$':ti,ab,kw OR 'thymic carcinoid'/exp OR 'thymic carcinoid$':ti,ab,kw OR ((thymus NEAR/3 carcinoid$):ti,ab,kw) OR ('neuroendocrine tumor'/de AND 'lung'/exp) OR ((('neuroendocrine tumo$r$' OR net) NEAR/3 lung$):ti,ab,kw) OR ('neuroendocrine tumor'/de AND (gastroenteropancreatic:ti,ab,kw OR 'gastro entero pancreatic':ti,ab,kw OR 'gep':ti,ab,kw)) OR ((('neuroendocrine tumo$r$' OR 'net') NEAR/3 (gastroenteropancreatic OR 'gastro entero pancreatic' OR 'gep')):ti,ab,kw) OR ('endocrine carcinoma'/de AND ('pancreas'/exp OR	129315

	'gastrointestinal tract'/exp)) OR (('endocrine carcinoma$' NEAR/3 (pancrea* OR digestive OR gastrointestinal)):ti,ab,kw) OR 'pancreatic carcinoid'/exp OR 'gastrointestinal carcinoid'/exp OR ((carcinoid$ NEAR/3 (pancrea* OR digestive OR gastrointestinal)):ti,ab,kw) OR 'pancreas islet cell carcinoma'/exp OR (('islet cell carcinoma$' NEAR/3 (pancrea* OR digestive OR gastrointestinal)):ti,ab,kw) OR ((insulinoma$ NEAR/3 malignan*):ti,ab,kw) OR 'glucagonoma'/de OR glucagonoma$:ti,ab,kw OR ((somatostatinoma$ NEAR/3 malignan*):ti,ab,kw) OR 'gastrinoma'/exp OR gastrinoma$:ti,ab,kw OR 'vipoma'/exp OR vipoma$:ti,ab,kw OR 'vip oma$':ti,ab,kw OR 'vasoactive intestinal peptide oma$':ti,ab,kw OR (('mixed pancreatic' NEAR/5 tumo$r$):ti,ab,kw) OR 'paraganglioma'/exp OR paraganglioma$:ti,ab,kw OR 'thyroid medullary carcinoma'/exp OR (('medullary carcinoma$' NEAR/3 thyroid*):ti,ab,kw) OR (('follicular carcinoma$' NEAR/3 thyroid*):ti,ab,kw) OR ('endocrine tumor'/de AND 'thyroid gland'/exp) OR (('endocrine tumo$r$' NEAR/3 thyroid*):ti,ab,kw) OR 'neuroendocrine carcinoma'/exp OR 'neuroendocrine carcinoma$':ti,ab,kw OR (('well differentiated' NEAR/3 'endocrine tumo$r$' NEAR/3 carcinoid$):ti,ab,kw) OR (('poorly differentiated' NEAR/3 'endocrine carcinoma$'):ti,ab,kw) OR 'neuroblastoma'/exp OR neuroblastoma$:ti,ab,kw OR ganglioneuroblastoma$:ti,ab,kw	
3	#1 OR #2	132463
4	'quality of life'/exp/mj OR 'quality of life':ti,ab,kw OR hrqol:ti,ab,kw OR qol:ti,ab,kw	574615
5	#3 AND #4	1338
6	#5 NOT ('conference abstract'/it OR 'conference review'/it)	889
7	#5 AND ('conference abstract'/it OR 'conference review'/it)	449

Domain 5 - Digestive Tract

No.	Query	Results
1	(rare NEAR/3 'digestive tract' NEAR/3 (cancer$ OR tumo$r$ OR malignan*)):ti,ab,kw	60
2	'esophageal squamous cell carcinoma'/de OR ((('squamous cell carcinoma$' OR scc) NEAR/3 (esophagus OR oesophagus)):ti,ab,kw) OR (('squamous carcinoma$' NEAR/3 (esophagus OR oesophagus)):ti,ab,kw) OR ('squamous carcinoma$':ti,ab,kw AND 'esophagus'/exp) OR ('adenosquamous carcinoma'/de AND 'esophagus'/exp) OR (('adenosquamous carcinoma$' NEAR/3 (esophagus OR oesophagus)):ti,ab,kw) OR ('verrucous carcinoma'/de AND 'esophagus'/exp) OR (('verrucous carcinoma$' NEAR/3 (esophagus OR oesophagus)):ti,ab,kw) OR 'esophageal adenocarcinoma'/de OR ((adenocarcinoma$ NEAR/3 (esophagus OR oesophagus)):ti,ab,kw) OR ('mucoepidermoid tumor'/de AND 'esophagus'/exp) OR (('mucoepidermoid carcinoma$' NEAR/3 (esophagus OR oesophagus)):ti,ab,kw) OR ('adenoid cystic carcinoma'/exp AND 'esophagus'/exp) OR (('adenoid cystic carcinoma$' NEAR/3 (esophagus OR oesophagus)):ti,ab,kw) OR (('undifferentiated carcinoma$' NEAR/3 (esophagus OR oesophagus)):ti,ab,kw) OR ('anaplastic carcinoma'/de AND 'esophagus'/exp) OR 'stomach adenocarcinoma'/exp OR ((adenocarcinoma$ NEAR/3 (stomach OR gastric)):ti,ab,kw) OR ((adenocarcinoma$ NEAR/3 'intestinal type'):ti,ab,kw) OR ('signet ring carcinoma'/exp AND 'stomach'/exp) OR ('signet ring cell carcinoma$':ti,ab,kw AND (stomach:ti,ab,kw OR gastric:ti,ab,kw)) OR ('colloid carcinoma'/de AND 'stomach'/exp) OR (('mucinous adenocarcinoma$' NEAR/3 (stomach OR gastric)):ti,ab,kw) OR ('adenosquamous carcinoma'/de AND	133349

	'stomach'/exp) OR (('adenosquamous carcinoma$' NEAR/3 (stomach OR gastric)):ti,ab,kw) OR 'squamous cell carcinoma of the stomach'/exp OR ((('squamous cell carcinoma$' OR scc) NEAR/3 (stomach OR gastric)):ti,ab,kw) OR (('squamous carcinoma$' NEAR/3 (stomach OR gastric)):ti,ab,kw) OR ('squamous carcinoma$':ti,ab,kw AND 'stomach'/exp) OR (('basaloid carcinoma$' NEAR/3 (stomach OR gastric)):ti,ab,kw) OR ('basaloid carcinoma$':ti,ab,kw AND 'stomach'/exp) OR ('verrucous carcinoma'/de AND 'stomach'/exp) OR (('verrucous carcinoma$' NEAR/3 (stomach OR gastric)):ti,ab,kw) OR ('mucoepidermoid tumor'/de AND 'stomach'/exp) OR (('mucoepidermoid carcinoma$' NEAR/3 (stomach OR gastric)):ti,ab,kw) OR ('adenoid cystic carcinoma'/exp AND 'stomach'/exp) OR (('adenoid cystic carcinoma$' NEAR/3 (stomach OR gastric)):ti,ab,kw) OR ('anaplastic carcinoma'/exp AND 'stomach'/exp) OR (('undifferentiated carcinoma$' NEAR/3 (stomach OR gastric)):ti,ab,kw) OR ('epithelium tumor'/exp AND 'small intestine'/exp) OR (('epithelial tumo$r$' NEAR/3 'small intestine$'):ti,ab,kw) OR 'intestinal adenocarcinoma'/exp OR ((adenocarcinoma$ NEAR/3 'small intestine$'):ti,ab,kw) OR ('colloid carcinoma'/de AND 'small intestine'/exp) OR (('mucinous adenocarcinoma$' NEAR/3 'small intestine$'):ti,ab,kw) OR ('signet ring carcinoma'/exp AND 'small intestine'/exp) OR ('signet ring cell carcinoma$':ti,ab,kw AND 'small intestine$':ti,ab,kw) OR ('adenosquamous carcinoma'/de AND 'small intestine'/exp) OR (('adenosquamous carcinoma$' NEAR/3 'small intestine$'):ti,ab,kw) OR ('medullary carcinoma'/de AND 'small intestine'/exp) OR (('medullary carcinoma$' NEAR/3 'small intestine$'):ti,ab,kw) OR ('anaplastic carcinoma'/de AND 'small intestine'/exp) OR (('undifferentiated carcinoma$' NEAR/3 'small intestine$'):ti,ab,kw) OR ('squamous cell carcinoma'/de AND 'small intestine'/exp) OR ((('squamous cell carcinoma$' OR scc) NEAR/3 'small intestine$'):ti,ab,kw) OR 'colon adenocarcinoma'/exp OR ((adenocarcinoma$ NEAR/3 colon*):ti,ab,kw) OR ('signet ring carcinoma'/exp AND 'colon'/de) OR ('signet ring cell carcinoma$':ti,ab,kw AND colon*:ti,ab,kw) OR ('colloid carcinoma'/de AND 'colon'/de) OR (('mucinous adenocarcinoma$' NEAR/3 colon*):ti,ab,kw) OR ('adenosquamous carcinoma'/de AND 'colon'/de) OR (('adenosquamous carcinoma$' NEAR/3 colon*):ti,ab,kw) OR ('anaplastic carcinoma'/de AND 'colon'/de) OR (('undifferentiated carcinoma$' NEAR/3 colon*):ti,ab,kw) OR ('medullary carcinoma'/de AND 'colon'/de) OR (('medullary carcinoma$' NEAR/3 colon*):ti,ab,kw) OR 'squamous cell carcinoma of the colon'/exp OR ((('squamous cell carcinoma$' OR scc) NEAR/3 colon*):ti,ab,kw) OR ('colloid carcinoma'/de AND 'appendix'/exp) OR (('mucinous adenocarcinoma$' NEAR/3 append*):ti,ab,kw) OR ((fibromyxoma$ NEAR/3 append*):ti,ab,kw) OR 'pancreas adenocarcinoma'/exp OR ((adenocarcinoma$ NEAR/3 pancrea*):ti,ab,kw) OR (('mucinous adenocarcinoma$' NEAR/3 pancrea*):ti,ab,kw) OR ('anaplastic carcinoma'/de AND 'pancreas'/exp) OR (('undifferentiated carcinoma$' NEAR/3 pancrea*):ti,ab,kw) OR ('signet ring carcinoma'/exp AND 'pancreas'/exp) OR ('signet ring cell carcinoma$':ti,ab,kw AND pancrea*:ti,ab,kw) OR ('adenosquamous carcinoma'/de AND 'pancreas'/exp) OR (('adenosquamous carcinoma$' NEAR/3 pancrea*):ti,ab,kw) OR 'squamous cell carcinoma of the pancreas'/exp OR ((('squamous cell carcinoma$' OR scc) NEAR/3 pancrea*):ti,ab,kw) OR 'acinar cell carcinoma of the pancreas'/exp OR (('acinar cell carcinoma$' NEAR/3 pancrea*):ti,ab,kw) OR ('colloid carcinoma'/de AND 'pancreas'/exp) OR (('mucinous cystadenocarcinoma' NEAR/3 pancrea*):ti,ab,kw) OR (('intraductal papillary mucinous carcinoma$' NEAR/3 pancrea*):ti,ab,kw) OR (('solid pseudopapillary carcinoma$' NEAR/3 pancrea*):ti,ab,kw) OR 'cystadenocarcinoma of the pancreas'/exp OR (('serous cystadenocarcinoma$'	

	NEAR/3 pancrea*):ti,ab,kw) OR (('osteoclast-like giant cell' NEAR/3 carcinoma$ NEAR/3 pancrea*):ti,ab,kw) OR 'pancreatoblastoma'/exp OR pancreatoblastoma$:ti,ab,kw	
3	'biliary tract cancer'/exp OR ((biliary NEAR/3 (neoplas* OR cancer$ OR tumo$r$ OR cystadenocarcinoma$)):ti,ab,kw) OR (('bile duct' NEAR/3 (neoplas* OR cancer$ OR tumo$r$ OR carcinoma$ OR adenocarcinoma$)):ti,ab,kw) OR 'bile duct carcinoma'/exp OR cholangiocarcinoma$:ti,ab,kw OR 'klatskin tumo$r$':ti,ab,kw OR 'gallbladder cancer'/exp OR ((gallbladder NEAR/3 (neoplas* OR cancer$ OR carcinoma$ OR sarcoma$ OR adenocarcinoma$)):ti,ab,kw) OR 'vater papilla carcinoma'/exp OR 'ampullary cancer$':ti,ab,kw OR 'vater papilla carcinoma$':ti,ab,kw OR 'liver cell carcinoma'/exp OR 'hepatocellular carcinoma$':ti,ab,kw OR ('adenocarcinoma'/de AND 'liver'/exp) OR ((adenocarcinoma$ NEAR/3 (liver OR 'intrahepatic bil*')):ti,ab,kw) OR ('anaplastic carcinoma'/de AND 'liver'/exp) OR (('undifferentiated carcinoma$' NEAR/3 (liver OR 'intrahepatic bil*')):ti,ab,kw) OR ((('squamous cell carcinoma$' OR scc) NEAR/3 (liver OR 'intrahepatic bil*')):ti,ab,kw) OR 'bile duct cystadenocarcinoma$':ti,ab,kw OR 'hepatoblastoma'/de OR hepatoblastoma$:ti,ab,kw OR ('epithelium tumor'/exp AND ('gallbladder'/exp OR 'extrahepatic bile duct'/exp)) OR (('epithelial tumo$r$' NEAR/3 (gallbladder OR 'extrahepatic bil*')):ti,ab,kw) OR 'bile duct adenocarcinoma'/exp OR ((adenocarcinoma$ NEAR/3 'extrahepatic bil*'):ti,ab,kw) OR ('squamous cell carcinoma'/de AND 'extrahepatic bile duct'/exp) OR ((('squamous cell carcinoma$' OR scc) NEAR/3 'extrahepatic bil*'):ti,ab,kw) OR 'gallbladder adenocarcinoma'/de OR ((adenocarcinoma$ NEAR/3 gallbladder$):ti,ab,kw) OR ('colloid carcinoma'/de AND 'gallbladder'/exp) OR (('mucinous adenocarcinoma$' NEAR/3 gallbladder$):ti,ab,kw) OR (('clear cell adenocarcinoma$' NEAR/3 gallbladder$):ti,ab,kw) OR ('adenosquamous carcinoma'/de AND 'gallbladder'/exp) OR (('adenosquamous carcinoma$' NEAR/3 gallbladder$):ti,ab,kw) OR ('anaplastic carcinoma'/de AND 'gallbladder'/exp) OR (('undifferentiated carcinoma$' NEAR/3 gallbladder$):ti,ab,kw)	286218
4	'anus cancer'/exp OR (((anal OR anus) NEAR/3 (cancer$ OR neoplas* OR carcinoma$ OR adenocarcinoma$)):ti,ab,kw) OR ssca:ti,ab,kw OR ('epithelium tumor'/exp AND 'rectum'/exp) OR (('epithelial tumo$r$' NEAR/3 rect*):ti,ab,kw) OR ('rectal adenocarcinoma'/exp AND 'rectum'/exp) OR ((adenocarcinoma$ NEAR/3 rect*):ti,ab,kw) OR ('signet ring carcinoma'/exp AND 'rectum'/exp) OR ('signet ring cell carcinoma$':ti,ab,kw AND rect*:ti,ab,kw) OR ('colloid carcinoma'/de AND 'rectum'/exp) OR (('mucinous adenocarcinoma$' NEAR/3 rect*):ti,ab,kw) OR ('adenosquamous carcinoma'/de AND 'rectum'/exp) OR (('adenosquamous carcinoma$' NEAR/3 rect*):ti,ab,kw) OR ('anaplastic carcinoma'/de AND 'rectum'/exp) OR (('undifferentiated carcinoma$' NEAR/3 rect*):ti,ab,kw) OR ('medullary carcinoma'/de AND 'rectum'/exp) OR (('medullary carcinoma$' NEAR/3 rect*):ti,ab,kw) OR 'squamous cell carcinoma of the rectum'/exp OR ((('squamous cell carcinoma$' OR scc) NEAR/3 rect*):ti,ab,kw) OR ('verrucous carcinoma'/exp AND 'rectum'/exp) OR (('verrucous carcinoma$' NEAR/3 rect*):ti,ab,kw) OR ('basaloid carcinoma$':ti,ab,kw AND 'rectum'/exp) OR (('basaloid carcinoma$' NEAR/3 rect*):ti,ab,kw) OR ('epithelium tumor'/exp AND 'anal canal'/exp) OR (('epithelial tumo$r$' NEAR/3 'anal canal'):ti,ab,kw) OR ((('squamous cell carcinoma$' OR scc) NEAR/3 'anal canal'):ti,ab,kw) OR ('verrucous carcinoma'/exp AND 'anal canal'/exp) OR (('verrucous carcinoma$' NEAR/3 'anal canal'):ti,ab,kw) OR ('basaloid carcinoma$':ti,ab,kw AND 'anal canal'/exp) OR (('basaloid carcinoma$' NEAR/3 'anal canal'):ti,ab,kw) OR 'anal	20151

	adenocarcinoma'/exp OR ((adenocarcinoma$ NEAR/3 'anal canal'):ti,ab,kw) OR (('mucinous adenocarcinoma$' NEAR/3 'anal canal'):ti,ab,kw) OR ('adenosquamous carcinoma'/de AND 'anal canal'/exp) OR (('adenosquamous carcinoma$' NEAR/3 'anal canal'):ti,ab,kw) OR ('paget skin disease'/exp AND 'anal canal'/exp) OR (('paget disease' NEAR/3 'anal canal'):ti,ab,kw)	
5	'peritoneum pseudomyxoma'/exp OR 'pseudomyxoma peritonei':ti,ab,kw OR 'peritoneum pseudomyxoma':ti,ab,kw OR 'gelatinous ascites':ti,ab,kw	2950
6	'peritoneum mesothelioma'/exp OR 'peritoneal mespthelioma$':ti,ab,kw OR 'peritoneum mesothelioma$':ti,ab,kw OR 'primary peritoneal carcinoma'/exp OR 'primary peritoneal serous carcinoma$':ti,ab,kw OR ppsc:ti,ab,kw OR 'peritoneal desmoplastic small round cell tumo$r$':ti,ab,kw OR 'peritoneal desmoplastic small cell tumo$r$':ti,ab,kw OR ('peritoneum tumor'/exp AND 'leiomyomatosis'/de) OR 'diffuse peritoneal leiomyomatosis':ti,ab,kw OR 'leiomyomatosis peritonealis disseminata'/exp OR 'leiomyomatosis peritonealis disseminata':ti,ab,kw OR 'carcinomatous peritonitis'/exp OR 'carcinomatous peritonitis':ti,ab,kw	8792
7	#1 OR #2 OR #3 OR #4 OR #5 OR #6	439424
8	'quality of life'/exp/mj OR 'quality of life':ti,ab,kw OR hrqol:ti,ab,kw OR qol:ti,ab,kw	574615
9	#7 AND #8	6387
10	#9 NOT ('conference abstract'/it OR 'conference review'/it)	4306
11	#9 AND ('conference abstract'/it OR 'conference review'/it)	2081

Domain 6 – Endocrine

No.	Query	Results
1	(rare NEAR/3 endocrine NEAR/3 (cancer$ OR tumo$r$ OR malignan*)):ti,ab,kw	545
2	'thyroid cancer'/exp OR ((thyroid* NEAR/3 (cancer$ OR neoplas* OR adenoma$ OR lymphoma$ OR sarcoma$ OR carcinoma$)):ti,ab,kw) OR 'anaplastic thyroid carcinoma$':ti,ab,kw OR 'hurthle cell carcinoma'/exp OR 'hurthle cell carcinoma$':ti,ab,kw OR ('papillary adenocarcinoma$':ti,ab,kw AND 'thyroid gland'/exp) OR (('papillary adenocarcinoma$' NEAR/3 thyroid*):ti,ab,kw) OR 'anaplastic thyroid carcinoma'/exp OR (('undifferentiated carcinoma$' NEAR/3 thyroid*):ti,ab,kw) OR ('mucoepidermoid tumor'/exp AND 'thyroid gland'/exp) OR (('mucoepidermoid carcinoma$' NEAR/3 thyroid*):ti,ab,kw) OR ('colloid carcinoma'/exp AND 'thyroid gland'/exp) OR (('mucinous adenocarcinoma$' NEAR/3 thyroid*):ti,ab,kw) OR ('spindle cell carcinoma'/exp AND 'thyroid gland'/exp) OR ((carcinoma$ NEAR/3 'thymus like differentiation'):ti,ab,kw) OR 'parathyroid carcinoma'/exp OR ((carcinoma$ NEAR/3 parathyroid*):ti,ab,kw)	101630
3	('adrenal cortex carcinoma'/exp OR 'adrenocortical carcinoma$':ti,ab,kw OR 'adrenal cortex carcionoma$':ti,ab,kw OR 'adrenal cortical carcinoma$':ti,ab,kw OR 'suprarenal carcinoma$':ti,ab,kw OR 'pheochromocytoma'/exp) AND malignan*:ti,ab,kw OR ((malignant NEAR/3 (ph$eochromocytoma$ OR ph$eochromoblastoma OR 'chromaffin cell tumo$r$' OR 'chromaffin paraganglioma$')):ti,ab,kw) OR ('pheochromocytoma'/exp AND 'paraganglioma'/exp) OR ((phaeochromocytoma$ NEAR/3 paraganglioma$):ti,ab,kw) OR ppgl:ti,ab,kw OR ((carcinoma$ NEAR/3 'adrenal gland$'):ti,ab,kw) OR 'pituitary carcinoma'/exp OR ((carcinoma$ NEAR/3 ('pituitar*' OR hypophys$s)):ti,ab,kw)	10429
4	#1 OR #2 OR #3	111315

5	'quality of life'/exp/mj OR 'quality of life':ti,ab,kw OR hrqol:ti,ab,kw OR qol:ti,ab,kw	574615
6	#4 AND #5	1767
7	#6 NOT ('conference abstract'/it OR 'conference review'/it)	1331
8	#6 AND ('conference abstract'/it OR 'conference review'/it)	436

Domain 7 – Head and Neck

No.	Query	Results
1	(rare NEAR/3 (head OR neck) NEAR/3 (cancer$ OR tumo$r$ OR malignan*)):ti,ab,kw	576
2	'nose carcinoma'/exp OR ((sinonasal NEAR/3 (carcinoma$ OR scc OR melanoma$ OR sarcoma$ OR cancer$ OR tumo$r$ OR malignan* OR adenocarcinoma$)):ti,ab,kw) OR ((paranasal NEAR/3 (carcinoma$ OR scc OR melanoma$ OR sarcoma$ OR cancer$ OR tumo$r$ OR malignan* OR adenocarcinoma$)):ti,ab,kw) OR ((nasal NEAR/3 (carcinoma$ OR scc OR melanoma$ OR sarcoma$ OR cancer$ OR tumo$r$ OR malignan* OR adenocarcinoma$)):ti,ab,kw) OR ((sinus NEAR/3 (carcinoma$ OR scc OR melanoma$ OR sarcoma$ OR cancer$ OR tumo$r$ OR malignan* OR adenocarcinoma$)):ti,ab,kw) OR 'sinonasal inverted papilloma'/exp OR 'inverting papilloma$':ti,ab,kw OR 'midline granuloma$':ti,ab,kw OR 'olfactory neuroblastoma$':ti,ab,kw OR 'esthesioneuroblastoma$':ti,ab,kw OR 'nose cavity cancer$':ti,ab,kw OR ('epithelium tumor'/exp AND ('nose cavity'/de OR 'paranasal sinus'/exp)) OR (('epithelial tumo$r$' NEAR/3 ('nasal cavit*' OR 'nasal sinus*' OR 'paranasal sinus*')):ti,ab,kw) OR 'sinonasal squamous cell carcinoma'/exp OR ((('squamous cell carcinoma$' OR scc) NEAR/3 ('nasal cavit*' OR 'nasal sinus*' OR 'paranasal sinus*')):ti,ab,kw) OR (('squamous carcinoma$' NEAR/3 ('nasal cavit*' OR 'nasal sinus*' OR 'paranasal sinus*')):ti,ab,kw) OR ('verrucous carcinoma'/de AND ('nose cavity'/de OR 'paranasal sinus'/exp)) OR (('verrucous carcinoma$' NEAR/3 ('nasal cavit*' OR 'nasal sinus*' OR 'paranasal sinus*')):ti,ab,kw) OR ('basaloid carcinoma$':ti,ab,kw AND ('nose cavity'/de OR 'paranasal sinus'/exp)) OR (('basaloid carcinoma$' NEAR/3 ('nasal cavit*' OR 'nasal sinus*' OR 'paranasal sinus*')):ti,ab,kw) OR ('adenosquamous carcinoma'/de AND ('nose cavity'/de OR 'paranasal sinus'/exp)) OR (('adenosquamous carcinoma$' NEAR/3 ('nasal cavit*' OR 'nasal sinus*' OR 'paranasal sinus*')):ti,ab,kw) OR ('lymphoepithelioma'/exp AND ('nose cavity'/de OR 'paranasal sinus'/exp)) OR (('lymphoepithelial carcinoma$' NEAR/3 ('nasal cavit*' OR 'nasal sinus*' OR 'paranasal sinus*')):ti,ab,kw) OR 'sinonasal undifferentiated carcinoma'/exp OR (('undifferentiated carcinoma$' NEAR/3 ('nasal cavit*' OR 'nasal sinus*' OR 'paranasal sinus*')):ti,ab,kw)	15648
3	'salivary gland cancer'/exp OR (('salivary gland$' NEAR/3 (cancer$ OR tumo$r$ OR malignan* OR carcinoma$)):ti,ab,kw) OR 'salivary gland lymphadenoma$':ti,ab,kw OR 'non-sebaceous lymphadenoma$':ti,ab,kw OR 'sebaceous lymphadenoma$':ti,ab,kw OR ((parotid NEAR/3 (cancer$ OR tumo$r$ OR malignan* OR carcinoma$)):ti,ab,kw) OR (('sublingual gland$' NEAR/3 (cancer$ OR tumo$r$ OR malignan* OR carcinoma$)):ti,ab,kw) OR (('submandibular gland$' NEAR/3 (cancer$ OR tumo$r$ OR malignan* OR carcinoma$)):ti,ab,kw) OR 'warthin tumor'/de OR 'warthin tumo$r$':ti,ab,kw OR ('squamous cell carcinoma'/exp AND 'salivary gland'/exp) OR (('squamous carcinoma$' NEAR/3 'salivary gland$'):ti,ab,kw) OR ('lymphoepithelioma'/exp AND 'salivary gland'/exp) OR (('lymphoepithelial carcinoma$' NEAR/3 'salivary gland$'):ti,ab,kw) OR	24209

	('adenocarcinoma'/de AND 'salivary gland'/exp) OR ((adenocarcinoma$ NEAR/3 'salivary gland$'):ti,ab,kw) OR ('colloid carcinoma'/de AND 'salivary glands'/exp) OR (('mucinous adenocarcinoma$' NEAR/3 'salivary gland$'):ti,ab,kw) OR ('papillary cystadenocarcinoma$':ti,ab,kw AND 'salivary gland'/exp) OR (('papillary cystadenocarcinoma$' NEAR/3 'salivary gland$'):ti,ab,kw) OR 'salivary adenoid cystic carcinoma'/exp OR (('adenoid cystic carcinoma$' NEAR/3 'salivary gland$'):ti,ab,kw) OR ('mucoepidermoid tumor'/de AND 'salivary gland'/exp) OR (('mucoepidermoid carcinoma$' NEAR/3 'salivary gland$'):ti,ab,kw) OR ('acinar cell carcinoma'/de AND 'salivary gland'/exp) OR (('acinar cell carcinoma$' NEAR/3 'salivary gland$'):ti,ab,kw) OR ('myoepithelial carcinoma'/exp AND 'salivary gland'/exp) OR ((myoepithelioma$ NEAR/3 malignan* NEAR/3 'salivary gland$'):ti,ab,kw) OR ((carcinoma$ NEAR/3 'pleomorphic adenoma$'):ti,ab,kw) OR (('malignant mixed tumo$r$' NEAR/3 'salivary gland$'):ti,ab,kw) OR ('epithelial myoepithelial carcinoma'/de AND 'salivary gland'/exp) OR ((epithelial NEAR/1 myoepithelial NEAR/1 carcinoma$ NEAR/3 'salivary gland$'):ti,ab,kw) OR ('sebaceous carcinoma'/de AND 'salivary gland'/exp) OR (('sebaceous adenocarcinoma$' NEAR/3 'salivary gland$'):ti,ab,kw) OR ('cystadenocarcinoma'/de AND 'salivary gland'/exp) OR ((cystadenocarcinoma$ NEAR/3 'salivary gland$'):ti,ab,kw) OR ('ductal carcinoma'/de AND 'salivary gland'/exp) OR (('ductal carcinoma$' NEAR/3 'salivary gland$'):ti,ab,kw) OR ('oncocytic carcinoma'/exp AND 'salivary gland'/exp) OR (('oncocytic carcinoma$' NEAR/3 'salivary gland$'):ti,ab,kw)	
4	'nasopharynx carcinoma'/exp OR (((nasopharyn* OR rhinopharyn*) NEAR/3 (carcinoma$ OR adenocarcinoma$ OR cancer$)):ti,ab,kw) OR ((nasopharyn* NEAR/3 scq):ti,ab,kw) OR 'lymphoepithelioma$':ti,ab,kw OR ('epithelium tumor'/exp AND 'nasopharynx'/exp) OR (('epithelial tumo$r$' NEAR/3 nasopharyn*):ti,ab,kw) OR ('squamous cell carcinoma'/de AND 'nasopharynx'/exp) OR ((('squamous cell carcinoma$' OR scc) NEAR/3 nasopharyn*):ti,ab,kw) OR (('squamous carcinoma$' NEAR/3 nasopharyn*):ti,ab,kw) OR (('basaloid carcinoma$' NEAR/3 nasopharyn*):ti,ab,kw) OR ('lymphoepithelioma'/exp AND 'nasopharynx'/exp) OR (('lymphoepithelial carcinoma$' NEAR/3 nasopharyn*):ti,ab,kw) OR ('anaplastic carcinoma'/de AND 'nasopharynx'/exp) OR (('undifferentiated carcinoma$' NEAR/3 nasopharyn*):ti,ab,kw) OR (('papillary adenocarcinoma$' NEAR/3 nasopharyn*):ti,ab,kw)	29483
5	'middle ear carcinoma'/exp OR (('middle ear' NEAR/3 (cancer$ OR tumo$r$ OR malignan* OR schwannoma$ OR meningioma$ OR adenoma$ OR paraganglioma$ OR carcinoma$)):ti,ab,kw) OR 'hypopharynx squamous cell carcinoma'/exp OR ((('squamous cell carcinoma$' OR scc) NEAR/3 hypophary*):ti,ab,kw) OR (('squamous carcinoma$' NEAR/3 hypophary*):ti,ab,kw) OR ('lymphoepithelioma'/de AND 'hypopharynx'/de) OR (('lymphoepithelial carcinoma$' NEAR/3 hypophary*):ti,ab,kw) OR ('verrucous carcinoma'/de AND 'hypopharynx'/de) OR (('verrucous carcinoma$' NEAR/3 hypophary*):ti,ab,kw) OR ('giant cell carcinoma'/exp AND 'hypopharynx'/de) OR (('giant cell carcinoma$' NEAR/3 hypophary*):ti,ab,kw) OR ('adenosquamous carcinoma'/de AND 'hypopharynx'/de) OR (('adenosquamous carcinoma$' NEAR/3 hypophary*):ti,ab,kw) OR ('anaplastic carcinoma'/de AND 'hypopharynx'/de) OR (('undifferentiated carcinoma$' NEAR/3 hypophary*):ti,ab,kw) OR 'larynx squamous cell carcinoma'/exp OR ((('squamous cell carcinoma$' OR scc) NEAR/3 laryn*):ti,ab,kw) OR (('squamous carcinoma$' NEAR/3 laryn*):ti,ab,kw) OR ('verrucous carcinoma'/de AND 'larynx'/exp) OR (('verrucous carcinoma$' NEAR/3 laryn*):ti,ab,kw) OR ('adenosquamous carcinoma'/de AND 'larynx'/exp) OR	33520

	(('adenosquamous carcinoma$' NEAR/3 laryn*):ti,ab,kw) OR ('anaplastic carcinoma'/de AND 'larynx'/exp) OR (('undifferentiated carcinoma$' NEAR/3 laryn*):ti,ab,kw) OR ('lymphoepithelioma'/exp AND 'larynx'/exp) OR (('lymphoepithelial carcinoma$' NEAR/3 laryn*):ti,ab,kw) OR ('giant cell carcinoma'/exp AND 'larynx'/exp) OR (('giant cell carcinoma$' NEAR/3 laryn*):ti,ab,kw) OR ('epithelium tumor'/exp AND 'oropharynx'/exp) OR (('epithelial tumo$r$' NEAR/3 oropharyn*):ti,ab,kw) OR 'oropharynx squamous cell carcinoma'/exp OR ((('squamous cell carcinoma$' OR scc) NEAR/3 oropharyn*):ti,ab,kw) OR (('squamous carcinoma$' NEAR/3 oropharyn*):ti,ab,kw) OR ('lymphoepithelioma'/de AND 'oropharynx'/exp) OR (('lymphoepithelial carcinoma$' NEAR/3 oropharyn*):ti,ab,kw) OR ('verrucous carcinoma'/de AND 'oropharynx'/exp) OR (('verrucous carcinoma$' NEAR/3 oropharyn*):ti,ab,kw) OR ('adenosquamous carcinoma'/de AND 'oropharynx'/exp) OR (('adenosquamous carcinoma$' NEAR/3 oropharyn*):ti,ab,kw) OR ('anaplastic carcinoma'/de AND 'oropharynx'/exp) OR (('undifferentiated carcinoma$' NEAR/3 oropharyn*):ti,ab,kw) OR ('epithelium tumor'/exp AND 'mouth'/exp) OR (('epithelial tumo$r$' NEAR/3 ('oral cavit*' OR mouth$ OR 'lip*')):ti,ab,kw) OR 'mouth squamous cell carcinoma'/exp OR ((('squamous cell carcinoma$' OR scc) NEAR/3 ('oral cavit*' OR mouth$)):ti,ab,kw) OR (('squamous carcinoma$' NEAR/3 ('oral cavit*' OR mouth$)):ti,ab,kw) OR ('verrucous carcinoma'/de AND 'mouth'/de) OR (('verrucous carcinoma$' NEAR/3 ('oral cavit*' OR mouth$)):ti,ab,kw) OR ('adenosquamous carcinoma'/de AND 'mouth'/de) OR (('adenosquamous carcinoma$' NEAR/3 ('oral cavit*' OR mouth$)):ti,ab,kw) OR ('lymphoepithelioma'/de AND 'mouth'/de) OR ('anaplastic carcinoma'/de AND 'mouth'/de) OR (('undifferentiated carcinoma$' NEAR/3 ('oral cavit*' OR mouth$)):ti,ab,kw) OR ('squamous cell carcinoma'/de AND 'lip'/exp) OR ((('squamous cell carcinoma$' OR scc) NEAR/3 lip$):ti,ab,kw) OR ('epithelium tumor'/exp AND 'middle ear'/exp) OR (('epithelial tumo$r$' NEAR/3 'middle ear$'):ti,ab,kw) OR ('squamous cell carcinoma'/de AND 'middle ear'/exp) OR ((('squamous cell carcinoma$' OR scc) NEAR/3 'middle ear$'):ti,ab,kw) OR ('adenocarcinoma'/de AND 'middle ear'/exp) OR ((adenocarcinoma$ NEAR/3 'middle ear$'):ti,ab,kw) OR ('adenoid cystic carcinoma'/exp AND 'middle ear'/exp) OR (('adenoid cystic carcinoma$' NEAR/3 'middle ear$'):ti,ab,kw) OR 'odontogenic malignant tumo$r$':ti,ab,kw OR 'clear cell odontogenic carcinoma$':ti,ab,kw OR 'dentinogenic ghost cell tumor'/de OR 'ghost cell odontogenic carcinoma$':ti,ab,kw OR 'esthesioneuroblastoma'/de OR 'olfactory neuroblastoma$':ti,ab,kw	
6	#1 OR #2 OR #3 OR #4 OR #5	97730
7	'quality of life'/exp/mj OR 'quality of life':ti,ab,kw OR hrqol:ti,ab,kw OR qol:ti,ab,kw	57461 5
8	#6 AND #7	2081
9	#8 NOT ('conference abstract'/it OR 'conference review'/it)	1531
10	#8 AND ('conference abstract'/it OR 'conference review'/it)	550

Domain 8 - Thorax

No.	Query	Results
1	(rare NEAR/3 (thorax OR thoracic) NEAR/3 (cancer$ OR tumo$r$ OR malignan*)):ti,ab,kw	169
2	'thymic neoplasm'/exp OR (((thymic OR thymus) NEAR/3 (cancer$ OR tumo$r$ OR malignan* OR carcinoma$)):ti,ab,kw) OR thymoma$:ti,ab,kw OR ('epithelium	23558

	tumor'/exp AND 'thymus'/exp) OR (('epithelial tumo$r$' NEAR/3 thym*):ti,ab,kw) OR thymolipoma$:ti,ab,kw OR 'good syndrome':ti,ab,kw OR 'good s syndrome':ti,ab,kw OR 'thymic squamous cell carcinoma'/exp OR ((('squamous cell carcinoma$' OR scc) NEAR/3 thym*):ti,ab,kw) OR ('adenocarcinoma'/de AND 'thymus'/exp) OR ((adenocarcinoma$ NEAR/3 thym*):ti,ab,kw) OR ('anaplastic carcinoma'/de AND 'thymus'/exp) OR (('undifferentiated carcinoma$' NEAR/3 thym*):ti,ab,kw)	
3	'lung cancer'/exp AND 'mesothelioma'/exp OR 'malignant mesothelioma'/de OR 'pleura$ mesothelioma$':ti,ab,kw OR (('squamous cell carcinoma$' NEAR/3 'small cell' NEAR/3 (lung$ OR pleura$)):ti,ab,kw) OR ('clear cell carcinoma'/de AND 'lung'/exp) OR (('squamous cell carcinoma$' NEAR/3 'clear cell' NEAR/3 (lung$ OR pleura$)):ti,ab,kw) OR ('adenocarcinoma'/de AND 'lung'/exp) OR ((adenocarcinoma$ NEAR/3 (lung$ OR pleura$)):ti,ab,kw) OR ('colloid carcinoma'/de AND 'lung'/exp) OR (('mucinous adenocarcinoma$' NEAR/3 (lung$ OR pleura$)):ti,ab,kw) OR ('solid carcinoma$':ti,ab,kw AND 'lung'/exp) OR (('solid carcinoma$' NEAR/3 (lung$ OR pleura$)):ti,ab,kw) OR ('clear cell adenocarcinoma$':ti,ab,kw AND 'lung'/exp) OR (('clear cell adenocarcinoma$' NEAR/3 (lung$ OR pleura$)):ti,ab,kw) OR ('acinar cell carcinoma'/de AND 'lung'/exp) OR (('acinar cell carcinoma$' NEAR/3 (lung$ OR pleura$)):ti,ab,kw) OR ('signet ring carcinoma'/exp AND 'lung'/exp) OR (('signet ring cell carcinoma$' NEAR/3 (lung$ OR pleura$)):ti,ab,kw) OR ('mucinous cystadenocarcinoma':ti,ab,kw AND 'lung'/exp) OR (('mucinous cystadenocarcinoma' NEAR/3 (lung$ OR pleura$)):ti,ab,kw) OR 'lung alveolus cell carcinoma'/de OR 'bronchiolo-alveolar adenocarcinoma$':ti,ab,kw OR 'bronchioloalveolar adenocarcinoma$':ti,ab,kw OR ('adenosquamous carcinoma'/de AND 'lung'/exp) OR (('adenosquamous carcinoma$' NEAR/3 (lung$ OR pleura$)):ti,ab,kw) OR 'large cell lung carcinoma'/exp OR (('large cell carcinoma$' NEAR/3 (lung$ OR pleura$)):ti,ab,kw) OR (('poorly differentiated' NEAR/3 'endocrine carcinoma$' NEAR/3 (lung$ OR pleura$)):ti,ab,kw) OR ((('small cell' OR 'large cell') NEAR/3 'endocrine carcionoma$' NEAR/3 (lung$ OR pleura$)):ti,ab,kw) OR ('mucoepidermoid tumor'/exp AND 'lung'/exp) OR (('mucoepidermoid carcinoma$' NEAR/3 (lung$ OR pleura$)):ti,ab,kw) OR ('adenoid cystic carcinoma'/de AND 'lung'/exp) OR (('adenoid cystic carcinoma$' NEAR/3 (lung$ OR pleura$)):ti,ab,kw) OR ('epithelial myoepithelial carcinoma'/exp AND 'lung'/exp) OR ((epithelial NEAR/1 myoepithelial NEAR/1 carcinoma$ NEAR/3 (lung$ OR pleura$)):ti,ab,kw) OR ('sarcomatoid carcinoma'/exp AND 'lung'/exp) OR (('sarcomatoid carcinoma$' NEAR/3 (lung$ OR pleura$)):ti,ab,kw) OR ('pleomorphic carcinoma$':ti,ab,kw AND 'lung'/exp) OR (('pleomorphic carcinoma$' NEAR/3 (lung$ OR pleura$)):ti,ab,kw) OR ('giant cell carcinoma'/exp AND 'lung'/exp) OR (('giant cell carcinoma$' NEAR/3 (lung$ OR pleura$)):ti,ab,kw) OR 'lung blastoma'/exp OR 'pulmonary blastoma$':ti,ab,kw	69584
4	'epithelium tumor'/exp AND 'trachea'/exp OR (('epithelial tumo$r$' NEAR/3 trachea$):ti,ab,kw) OR ('squamous cell carcinoma'/de AND 'trachea'/exp) OR ((('squamous cell carcinoma$' OR scc) NEAR/3 trachea$):ti,ab,kw) OR (('squamous carcinoma$' NEAR/3 trachea$):ti,ab,kw) OR ('adenosquamous carcinoma'/de AND 'trachea'/exp) OR (('adenosquamous carcinoma$' NEAR/3 trachea$):ti,ab,kw) OR ('verrucous carcinoma'/exp AND 'trachea'/exp) OR (('verrucous carcinoma$' NEAR/3 trachea$):ti,ab,kw) OR ('anaplastic carcinoma'/exp AND 'trachea'/exp) OR (('undifferentiated carcinoma$' NEAR/3 trachea$):ti,ab,kw) OR ('spindle cell carcinoma'/exp AND 'trachea'/exp) OR (('spindle cell carcinoma$' NEAR/3 trachea$):ti,ab,kw) OR	1513

	('lymphoepithelioma'/exp AND 'trachea'/exp) OR (('lymphoepithelial carcinoma$' NEAR/3 trachea$):ti,ab,kw) OR ('giant cell carcinoma'/exp AND 'trachea'/exp) OR (('giant cell carcinoma$' NEAR/3 trachea$):ti,ab,kw) OR ('adenocarcinoma'/exp AND 'trachea'/exp) OR ((adenocarcinoma$ NEAR/3 trachea$):ti,ab,kw) OR ('colloid carcinoma'/de AND 'trachea'/exp) OR (('mucinous adenocarcinoma$' NEAR/3 trachea$):ti,ab,kw) OR ('papillary adenocarcinoma$':ti,ab,kw AND 'trachea'/exp) OR (('papillary adenocarcinoma$' NEAR/3 trachea$):ti,ab,kw) OR ('solid carcinoma$':ti,ab,kw AND 'trachea'/exp) OR (('solid carcinoma$' NEAR/3 trachea$):ti,ab,kw) OR ('clear cell adenocarcinoma$':ti,ab,kw AND 'trachea'/exp) OR (('clear cell adenocarcinoma$' NEAR/3 trachea$):ti,ab,kw) OR ('signet ring carcinoma'/exp AND 'trachea'/exp) OR ('signet ring cell carcinoma$':ti,ab,kw AND trachea$:ti,ab,kw) OR ('cystadenocarcinoma'/de AND 'trachea'/exp) OR (('mucinous cystadenocarcinoma' NEAR/3 trachea$):ti,ab,kw) OR ('adenoid cystic carcinoma'/exp AND 'trachea'/exp) OR (('adenoid cystic carcinoma$' NEAR/3 trachea$):ti,ab,kw) OR ('mucoepidermoid tumor'/exp AND 'trachea'/exp) OR (('mucoepidermoid carcinoma$' NEAR/3 trachea$):ti,ab,kw) OR ('myoepithelial carcinoma'/exp AND 'trachea'/exp) OR ((myoepithelial NEAR/1 carcinoma$ NEAR/3 trachea$):ti,ab,kw)	
5	'invasive lobular breast carcinoma'/exp OR ((classic NEAR/3 'invasive lobular carcinoma$' NEAR/3 (breast$ OR mamma*)):ti,ab,kw) OR ('pleomorphic carcinoma$':ti,ab,kw AND 'breast'/exp) OR (('pleomorphic carcinoma$' NEAR/3 (breast$ OR mamma*)):ti,ab,kw) OR 'paget nipple disease'/exp OR (('paget s disease' NEAR/3 (breast$ OR mamma*)):ti,ab,kw) OR 'tubular carcinoma of the breast'/exp OR (('tubular adenocarcinoma$' NEAR/3 (breast$ OR mamma*)):ti,ab,kw) OR 'mucinous carcinoma of the breast'/exp OR (('mucinous carcinoma$' NEAR/3 (breast$ OR mamma*)):ti,ab,kw) OR 'medullary carcinoma of the breast'/exp OR (('medullary carcinoma$' NEAR/3 (breast$ OR mamma*)):ti,ab,kw) OR ('papillary adenocarcinoma$':ti,ab,kw AND 'breast'/exp) OR (('papillary adenocarcinoma$' NEAR/3 (breast$ OR mamma*)):ti,ab,kw) OR ('cribriform carcinoma$':ti,ab,kw AND 'breast'/exp) OR (('cribriform carcinoma$' NEAR/3 (breast$ OR mamma*)):ti,ab,kw) OR ('apocrine adenocarcinoma$' AND 'breast'/exp) OR (('apocrine adenocarcinoma$' NEAR/3 (breast$ OR mamma*)):ti,ab,kw) OR 'secretory breast carcinoma'/exp OR (('secretory carcinoma$' NEAR/3 (breast$ OR mamma*)):ti,ab,kw) OR ('glycogen-rich carcinoma$':ti,ab,kw AND 'breast'/exp) OR (('glycogen-rich carcinoma$' NEAR/3 (breast$ OR mamma*)):ti,ab,kw) OR ('lipid-rich carcinoma$':ti,ab,kw AND 'breast'/exp) OR (('lipid-rich carcinoma$' NEAR/3 (breast$ OR mamma*)):ti,ab,kw) OR ('oncocytic carcinoma'/exp AND 'breast'/exp) OR (('oncocytic carcinoma$' NEAR/3 (breast$ OR mamma*)):ti,ab,kw) OR 'metaplastic carcinoma of the breast'/exp OR (('metaplastic carcinoma$' NEAR/3 (breast$ OR mamma*)):ti,ab,kw) OR ('squamous cell carcinoma'/de AND 'breast'/exp) OR (('squamous carcinoma$' NEAR/3 (breast$ OR mamma*)):ti,ab,kw) OR ('adenosquamous carcinoma'/de AND 'breast'/exp) OR (('adenosquamous carcinoma$' NEAR/3 (breast$ OR mamma*)):ti,ab,kw) OR ((adenocarcinoma$ NEAR/3 (cartil* OR osseous) NEAR/3 metaplas* NEAR/3 (breast$ OR mamma*)):ti,ab,kw) OR ('mucoepidermoid tumor'/de AND 'breast'/exp) OR (('mucoepidermoid carcinoma$' NEAR/3 (breast$ OR mamma*)):ti,ab,kw) OR 'adenoid cystic carcinoma of the breast'/exp OR (('adenoid cystic carcinoma$' NEAR/3 (breast$ OR mamma*)):ti,ab,kw) OR ('myoepithelial carcinoma'/exp AND 'breast'/exp) OR ((myoepithelial NEAR/1 carcinoma$ NEAR/3 (breast$ OR mamma*)):ti,ab,kw) OR ('acinar cell carcinoma'/de AND 'breast'/exp) OR (('acinar cell carcinoma$' NEAR/3 (breast$	33010

	OR mamma*)):ti,ab,kw) OR 'malignant mesothelioma'/exp OR ((malignan* NEAR/3 mesothelioma$):ti,ab,kw) OR ((mesothelioma$ NEAR/3 (pleura OR pericardium)):ti,ab,kw) OR ((mesothelioma$ NEAR/3 (peritoneum OR 'tunica vaginalis')):ti,ab,kw) OR 'pleuropulmonary blastoma'/exp OR ((pleuropulmonary NEAR/3 blastoma$):ti,ab,kw)	
6	#1 OR #2 OR #3 OR #4 OR #5	114886
7	'quality of life'/exp/mj OR 'quality of life':ti,ab,kw OR hrqol:ti,ab,kw OR qol:ti,ab,kw	574615
8	#6 AND #7	1454
9	#8 NOT ('conference abstract'/it OR 'conference review'/it)	854
10	#8 AND ('conference abstract'/it OR 'conference review'/it)	600

Domain 9 – Skin and eye melanoma

No.	Query	Results
1	(rare NEAR/3 (skin OR cutan*) NEAR/3 (cancer$ OR tumo$r$ OR malignan* OR carcinoma$)):ti,ab,kw	2304
2	'uvea melanoma'/exp OR ((uvea$ NEAR/3 melanoma$):ti,ab,kw) OR 'iridociliary melanoma$':ti,ab,kw OR 'irido-ciliary melanoma$':ti,ab,kw OR ((melanoma$ NEAR/3 eye$):ti,ab,kw) OR 'choroid melanoma$':ti,ab,kw OR 'choroidal melanoma$':ti,ab,kw OR ((melanoma$ NEAR/3 ciliary):ti,ab,kw) OR ((melanoma$ NEAR/3 iris):ti,ab,kw) OR ((melanoma$ NEAR/3 conjunctiva):ti,ab,kw)	13455
3	'merkel cell carcinoma'/exp OR (('merkel cell' NEAR/1 (carcinoma$ OR tumo$r$ OR cancer$)):ti,ab,kw) OR ('neuroendocrine carcinoma'/de AND 'skin'/exp) OR 'neuroendocrine carcinoma of the skin':ti,ab,kw OR 'cutaneous neuroendocrine carcinoma$':ti,ab,kw OR 'trabecular cancer$':ti,ab,kw OR 'cutaneous apudoma$':ti,ab,kw	7480
4	'mucosal melanoma'/exp OR 'mucosal melanoma$':ti,ab,kw OR ((mucous NEAR/3 melanoma$):ti,ab,kw) OR ((extracutaneous NEAR/3 melanoma$):ti,ab,kw) OR ('basal cell carcinoma'/exp AND 'skin'/exp) OR (('basal cell carcinoma$' NEAR/3 (skin OR cutan* OR dermal)):ti,ab,kw) OR 'basal and squamous cell carcinoma'/exp OR (('basosquamous carcinoma$' NEAR/3 (skin OR cutan* OR dermal)):ti,ab,kw) OR 'squamous cell skin carcinoma'/exp OR (('squamous carcinoma$' NEAR/3 (skin OR cutan* OR dermal)):ti,ab,kw) OR ('verrucous carcinoma'/exp AND 'skin'/exp) OR (('verrucous carcinoma$' NEAR/3 (skin OR cutan* OR dermal)):ti,ab,kw) OR ('spindle cell carcinoma$':ti,ab,kw AND 'skin'/exp) OR (('spindle cell carcinoma$' NEAR/3 (skin OR cutan* OR dermal)):ti,ab,kw) OR ((adenoid NEAR/3 'squamous cell carcinoma$' NEAR/3 (skin OR cutan* OR dermal)):ti,ab,kw) OR (((pseudovascular OR 'pseudo vascular') NEAR/3 'squamous cell carcinoma$' NEAR/3 (skin OR cutan* OR dermal)):ti,ab,kw) OR ('adenosquamous carcinoma'/de AND 'skin'/exp) OR (('adenosquamous carcinoma$' NEAR/3 (skin OR cutan* OR dermal)):ti,ab,kw) OR 'skin appendage carcinoma'/exp OR (('adnexal carcinoma$' NEAR/3 (skin OR cutan* OR dermal)):ti,ab,kw) OR (('nodular hidradenoma' NEAR/3 malignant NEAR/3 (skin OR cutan* OR dermal)):ti,ab,kw) OR 'sebaceous carcinoma'/exp OR 'sebaceous adenocarcinoma$':ti,ab,kw OR 'cutaneous adenoid cystic carcinoma'/exp OR (('adenoid cystic carcinoma$' NEAR/3 (skin OR cutan* OR dermal)):ti,ab,kw) OR 'paget skin disease'/exp OR (('paget disease' NEAR/3 (skin OR cutan* OR dermal)):ti,ab,kw) OR ('apocrine adenocarcinoma$':ti,ab,kw AND 'skin'/exp) OR (('apocrine adenocarcinoma$' NEAR/3 (skin OR cutan* OR dermal)):ti,ab,kw) OR ('colloid carcinoma'/de AND 'skin'/exp) OR (('mucinous	62238

	adenocarcinoma$' NEAR/3 (skin OR cutan* OR dermal)):ti,ab,kw) OR ('pillomatrix carcinoma$':ti,ab,kw AND 'skin'/exp) OR (('pillomatrix carcinoma$' NEAR/3 (skin OR cutan* OR dermal)):ti,ab,kw) OR 'eccrine porocarcinoma'/exp OR 'eccrine porocarcinoma':ti,ab,kw OR 'malignant mixed tumor of the skin'/exp OR (('mixed tumo$r$' NEAR/3 malignant NEAR/3 (skin OR cutan* OR dermal)):ti,ab,kw) OR ((sclerosing NEAR/3 'sweat duct$' NEAR/3 carcinoma$):ti,ab,kw) OR 'spiradenoma'/exp OR (('eccrine spiradenoma' NEAR/3 malignant):ti,ab,kw) OR (('tubular adenocarcinoma$' NEAR/3 (skin OR cutan* OR dermal)):ti,ab,kw) OR ((eccrine NEAR/3 papillary NEAR/3 adenocarcinoma$):ti,ab,kw) OR 'superficial spreading melanoma'/exp OR (('superficial spreading' NEAR/3 melanoma$):ti,ab,kw) OR 'nodular melanoma'/exp OR ((nodular NEAR/3 melanoma$):ti,ab,kw) OR 'malignant lentigo'/exp OR (('lentigo maligna' NEAR/3 melanoma$):ti,ab,kw) OR 'acral lentiginous melanoma'/exp OR (('acral lentiginous' NEAR/3 melanoma$ NEAR/3 malignan*):ti,ab,kw) OR ('epithelium tumor'/exp AND 'eye'/exp) OR (('epithelial tumo$r$' NEAR/3 (eye$ OR adnexa$)):ti,ab,kw) OR ('squamous cell carcinoma'/de AND 'eye'/exp) OR ((('squamous cell carcinoma$' OR scc) NEAR/3 (eye$ OR adnexa$)):ti,ab,kw) OR (('squamous carcinoma$' NEAR/3 (eye$ OR adnexa$)):ti,ab,kw) OR ('basal cell carcinoma'/exp AND 'eye'/exp) OR (('basal cell carcinoma$' NEAR/3 (eye$ OR adnexa$)):ti,ab,kw) OR ('adenocarcinoma'/exp AND 'eye'/exp) OR ((adenocarcinoma$ NEAR/3 (eye$ OR adnexa$)):ti,ab,kw) OR ('adenoid cystic carcinoma'/de AND 'eye'/exp) OR (('adenoid cystic carcinoma$' NEAR/3 (eye$ OR adnexa$)):ti,ab,kw) OR ('mucoepidermoid tumor'/exp AND 'eye'/exp) OR (('mucoepidermoid carcinoma$' NEAR/3 (eye$ OR adnexa$)):ti,ab,kw) OR ('embryonal tumor'/de AND 'eye'/exp) OR (('embryonal tumo$r$' NEAR/3 eye$):ti,ab,kw) OR 'retinoblastoma'/exp OR retinoblastoma$:ti,ab,kw	
5	#1 OR #2 OR #3 OR #4	82259
6	'quality of life'/exp/mj OR 'quality of life':ti,ab,kw OR hrqol:ti,ab,kw OR qol:ti,ab,kw	574615
7	#5 AND #6	805
8	#7 NOT ('conference abstract'/it OR 'conference review'/it)	539
9	#7 AND ('conference abstract'/it OR 'conference review'/it)	266

Domain 10 - Brain and spinal cords

No.	Query	Results
1	(rare NEAR/3 (brain OR 'spinal cord' OR 'nervous system') NEAR/3 (cancer$ OR tumo$r$ OR malignan* OR carcinoma$)):ti,ab,kw	1529
2	'glioma'/exp OR ((neuroglial NEAR/3 tumo$r$):ti,ab,kw) OR 'glioneural tumo$r$':ti,ab,kw OR 'neuronal-glial tumo$r$':ti,ab,kw OR glioma$:ti,ab,kw OR ((glial NEAR/3 tumo$r$):ti,ab,kw) OR astrocytoma$:ti,ab,kw OR astroglioma$:ti,ab,kw OR oligoastrocytoma$:ti,ab,kw OR glioblastoma$:ti,ab,kw OR 'pleomorphic xanthoastrocytoma$':ti,ab,kw OR ependymoma$:ti,ab,kw OR ependymoblastoma$:ti,ab,kw OR subependymoma$:ti,ab,kw OR 'ependymal tumo$r$':ti,ab,kw OR ganglioglioma$:ti,ab,kw OR gliosarcoma$:ti,ab,kw OR oligodendroglioma$:ti,ab,kw OR oligodendrocytoma$:ti,ab,kw OR ((astrocytic NEAR/3 tumo$r$):ti,ab,kw) OR astroblastoma$:ti,ab,kw OR 'gliomatosis cerebri':ti,ab,kw OR ((oligodendroglioma$ NEAR/3 tumo$r$):ti,ab,kw) OR 'choroid plexus carcinoma$':ti,ab,kw OR 'malignant meningioma'/exp OR (('meningioma$' NEAR/3 malignan*):ti,ab,kw) OR 'pineocytoma'/exp OR pinealoma$:ti,ab,kw OR ((tumo$r$ NEAR/3 pineal):ti,ab,kw) OR	206864

	pineoblastoma$:ti,ab,kw OR ('embryonal tumor'/de AND 'central nervous system'/exp) OR (('embryonal tumo$r$' NEAR/3 ('central nervous system' OR cns)):ti,ab,kw) OR 'atypical teratoid rhabdoid tumor'/exp OR 'atypical teratoid tumo$r$':ti,ab,kw OR 'neuroepithelioma'/exp OR neuroepithelioma$:ti,ab,kw	
3	'medulloblastoma'/de AND 'adult'/exp OR ((medulloblastoma$:ti,ab,kw OR 'medullo blastoma$':ti,ab,kw) AND (adult$:ti,ab,kw OR aged:ti,ab,kw OR elderly:ti,ab,kw OR postpubertal:ti,ab,kw OR 'post pubertal':ti,ab,kw))	5369
4	#1 OR #2 OR #3	210491
5	'quality of life'/exp/mj OR 'quality of life':ti,ab,kw OR hrqol:ti,ab,kw OR qol:ti,ab,kw	574615
6	#4 AND #5	4218
7	#6 NOT ('conference abstract'/it OR 'conference review'/it)	2585
8	#6 AND ('conference abstract'/it OR 'conference review'/it)	1633

Scopus.com

Domain 0 – General Terms

Query	Hits
(<br>TITLE-ABS(rare W/2 (cancer* OR tumor* OR tumour* OR malignan* OR neoplas*))<br>OR AUTHKEY(rare W/2 (cancer* OR tumor* OR tumour* OR malignan* OR neoplas*))<br>)<br>AND (<br>TITLE-ABS("Quality Of Life" OR HRQOL OR QOL)<br>OR AUTHKEY("Quality Of Life" OR HRQOL OR QOL)<br>)<br>AND (<br>EXCLUDE (DOCTYPE,"cr")<br>OR EXCLUDE (DOCTYPE,"cp")<br>)	890

Domain 1 - Sarcoma

Query	Hits
(<br>TITLE-ABS(sarcoma*)<br>OR AUTHKEY(sarcoma*)<br>OR TITLE-ABS(adenosarcoma* OR carcinosarcoma* OR "desmoplastic small round cell tumor*" OR "desmoplastic small round cell tumour*" OR "desmoplastic small cell tumor*" OR "desmoplastic small cell tumour*" OR DSRCT* OR "endometrial stromal tumor*" OR "endometrial stromal tumour*" OR "endolymphatic stromal myos*" OR fibrosarcoma* OR dermatofibrosarcoma* OR DFSP OR neurofibrosarcoma* OR hemangiosarcoma* OR angiosarcoma* OR "malignant fibrous histiocytoma*" OR "malignant fibrohistiocytic tumor*" OR "malignant fibrohistiocytic tumour*" OR leiomyosarcoma* OR liposarcoma* OR lymphangiosarcoma* OR "mesodermal mixed tumor*" OR "mesodermal mixed tumour*" OR myosarcoma* OR rhabdomyosarcoma* OR myxosarcoma* OR "phyllodes tumor*" OR "phyllodes tumour*" OR fibromyxosarcoma* OR gliosarcoma* OR leukosarcoma* OR "stewart-treves syndrome" OR lymphosarcoma* OR reticulosarcoma* OR "rhabdoid tumor*" OR "rhabdoid tumour*" OR "fibroblastic sarcoma*" OR "epithelioid hemangioendothelioma*" OR	1815

"malignant peripheral nerve sheath tumor*" OR "malignant peripheral nerve sheath tumour*" OR glomangiosarcoma* OR "malignant glomus tumor*" OR "malignant glomus tumour*" OR "perivascular epithelioid cell tumor*" OR "perivascular epithelioid cell tumour*" OR PEComa* OR "ewing sarcoma*" OR ESFT OR "peripheral neuroectodermal tumor*" OR "peripheral neuroectodermal tumour*" OR "primitive neuroectodermal tumor*" OR "primitive neuroectodermal tumour*" OR pPNET OR medulloepithelioma*)<br>OR AUTHKEY(adenosarcoma* OR carcinosarcoma* OR "desmoplastic small round cell tumor*" OR "desmoplastic small round cell tumour*" OR "desmoplastic small cell tumor*" OR "desmoplastic small cell tumour*" OR DSRCT* OR "endometrial stromal tumor*" OR "endometrial stromal tumour*" OR "endolymphatic stromal myos*" OR fibrosarcoma* OR dermatofibrosarcoma* OR DFSP OR neurofibrosarcoma* OR hemangiosarcoma* OR angiosarcoma* OR "malignant fibrous histiocytoma*" OR "malignant fibrohistiocytic tumor*" OR "malignant fibrohistiocytic tumour*" OR leiomyosarcoma* OR liposarcoma* OR lymphangiosarcoma* OR "mesodermal mixed tumor*" OR "mesodermal mixed tumour*" OR myosarcoma* OR rhabdomyosarcoma* OR myxosarcoma* OR "phyllodes tumor*" OR "phyllodes tumour*" OR fibromyxosarcoma* OR gliosarcoma* OR leukosarcoma* OR "stewart-treves syndrome" OR lymphosarcoma* OR reticulosarcoma* OR "rhabdoid tumor*" OR "rhabdoid tumour*" OR "fibroblastic sarcoma*" OR "epithelioid hemangioendothelioma*" OR "malignant peripheral nerve sheath tumor*" OR "malignant peripheral nerve sheath tumour*" OR glomangiosarcoma* OR "malignant glomus tumor*" OR "malignant glomus tumour*" OR "perivascular epithelioid cell tumor*" OR "perivascular epithelioid cell tumour*" OR PEComa* OR "ewing sarcoma*" OR ESFT OR "peripheral neuroectodermal tumor*" OR "peripheral neuroectodermal tumour*" OR "primitive neuroectodermal tumor*" OR "primitive neuroectodermal tumour*" OR pPNET OR medulloepithelioma*)<br>OR TITLE-ABS(((gastrointestinal OR gastric OR GI) W/1 stroma* W/1 (tumor* OR tumour* OR neoplas* OR sarcoma*)) OR GIST* OR "carney triad*" OR "Carney-Stratakis syndrome" OR "Carney-Stratakis dyad*")<br>OR AUTHKEY(((gastrointestinal OR gastric OR GI) W/1 stroma* W/1 (tumor* OR tumour* OR neoplas* OR sarcoma*)) OR GIST* OR "carney triad*" OR "Carney-Stratakis syndrome" OR "Carney-Stratakis dyad*")<br>OR TITLE-ABS(osteosarcoma* OR "osteogenic sarcoma*" OR "bone sarcoma*" OR chondrosarcoma* OR (("giant cell tumor*" OR "giant cell tumour*") W/3 bone*) OR GCTB OR osteoclastoma* OR chordoma* OR "notochordal sarcoma*")<br>OR AUTHKEY(osteosarcoma* OR "osteogenic sarcoma*" OR "bone sarcoma*" OR chondrosarcoma* OR (("giant cell tumor*" OR "giant cell tumour*") W/3 bone*) OR GCTB OR osteoclastoma* OR chordoma* OR "notochordal sarcoma*")<br>)<br>AND (<br>TITLE-ABS("Quality Of Life" OR HRQOL OR QOL)<br>OR AUTHKEY("Quality Of Life" OR HRQOL OR QOL)<br>)<br>AND (<br>EXCLUDE(DOCTYPE,"cr")<br>OR EXCLUDE(DOCTYPE,"cp")<br>)	

Domain 2 – Female Genital and Placenta

**Query**	**Hits**
(	386

TITLE-ABS(rare W/3 ("female genital" OR fallopian OR uterine OR vaginal OR vulvar) W/3 (cancer* OR tumor* OR tumour* OR malignan*))<br>OR AUTHKEY(rare W/3 ("female genital" OR fallopian OR uterine OR vaginal OR vulvar) W/3 (cancer* OR tumor* OR tumour* OR malignan*))<br>OR TITLE-ABS(("non epithelial" W/2 (ovarian OR ovary) W/2 (cancer* OR tumour* OR tumor* OR malignan*)) OR "sex cord stromal tumour*" OR "sex cord stromal tumor*" OR ("sex cord tumor*" W/3 ovar*) OR SCST OR SCCO OR SCCOHT OR dysgerminoma* OR "endodermal sinus tumour*" OR "endodermal sinus tumor*" OR "yolk sac tumor*" OR "yolk sac tumour*" OR orchioblastoma* OR ((embryo OR embryon*) W/1 (carcinoma* OR adenocarcinoma* OR "cell cancer*")) OR "non-gestational choriocarcinoma*" OR "nongestational choriocarcinoma*" OR "mature teratoma*" OR teratosarcoma* OR "immature teratoma*" OR "mixed germ cell tumor*" OR "mixed germ cell tumor*" OR fibroma* OR thecoma* OR hemangiofibroma* OR fibrosarcoma* OR "fibroblastic sarcoma" OR "sclerosing stromal tumor*" OR "sclerosing stromal tumour*" OR "signet ring stromal tumor*" OR "signet ring stromal tumour*" OR "microcystic stromal tumor*" OR "microcystic stromal tumour*" OR "leydig cell tumor*" OR "leydig cell tumour*" OR "steroid cell tumor*" OR "steroid cell tumour*" OR "granulosa cell tumor*" OR "granulosa cell tumour*" OR "granulosa cell carcinoma*" OR "granulosa cancer*" OR "sertoli cell tumor*" OR "sertoli cell tumour*" OR "sertoli-leydig cell tumor*" OR "sertoli-leydig cell tumour*" OR androblastoma* OR (("endometrioid adenocarcinoma*" OR "mucinous adenocarcinoma*" OR "mixed cell adenocarcinoma*") W/3 uter*) OR "villous adenocarcinoma*" OR "brenner tumor*" OR (("transitional cell carcinoma*" OR "basaloid carcinoma*" OR "squamous cell carcinoma*" OR SCC OR "adenoid cystic carcinoma*" OR "clear cell adenocarcinoma*") W/3 uter*) OR (serous W/3 carcinoma*) OR "mullerian mixed tumor*" OR "mullerian mixed tumour*" OR (("squamous cell carcinoma*" OR SCC OR "basaloid carcinoma*" OR "lymphoepithelial carcinoma*" OR "transitional cell carcinoma*" OR "glassy cell carcinoma*" OR adenocarcinoma* OR "serous cystadenocarcinoma*" OR "undifferentiated carcinoma*" OR "signet ring cell carcinoma*" OR mesonephroma*) W/3 cervi*) OR CSCC OR ((verrucous OR warty OR "spindle cell") W/1 carcinoma* W/3 cervi*) OR ((adenocarcinoma* OR "serous cystadenocarcinoma*" OR "transitional cell carcinoma*" OR "basaloid carcinoma*" OR "mucinous adenocarcinoma*" OR "clear cell adenocarcinoma*") W/3 ovar*) OR (endometrioid* W/1 adenofibroma* W/3 ovar*) OR adenocarcinofibroma* OR (peritoneal W/3 (serous OR papillary) W/3 carcinoma*) OR ((adenocarcinoma* OR "serous cystadenocarcinoma*") W/3 ("fallopian tube*" OR oviduct*)) OR "struma ovarii" OR ("germ cell tumor*" W/3 ovar*))<br>OR AUTHKEY(("non epithelial" W/2 (ovarian OR ovary) W/2 (cancer* OR tumour* OR tumor* OR malignan*)) OR "sex cord stromal tumour*" OR "sex cord stromal tumor*" OR ("sex cord tumor*" W/3 ovar*) OR SCST OR SCCO OR SCCOHT OR dysgerminoma* OR "endodermal sinus tumour*" OR "endodermal sinus tumor*" OR "yolk sac tumor*" OR "yolk sac tumour*" OR orchioblastoma* OR ((embryo OR embryon*) W/1 (carcinoma* OR adenocarcinoma* OR "cell cancer*")) OR "non-gestational choriocarcinoma*" OR "nongestational choriocarcinoma*" OR "mature teratoma*" OR teratosarcoma* OR "immature teratoma*" OR "mixed germ cell tumor*" OR "mixed germ cell tumor*" OR fibroma* OR thecoma* OR hemangiofibroma* OR fibrosarcoma* OR "fibroblastic sarcoma" OR "sclerosing stromal tumor*" OR "sclerosing stromal tumour*" OR "signet ring stromal tumor*" OR "signet ring stromal tumour*" OR "microcystic stromal tumor*" OR "microcystic stromal tumour*" OR "leydig cell tumor*" OR "leydig cell tumour*" OR "steroid cell tumor*" OR "steroid cell tumour*" OR "granulosa cell tumor*" OR "granulosa cell tumour*" OR "granulosa cell carcinoma*" OR "granulosa cancer*" OR "sertoli cell tumor*" OR "sertoli cell tumour*" OR "sertoli-leydig cell tumor*" OR "sertoli-leydig cell tumour*" OR androblastoma* OR (("endometrioid adenocarcinoma*" OR	

"mucinous adenocarcinoma*" OR "mixed cell adenocarcinoma*") W/3 uter*) OR "villous adenocarcinoma*" OR "brenner tumor*" OR (("transitional cell carcinoma*" OR "basaloid carcinoma*" OR "squamous cell carcinoma*" OR SCC OR "adenoid cystic carcinoma*" OR "clear cell adenocarcinoma*") W/3 uter*) OR (serous W/3 carcinoma*) OR "mullerian mixed tumor*" OR "mullerian mixed tumour*" OR (("squamous cell carcinoma*" OR SCC OR "basaloid carcinoma*" OR "lymphoepithelial carcinoma*" OR "transitional cell carcinoma*" OR "glassy cell carcinoma*" OR adenocarcinoma* OR "serous cystadenocarcinoma*" OR "undifferentiated carcinoma*" OR "signet ring cell carcinoma*" OR mesonephroma*) W/3 cervi*) OR CSCC OR ((verrucous OR warty OR "spindle cell") W/1 carcinoma* W/3 cervi*) OR ((adenocarcinoma* OR "serous cystadenocarcinoma*" OR "transitional cell carcinoma*" OR "basaloid carcinoma*" OR "mucinous adenocarcinoma*" OR "clear cell adenocarcinoma*") W/3 ovar*) OR (endometrioid* W/1 adenofibroma* W/3 ovar*) OR adenocarcinofibroma* OR (peritoneal W/3 (serous OR papillary) W/3 carcinoma*) OR ((adenocarcinoma* OR "serous cystadenocarcinoma*") W/3 ("fallopian tube*" OR oviduct*)) OR "struma ovarii" OR ("germ cell tumor*" W/3 ovar*))<br>OR TITLE-ABS("gestational trophoblastic disease*" OR GTD OR "gestational trophoblastic neoplas*" OR "invasive mole*" OR "hydatidiform mole*" OR "molar pregnanc*" OR "partial mole*" OR choriocarcinoma* OR "placental site trophoblastic tumor*" OR "placental site trophoblastic tumour*" OR PSTT OR "epithelioid trophoblastic tumor*" OR "epithelioid trophoblastic tumour*" OR (polyembryoma* W/3 ovar*) OR (("epithelial tumor*" OR "epithelial tumour*" OR "squamous cell carcinoma*" OR adenocarcinoma* OR "paget disease" OR "undifferentiated carcinoma*") W/3 (vulva OR vagina*)))<br>OR AUTHKEY("gestational trophoblastic disease*" OR GTD OR "gestational trophoblastic neoplas*" OR "invasive mole*" OR "hydatidiform mole*" OR "molar pregnanc*" OR "partial mole*" OR choriocarcinoma* OR "placental site trophoblastic tumor*" OR "placental site trophoblastic tumour*" OR PSTT OR "epithelioid trophoblastic tumor*" OR "epithelioid trophoblastic tumour*" OR (polyembryoma* W/3 ovar*) OR (("epithelial tumor*" OR "epithelial tumour*" OR "squamous cell carcinoma*" OR adenocarcinoma* OR "paget disease" OR "undifferentiated carcinoma*") W/3 (vulva OR vagina*)))<br>)<br>AND (<br>TITLE-ABS("Quality Of Life" OR HRQOL OR QOL)<br>OR AUTHKEY("Quality Of Life" OR HRQOL OR QOL)<br>)<br>AND (<br>EXCLUDE(DOCTYPE,"cr")<br>OR EXCLUDE(DOCTYPE,"cp")<br>)	

Domain 3 – Male genital organs and urinary tract

**Query**	**Hits**
(<br>TITLE-ABS((rare W/3 ("male genital" OR testicular OR "urinary tract" OR penile OR prostat*) W/3 (cancer* OR tumor* OR tumour* OR malignan*)) OR (("acinar cell carcinoma*" OR "mucinous adenocarcinoma*" OR "signet ring cell carcinoma*" OR "oxyphilic adenocarcinoma*" OR "lymphoepithelial carcinoma*" OR "squamous cell carcinoma*" OR "squamous carcinoma*" OR "adenosquamous carcinoma*" OR "basaloid carcinoma*" OR "infiltrating duct carcinoma*" OR "cribriform carcinoma*" OR "solid carcinoma*" OR "papillary adenocarcinoma*" OR "transitional cell carcinoma*" OR	307

"basal cell adenocarcinoma*" OR "adenoid cystic carcinoma*" OR "endometrioid adenocarcinoma*" OR "clear cell adenocarcinoma*" OR "serous cystadenocarcinoma*" OR "mucinous adenocarcinoma*" OR "collecting duct carcinoma*") W/3 prostate*) OR ((adenocarcinoma* W/3 "neuroendocrine differentiation") W/3 prostate*) OR ("spindle cell" W/3 carcinoma* W/3 prostate*) OR "paratesticular adenocarcinoma*" OR "rete testis")<br>OR AUTHKEY((rare W/3 ("male genital" OR testicular OR "urinary tract" OR penile OR prostat*) W/3 (cancer* OR tumor* OR tumour* OR malignan*)) OR (("acinar cell carcinoma*" OR "mucinous adenocarcinoma*" OR "signet ring cell carcinoma*" OR "oxyphilic adenocarcinoma*" OR "lymphoepithelial carcinoma*" OR "squamous cell carcinoma*" OR "squamous carcinoma*" OR "adenosquamous carcinoma*" OR "basaloid carcinoma*" OR "infiltrating duct carcinoma*" OR "cribriform carcinoma*" OR "solid carcinoma*" OR "papillary adenocarcinoma*" OR "transitional cell carcinoma*" OR "basal cell adenocarcinoma*" OR "adenoid cystic carcinoma*" OR "endometrioid adenocarcinoma*" OR "clear cell adenocarcinoma*" OR "serous cystadenocarcinoma*" OR "mucinous adenocarcinoma*" OR "collecting duct carcinoma*") W/3 prostate*) OR ((adenocarcinoma* W/3 "neuroendocrine differentiation") W/3 prostate*) OR ("spindle cell" W/3 carcinoma* W/3 prostate*) OR "paratesticular adenocarcinoma*" OR "rete testis")<br>OR TITLE-ABS(seminoma* OR "non-seminoma*" OR "testicular germ cell tumor*" OR "testicular germ cell tumour*" OR "mixed germ cell tumor*" OR "mixed germ cell tumour*" OR teratocarcinoma* OR "malignant teratoma*" OR (("sex cord cancer*" OR "gonadal stromal tumor*" OR "gonadal stromal tumour*") W/3 (testes OR testi*)) OR "Sertoli cell carcinoma*" OR (("epithelial tumor*" OR "epithelial tumour*" OR "squamous cell carcinoma*" OR "adenosquamous carcinoma*" OR "verrucous carcinoma*" OR "basaloid carcinoma*" OR adenocarcinoma* OR "paget disease" OR "mixed tumor*" OR "mixed tumour*") W/3 peni*) OR "renal cell adenocarcinoma*" OR (("clear cell adenocarcinoma*" OR "papillary adenocarcinoma*" OR "collecting duct carcinoma*" OR "medullary carcinoma*" OR "spindle cell carcinoma*" OR "squamous cell carcinoma*") W/3 (kidney OR renal)) OR ("renal cell carcinoma*" W/3 chromophobe*) OR (xp* W/3 "translocation carcinoma*") OR (carcinoma* W/3 neuroblastoma*) OR (tubular W/3 carcinoma* W/3 (kidney OR renal)) OR (("epithelial tumor*" OR "epithelial tumour*" OR carcinoma* OR "squamous cell carcinoma*" OR "basaloid carcinoma*" OR "verrucous carcinoma*" OR adenocarcinoma* OR "signet ring cell carcinoma*" OR "adenoid cystic carcinoma*") W/3 ("kindey pelvis" OR "renal pelvis" OR ureter*)) OR (("epithelial tumor*" OR "epithelial tumour*" OR carcinoma* OR "squamous cell carcinoma*" OR "basaloid carcinoma*" OR "verrucous carcinoma*" OR adenocarcinoma* OR "signet ring cell carcinoma*" OR "adenoid cystic carcinoma*") W/3 urethra*) OR (("undifferentiated carcinoma*" OR "transitional cell carcinoma*" OR "lymphoepithelial carcinoma*" OR "giant cell carcinoma*" OR "squamous cell carcinoma*" OR "basaloid carcinoma*" OR "verrucous carcinoma*" OR adenocarcinoma* OR "signet ring cell carcinoma*" OR "adenoid cystic carcinoma*" OR "mucoepidermoid carcinoma*") W/3 bladder) OR "extragonadal germ cell tumor*" OR "extragonadal germ cell tumour*" OR "embryonal adenocarcinoma*" OR (("germ cell tumor*" OR "germ cell tumour*") W/3 ("central nervous system" OR CNS)) OR nephroblastoma*)<br>OR AUTHKEY(seminoma* OR "non-seminoma*" OR "testicular germ cell tumor*" OR "testicular germ cell tumour*" OR "mixed germ cell tumor*" OR "mixed germ cell tumour*" OR teratocarcinoma* OR "malignant teratoma*" OR (("sex cord cancer*" OR "gonadal stromal tumor*" OR "gonadal stromal tumour*") W/3 (testes OR testi*)) OR "Sertoli cell carcinoma*" OR (("epithelial tumor*" OR "epithelial tumour*" OR "squamous cell carcinoma*" OR "adenosquamous carcinoma*" OR "verrucous carcinoma*" OR "basaloid carcinoma*" OR adenocarcinoma* OR "paget disease" OR "mixed tumor*" OR	

"mixed tumour*") W/3 peni*) OR "renal cell adenocarcinoma*" OR (("clear cell adenocarcinoma*" OR "papillary adenocarcinoma*" OR "collecting duct carcinoma*" OR "medullary carcinoma*" OR "spindle cell carcinoma*" OR "squamous cell carcinoma*") W/3 (kidney OR renal)) OR ("renal cell carcinoma*" W/3 chromophobe*) OR (xp* W/3 "translocation carcinoma*") OR (carcinoma* W/3 neuroblastoma*) OR (tubular W/3 carcinoma* W/3 (kidney OR renal)) OR (("epithelial tumor*" OR "epithelial tumour*" OR carcinoma* OR "squamous cell carcinoma*" OR "basaloid carcinoma*" OR "verrucous carcinoma*" OR adenocarcinoma* OR "signet ring cell carcinoma*" OR "adenoid cystic carcinoma*") W/3 ("kindey pelvis" OR "renal pelvis" OR ureter*)) OR (("epithelial tumor*" OR "epithelial tumour*" OR carcinoma* OR "squamous cell carcinoma*" OR "basaloid carcinoma*" OR "verrucous carcinoma*" OR adenocarcinoma* OR "signet ring cell carcinoma*" OR "adenoid cystic carcinoma*") W/3 urethra*) OR (("undifferentiated carcinoma*" OR "transitional cell carcinoma*" OR "lymphoepithelial carcinoma*" OR "giant cell carcinoma*" OR "squamous cell carcinoma*" OR "basaloid carcinoma*" OR "verrucous carcinoma*" OR adenocarcinoma* OR "signet ring cell carcinoma*" OR "adenoid cystic carcinoma*" OR "mucoepidermoid carcinoma*") W/3 bladder) OR "extragonadal germ cell tumor*" OR "extragonadal germ cell tumour*" OR "embryonal adenocarcinoma*" OR (("germ cell tumor*" OR "germ cell tumour*") W/3 ("central nervous system" OR CNS)) OR nephroblastoma*)<br>)<br>AND (<br>TITLE-ABS("Quality Of Life" OR HRQOL OR QOL)<br>OR AUTHKEY("Quality Of Life" OR HRQOL OR QOL)<br>)<br>AND (<br>EXCLUDE(DOCTYPE,"cr")<br>OR EXCLUDE(DOCTYPE,"cp")<br>)	

Domain 4 – Neuroendocrine system

Query	Hits
(<br>TITLE-ABS(rare W/3 (neuroendocrine OR lung OR thymic OR thymus) W/3 (cancer* OR tumor* OR tumour* OR malignan*))<br>OR AUTHKEY(rare W/3 (neuroendocrine OR lung OR thymic OR thymus) W/3 (cancer* OR tumor* OR tumour* OR malignan*))<br>OR TITLE-ABS((lung* W/3 carcinoid*) OR "pulmonary carcinoid*" OR "pulmonary neuroendocrine tumor*" OR "pulmonary neuroendocrine tumour*" OR "thymic carcinoid*" OR (thymus W/3 carcinoid*) OR (("neuroendocrine tumor*" OR "neuroendocrine tumour*" OR NET) W/3 lung*) OR (("neuroendocrine tumor*" OR "neuroendocrine tumour*" OR "NET") W/3 (gastroenteropancreatic OR "gastro entero pancreatic" OR "GEP")) OR (("endocrine carcinoma*" OR carcinoid* OR "islet cell carcinoma*") W/3 (pancrea* OR digestive OR gastrointestinal)) OR (insulinoma* W/3 malignan*) OR glucagonoma* OR (somatostatinoma* W/3 malignan*) OR gastrinoma* OR (vipoma* OR "vip oma*" OR "vasoactive intestinal peptide oma*") OR ("mixed pancreatic" W/5 (tumor* OR tumour*)) OR paraganglioma* OR (("medullary carcinoma*" OR "follicular carcinoma*" OR "endocrine tumor*" OR "endocrine tumour*") W/3 thyroid*) OR "neuroendocrine carcinoma*" OR ("well differentiated" W/3 ("endocrine tumor*" OR "endocrine tumour*") W/3 carcinoid*) OR ("poorly differentiated" W/3 "endocrine carcinoma*") OR neuroblastoma* OR ganglioneuroblastoma*)	519

OR AUTHKEY((lung* W/3 carcinoid*) OR "pulmonary carcinoid*" OR "pulmonary neuroendocrine tumor*" OR "pulmonary neuroendocrine tumour*" OR "thymic carcinoid*" OR (thymus W/3 carcinoid*) OR (("neuroendocrine tumor*" OR "neuroendocrine tumour*" OR NET) W/3 lung*) OR (("neuroendocrine tumor*" OR "neuroendocrine tumour*" OR "NET") W/3 (gastroenteropancreatic OR "gastro entero pancreatic" OR "GEP")) OR (("endocrine carcinoma*" OR carcinoid* OR "islet cell carcinoma*") W/3 (pancrea* OR digestive OR gastrointestinal)) OR (insulinoma* W/3 malignan*) OR glucagonoma* OR (somatostatinoma* W/3 malignan*) OR gastrinoma* OR (vipoma* OR "vip oma*" OR "vasoactive intestinal peptide oma*") OR ("mixed pancreatic" W/5 (tumor* OR tumour*)) OR paraganglioma* OR (("medullary carcinoma*" OR "follicular carcinoma*" OR "endocrine tumor*" OR "endocrine tumour*") W/3 thyroid*) OR "neuroendocrine carcinoma*" OR ("well differentiated" W/3 ("endocrine tumor*" OR "endocrine tumour*") W/3 carcinoid*) OR ("poorly differentiated" W/3 "endocrine carcinoma*") OR neuroblastoma* OR ganglioneuroblastoma*)<br>)<br>AND (<br>TITLE-ABS("Quality Of Life" OR HRQOL OR QOL)<br>OR AUTHKEY("Quality Of Life" OR HRQOL OR QOL)<br>)<br>AND (<br>EXCLUDE(DOCTYPE,"cr")<br>OR EXCLUDE(DOCTYPE,"cp")<br>)	

Domain 5 – Digestive tract

**Query**	**Hits**
(<br>TITLE-ABS((rare W/3 "digestive tract" W/3 (cancer* OR tumor* OR tumour* OR malignan*)) OR (("squamous cell carcinoma*" OR SCC OR "squamous carcinoma*" OR "adenosquamous carcinoma*" OR "verrucous carcinoma*" OR adenocarcinoma* OR "mucoepidermoid carcinoma*" OR "adenoid cystic carcinoma*" OR "undifferentiated carcinoma*") W/3 (esophagus OR oesophagus)) OR ((adenocarcinoma* OR "signet ring cell carcinoma*" OR "mucinous adenocarcinoma*" OR "adenosquamous carcinoma*" OR "squamous cell carcinoma*" OR SCC OR "squamous carcinoma*" OR "basaloid carcinoma*" OR "verrucous carcinoma*" OR "mucoepidermoid carcinoma*" OR "adenoid cystic carcinoma*" OR "undifferentiated carcinoma*") W/3 (stomach OR gastric)) OR (adenocarcinoma* W/3 "intestinal type") OR (("epithelial tumor*" OR "epithelial tumour*" OR adenocarcinoma* OR "mucinous adenocarcinoma*" OR "signet ring cell carcinoma*" OR "adenosquamous carcinoma*" OR "medullary carcinoma*" OR "undifferentiated carcinoma*" OR "squamous cell carcinoma*" OR SCC) W/3 "small intestine*") OR ((adenocarcinoma* OR "signet ring cell carcinoma*" OR "mucinous adenocarcinoma*" OR "adenosquamous carcinoma*" OR "undifferentiated carcinoma*" OR "medullary carcinoma*" OR "squamous cell carcinoma*" OR SCC) W/3 colon*) OR (("mucinous adenocarcinoma*" OR fibromyxoma*) W/3 append*) OR ((adenocarcinoma* OR "mucinous adenocarcinoma*" OR "undifferentiated carcinoma*" OR "signet ring cell carcinoma*" OR "adenosquamous carcinoma*" OR "squamous cell carcinoma*" OR SCC OR "acinar cell carcinoma*" OR "mucinous cystadenocarcinoma" OR "intraductal papillary mucinous carcinoma*" OR "solid pseudopapillary carcinoma*" OR "serous cystadenocarcinoma*") W/3 pancrea*) OR ("osteoclast-like giant cell" W/3 carcinoma* W/3 pancrea*) OR pancreatoblastoma*)	3129

OR AUTHKEY((rare W/3 "digestive tract" W/3 (cancer* OR tumor* OR tumour* OR malignan*)) OR (("squamous cell carcinoma*" OR SCC OR "squamous carcinoma*" OR "adenosquamous carcinoma*" OR "verrucous carcinoma*" OR adenocarcinoma* OR "mucoepidermoid carcinoma*" OR "adenoid cystic carcinoma*" OR "undifferentiated carcinoma*") W/3 (esophagus OR oesophagus)) OR ((adenocarcinoma* OR "signet ring cell carcinoma*" OR "mucinous adenocarcinoma*" OR "adenosquamous carcinoma*" OR "squamous cell carcinoma*" OR SCC OR "squamous carcinoma*" OR "basaloid carcinoma*" OR "verrucous carcinoma*" OR "mucoepidermoid carcinoma*" OR "adenoid cystic carcinoma*" OR "undifferentiated carcinoma*") W/3 (stomach OR gastric)) OR (adenocarcinoma* W/3 "intestinal type") OR (("epithelial tumor*" OR "epithelial tumour*" OR adenocarcinoma* OR "mucinous adenocarcinoma*" OR "signet ring cell carcinoma*" OR "adenosquamous carcinoma*" OR "medullary carcinoma*" OR "undifferentiated carcinoma*" OR "squamous cell carcinoma*" OR SCC) W/3 "small intestine*") OR ((adenocarcinoma* OR "signet ring cell carcinoma*" OR "mucinous adenocarcinoma*" OR "adenosquamous carcinoma*" OR "undifferentiated carcinoma*" OR "medullary carcinoma*" OR "squamous cell carcinoma*" OR SCC) W/3 colon*) OR (("mucinous adenocarcinoma*" OR fibromyxoma*) W/3 append*) OR ((adenocarcinoma* OR "mucinous adenocarcinoma*" OR "undifferentiated carcinoma*" OR "signet ring cell carcinoma*" OR "adenosquamous carcinoma*" OR "squamous cell carcinoma*" OR SCC OR "acinar cell carcinoma*" OR "mucinous cystadenocarcinoma" OR "intraductal papillary mucinous carcinoma*" OR "solid pseudopapillary carcinoma*" OR "serous cystadenocarcinoma*") W/3 pancrea*) OR ("osteoclast-like giant cell" W/3 carcinoma* W/3 pancrea*) OR pancreatoblastoma*)<br>OR TITLE-ABS((biliary W/3 (neoplas* OR cancer* OR tumor* OR tumour* OR cystadenocarcinoma*)) OR ("bile duct" W/3 (neoplas* OR cancer* OR tumor* OR tumour* OR carcinoma* OR adenocarcinoma*)) OR cholangiocarcinoma* OR "klatskin tumor*" OR "klatskin tumour*" OR (gallbladder W/3 (neoplas* OR cancer* OR carcinoma* OR sarcoma* OR adenocarcinoma*)) OR "gallbladder" OR "ampullary cancer*" OR "vater papilla carcinoma*" OR "hepatocellular carcinoma*" OR ((adenocarcinoma* OR "undifferentiated carcinoma*" OR "squamous cell carcinoma*" OR SCC) W/3 (liver OR "intrahepatic bil*")) OR "bile duct cystadenocarcinoma*" OR hepatoblastoma* OR (("epithelial tumor*" OR "epithelial tumour*") W/3 (gallbladder OR "extrahepatic bil*")) OR ((adenocarcinoma* OR "squamous cell carcinoma*" OR SCC) W/3 "extrahepatic bil*") OR ((adenocarcinoma* OR "mucinous adenocarcinoma*" OR "clear cell adenocarcinoma*" OR "adenosquamous carcinoma*" OR "undifferentiated carcinoma*") W/3 gallbladder*))<br>OR AUTHKEY((biliary W/3 (neoplas* OR cancer* OR tumor* OR tumour* OR cystadenocarcinoma*)) OR ("bile duct" W/3 (neoplas* OR cancer* OR tumor* OR tumour* OR carcinoma* OR adenocarcinoma*)) OR cholangiocarcinoma* OR "klatskin tumor*" OR "klatskin tumour*" OR (gallbladder W/3 (neoplas* OR cancer* OR carcinoma* OR sarcoma* OR adenocarcinoma*)) OR "gallbladder" OR "ampullary cancer*" OR "vater papilla carcinoma*" OR "hepatocellular carcinoma*" OR ((adenocarcinoma* OR "undifferentiated carcinoma*" OR "squamous cell carcinoma*" OR SCC) W/3 (liver OR "intrahepatic bil*")) OR "bile duct cystadenocarcinoma*" OR hepatoblastoma* OR (("epithelial tumor*" OR "epithelial tumour*") W/3 (gallbladder OR "extrahepatic bil*")) OR ((adenocarcinoma* OR "squamous cell carcinoma*" OR SCC) W/3 "extrahepatic bil*") OR ((adenocarcinoma* OR "mucinous adenocarcinoma*" OR "clear cell adenocarcinoma*" OR "adenosquamous carcinoma*" OR "undifferentiated carcinoma*") W/3 gallbladder*))<br>OR TITLE-ABS(((anal OR anus) W/3 (cancer* OR neoplas* OR carcinoma* OR adenocarcinoma*)) OR SSCA OR (("epithelial tumor*" OR "epithelial tumour*" OR adenocarcinoma* OR "signet ring cell carcinoma*" OR "mucinous adenocarcinoma*" OR	

"adenosquamous carcinoma*" OR "undifferentiated carcinoma*" OR "medullary carcinoma*" OR "squamous cell carcinoma*" OR SCC OR "verrucous carcinoma*" OR "basaloid carcinoma*" OR "epithelial tumor*" OR "epithelial tumour*") W/3 rect*) OR (("squamous cell carcinoma*" OR SCC OR "verrucous carcinoma*" OR "basaloid carcinoma*" OR adenocarcinoma* OR "mucinous adenocarcinoma*" OR "adenosquamous carcinoma*" OR "paget disease") W/3 "anal canal"))<br>OR AUTHKEY(((anal OR anus) W/3 (cancer* OR neoplas* OR carcinoma* OR adenocarcinoma*)) OR SSCA OR (("epithelial tumor*" OR "epithelial tumour*" OR adenocarcinoma* OR "signet ring cell carcinoma*" OR "mucinous adenocarcinoma*" OR "adenosquamous carcinoma*" OR "undifferentiated carcinoma*" OR "medullary carcinoma*" OR "squamous cell carcinoma*" OR SCC OR "verrucous carcinoma*" OR "basaloid carcinoma*" OR "epithelial tumor*" OR "epithelial tumour*") W/3 rect*) OR (("squamous cell carcinoma*" OR SCC OR "verrucous carcinoma*" OR "basaloid carcinoma*" OR adenocarcinoma* OR "mucinous adenocarcinoma*" OR "adenosquamous carcinoma*" OR "paget disease") W/3 "anal canal"))<br>OR TITLE-ABS("pseudomyxoma peritonei" OR "peritoneum pseudomyxoma" OR "gelatinous ascites")<br>OR AUTHKEY("pseudomyxoma peritonei" OR "peritoneum pseudomyxoma" OR "gelatinous ascites")<br>OR TITLE-ABS("peritoneal mesothelioma*" OR "peritoneum mesothelioma*" OR "primary peritoneal serous carcinoma*" OR PPSC OR "peritoneal desmoplastic small round cell tumor*" OR "peritoneal desmoplastic small round cell tumour*" OR "peritoneal desmoplastic small cell tumor*" OR "peritoneal desmoplastic small cell tumour*" OR "diffuse peritoneal leiomyomatosis" OR "leiomyomatosis peritonealis disseminata" OR "carcinomatous peritonitis")<br>OR AUTHKEY("peritoneal mesothelioma*" OR "peritoneum mesothelioma*" OR "primary peritoneal serous carcinoma*" OR PPSC OR "peritoneal desmoplastic small round cell tumor*" OR "peritoneal desmoplastic small round cell tumour*" OR "peritoneal desmoplastic small cell tumor*" OR "peritoneal desmoplastic small cell tumour*" OR "diffuse peritoneal leiomyomatosis" OR "leiomyomatosis peritonealis disseminata" OR "carcinomatous peritonitis")<br>)<br>AND (<br>TITLE-ABS("Quality Of Life" OR HRQOL OR QOL)<br>OR AUTHKEY("Quality Of Life" OR HRQOL OR QOL)<br>)<br>AND (<br>EXCLUDE(DOCTYPE,"cr")<br>OR EXCLUDE(DOCTYPE,"cp")<br>)	

Domain 6 - Endocrine Organs

**Query**	**Hits**
(<br>TITLE-ABS(rare W/3 endocrine W/3 (cancer* OR tumor* OR tumour* OR malignan*))<br>OR AUTHKEY(rare W/3 endocrine W/3 (cancer* OR tumor* OR tumour* OR malignan*))<br>OR TITLE-ABS((thyroid* W/3 (cancer* OR neoplas* OR adenoma* OR lymphoma* OR sarcoma* OR carcinoma*)) OR "anaplastic thyroid carcinoma*" OR "hurthle cell carcinoma*" OR (("papillary adenocarcinoma*" OR "undifferentiated carcinoma*" OR "mucoepidermoid carcinoma*" OR "mucinous adenocarcinoma*") W/3 thyroid*) OR	1049

"spindle cell tumor with thymus like differentiation*" OR "spindle cell tumour with thymus like differentiation*" OR (carcinoma* W/3 "thymus like differentiation") OR (carcinoma* W/3 parathyroid*))<br>OR AUTHKEY((thyroid* W/3 (cancer* OR neoplas* OR adenoma* OR lymphoma* OR sarcoma* OR carcinoma*)) OR "anaplastic thyroid carcinoma*" OR "hurthle cell carcinoma*" OR (("papillary adenocarcinoma*" OR "undifferentiated carcinoma*" OR "mucoepidermoid carcinoma*" OR "mucinous adenocarcinoma*") W/3 thyroid*) OR "spindle cell tumor with thymus like differentiation*" OR "spindle cell tumour with thymus like differentiation*" OR (carcinoma* W/3 "thymus like differentiation") OR (carcinoma* W/3 parathyroid*))<br>OR TITLE-ABS("adrenocortical carcinoma*" OR "adrenal cortex carcionoma*" OR "adrenal cortical carcinoma*" OR "suprarenal carcinoma*" OR (malignant W/3 (pheochromocytoma* OR phaeochromocytoma* OR phaeochromoblastoma OR "chromaffin cell tumor*" OR "chromaffin cell tumour*" OR "chromaffin paraganglioma*")) OR ((pheochromocytoma* OR phaeochromocytoma*) W/3 paraganglioma*) OR PPGL OR (carcinoma* W/3 ("adrenal gland*" OR "pituitar*" OR hypophys*)))<br>OR AUTHKEY("adrenocortical carcinoma*" OR "adrenal cortex carcionoma*" OR "adrenal cortical carcinoma*" OR "suprarenal carcinoma*" OR (malignant W/3 (pheochromocytoma* OR phaeochromocytoma* OR phaeochromoblastoma OR "chromaffin cell tumor*" OR "chromaffin cell tumour*" OR "chromaffin paraganglioma*")) OR ((pheochromocytoma* OR phaeochromocytoma*) W/3 paraganglioma*) OR PPGL OR (carcinoma* W/3 ("adrenal gland*" OR "pituitar*" OR hypophys*)))<br>)<br>AND (<br>TITLE-ABS("Quality Of Life" OR HRQOL OR QOL)<br>OR AUTHKEY("Quality Of Life" OR HRQOL OR QOL)<br>)<br>AND (<br>EXCLUDE(DOCTYPE,"cr")<br>OR EXCLUDE(DOCTYPE,"cp")<br>)	

Domain 7 - Head and Neck

Query	Hits
(<br>TITLE-ABS(rare W/3 (head OR neck) W/3 (cancer* OR tumor* OR tumour* OR malignan*))<br>OR AUTHKEY(rare W/3 (head OR neck) W/3 (cancer* OR tumor* OR tumour* OR malignan*))<br>OR TITLE-ABS(((sinonasal OR paranasal OR nasal OR sinus) W/3 (carcinoma* OR SCC OR melanoma* OR sarcoma* OR cancer* OR tumor* OR tumour* OR malignan* OR adenocarcinoma*)) OR "inverting papilloma*" OR "midline granuloma*" OR "olfactory neuroblastoma*" OR "esthesioneuroblastoma*" OR "nose cavity cancer*" OR (("epithelial tumor*" OR "epithelial tumour*" OR "squamous cell carcinoma*" OR SCC OR "squamous carcinoma*" OR "verrucous carcinoma*" OR "basaloid carcinoma*" OR "adenosquamous carcinoma*" OR "lymphoepithelial carcinoma*" OR "undifferentiated carcinoma*") W/3 ("nasal cavit*" OR "nasal sinus*" OR "paranasal sinus*")))	1194

OR AUTHKEY(((sinonasal OR paranasal OR nasal OR sinus) W/3 (carcinoma* OR SCC OR melanoma* OR sarcoma* OR cancer* OR tumor* OR tumour* OR malignan* OR adenocarcinoma*)) OR "inverting papilloma*" OR "midline granuloma*" OR "olfactory neuroblastoma*" OR "esthesioneuroblastoma*" OR "nose cavity cancer*" OR (("epithelial tumor*" OR "epithelial tumour*" OR "squamous cell carcinoma*" OR SCC OR "squamous carcinoma*" OR "verrucous carcinoma*" OR "basaloid carcinoma*" OR "adenosquamous carcinoma*" OR "lymphoepithelial carcinoma*" OR "undifferentiated carcinoma*") W/3 ("nasal cavit*" OR "nasal sinus*" OR "paranasal sinus*")))<br>OR TITLE-ABS((("salivary gland*" OR parotid OR "sublingual gland*" OR "submandibular gland*") W/3 (cancer* OR tumor* OR tumour* OR malignan* OR carcinoma*)) OR "salivary gland lymphadenoma*" OR "non-sebaceous lymphadenoma*" OR "sebaceous lymphadenoma*" OR "warthin tumor*" OR "warthin tumour*" OR (("squamous carcinoma*" OR "lymphoepithelial carcinoma*" OR adenocarcinoma* OR "mucinous adenocarcinoma*" OR "papillary cystadenocarcinoma*" OR "adenoid cystic carcinoma*" OR "mucoepidermoid carcinoma*" OR "acinar cell carcinoma*" OR "malignant mixed tumor*" OR "malignant mixed tumour*" OR "sebaceous adenocarcinoma*" OR cystadenocarcinoma* OR "ductal carcinoma*" OR "oncocytic carcinoma*") W/3 "salivary gland*") OR (myoepithelioma* W/3 malignan* W/3 "salivary gland*") OR (carcinoma* W/3 "pleomorphic adenoma*") OR (epithelial W/1 myoepithelial W/1 carcinoma* W/3 "salivary gland*"))<br>OR AUTHKEY((("salivary gland*" OR parotid OR "sublingual gland*" OR "submandibular gland*") W/3 (cancer* OR tumor* OR tumour* OR malignan* OR carcinoma*)) OR "salivary gland lymphadenoma*" OR "non-sebaceous lymphadenoma*" OR "sebaceous lymphadenoma*" OR "warthin tumor*" OR "warthin tumour*" OR (("squamous carcinoma*" OR "lymphoepithelial carcinoma*" OR adenocarcinoma* OR "mucinous adenocarcinoma*" OR "papillary cystadenocarcinoma*" OR "adenoid cystic carcinoma*" OR "mucoepidermoid carcinoma*" OR "acinar cell carcinoma*" OR "malignant mixed tumor*" OR "malignant mixed tumour*" OR "sebaceous adenocarcinoma*" OR cystadenocarcinoma* OR "ductal carcinoma*" OR "oncocytic carcinoma*") W/3 "salivary gland*") OR (myoepithelioma* W/3 malignan* W/3 "salivary gland*") OR (carcinoma* W/3 "pleomorphic adenoma*") OR (epithelial W/1 myoepithelial W/1 carcinoma* W/3 "salivary gland*"))<br>OR TITLE-ABS(((nasopharyn* OR rhinopharyn*) W/3 (carcinoma* OR adenocarcinoma* OR cancer*)) OR (nasopharyn* W/3 SCQ) OR "lymphoepithelioma*" OR (("epithelial tumor*" OR "epithelial tumour*" OR "squamous cell carcinoma*" OR SCC OR "squamous carcinoma*" OR "basaloid carcinoma*" OR "lymphoepithelial carcinoma*" OR "undifferentiated carcinoma*" OR "papillary adenocarcinoma*") W/3 nasopharyn*))<br>OR AUTHKEY(((nasopharyn* OR rhinopharyn*) W/3 (carcinoma* OR adenocarcinoma* OR cancer*)) OR (nasopharyn* W/3 SCQ) OR "lymphoepithelioma*" OR (("epithelial tumor*" OR "epithelial tumour*" OR "squamous cell carcinoma*" OR SCC OR "squamous carcinoma*" OR "basaloid carcinoma*" OR "lymphoepithelial carcinoma*" OR "undifferentiated carcinoma*" OR "papillary adenocarcinoma*") W/3 nasopharyn*))<br>OR TITLE-ABS(("middle ear" W/3 (cancer* OR tumor* OR tumour* OR malignan* OR schwannoma* OR meningioma* OR adenoma* OR paraganglioma* OR carcinoma*)) OR (("squamous cell carcinoma*" OR SCC OR "squamous carcinoma*" OR "lymphoepithelial carcinoma*" OR "verrucous carcinoma*" OR "giant cell carcinoma*" OR "adenosquamous carcinoma*" OR "undifferentiated carcinoma*") W/3 hypophary*) OR (("squamous cell carcinoma*" OR SCC OR "squamous carcinoma*" OR "verrucous carcinoma*" OR "adenosquamous carcinoma*" OR "undifferentiated carcinoma*" OR "lymphoepithelial carcinoma*" OR "giant cell carcinoma*") W/3 laryn*) OR (("epithelial tumor*" OR "epithelial tumour*" OR "squamous cell carcinoma*" OR SCC OR "squamous carcinoma*" OR "lymphoepithelial carcinoma*" OR "verrucous carcinoma*" OR	

"adenosquamous carcinoma*" OR "undifferentiated carcinoma*") W/3 oropharyn*) OR (("epithelial tumor*" OR "epithelial tumour*") W/3 ("oral cavit*" OR mouth* OR "lip*")) OR (("squamous cell carcinoma*" OR SCC OR "squamous carcinoma*" OR "verrucous carcinoma*" OR "adenosquamous carcinoma*" OR "lymphoepithelial carcinoma*" OR "undifferentiated carcinoma*") W/3 ("oral cavit*" OR mouth*)) OR (("squamous cell carcinoma*" OR SCC) W/3 lip*) OR (("epithelial tumor*" OR "epithelial tumour*" OR "squamous cell carcinoma*" OR SCC OR adenocarcinoma* OR "adenoid cystic carcinoma*") W/3 "middle ear*") OR "odontogenic malignant tumor*" OR "odontogenic malignant tumour*" OR "clear cell odontogenic carcinoma*" OR "ghost cell odontogenic carcinoma*" OR "olfactory neuroblastoma*")<br>OR AUTHKEY(("middle ear" W/3 (cancer* OR tumor* OR tumour* OR malignan* OR schwannoma* OR meningioma* OR adenoma* OR paraganglioma* OR carcinoma*)) OR (("squamous cell carcinoma*" OR SCC OR "squamous carcinoma*" OR "lymphoepithelial carcinoma*" OR "verrucous carcinoma*" OR "giant cell carcinoma*" OR "adenosquamous carcinoma*" OR "undifferentiated carcinoma*") W/3 hypophary*) OR (("squamous cell carcinoma*" OR SCC OR "squamous carcinoma*" OR "verrucous carcinoma*" OR "adenosquamous carcinoma*" OR "undifferentiated carcinoma*" OR "lymphoepithelial carcinoma*" OR "giant cell carcinoma*") W/3 laryn*) OR (("epithelial tumor*" OR "epithelial tumour*" OR "squamous cell carcinoma*" OR SCC OR "squamous carcinoma*" OR "lymphoepithelial carcinoma*" OR "verrucous carcinoma*" OR "adenosquamous carcinoma*" OR "undifferentiated carcinoma*") W/3 oropharyn*) OR (("epithelial tumor*" OR "epithelial tumour*") W/3 ("oral cavit*" OR mouth* OR "lip*")) OR (("squamous cell carcinoma*" OR SCC OR "squamous carcinoma*" OR "verrucous carcinoma*" OR "adenosquamous carcinoma*" OR "lymphoepithelial carcinoma*" OR "undifferentiated carcinoma*") W/3 ("oral cavit*" OR mouth*)) OR (("squamous cell carcinoma*" OR SCC) W/3 lip*) OR (("epithelial tumor*" OR "epithelial tumour*" OR "squamous cell carcinoma*" OR SCC OR adenocarcinoma* OR "adenoid cystic carcinoma*") W/3 "middle ear*") OR "odontogenic malignant tumor*" OR "odontogenic malignant tumour*" OR "clear cell odontogenic carcinoma*" OR "ghost cell odontogenic carcinoma*" OR "olfactory neuroblastoma*")<br>)<br>AND (<br>TITLE-ABS("Quality Of Life" OR HRQOL OR QOL)<br>OR AUTHKEY("Quality Of Life" OR HRQOL OR QOL)<br>)<br>AND (<br>EXCLUDE (DOCTYPE,"cr")<br>OR EXCLUDE (DOCTYPE,"cp")<br>)	

Domain 8 – Thorax

Query	Hits
(<br>TITLE-ABS(rare W/3 (thorax OR thoracic) W/3 (cancer* OR tumor* OR tumour* OR malignan*))<br>OR AUTHKEY(rare W/3 (thorax OR thoracic) W/3 (cancer* OR tumor* OR tumour* OR malignan*))<br>OR TITLE-ABS(((thymic OR thymus) W/3 (cancer* OR tumor* OR tumour* OR malignan* OR carcinoma*)) OR thymoma* OR (("epithelial tumor*" OR "epithelial tumour*" OR "squamous cell carcinoma*" OR SCC OR adenocarcinoma* OR "undifferentiated	569

carcinoma*") W/3 thym*) Or thymolipoma* OR "good syndrome" OR "good's syndrome")<br>OR AUTHKEY(((thymic OR thymus) W/3 (cancer* OR tumor* OR tumour* OR malignan* OR carcinoma*)) OR thymoma* OR (("epithelial tumor*" OR "epithelial tumour*" OR "squamous cell carcinoma*" OR SCC OR adenocarcinoma* OR "undifferentiated carcinoma*") W/3 thym*) Or thymolipoma* OR "good syndrome" OR "good's syndrome")<br>OR TITLE-ABS("pleura* mesothelioma*" OR ("squamous cell carcinoma*" W/3 ("small cell" OR "clear cell") W/3 (lung* OR pleura*)) OR ((adenocarcinoma* OR "mucinous adenocarcinoma*" OR "solid carcinoma*" OR "clear cell adenocarcinoma*" OR "acinar cell carcinoma*" OR "signet ring cell carcinoma*" OR "mucinous cystadenocarcinoma" OR "adenosquamous carcinoma*" OR "large cell carcinoma*" OR "mucoepidermoid carcinoma*" OR "adenoid cystic carcinoma*" OR "sarcomatoid carcinoma*" OR "pleomorphic carcinoma*" OR "giant cell carcinoma*") W/3 (lung* OR pleura*)) OR "bronchiolo-alveolar adenocarcinoma*" OR "bronchioloalveolar adenocarcinoma*" OR (("poorly differentiated" OR "small cell" OR "large cell") W/3 "endocrine carcinoma*" W/3 (lung* OR pleura*)) OR (epithelial W/1 myoepithelial W/1 carcinoma* W/3 (lung* OR pleura*)) OR ("giant cell carcinoma*" W/3 (lung* OR pleura*)) OR "pulmonary blastoma*")<br>OR AUTHKEY("pleura* mesothelioma*" OR ("squamous cell carcinoma*" W/3 ("small cell" OR "clear cell") W/3 (lung* OR pleura*)) OR ((adenocarcinoma* OR "mucinous adenocarcinoma*" OR "solid carcinoma*" OR "clear cell adenocarcinoma*" OR "acinar cell carcinoma*" OR "signet ring cell carcinoma*" OR "mucinous cystadenocarcinoma" OR "adenosquamous carcinoma*" OR "large cell carcinoma*" OR "mucoepidermoid carcinoma*" OR "adenoid cystic carcinoma*" OR "sarcomatoid carcinoma*" OR "pleomorphic carcinoma*" OR "giant cell carcinoma*") W/3 (lung* OR pleura*)) OR "bronchiolo-alveolar adenocarcinoma*" OR "bronchioloalveolar adenocarcinoma*" OR (("poorly differentiated" OR "small cell" OR "large cell") W/3 "endocrine carcinoma*" W/3 (lung* OR pleura*)) OR (epithelial W/1 myoepithelial W/1 carcinoma* W/3 (lung* OR pleura*)) OR ("giant cell carcinoma*" W/3 (lung* OR pleura*)) OR "pulmonary blastoma*")<br>OR TITLE-ABS((("epithelial tumor*" OR "epithelial tumour*" OR "squamous cell carcinoma*" OR SCC OR "adenosquamous carcinoma*" OR "verrucous carcinoma*" OR "undifferentiated carcinoma*" OR "spindle cell carcinoma*" OR "lymphoepithelial carcinoma*" OR "giant cell carcinoma*" OR adenocarcinoma* OR "mucinous adenocarcinoma*" OR "papillary adenocarcinoma*" OR "solid carcinoma*" OR "clear cell adenocarcinoma*" OR "signet ring cell carcinoma*" OR "mucinous cystadenocarcinoma" OR "adenoid cystic carcinoma*" OR "mucoepidermoid carcinoma*") W/3 trachea*) OR (myoepithelial W/1 carcinoma* W/3 trachea*))<br>OR AUTHKEY((("epithelial tumor*" OR "epithelial tumour*" OR "squamous cell carcinoma*" OR SCC OR "adenosquamous carcinoma*" OR "verrucous carcinoma*" OR "undifferentiated carcinoma*" OR "spindle cell carcinoma*" OR "lymphoepithelial carcinoma*" OR "giant cell carcinoma*" OR adenocarcinoma* OR "mucinous adenocarcinoma*" OR "papillary adenocarcinoma*" OR "solid carcinoma*" OR "clear cell adenocarcinoma*" OR "signet ring cell carcinoma*" OR "mucinous cystadenocarcinoma" OR "adenoid cystic carcinoma*" OR "mucoepidermoid carcinoma*") W/3 trachea*) OR (myoepithelial W/1 carcinoma* W/3 trachea*))<br>OR TITLE-ABS((classic W/3 "invasive lobular carcinoma*" W/3 (breast* OR mamma*)) OR (("pleomorphic carcinoma*" OR "paget's disease" OR "tubular adenocarcinoma*" OR "mucinous carcinoma*" OR "medullary carcinoma*" OR "papillary adenocarcinoma*" OR "cribriform carcinoma*" OR "apocrine adenocarcinoma*" OR "secretory carcinoma*" OR "glycogen-rich carcinoma*" OR "lipid-rich carcinoma*" OR "oncocytic carcinoma*" OR	

"metaplastic carcinoma*" OR "squamous carcinoma*" OR "adenosquamous carcinoma*" OR "mucoepidermoid carcinoma*" OR "adenoid cystic carcinoma*" OR "acinar cell carcinoma*") W/3 (breast* OR mamma*)) OR (adenocarcinoma* W/3 (cartil* OR osseous) W/3 metaplas* W/3 (breast* OR mamma*)) OR (myoepithelial W/1 carcinoma* W/3(breast* OR mamma*)) OR (malignan* W/3 mesothelioma*) OR (mesothelioma* W/3 (pleura OR pericardium)) OR (mesothelioma* W/3 (peritoneum OR "tunica vaginalis")) OR (pleuropulmonary W/3 blastoma*))<br>OR AUTHKEY((classic W/3 "invasive lobular carcinoma*" W/3 (breast* OR mamma*)) OR (("pleomorphic carcinoma*" OR "paget's disease" OR "tubular adenocarcinoma*" OR "mucinous carcinoma*" OR "medullary carcinoma*" OR "papillary adenocarcinoma*" OR "cribriform carcinoma*" OR "apocrine adenocarcinoma*" OR "secretory carcinoma*" OR "glycogen-rich carcinoma*" OR "lipid-rich carcinoma*" OR "oncocytic carcinoma*" OR "metaplastic carcinoma*" OR "squamous carcinoma*" OR "adenosquamous carcinoma*" OR "mucoepidermoid carcinoma*" OR "adenoid cystic carcinoma*" OR "acinar cell carcinoma*") W/3 (breast* OR mamma*)) OR (adenocarcinoma* W/3 (cartil* OR osseous) W/3 metaplas* W/3 (breast* OR mamma*)) OR (myoepithelial W/1 carcinoma* W/3(breast* OR mamma*)) OR (malignan* W/3 mesothelioma*) OR (mesothelioma* W/3 (pleura OR pericardium)) OR (mesothelioma* W/3 (peritoneum OR "tunica vaginalis")) OR (pleuropulmonary W/3 blastoma*))<br>)<br>AND (<br>TITLE-ABS("Quality Of Life" OR HRQOL OR QOL)<br>OR AUTHKEY("Quality Of Life" OR HRQOL OR QOL)<br>)<br>AND (<br>EXCLUDE(DOCTYPE,"cr")<br>OR EXCLUDE(DOCTYPE,"cp")<br>)	

Domain 9 - Skin and eye melanoma

**Query**	**Hits**
(<br>TITLE-ABS(rare W/3 (skin OR cutan*) W/3 (cancer* OR tumor* OR tumour* OR malignan* OR carcinoma*))<br>OR AUTHKEY(rare W/3 (skin OR cutan*) W/3 (cancer* OR tumor* OR tumour* OR malignan* OR carcinoma*))<br>OR TITLE-ABS((uvea* W/3 melanoma*) OR "iridociliary melanoma*" OR "irido-ciliary melanoma*" OR (melanoma* W/3 (eye* OR ciliary OR iris OR conjunctiva)) OR "choroid melanoma*" OR "choroidal melanoma*")<br>OR AUTHKEY((uvea* W/3 melanoma*) OR "iridociliary melanoma*" OR "irido-ciliary melanoma*" OR (melanoma* W/3 (eye* OR ciliary OR iris OR conjunctiva)) OR "choroid melanoma*" OR "choroidal melanoma*")<br>OR TITLE-ABS(("merkel cell" W/1 (carcinoma* OR tumor* OR tumour* OR cancer*)) OR "neuroendocrine carcinoma of the skin" OR "cutaneous neuroendocrine carcinoma*" OR "trabecular cancer*" Or "cutaneous apudoma*")<br>OR AUTHKEY(("merkel cell" W/1 (carcinoma* OR tumor* OR tumour* OR cancer*)) OR "neuroendocrine carcinoma of the skin" OR "cutaneous neuroendocrine carcinoma*" OR "trabecular cancer*" Or "cutaneous apudoma*")<br>OR TITLE-ABS("mucosal melanoma*" OR ((mucous OR extracutaneous) W/3 melanoma*) OR (("basal cell carcinoma*" OR "basosquamous carcinoma*" OR "squamous	268

carcinoma*" OR "verrucous carcinoma*" OR "spindle cell carcinoma*" OR "adenosquamous carcinoma*" OR "adnexal carcinoma*" OR "adenoid cystic carcinoma*" OR "paget disease" OR "apocrine adenocarcinoma*" OR "mucinous adenocarcinoma*" OR "pillomatrix carcinoma*" OR "tubular adenocarcinoma*") W/3 (skin OR cutan* OR dermal)) OR ((adenoid OR pseudovascular OR "pseudo vascular") W/3 "squamous cell carcinoma*" W/3 (skin OR cutan* OR dermal)) OR (("nodular hidradenoma" OR "mixed tumor*" OR "mixed tumour*") W/3 malignant W/3 (skin OR cutan* OR dermal)) OR "sebaceous adenocarcinoma*" OR "eccrine porocarcinoma" OR (sclerosing W/3 "sweat duct*" W/3 carcinoma*) OR ("eccrine spiradenoma" W/3 malignant) OR (eccrine W/3 papillary W/3 adenocarcinoma*) OR (("superficial spreading" OR nodular OR "lentigo maligna") W/3 melanoma*) OR ("acral lentiginous" W/3 melanoma* W/3 malignan*) OR (("epithelial tumor*" OR "epithelial tumour*" OR "squamous cell carcinoma*" OR SCC OR "squamous carcinoma*" OR "basal cell carcinoma*" OR adenocarcinoma* OR "adenoid cystic carcinoma*" OR "mucoepidermoid carcinoma*" OR "embryonal tumor*" OR "embryonal tumour*") W/3 (eye* OR adnexa*)) OR retinoblastoma*)<br>OR AUTHKEY("mucosal melanoma*" OR ((mucous OR extracutaneous) W/3 melanoma*) OR (("basal cell carcinoma*" OR "basosquamous carcinoma*" OR "squamous carcinoma*" OR "verrucous carcinoma*" OR "spindle cell carcinoma*" OR "adenosquamous carcinoma*" OR "adnexal carcinoma*" OR "adenoid cystic carcinoma*" OR "paget disease" OR "apocrine adenocarcinoma*" OR "mucinous adenocarcinoma*" OR "pillomatrix carcinoma*" OR "tubular adenocarcinoma*") W/3 (skin OR cutan* OR dermal)) OR ((adenoid OR pseudovascular OR "pseudo vascular") W/3 "squamous cell carcinoma*" W/3 (skin OR cutan* OR dermal)) OR (("nodular hidradenoma" OR "mixed tumor*" OR "mixed tumour*") W/3 malignant W/3 (skin OR cutan* OR dermal)) OR "sebaceous adenocarcinoma*" OR "eccrine porocarcinoma" OR (sclerosing W/3 "sweat duct*" W/3 carcinoma*) OR ("eccrine spiradenoma" W/3 malignant) OR (eccrine W/3 papillary W/3 adenocarcinoma*) OR (("superficial spreading" OR nodular OR "lentigo maligna") W/3 melanoma*) OR ("acral lentiginous" W/3 melanoma* W/3 malignan*) OR (("epithelial tumor*" OR "epithelial tumour*" OR "squamous cell carcinoma*" OR SCC OR "squamous carcinoma*" OR "basal cell carcinoma*" OR adenocarcinoma* OR "adenoid cystic carcinoma*" OR "mucoepidermoid carcinoma*" OR "embryonal tumor*" OR "embryonal tumour*") W/3 (eye* OR adnexa*)) OR retinoblastoma*)<br>)<br>AND (<br>TITLE-ABS("Quality Of Life" OR HRQOL OR QOL)<br>OR AUTHKEY("Quality Of Life" OR HRQOL OR QOL)<br>)<br>AND (<br>EXCLUDE(DOCTYPE,"cr")<br>OR EXCLUDE(DOCTYPE,"cp")<br>)	

Domain 10 – Brain and spinal Cord

Query	Hits
(<br>TITLE-ABS(rare W/3 (brain OR "spinal cord" OR "nervous system") W/3 (cancer* OR tumor* OR tumour* OR malignan* OR carcinoma*))<br>OR AUTHKEY(rare W/3 (brain OR "spinal cord" OR "nervous system") W/3 (cancer* OR tumor* OR tumour* OR malignan* OR carcinoma*))	2137

OR TITLE-ABS((neuroglial W/3 (tumor* OR tumour*)) OR "glioneural tumor*" OR "glioneural tumour*" OR "neuronal-glial tumor*" OR "neuronal-glial tumour*" OR glioma* OR (glial W/3 (tumor* OR tumour*)) OR astrocytoma* OR astroglioma* OR oligoastrocytoma* OR glioblastoma* OR "pleomorphic xanthoastrocytoma*" OR ependymoma* OR ependymoblastoma* OR subependymoma* OR "ependymal tumor*" OR "ependymal tumour*" OR ganglioglioma* OR gliosarcoma* OR oligodendroglioma* OR oligodendrocytoma* OR (astrocytic W/3 (tumor* OR tumour*)) OR astroblastoma* OR "gliomatosis cerebri" OR (oligodendroglioma* W/3 (tumor* OR tumour*)) OR "choroid plexus carcinoma*" OR ("meningioma*" W/3 malignan*) OR pinealoma* OR ((tumor* OR tumour*) W/3 pineal) OR pineoblastoma* OR (("embryonal tumor*" OR "embryonal tumour*") W/3 ("central nervous system" OR CNS)) OR "atypical teratoid tumor*" OR "atypical teratoid tumour*" OR neuroepithelioma*)<br>OR AUTHKEY((neuroglial W/3 (tumor* OR tumour*)) OR "glioneural tumor*" OR "glioneural tumour*" OR "neuronal-glial tumor*" OR "neuronal-glial tumour*" OR glioma* OR (glial W/3 (tumor* OR tumour*)) OR astrocytoma* OR astroglioma* OR oligoastrocytoma* OR glioblastoma* OR "pleomorphic xanthoastrocytoma*" OR ependymoma* OR ependymoblastoma* OR subependymoma* OR "ependymal tumor*" OR "ependymal tumour*" OR ganglioglioma* OR gliosarcoma* OR oligodendroglioma* OR oligodendrocytoma* OR (astrocytic W/3 (tumor* OR tumour*)) OR astroblastoma* OR "gliomatosis cerebri" OR (oligodendroglioma* W/3 (tumor* OR tumour*)) OR "choroid plexus carcinoma*" OR ("meningioma*" W/3 malignan*) OR pinealoma* OR ((tumor* OR tumour*) W/3 pineal) OR pineoblastoma* OR (("embryonal tumor*" OR "embryonal tumour*") W/3 ("central nervous system" OR CNS)) OR "atypical teratoid tumor*" OR "atypical teratoid tumour*" OR neuroepithelioma*)<br>OR TITLE-ABS((medulloblastoma* OR "medullo blastoma*") AND (adult* OR aged OR elderly OR postpubertal OR "post pubertal"))<br>OR AUTHKEY((medulloblastoma* OR "medullo blastoma*") AND (adult* OR aged OR elderly OR postpubertal OR "post pubertal"))<br>)<br>AND (<br>TITLE-ABS("Quality Of Life" OR HRQOL OR QOL)<br>OR AUTHKEY("Quality Of Life" OR HRQOL OR QOL)<br>)<br>AND (<br>EXCLUDE(DOCTYPE,"cr")<br>OR EXCLUDE(DOCTYPE,"cp")<br>)	
